# Nickel-catalyzed intermolecular oxidative Heck arylation driven by transfer hydrogenation

**DOI:** 10.1038/s41467-019-12949-1

**Published:** 2019-11-05

**Authors:** Honggui Lv, Huiying Kang, Biying Zhou, Xiaosong Xue, Keary M. Engle, Dongbing Zhao

**Affiliations:** 1 State Key Laboratory and Institute of Elemento-Organic Chemistry, College of Chemistry, Nankai University, Tianjin, 300071 China; 2 Department of Chemistry, The Scripps Research Institute, 10550 North Torrey Pines Road, La Jolla, CA 92037 USA

**Keywords:** Catalytic mechanisms, Homogeneous catalysis, Synthetic chemistry methodology

## Abstract

The conventional oxidative Heck reaction between aryl boronic acids and alkenes typically involved the Pd^II^/Pd^0^/Pd^II^ catalytic cycle incorporating an external oxidant and often suffered C=C bond isomerization for internal alkyl-substituted alkenes via chain-walking. Herein, we demonstrate that the regioselectivity (γ-selectivity vs. δ-selectivity) and pathway selectivity (hydroarylation vs. oxidative Heck coupling) of a directed Ni-catalyzed alkene arylation can be controlled by judicious tuning of the coordination environment around the nickel catalyst via optimization of an appropriate phosphine ligand and directing group. In this way, the Ni(0)-catalyzed oxidative Heck arylation that relies on transfer hydrogenation of an acceptor olefin is developed with excellent *E*/*Z* selectivity and regioselectivity. Mechanistic investigations suggest that the addition of the acceptor is crucial for lowering the energy for carbometalation and for enabling catalytic turnover.

## Abstract (web summary)

Conventional palladium-catalyzed Heck reactions rely on the addition of oxidants and may suffer from low selectivity. Here, the authors report a nickel-catalyzed oxidative Heck arylation driven by transfer hydrogenation of an acceptor olefin leading to high *E/Z* selectivity and regioselectivity.

## Introduction

**Fig. 1:**
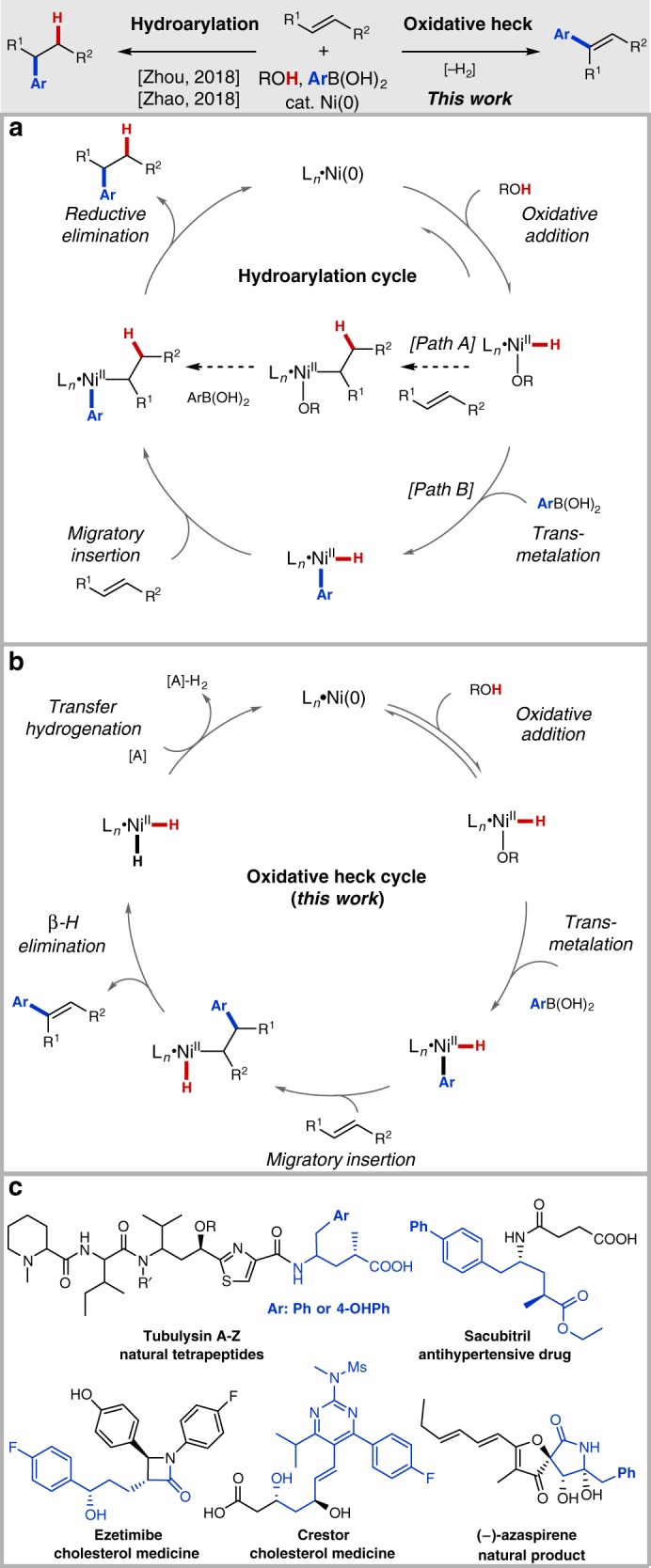
Inspiration and motivation for the present study. **a** Catalytic cycle for previously reported Ni-catalyzed hydroarylation reaction. **b** Envisioned oxidative Heck reaction. **c** Relevant biologically active compounds containing γ-aryl carboxylic acid substructure

Transition-metal-catalyzed cross-coupling reactions involving alkenes enable conversion of abundant feedstocks to value-added products through C–C bond formation^1–16^. Heck-type reactions, which can be carried out both in classical polarity and oxidative modes, are the archetypal method for arene–alkene coupling. Despite significant advances during the past several decades^1–10^, Heck-type reactions still suffer from significant limitations, including a difficulty controlling selectivity with internal alkyl-substituted alkenes due to the lack of steric and electronic differentiation in the key migratory insertion step and issues of C=C bond isomerization due to chain-walking^17^. Additionally, though notable exceptions exist^18,19^, oxidative Heck reactions with arylmetal species have traditionally required use of comparatively expensive palladium catalysts with specially tailored reoxidation systems^20–26^. Recently, Zhou reported a highly selective Ni-catalyzed hydroarylation of styrenes and 1,3-dienes with arylboron compounds, which was proposed to proceed via oxidation addition of Ni(O) to an alcohol O–H bond to form a Ni–H species, hydrometallation across the alkene, transmetalation with the organoboron reagents, and C–C reductive elimination (Fig. 1a, path A)^27,28^. In 2018, we extended this strategy to hydroarylation of non-conjugated alkenes by introducing a directing group on the substrate^29–35^. During the course of this study, we detected byproducts arising from hydrogenolysis of the Ar–B bond, indicating that transmetalation might precede migratory insertion. In this case, a Ni(H)(Ar) intermediate would be the active form of the catalyst that engages the alkene (Fig. 1a, path B). This catalytic paradigm offers exciting possibilities for controlling regioselectivity and pathway selectivity to access different products from a common set of reagents.

We were attracted to the possibility of diverting this process to obtain non-chain-walking oxidative-Heck products by using internal alkyl-substituted alkenes and aryl boronates as the coupling partners. In the proposed cycle (Fig. 1b), migratory insertion of the aryl group in preference to the hydride group would first take place. Subsequently, β-hydride elimination would then furnish the arylated alkene product along with a Ni(H)_2_ species that would reduce a sacrificial alkene to close the catalytic cycle. If successful, this sequence of events would result in a Ni(O)-catalyzed oxidative.

Heck reaction coupled to a transfer hydrogenation process^36–44^. We envisioned that selectivity for this pathway over other alternatives could be achieved by tuning the coordination environment around nickel with an appropriate phosphine ligand and directing group^45–52^. Herein we describe the realization of this goal. In particular, we report complementary transformations for both γ-selective hydroarylation and oxidative Heck coupling. These methods are efficient and scalable, providing a modular means of assembling δ-aryl olefinic acids, which are versatile building blocks for synthesizing drugs and other biologically active compounds (Fig. 1c)^53–57^.

## Results

### Condition screening

In a pilot experiment, 4-pentenoic acid masked with the 8-aminoquinoline (AQ) directing group (**1a**, 0.2 mmol) was subjected to our reported conditions for hydroarylation of 3-butenoic acid derivatives^29,58,59^, which gave a complicated mixture of hydoarylation and Heck-type products (Table 1, entry 1). Next, various phosphine ligands were evaluated, and the effects were pronounced (entries 2–14). With PCy_3_ or P*n*Bu_3_ as the ligand, the *δ*-selective Heck-type product **5a** was the major product, albeit in low yield (entries 2-3). Surprisingly, use of bulkier phosphine ligands resulted in exclusive formation of the δ-selective hydroarylation product **3a** (entries 4–5). In contrast, when PMe_3_ was used as the ligand, only γ-selective hydroarylation product **4a** was observed in 85% yield. Notably, the γ-selectivity of this directed hydroarylation protocol is opposite to that of Loh’s Pd-catalyzed reductive Heck system, which is δ-selective for this type of substrate^60^. Using PCy_3_ or P*n*Bu_3_ as ligand, we found that adjusting the molar ratio of **1a** and **2a** to 1.5:1 dramatically increased the yield of oxidative Heck product **5a** (entries 15–16). Notably, the reduced form of alkene **1a** was detected in all the reactions, suggesting that **1a** was serving as an H_2_ acceptor. Based on this insight, we systematically examined various hydrogen acceptors so that alkene **1a** could be used as the limiting reagent. Pleasingly, after an exhaustive screen of alkene acceptors, we identified cinnamaldehyde derivative **A18** (1.0 equiv) as an efficient sacrificial alkene, and with this additive, 1 equiv **1a** could be converted to **5a** in 94% isolated yield.

**Table 1:** Reaction optimization


Entry	Ligand	Yield[%]^a^ (3a:4a:5a:6a)	Entry	Ligand	Yield[%]^a^ (3a:4a:5a:6a)
1	PPh_3_	47 (0/17/25/5)	9	PPhCy_2_	43 (0/10/29/4)
2	PCy_3_	41 (0/0/35/6)	10	P(OMe)_3_	30 (0/14/16/0)
3	P*n*Bu_3_	39 (0/9/30/0)	11	P*n*Pr_3_	29 (0/13/16/0)
4	P*t*Bu_3_	25 (25/0/0/0)	12	PMe_2_Ph	32 (0/25/7/0)
5	XPhos	35 (35/0/0/0)	13	P*t*BuCy_2_	32 (0/15/17/0)
6	PMe_3_	85 (0/85/0/0)	14	Xantphos	38 (0/16/19/3)
7	PPh_2_Cy	44 (0/17/22/5)	15^b^	PCy_3_	79 (0/0/79/0)
8	DCYPE	0	16^b^	P*n*Bu_3_	92 (0/0/92/0)


Reactions conditions: Ni(cod)_2_ (10 mol%), ligand (20 mol%), CsOPiv (1.5 equiv), **1a** (0.2 mmol), **2a** (0.4 mmol), *t*-AmylOH (1 mL), 70 °C, 48 h

^a1^H NMR yields with C_2_H_2_Br_4_ as internal standard

^b^Molar ratio of **1a**:**2a** = 1.5:1

^c^P*n*Bu_3_ (20 mol%), H_2_ scavenger (2.0 equiv). The ^1^H NMR yield of **5a** is given

^d^1.0 equiv H_2_ acceptor

^e^The isolated yield is given in parenthesis

### Substrate scope of hydroarylation

Given the opposite selectivity of our Ni-catalytic system compared to Loh’s Pd-catalyzed reductive Heck system for hydroarylation of 4-pentenoic acids^60^, we next briefly probed the substrate scope of the γ-selective hydroarylation (Table 2). A diverse collection of arylboronic acids could be coupled to **1a**, furnishing the corresponding γ-arylated products in good to excellent yields. Cyclic alkenes and δ,δ-disubstituted alkenes also underwent hydroarylation, though in these cases the reactions proceeded with δ-selectivity. Notably, these substrates are unreactive in Loh’s Pd-catalyzed system.

**Table 2:** Substrate scope of hydroarylation

Reaction conditions: Ni(cod)_2_ (10 mol%), PMe_3_ (20 mol%), CsOPiv (1.5 equiv), alkene **1** (0.2 mmol), arylboronic acid **2** (0.4 mmol), *t*-AmylOH (1 mL), 70 °C, 48 h. Values represent isolated yields

^a^The isolated yield in parenthesis was obtained by use of Ni(cod)_2_ (5 mol%) and PMe_3_ (10 mol%)

^b^120 °C

^c^P*n*Bu_3_ as ligand

### Substrate scope of oxidative Heck reaction

Next, we investigated the substrate scope and functional group tolerance of the oxidative Heck reaction with respect to the organoboron reagent using **1a** as the model alkene substrate (Table 3). To our delight, a wide variety of *para-* and *meta*-substituted arylboronic acids with different electronic properties afforded the desired products in good to excellent yields and with high *E*-selectivity (**5ae–ao**). *ortho*-Substituted arylboronic acids were slightly lower yielding, presumably due to steric encumbrance (**5ac**−**ad**). Multi-substituted arylboronic acids (**5ap**−**ar**) and heteroarylboronic acids (**5as**−**av**) also provided the corresponding Heck products in high yields. Additionally, cyclic and acyclic alkenyl boronic acids were competent coupling partners, enabling access to conjugated dienoic acid derivatives (**5aw**−**ax**). Importantly, this method was found to be compatible with an array of functional groups, including halo, acetyl, alkoxycarbonyl, cyano, and methoxy substituents, and could be carried out on gram scale (1.14 g of **5ab**, 84%). The structure of **5a** was confirmed by single-crystal X-ray diffraction.

**Table 3:** Scope of organoboronic acids and alkenes in oxidative Heck arylation

Reaction conditions: Ni(cod)_2_ (10 mol%), ligand (20 mol%), CsOPiv (1.5 equiv), alkene (0.2 mmol), arylboronic acid (0.4 mmol), *t*-AmylOH (1 mL), 70 °C, 48 h. Values correspond to isolated yields. In cases where the *E*/*Z* ratio is not specified, only the *E*-alkene product was observed

^a^The values parentheses represent the isolated yield using Ni(cod)_2_ (5 mol%) and ligand (10 mol%)

^b^PMe_3_ (20 mol%), 120 °C

^c^*t*-AmylOH (1.5 mL)

^d^*Z*/*E* ratios were determined by ^1^H NMR

^e^The *Z*/*E* ratio of substrate **1i** was 91:9

^f^Ni(cod)_2_ (20 mol%), P*n*Bu_3_ (40 mol%), 120 °C, 72 h

We then evaluated various γ,δ-unsaturated carboxamides (Table 3). Terminal alkenes bearing a single substituent at the α- or β-position reacted smoothly to afford the desired products with the complete *E*-selectivity (**5ba**−**ea**). The β,β-disubstituted terminal alkene was also reactive, selectively furnishing desired product **5fa**. In contrast, the sterically more congested α,α-disubstituted terminal alkene only gave 36% yield, even after brief optimization of the conditions (**5ga**). (*E*)-Internal alkenes bearing an alkyl group at the δ-position could also be efficiently converted into the corresponding *E*- configured products (**5ha**–**ka**). However, aryl substitution at the δ-position led to clear attenuation of reactivity (**5la**). Notably, when (*Z*)-alkene **1i** was used, the reaction stereospecifically generated (*Z*)-product **5ia**. In addition, the reaction was also compatible with β-substituted internal alkenes (**5ma**–**na**), a γ-substituted terminal alkene (**5oa**), and an α,δ-disubstituted alkene (**5pa**), though in the latter two cases *Z*/*E* selectivity was low. In addition to γ,δ-unsaturated substrates, δ,ε-unsaturated AQ carboxamides were also examined. Surprisingly, with this substrate class, the connectivity of the products was the same as with the (*E*)-γ,δ-unsaturated internal alkenes (**5qa**–**qd**) suggesting potential alkene isomerization. A representative internal δ,ε-unsaturated alkene was unreactive in this isomerization/oxidative Heck cascade reaction.

### Synthetic utility

**Fig. 2:**
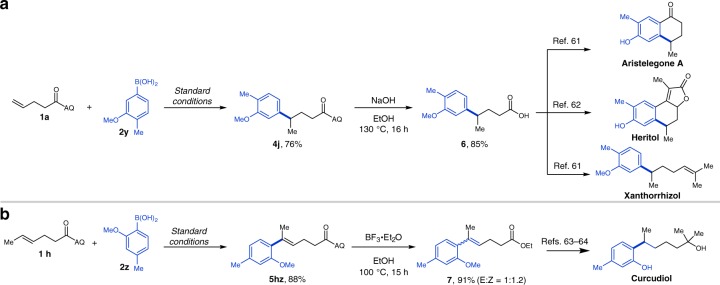
Synthesis of key intermediates towards natural products. **a** The application of hydroarylation to approach the key intermediates in the synthesis of the natural products. **b** The application of oxidative Heck coupling toward the key intermediate in the synthesis of the natural product curcudiol

To demonstrate the synthetic utility of our method, we first sought to prepare carboxylic acid **4j**, a key intermediate in the synthesis of the natural products aristelegone A, heritol, and xanthorrhizol (Fig. 2, upper panel)^61,62^. This intermediate has previously been synthesized through a sequence involving condensation followed by Michael addition and decarboxylation. As a second example, unsaturated carboxylic ester **7** was also prepared via δ-selective oxidative Heck arylation. Compound **7** has been employed as a key intermediate in the synthesis of the natural product curcudiol (Fig. 2, lower panel)^63,64^. The literature method to access **7** involves a multi-step sequence including Grignard addition and Johnson–Claisen rearrangement.

## Discussion

### Mechanism investigation

**Fig. 3:**
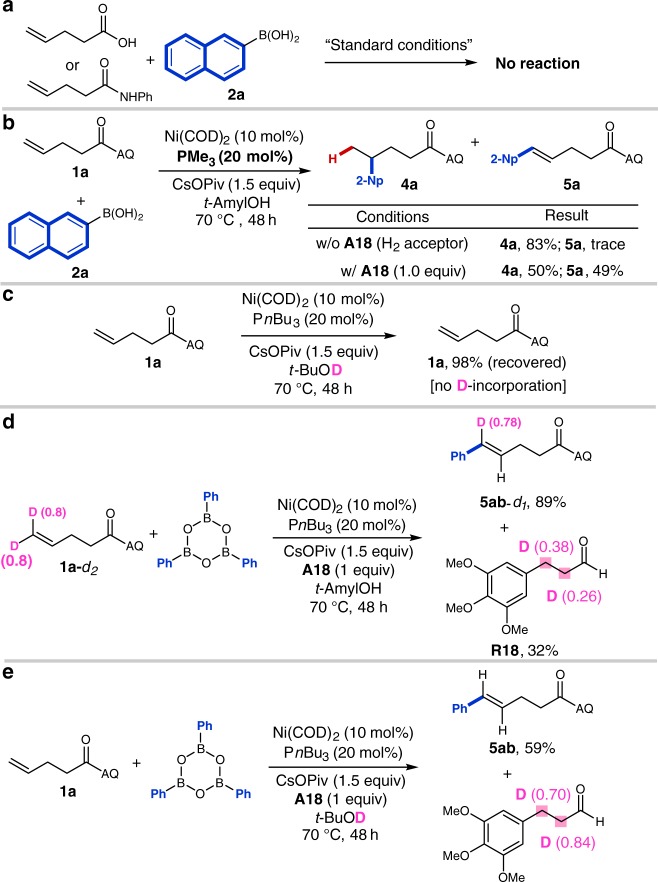
Experiments to elucidate Heck coupling mechanism. **a** Control experiments establishing the need for the AQ-directing group in both hydroarylation and Heck coupling. **b** The role of the compound **A18**. **c** The importance of aryl boronic acid in alkene insertion. **d** The reaction with δ,δ-di-deuterated alkene **1a**-*d*_*2*_. **e** Deuteration experiments in *t*-BuOD

We then carried out a series of experiments to elucidate the mechanism of the directed oxidative Heck reaction. As an initial control experiments, we first subjected two representative substrates lacking the AQ directing group, pent-4-enoic acid and *N*-phenylpent-4-enamide, to the standard reaction conditions using 2-naphthalenylboronic acid as the coupling partner (Fig. 3a). In these experiments, no reaction took place, indicating that the AQ directing group on the olefin substrate is indispensable for reactivity. Next, to probe the formation of putative Ni–H species, we performed a stoichiometric reaction between *t*-amylOH (1.0 equiv.) and Ni(COD)_2_ (1.0 equiv.) in C_6_D_6_ at r.t. and analyzed the crude reaction mixture by ^1^H NMR. We observed a new peak at −23.24 ppm, which is consistent with previously described Ni–H species in the literature^65–67^. Several additional experiments were then performed under catalytic conditions. First, we found that the addition of hydrogen acceptor **A18** (1.0 equiv) under conditions that were otherwise identical to the optimal conditions for γ-selective hydroarylation resulted in a significant increase in the yield of Heck product **5a** (Fig. 3b). This result indicates that compound **A18** may play a role beyond serving as a hydrogen acceptor. In particular, this additive could be bound to nickel as a ligand during the key migratory insertion event and could lower the activation energies for carbometalation compared to hydrometalation (see below). Second, we exposed alkene **1a** to the reaction conditions in *t-*BuOD and were unable to detect deuterium incorporation (Fig. 3c). The result is consistent with a scenario in which [Ni(H)(OR)] is inactive in alkene insertion until it undergoes transmetalation with arylboronic acid to give an active [Ni(H)(Ar)] species. Third we performed the reaction with δ,δ-di-deuterated alkene **1a**-*d*_*2*_. The product, **5ab-***d*_1_ (89% yield), contained a single deuterium atom at the δ position (Fig. 3d). In addition, it was found that the reduced acceptor **R18** was partially deuterated at both the α- and β-positions (Fig. 3d). These results are consistent with an irreversible β-H elimination step followed by transfer hydrogenation to acceptor **A18**. Last, we performed the standard reaction in *t*-BuOD (Fig. 3e). There was no deuterium incorporation in the Heck product **5ab**. Instead, the deuterium was transfered to the α- and β-positions of reduced acceptor **R18** implying that: (1) hydrometalation of alkene **1a** does not happen under the oxidative Heck reaction conditions; (2) hydrogen is transferred from *t*-BuOH to compound **A18** as part of catalyst turnover.

**Fig. 4:**
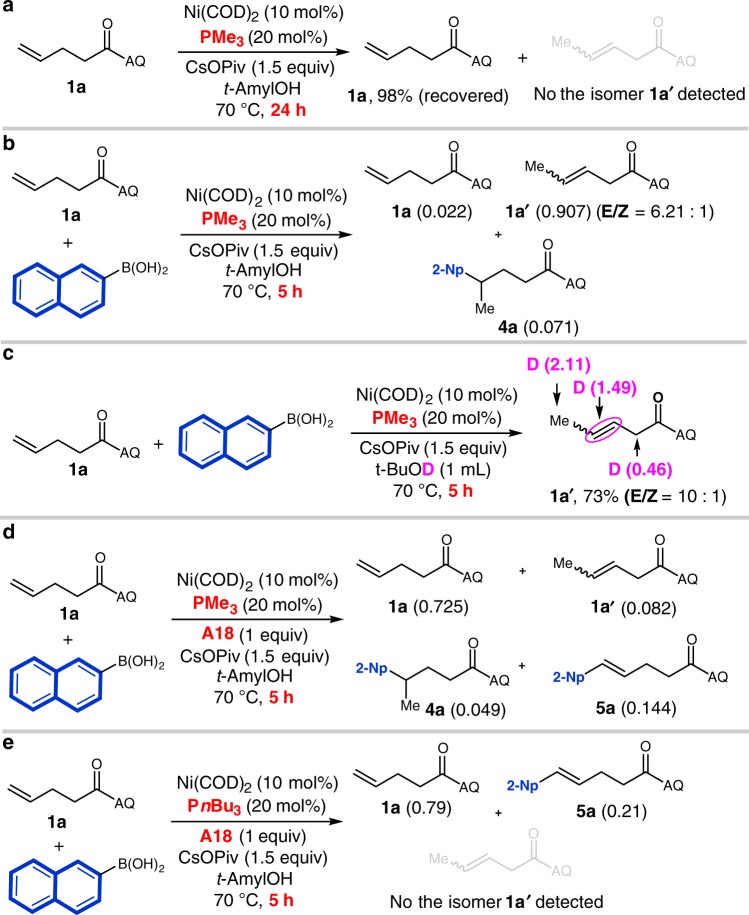
A series of experiments to study the mechanism of the γ-selective hydroarylation. **a** The control experiment in the absence of arylboronic acid. **b** The monitoring of the alkene substrate in hydroarylation for 5 h. **c** Deuterium incorporation into isomerized alkene. **d** The hydroarylation reaction in the presence of the compound **A18** for 5 h. **e** The reaction by use of P*n*Bu_3_ as the ligand for 5 h

To gain further insight into mechanism and the turnover-limiting step, we examined reaction kinetics at various initial concentrations of alkene **1a**, aryl boronic acid **2o**, acceptor **A18**, and Ni(COD)_2_ (See Supplementary Figs. 12–19). Based on initial rates, the reaction is first-order with respect to the Ni^0^ catalyst and zeroth-order with respect to the alkene **1a**, aryl boronic acid **2o**, and the acceptor **A18**. These results are consistent with turnover-limiting migratory insertion from a complex of the general form, [Ni(Ar)(**1a**)(**A18**)], as discussed more below. Additionally, we also performed a brief series of experiments to study the mechanism of the γ-selective hydroarylation (Fig. 4). First, we found that the alkene **1a** is unreactive in the absence of arylboronic acid under the hydroarylation conditions (Fig. 4a). However, in the presence of arylboronic acid **2a**, most of the terminal alkene **1a** is isomerized to internal alkene **1a’** after 5 h under the hydroarylation conditions (Fig. 4b). In addition, we exposed alkene **1a** to hydroarylation conditions in *t-*BuOD for 5 h. Deuterium incorporation was detected at all four positions of isomerized alkene **1a’** (Fig. 4c). Several conclusions can be drawn from these results: (1) [Ni(H)(OR)] is unreactive in alkene insertion in the absence of arylboronic acid; (2) [Ni(H)(Ar)] species promotes isomerization of alkene **1a**; (3) internal alkene **1a’** is likely an intermediate for this γ-selective hydroarylation; and (4) isomerization of alkene **1a** is reversible. Furthermore, we find that the addition of the hydrogen acceptor **A18** in the hydroarylation reaction for 5 h partially inhibits the isomerization of alkene **1a** and initiates the oxidative Heck arylation (Fig. 4d). The isomerization of alkene **1a** does not occur under the Heck reaction conditions (Fig. 4e).

### DFT calculations

**Fig. 5:**
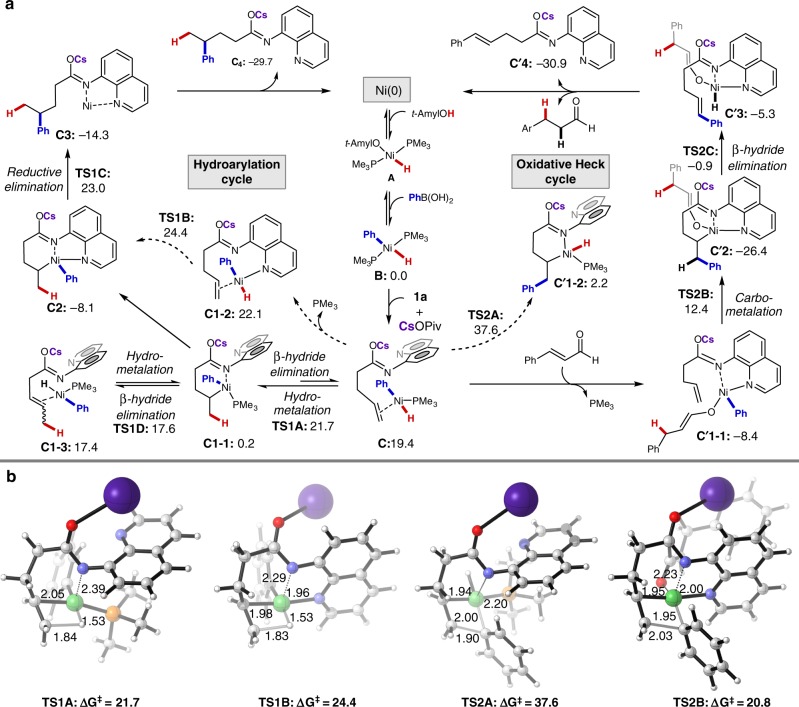
DFT calculations for reaction mechanism. **a** Proposed catalytic cycle with free energies computed by DFT (kcal mol^−1^). **b** Structures of transition states **TS1A**, **TS1B**, **TS2A**, and **TS2B**

On the basis of literature reports and our own mechanistic experiments, we propose a catalytic cycle that begins with oxidation addition of Ni(O) to an alcohol O–H bond to form intermediate **A**. This is followed by transmetalation of the arylboronic acid, which affords the key [Ni(Ar)(H)] species (Fig. 5). An alternative sequence involving initial coordination of Ni(O) to the AQ group and subsequent O–H oxidation addition was also considered; however, this pathway was found to be much higher in energy (see Supplementary Fig. 21). To understand the underlying origin of the divergent reactivity, DFT calculations were performed using alkene **1a** and phenylboronic acid (**2b**) as model reactants (Fig. 5). First, starting from [Ni(Ar)(H)] species **B**, ligand exchange of one PMe_3_ for **1a** in the presence of CsOPiv delivers complex **C**. Then, the reaction can proceed along two divergent pathways: Pathway I (hydroarylation; left) or Pathway II (oxidative Heck coupling; right). In Pathway I, **C** undergoes γ-selective hydrometalation via **TS1A**. The resulting six-membered nickelacycle **C1-1** either directly loses a second PMe_3_ ligand upon coordination of the quinoline group to form a more stable species **C2** or undergoes β-hydride elimination to form **C1-3** via **TS1D**. The other pathway to form **C2** via **TS1B** is also possible, though it requires higher energy. Next, **C2** undergoes reductive elimination via transition state **TS1C** to produce complex **C3**. Finally, **C3** releases the hydroarylated product **C4** by binding a PMe_3_ ligand to regenerate the Ni(O) catalyst, thereby closing the catalytic cycle. In contrast, the oxidative Heck coupling cycle proceeds in the presence of a hydrogen acceptor and involves the following steps. First, 1,4-addition of the Ni–H moiety from [Ni(Ar)(H)] species **C** to the hydrogen acceptor and concomitant release of PMe_3_ forms intermediate **C'1-1**. Subsequent carbometalation across the C=C bond via **TS2B** leads to formation of linear intermediate **C'2**. β-Hydride elimination via **TS2C** results in olefinic complex **C'3**, and subsequent transfer hydrogenation furnishes the product **C'4** and regenerates the Ni^0^ catalyst. DFT calculations indicate that: (1) the energy of direct carbometalation of **C** via **TS2A** in the absence of hydrogen acceptor is much higher than that of hydrometalation step via **TS1A** (37.6 vs. 21.0); and (2) the addition and coordination of hydrogen acceptor significantly lowers the energy of the carbometalation step (37.6 vs. 20.8). Therefore, the presence of a hydrogen acceptor is expected to accelerate the oxidative Heck coupling, which is consistent with the experimental results. Transition states **TS2B** and **TS1A** are close in energy (20.8 vs. 21.7), meaning that hydroarylation and Heck coupling are competitive.

In summary, we describe a transfer-hydrogenation-coupled oxidative Heck reaction catalyzed by a versatile Ni(O)/PR_3_/alcohol catalytic system. With the aid of a cleavable AQ directing group, a broad range of γ,δ- and δ,ε-unsaturated carboxamides, including traditionally challenging non-conjugated internal alkenes, are tolerated. The arylated alkene products are typically obtained with high *E*/*Z* selectivity and as single positional isomers. Mechanistic studies are consistent with a transfer hydrogenation process involving an active [Ni(Ar)(H)] species. The hydrogen acceptor promotes the carbometalation step and formally accepts dihydrogen from the metal. To our knowledge, intermolecular Ni(O)-catalyzed oxidative boron Heck reaction has not been reported before.

## Methods

### General procedure for Heck arylation

In an argon-filled glovebox, an oven-dried 25-mL Schlenk tube equipped with a Teflon-coated magnetic stir bar was charged successively with alkene **1** (0.2 mmol, 1.0 equiv.), arylboronic acid **2** (0.4 mmol, 2.0 equiv.), Ni(cod)_2_ (0.02 mmol, 0.0055 g, 0.1 equiv.), CsOPiv (0.3 mmol, 0.0702 g, 1.5 equiv.), (*E*)-3-(3,4,5-trimethoxyphenyl)acrylaldehyde **A18** (0.2 mmol, 0.0444 g, 1.0 equiv.), anhydrous *t*-AmylOH (1 mL), and P*n*Bu_3_ (0.04 mmol, 0.0081 g, 0.2 equiv.). The tube was sealed with a Teflon screw cap, moved out of the glovebox, and placed on a hotplate pre-heated to 70 °C with vigorous stirring. After 48 h, the reaction mixture was cooled to room temperature and diluted with EtOAc. The organic layer was washed with brine solution and was then dried over anhydrous MgSO_4_. The reaction mixture was filtered through a short pad of Celite, and the solvent was evaporated under vacuum to give the crude product. The resulting residue was purified by silica gel flash column chromatography (hexane/EtOAc = 10/1) to afford the corresponding product. For details of the synthetic procedures and NMR spectra of these compounds in this manuscript see Supplementary information.

## Supplementary information

Supplementary Information

Description of Additional Supplementary Files

Supplementary Data 1

## Data Availability

The authors declare that the data supporting the findings of this study are available within the article and its Supplementary Information Files.
