# Supplementary material for: Nickel-catalyzed intermolecular oxidative Heck arylation driven by transfer hydrogenation
Source: Nat Commun. 2019 Nov 5;10:5025. doi: 10.1038/s41467-019-12949-1 (PMC6831602; doi:10.1038/s41467-019-12949-1)
Supplement: Supplementary file 1 — Supplementary Information [file 41467_2019_12949_MOESM1_ESM.pdf]

Supplementary Information

Nickel-Catalyzed Intermolecular Oxidative Heck Arylation Driven by Transfer Hydrogenation

Lv et al.

Supplementary Methods

General Information: Unless otherwise noted, all manipulations were performed in an argon-filled glovebox or using standard Schlenk techniques. Commercially available reagents were received from commercial suppliers and were used without further purification, and anhydrous solvents (<50 ppm H_2O) stored over molecular sieves under argon atmosphere and were transferred under argon. NMR spectra were recorded on a Bruker AV 400 spectrometer at 400 MHz (^{1}H NMR), 100 MHz (^{13}C NMR), 376 MHz (^{19}F NMR). Chemical shifts (δ) for ^{1}H and ^{13}C NMR spectra are given in ppm relative to TMS. The residual solvent signals were used as references for ^{1}H and ^{13}C NMR spectra, and the chemical shifts were converted to the TMS scale ($CDCl_3$: δ_H = 7.26 ppm, δ_C = 77.16 ppm; $(CD_3)_2SO$: δ_H = 2.50 ppm, δ_C = 39.52 ppm; CD_3OD: δ_H = 3.34 ppm, δ_C = 49.86 ppm). ^{1}H, ^{13}C and ^{19}F multiplicities are reported as follows: singlet (s), doublet (d), triplet (t), quartet (q), doublet of doublets (dd), triplet of quartets (tq), multiplet (m), and broad resonance (br). High resolution mass spectra (HRMS) were recorded on an Agilent 6520 Q-TOF LC/MS with an Electron Spray Ionization (ESI) source. Thin-layer chromatography was performed on pre-coated silica gel 60 F254 plates (Merck). Silica gel 60H (200–300 mesh) manufactured by Qingdao Haiyang Chemical Group Co. (China) was used for general silica gel flash column chromatography.

Supplementary Table 1. Solvent Effects [a]

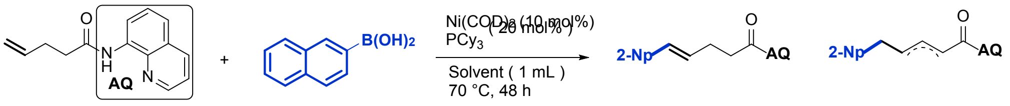

Entry	Solvent	Yield(%)[b]
1	MeOH	21% （**5a**/**6a**= 1.6:1）
2	EtOH	27% （**5a**/**6a** = 1.5:1）
3	*t*-AmylOH	19% （**5a**/**6a**= 6:1）

[a] Reaction conditions: **1a** (0.2 mmol), **2a** (0.4 mmol). [b] Yields were determined by NMR spectroscopy with an internal standard ($C_2H_2Br_4$).

Supplementary Table 2. Ligand Effects[a]

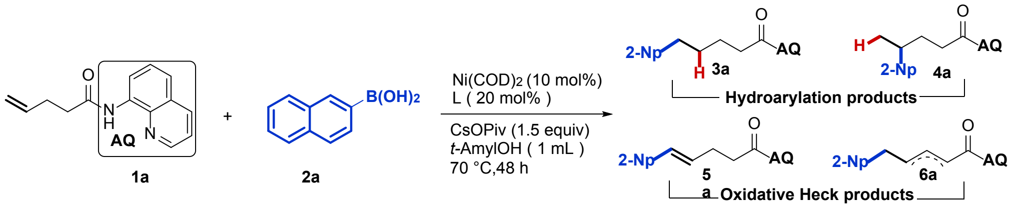

Entry	Ligand	Yield [%][b] (**3a**:**4a**:**5a**:**6a**)
1	PPh_3	47 (0/17/25/5)
2	PCy_3	41 (0/0/35/6)
3	P*n*Bu_3	39 (0/9/30/0)
4	P*t*Bu_3	25 (25/0/0/0)
5	XPhos	35 (35/0/0/0)
6	PMe_3	85 (0/85/0/0)
7	PPh_2Cy	44 (0/17/22/5)
8	Dcype	0
9	$PPhCy_2$	43 (0/10/29/4)
10	$P(OMe)_3$	30 (0/14/16/0)
11	P*n*Pr_3	29 (0/13/16/0)
12	PMe_2Ph	32 (0/25/7/0)
13	P*t*$BuCy_2$	32 (0/15/17/0)
14	Xantphos	38 (0/16/19/3)
15[c]	PCy_3	79 (0/0/79/0)
16[c]	P*n*Bu_3	92 (0/0/92/0)

[a] Reaction conditions: **1a** (0.2 mmol), **2a** (0.4 mmol). [b] Yields were determined by NMR spectroscopy with an internal standard ($C_2H_2Br_4$). [c] **1a** : **2a** = 1.5:1

Supplementary Table 3. H_2 Scavenger Optimization[a,b]

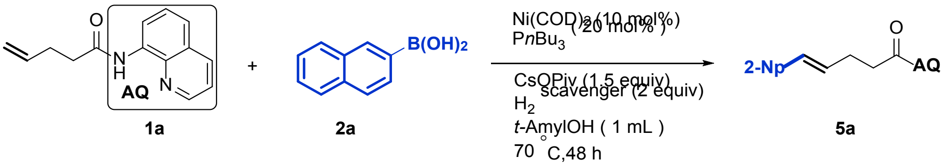

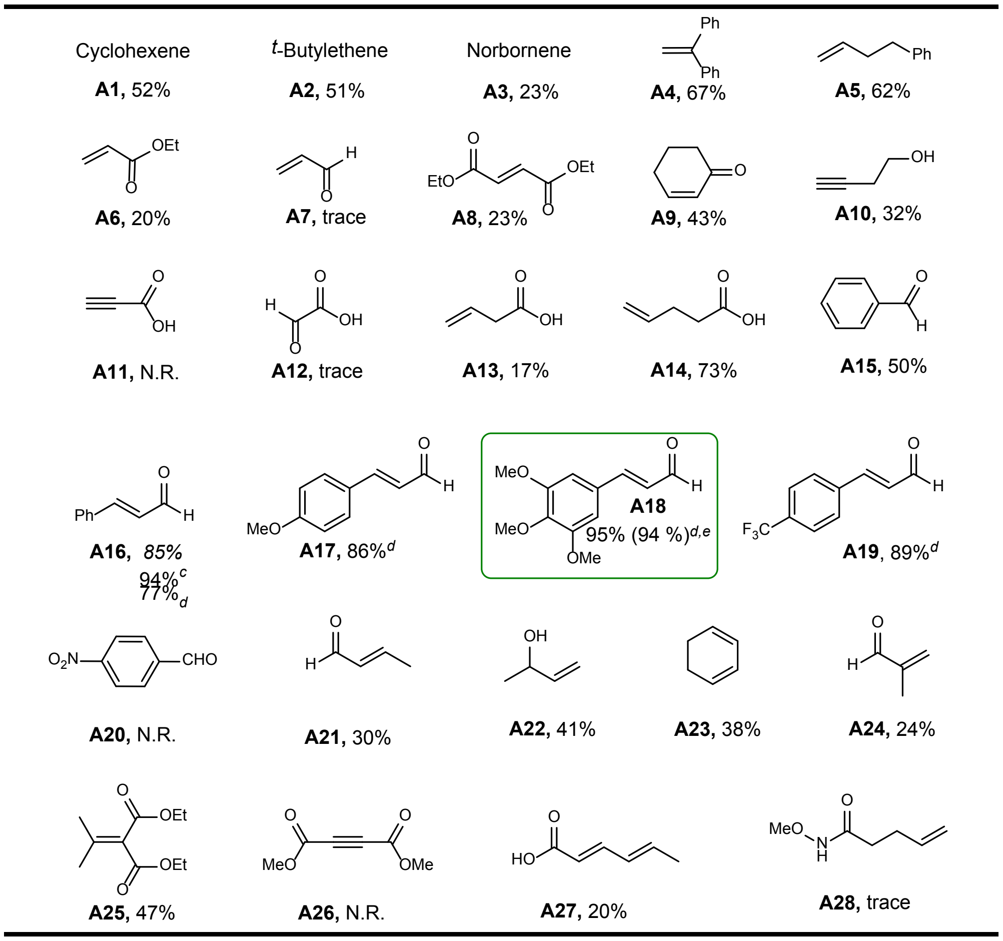

[a] Reaction conditions: **1a** (0.2 mmol), **2a** (0.4 mmol). N.R. = no reaction. [b] Yields were determined by NMR spectroscopy with an internal standard ($C_2H_2Br_4$). [c] 3.0 equiv H_2 scavenger. [d] 1.0 equiv H_2 scavenger. [e] The isolated yield is given in parenthesis.

Supplementary Table 4. The Amount of Catalyst Optimization[a]

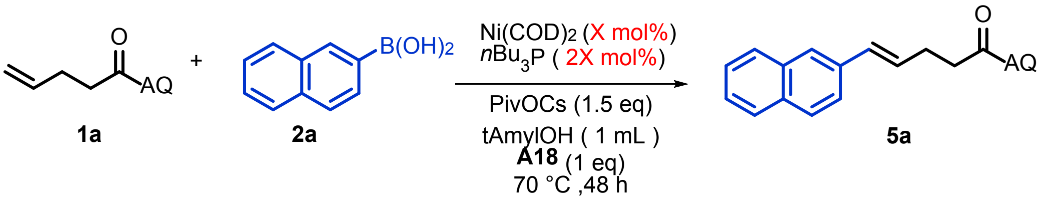

Entry	Ni(COD)$_2$	Yield(%)[b]
1	2.5 mol%	44
2	5 mol%	83
3	10 mol%	92

[a] Reaction conditions: **1a** (0.2 mmol), **2a** (0.4 mmol). [b] Yields were determined by NMR spectroscopy with an internal standard ($C_2H_2Br_4$).

Supplementary Table 5. The Screening of Ni(II) Salts as the Catalyst[a]

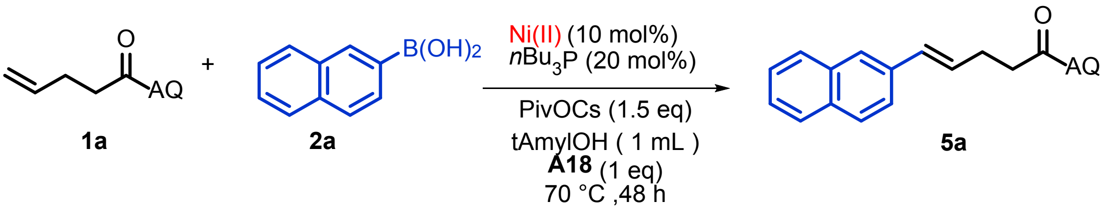

Entry	Ni(II)	Yield(%)[b]
1	$NiCl_2$	72
2	$NiBr_2$	46
3	$Ni(OTf)_2$	17
4	$Ni(acac)_2$	68

[a] Reaction conditions: **1a** (0.2 mmol), **2a** (0.4 mmol). [b] Yields were determined by NMR spectroscopy with an internal standard ($C_2H_2Br_4$).

Supplementary Table 6. The Screening of the Directing Group[a]

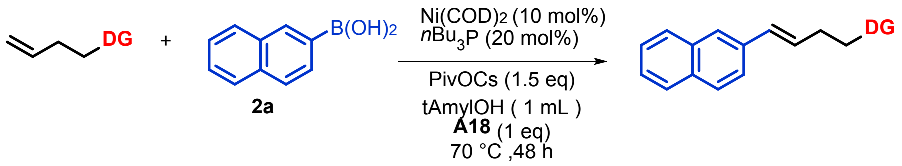

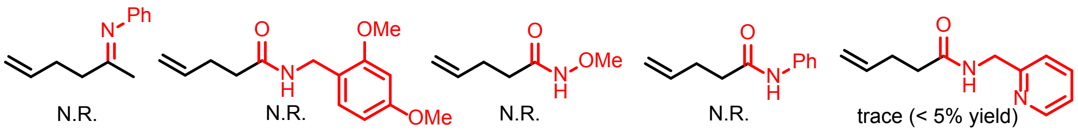

[a] Reaction conditions: alkene with directing group (0.2 mmol), **2a** (0.4 mmol). N.R. = no reaction.

General procedure for synthesis of alkene substrates

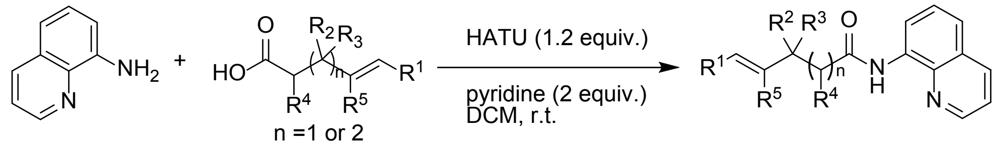

Following literature procedures,[1-5] the corresponding vinylacetic acid (30 mmol, 1.2 equiv.) was charged into a 250-mL RB flask containing dichloromethane (60 mL). 8-Aminoquinoline (25 mmol, 3.6 g, 1 equiv.), pyridine (50 mmol, 3.96 g, 2 equiv.), and HATU (30 mmol, 11.4 g, 1.2 equiv.) were added sequentially, and the reaction was stirred at ambient temperature for 16 h. The residue was dissolved in EtOAc (100 mL), washed with sat. $NaHCO_3$ (2 × 70 mL) and brine (1 × 70 mL), and purified by silica gel flash column chromatography to yield the corresponding product.

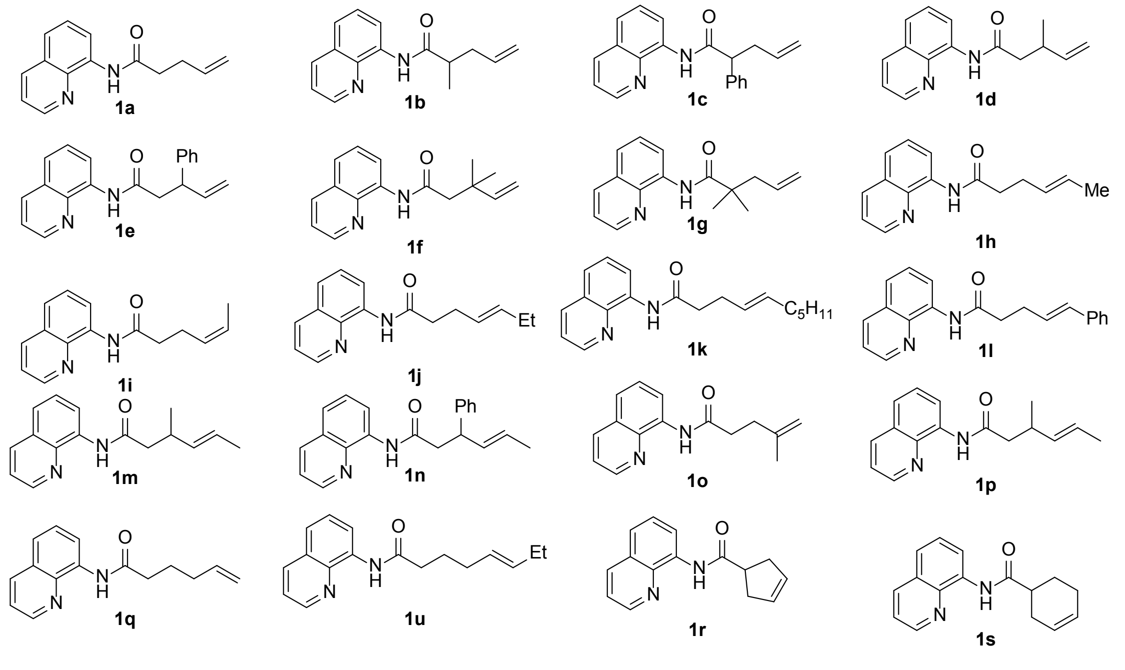

Supplementary Figure 1. List of known alkene substrates

Synthesis of alkene substrate 1t

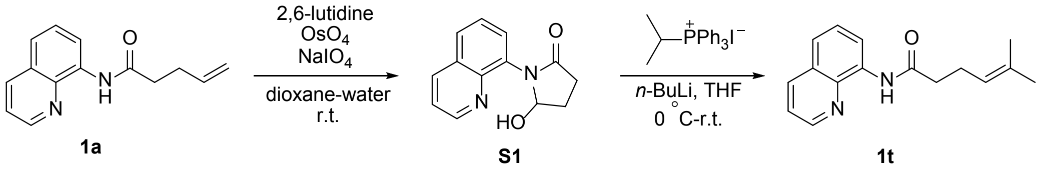

To a solution of **1a** (4.2 mmol, 0.951 g) in a solution of dioxane/water (3:1, 48 mL) was added 2,6-lutidine (12.6 mmol, 1.35 g), OsO_4 (2% in *tert*-butanol, 0.084 mmol), and $NaIO_4$ (16.8 mmol, 3.6 g,). The reaction was stirred at rt for 24 h, diluted with

water (40 mL), and extracted with CH_2Cl_2 (40 mL × 3). The combined organic layers were washed with brine, dried over $MgSO_4$, concentrated, and purified by column chromatography (ethyl acetate as eluent) to obtain the intermediate **S1** (0.786 g, 82 %) as a white solid. **^{1}H NMR (400 MHz, $CDCl_3$):** δ 8.87–8.73 (m, 1H), 8.69 (br, 1H), 8.33–8.15 (m, 1H), 7.86–7.70 (m, 2H), 7.63–7.55 (m, 1H), 7.50–7.41 (m, 1H), 5.81–5.20 (m, 1H), 3.10–2.97 (m, 1H), 2.58–2.49 (m, 1H), 2.37–2.26 (m, 2H). **^{13}C NMR (100 MHz, $CDCl_3$):** δ 176.70, 149.06, 142.64, 137.99, 135.48, 129.71, 128.95, 127.03, 126.80, 121.45, 86.07, 29.72, 29.02. **HRMS (ESI)** m/z Calcd. for $C_{13}H_{12}N_2O_2$ $[M+Na]^+$ 251.0796 , Found 251.0806.

To a stirred solution of isopropyltriphenylphosphonium iodide (3.4 g, 7.9 mmol) in THF (30 mL) at –78 °C was dropwise *n*-BuLi (3.2 mL, 2.5 M solution in hexanes) via syringe. Then the mixture was warmed to 0 °C and stirred for 2 h, and a solution of compound **S1** (2.63 mmol, 0.6 g) in THF (10 mL) was added dropwise via syringe, whereupon the mixture was allowed to warm to r.t. and stir overnight. The mixture was then quenched with H_2O (20 mL) and extracted with EtOAc (3 × 40 mL). The combined organic layers were washed with brine and dried over $MgSO_4$. After removal of the solvent under reduced pressure, the residue was purified by silica gel flash column chromatography to obtain **1t** (0.454 g, 68 %) as a colorless oil. **^{1}H NMR (400 MHz, $CDCl_3$):** δ 9.81 (s, 1H), 9.08–8.59 (m, 2H), 8.13 (dd, J = 8.3, 1.6 Hz, 1H), 7.88–7.25 (m, 3H), 5.23 (t, J = 7.0 Hz, 1H), 2.61–2.57 (m, 2H), 2.53–2.48 (m, 2H), 1.70 (s, 3H), 1.66 (s, 3H). **^{13}C NMR (100 MHz, $CDCl_3$):** δ 171.62, 148.18, 138.44, 136.43, 134.70, 133.55, 128.03, 127.54, 122.66, 121.65, 121.42, 116.52, 38.39, 25.86, 24.33, 17.91.

General procedure A for hydroarylation of γ,δ-unsaturated amides

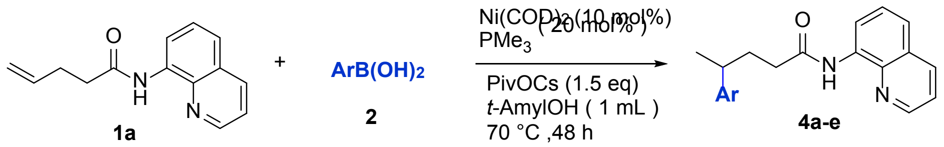

In an argon-filled glovebox, an oven-dried 25-mL Schlenk tube equipped with a Teflon-coated magnetic stir bar was charged successively with alkene **1** (0.2 mmol, 1.0 equiv.), phenylboronic acid **2** (0.4 mmol, 2.0 equiv.), $Ni(cod)_2$ (0.02 mmol, 0.0055

g, 0.1 equiv.), CsOPiv (0.3 mmol, 0.0702 g, 1.5 equiv.), anhydrous *t*-AmylOH (1 mL), and PMe_3 (0.04 mmol, 10% in toluene, 0.2 equiv.). The tube was sealed with a Teflon screw cap, moved out of the glovebox, and placed on a hotplate pre-heated to 70 °C with vigorous stirring. After 48 h, the reaction mixture was cooled to rt and diluted with EtOAc. The organic layer was washed with brine solution and was then dried over anhydrous $MgSO_4$. The reaction mixture was filtered through a short pad of Celite, and the solvent was evaporated under vacuum to give the crude product. The resulting residue was purified by silica gel flash column chromatography (hexane/EtOAc = 10/1) to afford the corresponding product **4a–e**.

General procedure B for hydroarylation of δ,δ-disubstituted alkenes

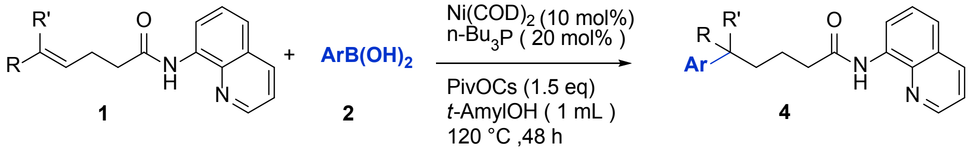

In an argon-filled glovebox, an oven-dried 25-mL Schlenk tube equipped with a Teflon-coated magnetic stir bar was charged successively with alkene **1** (0.2 mmol, 1.0 equiv.), phenylboronic acid **2** (0.4 mmol, 2.0 equiv.), $Ni(cod)_2$ (0.02 mmol, 0.0055 g, 0.1 equiv.), CsOPiv (0.3 mmol, 0.0702 g, 1.5 equiv.), anhydrous *t*-AmylOH (1 mL), and *n*-Bu_3P (0.04 mmol, 0.0081 g, 0.2 equiv.). The tube was sealed with a Teflon screw cap, moved out of the glovebox, and placed on a hotplate pre-heated to 120 °C with vigorous stirring. After 48 h, the reaction mixture was cooled to rt and diluted with EtOAc. The organic layer was washed with brine solution and was then dried over anhydrous $MgSO_4$. The reaction mixture was filtered through a short pad of Celite, and the solvent was evaporated under vacuum to give the crude product. The resulting residue was purified by silica gel flash column chromatography (hexane/EtOAc = 10/1) to afford the corresponding product **4**.

General procedure C for oxidative Heck arylation of γ,δ-unsaturated amides

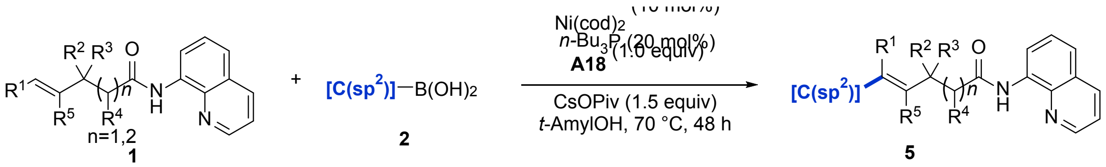

In an argon-filled glovebox, an oven-dried 25-mL Schlenk tube equipped with a Teflon-coated magnetic stir bar was charged successively with alkene **1** (0.2 mmol, 1.0 equiv.), arylboronic acid **2** (0.4 mmol, 2.0 equiv.), $Ni(cod)_2$ (0.02 mmol, 0.0055 g, 0.1 equiv.), CsOPiv (0.3 mmol, 0.0702 g, 1.5 equiv.), (*E*)-3-(3,4,5-trimethoxyphenyl)acrylaldehyde **A18** (0.2 mmol, 0.0444 g, 1.0 equiv.), anhydrous *t*-AmylOH (1 mL), and *n*-Bu_3P (0.04 mmol, 0.0081 g, 0.2 equiv.). The tube was sealed with a Teflon screw cap, moved out of the glovebox, and placed on a hotplate pre-heated to 70 °C with vigorous stirring. After 48 h, the reaction mixture was cooled to rt and diluted with EtOAc. The organic layer was washed with brine solution and was then dried over anhydrous $MgSO_4$. The reaction mixture was filtered through a short pad of Celite, and the solvent was evaporated under vacuum to give the crude product. The resulting residue was purified by silica gel flash column chromatography (hexane/EtOAc = 10/1) to afford the corresponding product **5**.

General procedure D for oxidative Heck arylation of δ,ε-unsaturated amides

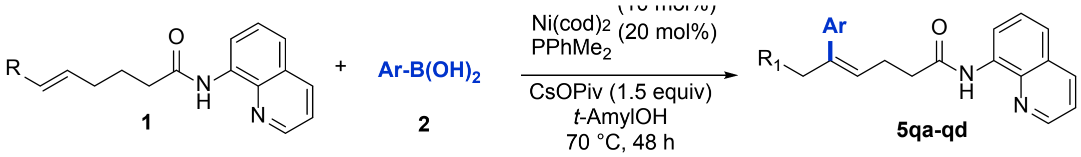

In an argon-filled glove-box, an oven-dried 25-mL Schlenk tube equipped with a Teflon-coated magnetic stir bar was charged successively alkene **1** (0.3 mmol, 1.5 equiv.), arylboronic acid **2** (0.2 mmol, 1.0 equiv.), $Ni(cod)_2$ (0.02 mmol, 0.0055 g, 0.1 equiv.), CsOPiv (0.3 mmol, 0.0702 g, 1.5 equiv.), anhydrous *t*-AmylOH (1 mL), and $PPhMe_2$ (0.04 mmol, 0.0055 g, 0.2 equiv.). The tube was sealed with a Teflon screw cap, moved out of the glovebox, and placed on a hotplate pre-heated to 70 °C with

vigorous stirring. After 48 h, the reaction mixture was cooled to rt and diluted with EtOAc. The organic layer was washed with brine solution and was then dried over anhydrous $MgSO_4$. The reaction mixture was filtered through a short pad of Celite, and the solvent was evaporated under vacuum to give the crude product. The resulting residue was purified by flash chromatography on silica gel (hexane/EtOAc = 10/1) to afford the corresponding product (**5qa–qd**).

Gram-scale experiment

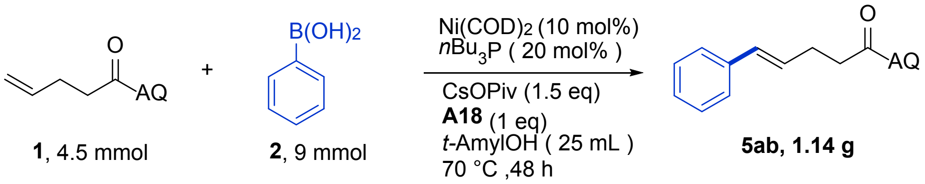

In an argon-filled glovebox, an oven-dried 100-mL Schlenk tube equipped with a Teflon-coated magnetic stir bar was successively charged with alkene **1** (4.5 mmol, 1.02 g, 1.0 equiv.), phenylboronic acid **2** (9 mmol, 1.1 g, 2.0 equiv.), $Ni(cod)_2$ (0.45 mmol, 0.124 g, 0.1 equiv.), CsOPiv (6.75 mmol, 1.58 g, 1.5 equiv.), (*E*)-3-(3,4,5-trimethoxyphenyl)acrylaldehyde **A18** (4.5 mmol, 1.0 g, 1.0 equiv.), anhydrous *t*-AmylOH (25 mL), and *n*-Bu_3P (9 mmol, 0.183 g, 0.2 equiv.). The tube was sealed with a Teflon screw cap, moved out of the glovebox, and placed on a hotplate pre-heated to 70 °C with vigorous stirring. After 48 h, the reaction mixture was cooled to rt and diluted with EtOAc. The organic layer was washed with brine solution and was then dried over anhydrous $MgSO_4$. The reaction mixture was filtered through a short pad of Celite, and the solvent was evaporated under vacuum to give the crude product. The resulting residue was purified by silica gel flash column chromatography (hexane/ethyl acetate = 10:1) to give **5ab** (1.14 g) as a light yellow solid in 84% yield.

4-(Naphthalen-2-yl)-*N*-(quinolin-8-yl)pentanamide (4a)

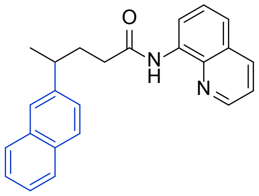

Following general procedure A, the reaction was carried out with alkene **1a** (0.2 mmol, 1.0 equiv.), naphthalen-2-ylboronic

acid **2a** (0.4 mmol, 2.0 equiv.), Ni(cod)$_2$ (0.02 mmol, 0.0055 g, 0.1 equiv.), CsOPiv (0.3 mmol, 0.0702 g, 1.5 equiv.), and PMe$_3$ (0.04 mmol, 10% in toluene, 0.2 equiv.) in anhydrous *t*-AmylOH (1 mL) at 70 °C for 48 h. 4-(Naphthalen-2-yl)-*N*-(quinolin-8-yl)pentanamide (**4a**) was isolated by column chromatography (PE/EtOAc = 10/1) as a white solid in 83 % yield. **M.P.**: 78–79 ºC. **^{1}H NMR (400 MHz, CDCl$_3$):** δ 9.70 (s, 1H), 8.81 (d, *J* = 7.4 Hz, 1H), 8.73 (d, *J* = 3.0 Hz, 1H), 8.13 (d, *J* = 8.0 Hz, 1H), 7.84–7.77 (m, 3H), 7.78 (d, *J* = 7.1 Hz, 1H), 7.69 (s, 1H), 7.59–7.36 (m, 6H), 3.07–2.99 (m, 1H), 2.47 (t, *J* = 7.5 Hz, 2H), 2.39–2.09 (m, 2H), 1.43 (d, *J* = 6.9 Hz, 3H). **^{13}C NMR (100 MHz, CDCl$_3$):** δ 171.76, 148.14, 143.86, 138.33, 136.38, 134.54, 133.69, 132.42, 128.36, 127.97, 127.69, 127.47, 125.98, 125.69, 125.60, 125.35, 121.62, 121.47, 116.49, 39.73, 36.29, 33.59, 22.68. **HRMS (ESI)** m/z Calcd. for $C_{24}H_{22}N_2O$ $[M+H]^+$ 355.1810, Found 355.1806.

4-(Benzo[*d*][1,3]dioxol-5-yl)-*N*-(quinolin-8-yl)pentanamide (**4b**)

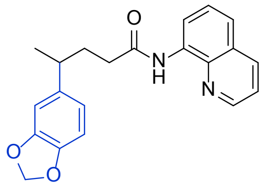

Following general procedure A, the reaction was carried out with alkene **1a** (0.2 mmol, 1.0 equiv.), benzo[*d*][1,3]dioxol-5-ylboronic acid **2q** (0.4 mmol, 2.0 equiv.), Ni(cod)$_2$ (0.02 mmol, 0.0055 g, 0.1 equiv.), CsOPiv (0.3 mmol, 0.0702 g, 1.5 equiv.), and PMe$_3$ (0.04 mmol, 10% in toluene, 0.2 equiv.) in anhydrous *t*-AmylOH (1 mL) at 70 °C for 48 h. 4-(Benzo[*d*][1,3]dioxol-5-yl)-*N*-(quinolin-8-yl)pentanamide (**4b**) was isolated by column chromatography (PE/EtOAc = 10/1) as a colorless oil in 85 % yield. **^{1}H NMR (400 MHz, CDCl$_3$):** δ 9.69 (s, 1H), 8.86–8.66 (m, 2H), 8.13 (d, *J* = 8.2 Hz, 1H), 7.58–7.36 (m, 3H), 6.75–6.67 (m, 3H), 5.90 (s, 2H), 2.83–2.68 (m, 1H), 2.42 (t, *J* = 7.6 Hz, 2H), 2.13 (td, *J* = 13.5, 7.7 Hz, 1H), 2.06–1.91 (m, 1H), 1.28 (d, *J* = 6.9 Hz, 3H). **^{13}C NMR (100 MHz, CDCl$_3$):** δ 171.76, 148.16, 147.83, 145.87, 140.39, 138.33, 136.42, 134.52, 127.98, 127.47, 121.64, 121.46, 120.22, 116.50, 108.27, 107.35, 100.87, 39.40, 36.24, 33.92, 22.89. **HRMS (ESI)** m/z Calcd. for $C_{21}H_{20}N_2O_3$ $[M+H]^+$ 349.1552, Found 349.1550.

4-(Benzofuran-5-yl)-*N*-(quinolin-8-yl)pentanamide (**4c**)

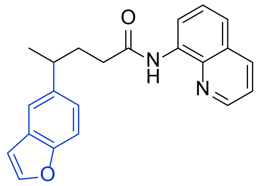

Following general procedure A, the reaction was carried out with alkene **1a** (0.2 mmol, 1.0 equiv.), benzofuran-5-ylboronic acid **2u** (0.4 mmol, 2.0 equiv.), Ni(cod)$_2$ (0.02 mmol, 0.0055 g, 0.1 equiv.), CsOPiv (0.3 mmol, 0.0702 g, 1.5 equiv.), and PMe$_3$ (0.04 mmol, 10% in toluene, 0.2 equiv.) in anhydrous *t*-AmylOH (1 mL) at 70 °C for 48 h. 4-(Benzofuran-5-yl)-*N*-(quinolin-8-yl) pentanamide (**4c**) was isolated by column chromatography (PE/EtOAc = 10/1) as a colorless oil in 81 % yield. **^{1}H NMR (400 MHz, CDCl$_3$):** δ 9.46 (s, 1H), 8.61–8.47 (m, 2H), 7.90 (dd, J = 8.3, 1.5 Hz, 1H), 7.37 (d, J = 2.1 Hz, 1H), 7.33–7.14 (m, 5H), 6.97 (dd, J = 8.5, 1.6 Hz, 1H), 6.48 (d, J = 1.3 Hz, 1H), 2.82–2.63 (m, 1H), 2.22 (t, J = 7.6 Hz, 2H), 2.07–1.95 (m, 1H), 1.93–1.86 (m, 1H), 1.16 (d, J = 6.9 Hz, 3H). **^{13}C NMR (100 MHz, CDCl$_3$):** δ 171.79, 153.84, 148.11, 145.21, 140.96, 138.35, 136.39, 134.57, 127.98, 127.66, 127.47, 123.61, 121.61, 121.43, 119.38, 116.47, 111.35, 106.58, 39.56, 36.34, 34.19, 23.25. **HRMS (ESI)** m/z Calcd. for $C_{22}H_{20}N_2O_2$ $[M+H]^+$ 345.1603, Found 345.1600.

4-(4-Acetylphenyl)-*N*-(quinolin-8-yl)pentanamide (**4d**)

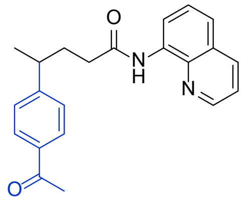

Following general procedure A, the reaction was carried out with alkene **1a** (0.2 mmol, 1.0 equiv.), 4-acetylphenylboronic acid **2n** (0.4 mmol, 2.0 equiv.), Ni(cod)$_2$ (0.02 mmol, 0.0055 g, 0.1 equiv.), CsOPiv (0.3 mmol, 0.0702 g, 1.5 equiv.), and PMe$_3$ (0.04 mmol, 10% in toluene, 0.2 equiv.) in anhydrous *t*-AmylOH (1 mL) at 70 °C for 48 h. 4-(4-Acetylphenyl)-*N*-(quinolin-8-yl)pentanamide (**4d**) was isolated by column chromatography (PE/EtOAc = 10/1) as a white solid in 86 % yield. **M.P.**: 79–80 ºC. **^{1}H NMR (400 MHz, CDCl$_3$):** δ 9.67 (s, 1H), 8.73 (d, J = 6.6 Hz, 2H), 8.12 (d, J = 8.2 Hz, 1H), 7.88 (d, J = 8.1 Hz, 2H), 7.54–7.44 (m, 2H), 7.41 (dd, J = 8.2, 4.2 Hz, 1H), 7.31 (d, J = 8.1 Hz, 2H), 2.90 (dq, J = 13.3, 6.6 Hz, 1H), 2.54 (s, 3H), 2.41 (t, J = 7.5 Hz, 2H), 2.26–1.98 (m, 2H), 1.32 (d, J = 6.9 Hz, 3H). **^{13}C NMR (100 MHz, CDCl$_3$):** δ 197.82, 171.29, 152.23, 148.15, 138.27, 136.40, 135.47, 134.42, 128.79, 127.95, 127.43, 127.41, 121.66, 121.53, 116.45, 39.59, 36.04, 33.40, 26.58, 22.24. **HRMS (ESI)** m/z Calcd. for $C_{22}H_{22}N_2O_2$ $[M+H]^+$ 347.1760, Found 347.1755.

N-(Quinolin-8-yl)-4-o-tolylpentanamide (4e)

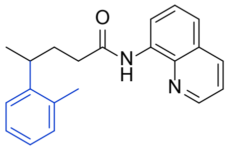

Following general procedure A, the reaction was carried out with alkene **1a** (0.2 mmol, 1.0 equiv.), 4-acetylphenylboronic acid **2c** (0.4 mmol, 2.0 equiv.), Ni(cod)$_2$ (0.02 mmol, 0.0055 g, 0.1 equiv.), CsOPiv (0.3 mmol, 0.0702 g, 1.5 equiv.), and PMe$_3$ (0.04 mmol, 10 % in toluene, 0.2 equiv.) in anhydrous *t*-AmylOH (1 mL) at 70 °C for 48 h. *N*-(Quinolin-8-yl)-4-*o*-tolylpentanamide (**4e**) was isolated by column chromatography (PE/EtOAc = 10/1) as a colorless oil in 77 % yield. **^{1}H NMR (400 MHz, CDCl$_3$):** δ 9.61 (s, 1H), 8.76–8.61 (m, 2H), 8.07 (d, J = 8.3 Hz, 1H), 7.48–7.38 (m, 2H), 7.35 (dd, J = 8.2, 4.2 Hz, 1H), 7.19 (d, J = 8.0 Hz, 1H), 7.13 (t, J = 7.1 Hz, 2H), 7.08–6.97 (m, 2H), 3.16–2.98 (m, 1H), 2.39 (t, J = 7.7 Hz, 2H), 2.23 (s, 3H), 2.13–1.99 (m, 2H), 1.20 (d, J = 6.9 Hz, 3H). **^{13}C NMR (100 MHz, CDCl$_3$):** δ 171.78, 148.09, 144.62, 138.30, 136.57, 135.76, 134.59, 130.51, 128.04, 127.59, 126.50, 125.87, 125.36, 121.66, 121.47, 116.63, 36.21, 34.10, 32.98, 22.12, 19.74. **HRMS (ESI)** m/z Calcd. for $C_{21}H_{22}N_2O$ $[M+H]^+$ 319.1810, Found 319.1806.

3-(Naphthalen-2-yl)-N-(quinolin-8-yl)cyclopentanecarboxamide (4f)

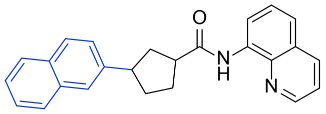

Following general procedure B, the reaction was carried out with alkene **1r** (0.2 mmol, 1.0 equiv.), naphthalen-2-ylboronic acid **2a** (0.4 mmol, 2.0 equiv.), Ni(cod)$_2$ (0.02 mmol, 0.0055 g, 0.1 equiv.), CsOPiv (0.3 mmol, 0.0702 g, 1.5 equiv.), and PMe$_3$ (0.04 mmol, 10% in toluene, 0.2 equiv.) in anhydrous *t*-AmylOH (1 mL) at 120 °C for 48 h. 3-(Naphthalen-2-yl)-*N*-(quinolin-8-yl) cyclopentanecarboxamide (**4f**) was isolated by column chromatography (PE/EtOAc = 10/1) as a light yellow oil in 41 % yield. **^{1}H NMR (400 MHz, CDCl$_3$):** δ 9.97 (s, 1H), 8.87–8.82 (m, 2H), 8.18 (d, J = 8.0 Hz, 1H), 7.92–7.70 (m, 4H), 7.66–7.35 (m, 6H), 3.42–3.29 (m, 1H), 3.29–3.19 (m, 1H), 2.65–2.52 (m, 1H), 2.45–2.15 (m, 4H), 2.11–1.97 (m, 1H). **^{13}C NMR (100 MHz, CDCl$_3$):** δ 174.72, 148.11, 142.18, 138.36, 136.77, 134.73, 133.71, 132.40, 128.16, 128.12, 127.70, 126.16, 126.04, 125.35, 125.33, 121.68, 121.53, 116.87, 100.10, 47.37, 46.75, 39.06, 34.28, 29.98. **HRMS (ESI)** m/z Calcd. for $C_{25}H_{22}N_2O$

[M+H]$^+$ 367.1810, Found 367.1806.

4-(Naphthalen-2-yl)-*N*-(quinolin-8-yl)cyclohexanecarboxamide (4g)

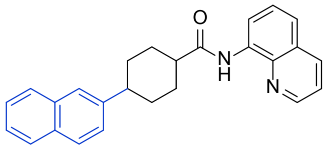

Following general procedure B, the reaction was carried out with alkene **1s** (0.2 mmol, 1.0 equiv.), naphthalen-2-ylboronic acid **2a** (0.4 mmol, 2.0 equiv.), Ni(cod)$_2$ (0.02 mmol, 0.0055 g, 0.1 equiv.), CsOPiv (0.3 mmol, 0.0702 g, 1.5 equiv.), and *n*-Bu$_3$P (0.04 mmol, 0.0081 g, 0.2 equiv.) in anhydrous *t*-AmylOH (1 mL) at 120 °C for 48 h. 4-(Naphthalen-2-yl)-*N*-(quinolin-8-yl)cyclohexanecarboxamide (**4 g**) was isolated by column chromatography (PE/EtOAc = 10/1) as a white solid in 52 % yield. **M.P.**: 94-95 ºC. **^{1}H NMR (400 MHz, CDCl$_3$):** δ 10.15 (s, 1H), 8.88 (d, J = 7.5 Hz, 1H), 8.81 (dd, J = 4.1, 1.4 Hz, 1H), 8.16 (dd, J = 8.2, 1.3 Hz, 1H), 7.78 (t, J = 8.6 Hz, 3H), 7.70 (s, 1H), 7.58 (t, J = 7.9 Hz, 1H), 7.51 (d, J = 8.1 Hz, 1H), 7.48–7.36 (m, 4H), 3.02–2.94 (m, 1H), 2.88–2.81 (m, 1H), 2.49–2.45 (m, 2H), 2.20–2.06 (m, 2H), 2.01–1.88 (m, 4H). **^{13}C NMR (100 MHz, CDCl$_3$):** δ 173.91, 148.27, 144.50, 138.67, 136.51, 134.83, 133.75, 132.24, 128.12, 127.88, 127.76, 127.61, 126.37, 125.88, 125.21, 124.99, 121.67, 121.36, 116.47, 43.60, 41.45, 30.38, 28.30. **HRMS (ESI)** m/z Calcd. for $C_{26}H_{24}N_2O$ [M+H]$^+$ 381.1967, Found 381.1963.

5-Methyl-5-(naphthalen-2-yl)-*N*-(quinolin-8-yl)hexanamide (4h)

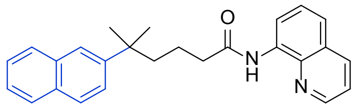

Following general procedure B, the reaction was carried out with alkene **1t** (0.2 mmol, 1.0 equiv.), naphthalen-2-ylboronic acid **2a** (0.4 mmol, 2.0 equiv.), Ni(cod)$_2$ (0.02 mmol, 0.0055 g, 0.1 equiv.), CsOPiv (0.3 mmol, 0.0702 g, 1.5 equiv.), and *n*-Bu$_3$P (0.04 mmol, 0.0081 g, 0.2 equiv.) in anhydrous *t*-AmylOH (1 mL) at 120 °C for 48 h. 5-Methyl-5-(naphthalen-2-yl)-*N*-(quinolin-8-yl)hexanamide (**4h**) was isolated by column chromatography (PE/EtOAc = 10/1) as a light yellow oil in 31 % yield. **^{1}H NMR (400 MHz, CDCl$_3$):** δ 9.71 (s, 1H), 8.75 (d, J = 6.5 Hz, 1H), 8.71 (dd, J = 4.1, 1.4 Hz, 1H), 8.14 (d, J = 9.4 Hz, 1H), 7.81–7.71 (m, 4H), 7.56–7.46 (m, 3H), 7.45–7.37 (m, 3H), 2.47 (t, J = 7.4 Hz, 2H), 1.90–1.80 (m, 2H), 1.68–1.57 (m, 2H), 1.45 (s, 6H). **^{13}C NMR (100 MHz, CDCl$_3$):** δ 171.73, 148.12, 146.66, 136.53, 134.62,

133.51, 131.83, 128.06, 127.82, 127.60, 127.44, 125.87, 125.49, 125.36, 124.93, 124.12, 121.66, 121.44, 116.61, 43.85, 38.73, 38.04, 29.84, 28.99, 21.33. **HRMS (ESI)** m/z Calcd. for $C_{26}H_{26}N_2O$ $[M+H]^+$ 383.2123, Found 383.2120.

5-Methyl-5-phenyl-*N*-(quinolin-8-yl)hexanamide (4i)

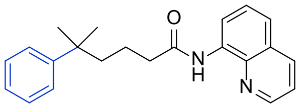

Following general procedure B, the reaction was carried out with alkene **1t** (0.2 mmol, 1.0 equiv.), phenylboronic acid **2** (0.4 mmol, 2.0 equiv.), Ni(cod)$_2$ (0.02 mmol, 0.0055 g, 0.1 equiv.), CsOPiv (0.3 mmol, 0.0702 g, 1.5 equiv.), and *n*-Bu$_3$P (0.04 mmol, 0.0081 g, 0.2 equiv.) in anhydrous *t*-AmylOH (1 mL) at 120 °C for 48 h. 5-Methyl-5-phenyl-*N*-(quinolin-8-yl)hexanamide (**4i**) was isolated by column chromatography (PE/EtOAc = 10/1) as a light yellow oil in 44 % yield. **^{1}H NMR (400 MHz, CDCl$_3$):** δ 9.65 (s, 1H), 8.69 (t, *J* = 5.3 Hz, 2H), 8.06 (d, *J* = 8.2 Hz, 1H), 7.50–7.31 (m, 2H), 7.27 (d, *J* = 7.6 Hz, 2H), 7.22–7.17 (m, 3H), 7.06 (t, *J* = 7.2 Hz, 1H), 2.37 (t, *J* = 7.3 Hz, 2H), 1.76–1.61 (m, 2H), 1.61–1.47 (m, 2H), 1.21 (s, 6H). **^{13}C NMR (100 MHz, CDCl$_3$):** δ 171.74, 149.26, 148.14, 138.42, 136.51, 134.65, 128.23, 128.06, 127.58, 125.92, 125.60, 121.66, 121.43, 116.57, 44.16, 38.75, 37.81, 28.98, 21.23. **HRMS (ESI)** m/z Calcd. for $C_{22}H_{25}N_2O$ $[M+H]^+$ 333.1967, Found 333.1964.

4-(3-Methoxy-4-methylphenyl)-*N*-(quinolin-8-yl)pentanamide (4j)

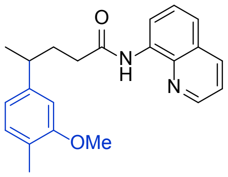

Following general procedure A, the reaction was carried out with alkene **1a** (0.2 mmol, 1.0 equiv.), 3-methoxy-4-methylphenylboronic acid **2y** (0.4 mmol, 2.0 equiv.), Ni(cod)$_2$ (0.02 mmol, 0.0055 g, 0.1 equiv.), CsOPiv (0.3 mmol, 0.0702 g, 1.5 equiv.), and PMe$_3$ (0.04 mmol, 0.2 equiv.) in anhydrous *t*-AmylOH (1 mL) at 70 °C for 48 h. 4-(3-Methoxy-4-methylphenyl)-*N*-(quinolin-8-yl) pentanamide **(4j)** was isolated by column chromatography (PE/EtOAc = 10/1) as a colorless oil in 76 % yield. **^{1}H NMR (400 MHz, CDCl$_3$):** δ 9.69 (s, 1H), 8.78 (t, *J* = 5.4 Hz, 2H), 8.14 (d, *J* = 9.2 Hz, 1H), 7.51 (dt, *J* = 15.3, 7.7 Hz, 2H), 7.44 (dd, *J* = 8.2, 4.2 Hz, 1H), 7.07 (d, *J* = 7.5 Hz, 1H), 6.76 (d, *J* = 7.5 Hz, 1H), 6.70 (s, 1H), 3.79 (s, 3H),

2.86–2.72 (m, 1H), 2.46 (t, *J* = 7.6 Hz, 2H), 2.26–2.12 (m, 4H), 2.09 - 2.01 (m, 1H), 1.33 (d, *J* = 6.9 Hz, 3H). **^{13}C NMR (100 MHz, $CDCl_3$):** δ 171.86, 157.88, 148.13, 145.45, 138.34, 136.46, 134.61, 130.69, 128.01, 127.52, 124.41, 121.66, 121.44, 118.84, 116.49, 108.98, 55.29, 39.72, 36.36, 33.76, 22.90, 15.97. **HRMS (ESI)** m/z Calcd. for $C_{22}H_{24}N_2O_2$ $[M+H]^+$ 349.1916, Found 349.1913.

(*E*)-5-(Naphthalen-2-yl)-*N*-(quinolin-8-yl)pent-4-enamide (5a)

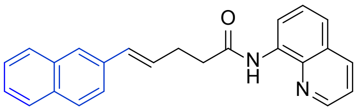

Following general procedure C, the reaction was carried out with alkene **1a** (0.2 mmol, 1.0 equiv.), naphthalen-2-ylboronic acid **2a** (0.4 mmol, 2.0 equiv.), Ni(cod)$_2$ (0.02 mmol, 0.0055 g, 0.1 equiv.), CsOPiv (0.3 mmol, 0.0702 g, 1.5 equiv.), (*E*)-3-(3,4,5-trimethoxyphenyl)acrylaldehyde **A18** (0.2 mmol, 0.0444 g, 1.0 equiv.), and *n*-Bu$_3$P (0.04 mmol, 0.0081 g, 0.2 equiv.) in anhydrous *t*-AmylOH (1 mL) at 70 °C for 48 h. (*E*)-5-(Naphthalen-2-yl)-*N*-(quinolin-8-yl)pent-4-enamide (**5a**) was isolated by column chromatography (PE/EtOAc = 10/1) as a white solid in 94 % yield. **M.P.**: 130–131 °C. **^{1}H NMR (400 MHz, $CDCl_3$):** δ 9.90 (s, 1H), 8.88–8.79 (m, 1H), 8.74 (dd, *J* = 4.2, 1.6 Hz, 1H), 8.14 (dd, *J* = 8.3, 1.6 Hz, 1H), 7.82–7.73 (m, 3H), 7.68 (s, 1H), 7.59 (dd, *J* = 8.8, 1.8 Hz, 1H), 7.54 (d, *J* = 7.7 Hz, 1H), 7.50 (dd, *J* = 8.2, 1.4 Hz, 1H), 7.47–7.40 (m, 3H), 6.69 (d, *J* = 15.8 Hz, 1H), 6.57–6.36 (m, 1H), 2.80–2.76 (m, 4H). **^{13}C NMR (100 MHz, $CDCl_3$):** δ 170.99, 148.22, 138.40, 136.48, 134.97, 134.55, 133.72, 132.86, 131.43, 129.21, 128.17, 128.03, 127.99, 127.72, 127.53, 126.25, 125.82, 125.70, 123.69, 121.69, 121.60, 116.63, 37.89, 29.12. **HRMS (ESI)** m/z Calcd. for $C_{24}H_{20}N_2O$ $[M+H]^+$ 353.1654, Found 353.1651.

(*E*)-5-Phenyl-*N*-(quinolin-8-yl)pent-4-enamide (5ab)

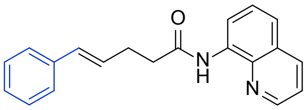

Following general procedure C, the reaction was carried out with alkene **1a** (0.2 mmol, 1.0 equiv.), phenylboronic acid **2b** (0.4 mmol, 2.0 equiv.), Ni(cod)$_2$ (0.02 mmol, 0.0055 g, 0.1 equiv.), CsOPiv (0.3 mmol, 0.0702 g, 1.5 equiv.), (*E*)-3-(3,4,5-trimethoxyphenyl)acrylaldehyde **A18** (0.2 mmol, 0.0444 g, 1.0 equiv.),

and n-Bu_3P (0.04 mmol, 0.0081 g, 0.2 equiv.) in anhydrous t-AmylOH (1 mL) at 70 °C for 48 h. (E)-5-Phenyl-N-(quinolin-8-yl)pent-4-enamide **(5ab)** was isolated by column chromatography (PE/EtOAc = 10/1) as a light yellow solid in 92 % yield. **M.P.**: 64–65 ºC. **^{1}H NMR (400 MHz, $CDCl_3$):** δ 9.90 (s, 1H), 8.84 (dd, J = 7.4, 0.9 Hz, 1H), 8.77 (dd, J = 4.2, 1.5 Hz, 1H), 8.16 (dd, J = 8.3, 1.4 Hz, 1H), 7.66–7.48 (m, 2H), 7.45 (dd, J = 8.3, 4.2 Hz, 1H), 7.38 (d, J = 7.3 Hz, 2H), 7.30 (dd, J = 13.2, 5.4 Hz, 2H), 7.23 (t, J = 7.2 Hz, 1H), 6.56 (d, J = 15.8 Hz, 1H), 6.36 (dt, J = 15.8, 4.5 Hz, 1H), 2.78–2.77 (m, 4H). **^{13}C NMR (100 MHz, $CDCl_3$):** δ 170.96, 148.20, 138.40, 137.50, 136.45, 134.55, 131.30, 128.73, 128.57, 128.02, 127.50, 127.20, 126.20, 121.68, 121.57, 116.60, 37.85, 28.97. **HRMS (ESI)** m/z Calcd. for $C_{20}H_{18}N_2O$ $[M+H]^+$ 303.1497, Found 303.1495.

(E)-N-(Quinolin-8-yl)-5-o-tolylpent-4-enamide (5ac)

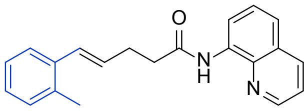

Following general procedure C, the reaction was carried out with alkene **1a** (0.2 mmol, 1.0 equiv.), o-tolylboronic acid **2c** (0.4 mmol, 2.0 equiv.), $Ni(cod)_2$ (0.02 mmol, 0.0055 g, 0.1 equiv.), CsOPiv (0.3 mmol, 0.0702 g, 1.5 equiv.), (E)-3-(3,4,5-trimethoxyphenyl)acrylaldehyde **A18** (0.2 mmol, 0.0444 g, 1.0 equiv.), and PMe_3 (0.04 mmol, 0.2 equiv.) in anhydrous t-AmylOH (1 mL) at 120 °C for 48 h. (E)-N-(Quinolin-8-yl)-5-o-tolylpent-4-enamide **(5ac)** was isolated by column chromatography (PE/EtOAc = 10/1) as a white solid in 78 % yield. **M.P.**: 101–102 ºC. **^{1}H NMR (400 MHz, $CDCl_3$):** δ 9.88 (s, 1H), 8.82 (d, J = 7.4 Hz, 1H), 8.76 (dd, J = 4.2, 1.5 Hz, 1H), 8.14 (dd, J = 8.3, 1.3 Hz, 1H), 7.60–7.46 (m, 2H), 7.43 (dd, J = 8.2, 4.1 Hz, 2H), 7.18–7.07 (m, 3H), 6.74 (d, J = 15.6 Hz, 1H), 6.27–6.13 (m, 1H), 2.77–2.76 (m, 4H), 2.30 (s, 3H). **^{13}C NMR (100 MHz, $CDCl_3$):** δ 170.97, 148.20, 138.41, 136.65, 136.43, 135.16, 134.55, 130.21, 130.02, 129.24, 128.01, 127.49, 127.14, 126.08, 125.69, 121.67, 121.56, 116.58, 38.05, 29.32, 19.87. **HRMS (ESI)** m/z Calcd. for $C_{21}H_{20}N_2O$ $[M+H]^+$ 317.1654, Found 317.1650.

(*E*)-5-(2-Fluorophenyl)-*N*-(quinolin-8-yl)pent-4-enamide (5ad)

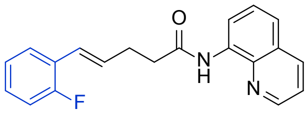

Following general procedure C, the reaction was carried out with alkene **1a** (0.2 mmol, 1.0 equiv.), 2-fluorophenylboronic acid **2d** (0.4 mmol, 2.0 equiv.), Ni(cod)$_2$ (0.02 mmol, 0.0055 g, 0.1 equiv.), CsOPiv (0.3 mmol, 0.0702 g, 1.5 equiv.), (*E*)-3-(3,4,5-trimethoxyphenyl)acrylaldehyde **A18** (0.2 mmol, 0.0444 g, 1.0 equiv.), and PMe$_3$ (0.04 mmol, 0.0081 g, 0.2 equiv.) in anhydrous *t*-AmylOH (1 mL) at 120 °C for 48 h. (*E*)-5-(2-Fluorophenyl)-*N*-(quinolin-8-yl)pent-4-enamide **(5ad)** was isolated by column chromatography (PE/EtOAc = 10/1) as a white solid in 84 % yield. **M.P.**: 57–58 °C. **^{1}H NMR (400 MHz, CDCl$_3$):** *δ* 9.87 (s, 1H), 8.89–8.70 (m, 2H), 8.14 (d, *J* = 8.2 Hz, 1H), 7.58–7.38 (m, 4H), 7.18–7.13 (m, 1H), 7.08–6.95 (m, 2H), 6.69 (d, J = 16.0 Hz, 1H), 6.41 (dt, J = 16.0, 6.7 Hz, 1H), 2.77–2.76 (m, 4H). **^{13}C NMR (100 MHz, CDCl$_3$):** *δ* 170.83, 160.07 (d, *J* = 248.6 Hz), 148.22, 138.41, 136.43, 134.54, 131.45 (d, *J* = 4.5 Hz), 128.41 (d, *J* = 8.4 Hz), 128.01, 127.49, 127.28 (d, *J* = 3.9 Hz), 125.29, 125.16, 124.09 (d, *J* = 3.4 Hz), 123.64 (d, *J* = 3.8 Hz), 121.64 (d, *J* = 11.4 Hz), 116.58, 115.69 (d, *J* = 22.2 Hz), 37.70, 29.32. **^{19}F NMR (376 MHz, CDCl$_3$):** *δ* -123.27–-123.35 (m). **HRMS (ESI)** m/z Calcd. for $C_{20}H_{17}FN_2O$ $[M+H]^+$ 321.1403, Found 321.1400.

(*E*)-*N*-(Quinolin-8-yl)-5-*m*-tolylpent-4-enamide (5ae)

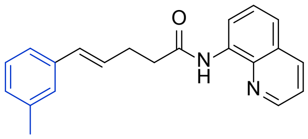

Following general procedure C, the reaction was carried out with alkene **1a** (0.2 mmol, 1.0 equiv.), *m*-tolylboronic acid **2e** (0.4 mmol, 2.0 equiv.), Ni(cod)$_2$ (0.02 mmol, 0.0055 g, 0.1 equiv.), CsOPiv (0.3 mmol, 0.0702 g, 1.5 equiv.), (*E*)-3-(3,4,5-trimethoxyphenyl)acrylaldehyde **A18** (0.2 mmol, 0.0444 g, 1.0 equiv.), and *n*-Bu$_3$P (0.04 mmol, 0.0081 g, 0.2 equiv.) in anhydrous *t*-AmylOH (1 mL) at 70 °C for 48 h. (*E*)-*N*-(Quinolin-8-yl)-5-*m*-tolylpent-4-enamide (**5ae**) was isolated by column chromatography (PE/EtOAc = 10/1) as a white solid in 91 % yield. **M.P.**: 96–97 ºC. **^{1}H NMR (400 MHz, CDCl$_3$):** *δ* 9.88 (s, 1H), 8.82 (dd, *J* = 7.4, 1.2 Hz, 1H), 8.75 (dd, *J* = 4.2, 1.6 Hz, 1H), 8.14 (dd, *J* = 8.3, 1.5 Hz, 1H), 7.61–7.47 (m, 2H), 7.43 (dd, *J* = 8.3, 4.2 Hz, 1H), 7.23–7.12 (m, 3H), 7.03–7.02 (m, 1H), 6.51 (d, *J* = 15.7 Hz,

1H), 6.41–6.24 (m, 1H), 2.75–2.74 (m, 4H), 2.32 (s, 3H). **^{13}C NMR (100 MHz, $CDCl_3$):** δ 170.97, 148.20, 138.41, 136.65, 136.43, 135.16, 134.55, 130.21, 130.02, 129.24, 128.01, 127.49, 127.14, 126.08, 125.69, 121.67, 121.56, 116.58, 38.05, 29.32, 19.87. **HRMS (ESI)** m/z Calcd. for $C_{21}H_{20}N_2O$ $[M+H]^+$ 317.1654, Found 317.1651.

(*E*)-5-(3-Methoxyphenyl)-*N*-(quinolin-8-yl)pent-4-enamide (5af)

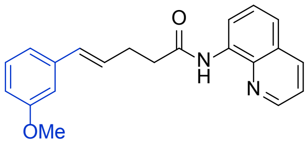

Following general procedure C, the reaction was carried out with alkene **1a** (0.2 mmol, 1.0 equiv.), 3-methoxyphenylboronic acid **2f** (0.4 mmol, 2.0 equiv.), $Ni(cod)_2$ (0.02 mmol, 0.0055 g, 0.1 equiv.), CsOPiv (0.3 mmol, 0.0702 g, 1.5 equiv.), (*E*)-3-(3,4,5-trimethoxyphenyl)acrylaldehyde **A18** (0.2 mmol, 0.0444 g, 1.0 equiv.), and *n*-Bu_3P (0.04 mmol, 0.0081 g, 0.2 equiv.) in anhydrous *t*-AmylOH (1 mL) at 70 °C for 48 h. (*E*)-5-(3-Methoxyphenyl)-*N*-(quinolin-8-yl) pent-4-enamide **(5af)** was isolated by column chromatography (PE/EtOAc = 10/1) as a brown oil in 93 % yield. **^{1}H NMR (400 MHz, $CDCl_3$):** δ 9.87 (s, 1H), 8.80 (dd, J = 7.4, 1.3 Hz, 1H), 8.74 (dd, J = 4.2, 1.6 Hz, 1H), 8.13 (dd, J = 8.3, 1.6 Hz, 1H), 7.59–7.45 (m, 2H), 7.42 (dd, J = 8.3, 4.2 Hz, 1H), 7.20 (t, J = 7.9 Hz, 1H), 6.95 (d, J = 7.7 Hz, 1H), 6.91–6.86 (m, 1H), 6.76 (dd, J = 8.1, 2.2 Hz, 1H), 6.50 (d, J = 15.7 Hz, 1H), 6.39–6.26 (m, 1H), 3.78 (s, 3H), 2.74–2.71 (m, 4H). **^{13}C NMR (100 MHz, $CDCl_3$):** δ 170.89, 159.80, 148.18, 138.94, 138.34, 136.41, 134.49, 131.16, 129.50, 129.05, 127.97, 127.44, 121.64, 121.55, 118.85, 116.55, 112.86, 111.45, 55.22, 37.74, 28.88. **HRMS (ESI)** m/z Calcd. for $C_{21}H_{20}N_2O_2$ $[M+H]^+$ 333.1603, Found 333.1600.

(*E*)-5-(3-Chlorophenyl)-*N*-(quinolin-8-yl)pent-4-enamide (5ag)

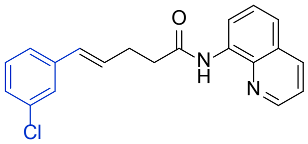

Following general procedure C, the reaction was carried out with alkene **1a** (0.2 mmol, 1.0 equiv.), 3-chlorophenylboronic acid **2 g** (0.4 mmol, 2.0 equiv.), $Ni(cod)_2$ (0.02 mmol, 0.0055 g, 0.1 equiv.), CsOPiv (0.3 mmol, 0.0702 g, 1.5 equiv.), (*E*)-3-(3,4,5-trimethoxyphenyl)acrylaldehyde **A18** (0.2 mmol, 0.0444 g, 1.0 equiv.), and *n*-Bu_3P (0.04 mmol, 0.0081 g, 0.2 equiv.) in anhydrous *t*-AmylOH (1 mL) at 70 °C for 48 h. (*E*)-5-(3-Chlorophenyl)-*N*-(quinolin-8-yl) pent-4-enamide **(5ag)** was

isolated by column chromatography (PE/EtOAc = 10/1) as a light yellow solid in 91 % yield. **M.P.**: 44–45 ºC. **^{1}H NMR (400 MHz, $CDCl_3$):** δ 9.85 (s, 1H), 8.80 (dd, J = 7.4, 1.1 Hz, 1H), 8.75 (dd, J = 4.2, 1.5 Hz, 1H), 8.13 (dd, J = 8.3, 1.5 Hz, 1H), 7.59–7.46 (m, 2H), 7.42 (dd, J = 8.3, 4.2 Hz, 1H), 7.32 (s, 1H), 7.16 (ddd, J = 8.0, 6.5, 3.5 Hz, 3H), 6.45 (d, J = 15.8 Hz, 1H), 6.39–6.27 (m, 1H), 2.94–2.58 (m, 4H). **^{13}C NMR (100 MHz, $CDCl_3$):** δ 170.72, 148.21, 139.36, 138.35, 136.44, 134.45, 130.38, 130.02, 129.74, 128.00, 127.46, 127.09, 126.07, 124.44, 121.68, 121.61, 116.59, 37.59, 28.85. **HRMS (ESI)** m/z Calcd. for $C_{20}H_{17}ClN_2O$ $[M+H]^+$ 337.1108 (100.0%), 339.1078 (32.0%), Found 337.1105 (100.0%), 339.1054 (32.0%).

(*E*)-5-(3-Fluorophenyl)-*N*-(quinolin-8-yl)pent-4-enamide (5ah)

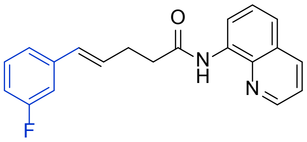

Following general procedure C, the reaction was carried out with alkene **1a** (0.2 mmol, 1.0 equiv.), 3-fluorophenylboronic acid **2h** (0.4 mmol, 2.0 equiv.), Ni(cod)$_2$ (0.02 mmol, 0.0055 g, 0.1 equiv.), CsOPiv (0.3 mmol, 0.0702 g, 1.5 equiv.), (*E*)-3-(3,4,5-trimethoxyphenyl)acrylaldehyde **A18** (0.2 mmol, 0.0444 g, 1.0 equiv.), and *n*-Bu$_3$P (0.04 mmol, 0.0081 g, 0.2 equiv.) in anhydrous *t*-AmylOH (1 mL) at 70 °C for 48 h. (*E*)-5-(3-Fluorophenyl)-*N*-(quinolin-8-yl) pent-4-enamide **(5ah)** was isolated by column chromatography (PE/EtOAc = 10/1) as a brown oil in 88 % yield. **^{1}H NMR (400 MHz, $CDCl_3$):** δ 9.75 (s, 1H), 8.66 (dd, J = 23.6, 5.7 Hz, 2H), 8.01 (d, J = 8.2 Hz, 1H), 7.53–7.26 (m, 3H), 7.20–7.05 (m, 1H), 6.95 (dd, J = 15.7, 9.0 Hz, 2H), 6.88–6.72 (m, 1H), 6.36 (d, J = 15.7 Hz, 1H), 6.28–6.11 (m, 1H), 2.62–2.57 (m, 4H). **^{13}C NMR (100 MHz, $CDCl_3$):** δ 170.74, 163.11 (d, J = 244.9 Hz), 148.18, 139.84 (d, J = 7.7 Hz), 138.33, 136.41, 134.44, 130.20, 129.92 (d, J = 8.5 Hz), 127.97, 127.42, 122.07, 122.05, 121.65, 121.60, 116.59, 113.90 (d, J = 21.4 Hz), 112.56 (d, J = 21.7 Hz), 37.55, 28.78. **^{19}F NMR (376 MHz, $CDCl_3$):** δ –118.47 (dd, J = 15.4, 8.8 Hz). **HRMS (ESI)** m/z Calcd. for $C_{20}H_{17}FN_2O$ $[M+H]^+$ 321.1403, Found 321.1399.

(*E*)-5-(3-Cyanophenyl)-*N*-(quinolin-8-yl)pent-4-enamide (5ai)

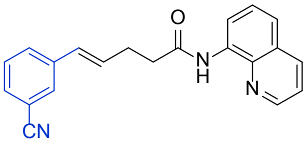

Following general procedure C, the reaction was carried out with alkene **1a** (0.2 mmol, 1.0 equiv.),

3-cyanophenylboronic acid **2i** (0.4 mmol, 2.0 equiv.), Ni(cod)$_2$ (0.02 mmol, 0.0055 g, 0.1 equiv.), CsOPiv (0.3 mmol, 0.0702 g, 1.5 equiv.), (*E*)-3-(3,4,5-trimethoxyphenyl)acrylaldehyde **A18** (0.2 mmol, 0.0444 g, 1.0 equiv.), and *n*-Bu$_3$P (0.04 mmol, 0.0081 g, 0.2 equiv.) in anhydrous *t*-AmylOH (1 mL) at 70 °C for 48 h. (*E*)-5-(3-Cyanophenyl)-*N*-(quinolin-8-yl) pent-4-enamide **(5ai)** was isolated by column chromatography (PE/EtOAc = 10/1) as a brown oil in 83 % yield. **^{1}H NMR (400 MHz, CDCl$_3$):** δ 9.85 (s, 1H), 8.76 (dd, *J* = 11.8, 4.9 Hz, 2H), 8.13 (d, *J* = 9.3 Hz, 1H), 7.63–7.46 (m, 4H), 7.46–7.38 (m, 2H), 7.33 (t, *J* = 7.7 Hz, 1H), 6.47 (d, J = 15.9 Hz, 1H), 6.41–6.34 (m, 1H), 2.75–2.70 (m, 4H). **^{13}C NMR (100 MHz, CDCl$_3$):** δ 170.54, 148.21, 138.63, 138.28, 136.46, 134.34, 131.73, 130.38, 130.33, 129.56, 129.28, 129.19, 127.97, 127.42, 121.71, 121.67, 118.90, 116.58, 112.60, 37.33, 28.76. **HRMS (ESI)** m/z Calcd. for $C_{21}H_{17}N_3O$ $[M+H]^+$ 328.1450, Found 328.1447.

(*E*)-Methyl 3-(5-oxo-5-(quinolin-8-ylamino)pent-1-enyl)benzoate (5aj)

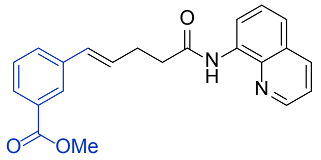

Following general procedure C, the reaction was carried out with alkene **1a** (0.2 mmol, 1.0 equiv.), 3-(methoxycarbonyl)phenylboronic acid **2j** (0.4 mmol, 2.0 equiv.), Ni(cod)$_2$ (0.02 mmol, 0.0055 g, 0.1 equiv.), CsOPiv (0.3 mmol, 0.0702 g, 1.5 equiv.), (*E*)-3-(3,4,5-trimethoxyphenyl)acrylaldehyde **A18** (0.2 mmol, 0.0444 g, 1.0 equiv.), and *n*-Bu$_3$P (0.04 mmol, 0.0081 g, 0.2 equiv.) in anhydrous *t*-AmylOH (1.5 mL) at 70 °C for 48 h. (*E*)-Methyl 3-(5-oxo-5-(quinolin-8-ylamino)pent-1-enyl)benzoate **(5aj)** was isolated by column chromatography (PE/EtOAc = 10/1) as a light brown solid in 87 % yield. **M.P.**: 56–57 °C. **^{1}H NMR (400 MHz, CDCl$_3$):** δ 9.86 (s, 1H), 8.78 (dd, *J* = 7.4, 1.1 Hz, 1H), 8.72 (dd, *J* = 4.2, 1.6 Hz, 1H), 8.11 (dd, *J* = 8.3, 1.5 Hz, 1H), 8.01 (s, 1H), 7.85 (d, *J* = 7.7 Hz, 1H), 7.56–7.44 (m, 3H), 7.40 (dd, *J* = 8.2, 4.2 Hz, 1H), 7.32 (t, *J* = 7.7 Hz, 1H), 6.53 (d, *J* = 15.8 Hz, 1H), 6.42–6.35 (m, 1H), 3.88 (s, 3H), 2.78–2.68 (m, 4H). **^{13}C NMR (100 MHz, CDCl$_3$):** δ 170.80, 167.12, 148.17, 138.31, 137.75, 136.40, 134.41, 130.54, 130.37, 130.31, 130.08, 128.57, 128.15, 127.95, 127.41, 127.19, 121.64,

121.59, 116.56, 52.17, 37.57, 28.84. **HRMS (ESI)** m/z Calcd. for $C_{22}H_{20}N_2O_3$ $[M+H]^+$ 361.1552, Found 361.1550.

(*E*)-*N*-(Quinolin-8-yl)-5-*p*-tolylpent-4-enamide (5ak)

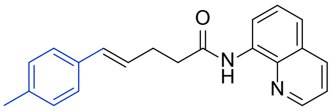

Following general procedure C, the reaction was carried out with alkene **1a** (0.2 mmol, 1.0 equiv.), *p*-tolylboronic acid **2k** (0.4 mmol, 2.0 equiv.), $Ni(cod)_2$ (0.02 mmol, 0.0055 g, 0.1 equiv.), CsOPiv (0.3 mmol, 0.0702 g, 1.5 equiv.), (*E*)-3-(3,4,5-trimethoxyphenyl)acrylaldehyde **A18** (0.2 mmol, 0.0444 g, 1.0 equiv.), and *n*-Bu_3P (0.04 mmol, 0.0081 g, 0.2 equiv.) in anhydrous *t*-AmylOH (1 mL) at 70 °C for 48 h. (*E*)-*N*-(Quinolin-8-yl)-5-*p*-tolylpent-4-enamide **(5ak)** was isolated by column chromatography (PE/EtOAc = 10/1) as a white solid in 89 % yield. **M.P.**: 96–97 ºC. **^{1}H NMR (400 MHz, $CDCl_3$):** δ 9.88 (s, 1H), 8.83 (dd, *J* = 7.5, 1.1 Hz, 1H), 8.76 (dd, *J* = 4.2, 1.6 Hz, 1H), 8.14 (dd, *J* = 8.3, 1.5 Hz, 1H), 7.59–7.47 (m, 2H), 7.43 (dd, *J* = 8.3, 4.2 Hz, 1H), 7.27 (d, *J* = 8.0 Hz, 2H), 7.11 (d, *J* = 7.9 Hz, 2H), 6.52 (d, *J* = 15.8 Hz, 1H), 6.33–6.26 (m, 1H), 2.77–2.71 (m, 4H), 2.34 (s, 3H). **^{13}C NMR (100 MHz, $CDCl_3$):** δ 170.98, 148.17, 138.40, 136.89, 136.40, 134.72, 134.56, 131.12, 129.24, 127.99, 127.64, 127.47, 126.08, 121.64, 121.51, 116.57, 37.93, 28.96, 21.22. **HRMS (ESI)** m/z $C_{21}H_{20}N_2O$ $[M+H]^+$ 317.1654, Found 317.1651.

(*E*)-*N*-(Quinolin-8-yl)-5-(4-(trifluoromethoxy)phenyl)pent-4-enamide (5al)

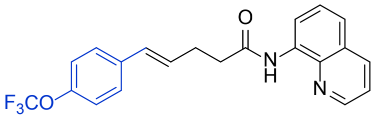

Following general procedure C, the reaction was carried out with alkene **1a** (0.2 mmol, 1.0 equiv.), 4-(trifluoromethoxy) phenylboronic acid **2l** (0.4 mmol, 2.0 equiv.), $Ni(cod)_2$ (0.02 mmol, 0.0055 g, 0.1 equiv.), CsOPiv (0.3 mmol, 0.0702 g, 1.5 equiv.), (*E*)-3-(3,4,5-trimethoxyphenyl)acrylaldehyde **A18** (0.2 mmol, 0.0444 g, 1.0 equiv.), and *n*-Bu_3P (0.04 mmol, 0.0081 g, 0.2 equiv.) in anhydrous *t*-AmylOH (1 mL) at 70 °C for 48 h. (*E*)-*N*-(Quinolin-8-yl)-5-(4-(trifluoromethoxy)phenyl)pent-4-enamide **(5al)** was isolated by column chromatography (PE/EtOAc = 10/1) as a brown solid in 89 % yield. **M.P.**: 48–49 ºC. **^{1}H NMR (400 MHz, $CDCl_3$):** δ 9.86 (s, 1H), 8.80 (dd, *J* = 7.4, 1.3 Hz, 1H), 8.73 (dd, *J* = 4.2, 1.6 Hz, 1H), 8.14 (dd, *J* = 8.3, 1.6 Hz, 1H), 7.57–7.47

(m, 2H), 7.43 (dd, J = 8.3, 4.2 Hz, 1H), 7.34 (d, J = 8.7 Hz, 2H), 7.11 (d, J = 8.2 Hz, 2H), 6.50 (d, J = 15.8 Hz, 1H), 6.37–6.25 (m, 1H), 2.85–2.65 (m, 4H). **^{13}C NMR (100 MHz, $CDCl_3$):** δ 170.80, 148.21, 138.40, 136.50, 136.33, 134.51, 129.93, 129.91, 128.05, 127.52, 127.39, 121.71, 121.64, 121.11, 120.58 (q, J = 257.0 Hz), 116.63, 37.70, 28.90. **^{19}F NMR (376 MHz, $CDCl_3$):** δ –62.58. **HRMS (ESI)** m/z Calcd. for $C_{21}H_{17}F_3N_2O_2$ $[M+H]^+$ 387.1320, Found 387.1318.

(*E*)-5-(Biphenyl-4-yl)-*N*-(quinolin-8-yl)pent-4-enamide (5am)

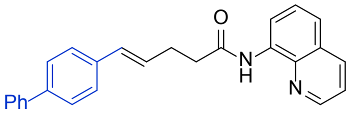

Following general procedure C, the reaction was carried out with alkene **1a** (0.2 mmol, 1.0 equiv.), biphenyl-4-ylboronic acid **2m** (0.4 mmol, 2.0 equiv.), Ni(cod)$_2$ (0.02 mmol, 0.0055 g, 0.1 equiv.), CsOPiv (0.3 mmol, 0.0702 g, 1.5 equiv.), (*E*)-3-(3,4,5-trimethoxyphenyl)acrylaldehyde **A18** (0.2 mmol, 0.0444 g, 1.0 equiv.), and *n*-Bu$_3$P (0.04 mmol, 0.0081 g, 0.2 equiv.) in anhydrous *t*-AmylOH (1 mL) at 70 °C for 48 h. (*E*)-5-(Biphenyl-4-yl)-*N*-(quinolin-8-yl)pent-4-enamide (**5am**) was isolated by column chromatography (PE/EtOAc = 10/1) as a light yellow solid in 78 % yield. **M.P.**: 135–136 ºC. **^{1}H NMR (400 MHz, $CDCl_3$):** δ 9.90 (s, 1H), 8.84 (d, J = 6.9 Hz, 1H), 8.76 (dd, J = 4.1, 1.4 Hz, 1H), 8.14 (dd, J = 8.3, 1.3 Hz, 1H), 7.59 (t, J = 6.6 Hz, 2H), 7.56–7.48 (m, 4H), 7.44 (t, J = 7.7 Hz, 5H), 7.34 (t, J = 7.3 Hz, 1H), 6.58 (d, J = 15.8 Hz, 1H), 6.49–6.28 (m, 1H), 2.78–2.76 (m, 4H). **^{13}C NMR (100 MHz, $CDCl_3$):** δ 170.96, 148.21, 140.84, 139.90, 138.41, 136.56, 136.44, 134.53, 130.87, 128.89, 128.84, 128.01, 127.49, 127.29, 127.24, 126.96, 126.62, 121.68, 121.59, 116.62, 37.85, 29.04. **HRMS (ESI)** m/z Calcd. for $C_{26}H_{22}N_2O$ $[M+H]^+$ 379.1810, Found 379.1808.

(*E*)-5-(4-Acetylphenyl)-*N*-(quinolin-8-yl)pent-4-enamide (5an)

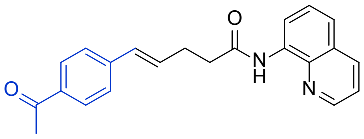

Following general procedure C, the reaction was carried out with alkene **1a** (0.2 mmol, 1.0 equiv.), 4-acetylphenylboronic acid **2n** (0.4 mmol, 2.0 equiv.), Ni(cod)$_2$ (0.02 mmol, 0.0055 g, 0.1 equiv.), CsOPiv (0.3 mmol, 0.0702 g, 1.5 equiv.),

(*E*)-3-(3,4,5-trimethoxyphenyl)acrylaldehyde **A18** (0.2 mmol, 0.0444 g, 1.0 equiv.), and *n*-Bu_3P (0.04 mmol, 0.0081 g, 0.2 equiv.) in anhydrous *t*-AmylOH (1 mL) at 70 °C for 48 h. (*E*)-5-(4-Acetylphenyl)-*N*-(quinolin-8-yl)pent-4-enamide **(5an)** was isolated by column chromatography (PE/EtOAc = 10/1) as a brown solid in 85 % yield. **M.P.**: 94–95 ºC. **^{1}H NMR (400 MHz, $CDCl_3$):** δ 9.84 (s, 1H), 8.78 (d, *J* = 7.2 Hz, 1H), 8.73 (dd, *J* = 4.1, 1.3 Hz, 1H), 8.12 (d, *J* = 8.2 Hz, 1H), 7.85 (d, *J* = 8.3 Hz, 2H), 7.56–7.44 (m, 2H), 7.44–7.34 (m, 3H), 6.54 (d, *J* = 15.9 Hz, 1H), 6.50–6.39 (m, 1H), 2.75–2.74 (m, 4H), 2.54 (s, 3H). **^{13}C NMR (100 MHz, $CDCl_3$):** δ 197.58, 170.59, 148.17, 142.14, 138.32, 136.42, 135.69, 134.43, 131.99, 130.38, 128.74, 127.97, 127.43, 126.18, 121.67, 121.60, 116.54, 37.41, 28.93, 26.59. **HRMS (ESI)** m/z Calcd. for $C_{22}H_{20}N_2O_2$ $[M+H]^+$ 345.1603, Found 345.1600.

(*E*)-*N*-(Quinolin-8-yl)-5-(4-(trifluoromethyl)phenyl)pent-4-enamide (5ao)

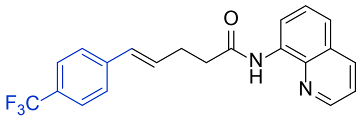

Following general procedure C, the reaction was carried out with alkene **1a** (0.2 mmol, 1.0 equiv.), 4-(trifluoromethyl)phenylboronic acid **2o** (0.4 mmol, 2.0 equiv.), $Ni(cod)_2$ (0.02 mmol, 0.0055 g, 0.1 equiv.), CsOPiv (0.3 mmol, 0.0702 g, 1.5 equiv.), (*E*)-3-(3,4,5-trimethoxyphenyl)acrylaldehyde **A18** (0.2 mmol, 0.0444 g, 1.0 equiv.), and *n*-Bu_3P (0.04 mmol, 0.0081 g, 0.2 equiv.) in anhydrous *t*-AmylOH (1 mL) at 70 °C for 48 h. (*E*)-*N*-(Quinolin-8-yl)-5-(4-(trifluoromethyl) phenyl)pent-4-enamide **(5ao)** was isolated by column chromatography (PE/EtOAc = 10/1) as a white solid in 86 % yield. **M.P.**: 80–81 ºC. **^{1}H NMR (400 MHz, $CDCl_3$):** δ 9.86 (s, 1H), 8.80 (dd, *J* = 7.3, 1.2 Hz, 1H), 8.74 (dd, *J* = 4.2, 1.5 Hz, 1H), 8.14 (dd, *J* = 8.3, 1.5 Hz, 1H), 7.58–7.47 (m, 4H), 7.45–7.40 (m, 3H), 6.54 (d, *J* = 15.9 Hz, 1H), 6.50–6.36 (m, 1H), 2.77–2.76 (m, 4H). **^{13}C NMR (100 MHz, $CDCl_3$):** δ 170.69, 148.22, 140.99, 138.39, 136.53, 134.48, 131.65, 130.12, 128.98 (q, *J* = 32.4 Hz), 128.06, 127.53, 126.34, 125.52 (q, *J* = 3.8 Hz), 124.36 (q, *J* = 271.7 Hz), 121.74, 121.68, 116.65, 37.52, 28.92. **^{19}F NMR (376 MHz, $CDCl_3$):** δ –66.86. **HRMS (ESI)** m/z Calcd. for $C_{21}H_{17}F_3N_2O$ $[M+H]^+$ 371.1371, Found 371.1368.

(*E*)-5-(3,4-Dimethylphenyl)-*N*-(quinolin-8-yl)pent-4-enamide (5ap)

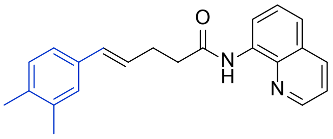

Following general procedure C, the reaction was carried out with alkene **1a** (0.2 mmol, 1.0 equiv.), 3,4-dimethylphenylboronic acid **2q** (0.4 mmol, 2.0 equiv.), $Ni(cod)_2$ (0.02 mmol, 0.0055 g, 0.1 equiv.), CsOPiv (0.3 mmol, 0.0702 g, 1.5 equiv.), (*E*)-3-(3,4,5-trimethoxyphenyl)acrylaldehyde **A18** (0.2 mmol, 0.0444 g, 1.0 equiv.), and *n*-Bu_3P (0.04 mmol, 0.0081 g, 0.2 equiv.) in anhydrous *t*-AmylOH (1 mL) at 70 °C for 48 h. (*E*)-5-(3,4-Dimethylphenyl)-*N*-(quinolin-8-yl)pent-4-enamide **(5ap)** was isolated by column chromatography (PE/EtOAc = 10/1) as a light brown solid in 95 % yield. **M.P.**: 91–92 ºC. **^{1}H NMR (400 MHz, $CDCl_3$):** *δ* 9.88 (s, 1H), 8.83 (dd, *J* = 7.5, 1.0 Hz, 1H), 8.76 (dd, *J* = 4.2, 1.6 Hz, 1H), 8.13 (dd, *J* = 8.3, 1.5 Hz, 1H), 7.62–7.47 (m, 2H), 7.42 (dd, *J* = 8.3, 4.2 Hz, 1H), 7.20–7.02 (m, 3H), 6.49 (d, *J* = 15.7 Hz, 1H), 6.39–6.19 (m, 1H), 2.75–2.74 (m, 4H), 2.24 (s, 6H). **^{13}C NMR (100 MHz, $CDCl_3$):** *δ* 171.08, 148.15, 138.34, 136.54, 136.39, 135.57, 135.11, 134.48, 131.16, 129.78, 127.95, 127.41, 123.63, 121.61, 121.53, 121.42, 116.57, 116.48, 37.93, 28.97, 19.81, 19.53. **HRMS (ESI)** m/z Calcd. for $C_{22}H_{22}N_2O$ $[M+H]^+$ 331.1810, Found 331.1808.

(*E*)-5-(Benzo[*d*][1,3]dioxol-5-yl)-*N*-(quinolin-8-yl)pent-4-enamide (5aq)

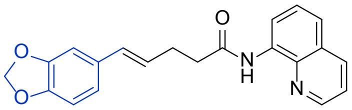

Following general procedure C, the reaction was carried out with alkene **1a** (0.2 mmol, 1.0 equiv.), benzo[*d*][1,3]dioxol-5-ylboronic acid **2q** (0.4 mmol, 2.0 equiv.), $Ni(cod)_2$ (0.02 mmol, 0.0055 g, 0.1 equiv.), CsOPiv (0.3 mmol, 0.0702 g, 1.5 equiv.), (*E*)-3-(3,4,5-trimethoxyphenyl)acrylaldehyde **A18** (0.2 mmol, 0.0444 g, 1.0 equiv.), and *n*-Bu_3P (0.04 mmol, 0.0081 g, 0.2 equiv.) in anhydrous *t*-AmylOH (1 mL) at 70 °C for 48 h. (*E*)-5-(Benzo[*d*][1,3]dioxol-5-yl)-*N*-(quinolin-8-yl) pent-4-enamide **(5aq)** was isolated by column chromatography (PE/EtOAc = 10/1) as a white solid in 87 % yield. **M.P.**: 104–105 ºC. **^{1}H NMR (400 MHz, $CDCl_3$):** *δ* 9.85 (s, 1H), 8.79 (dd, J = 7.4, 1.3 Hz, 1H), 8.75 (dd, J = 4.2, 1.6 Hz, 1H)., 8.13 (dd, *J* = 8.3, 1.6 Hz, 1H), 7.58–7.45 (m, 2H), 7.43 (dd, *J* = 8.3, 4.2 Hz, 1H), 6.90 (d, *J* = 1.4 Hz, 1H), 6.77–6.70 (m, 2H), 6.43 (d, *J* = 15.7 Hz, 1H), 6.18–6.11 (m, 1H), 5.91 (s, 2H), 2.83–2.61 (m,

4H). **^{13}C NMR (100 MHz, CDCl$_3$):** δ 170.98, 148.21, 148.00, 146.88, 138.40, 136.45, 134.54, 132.03, 130.86, 128.02, 127.50, 126.97, 121.67, 121.55, 120.63, 116.59, 108.28, 105.62, 101.04, 37.96, 28.88. **HRMS (ESI)** m/z Calcd. for $C_{21}H_{18}N_2O_3$ [M+H]$^+$ 347.1396, Found 347.1393.

(*E*)-5-(3,5-Dimethylphenyl)-*N*-(quinolin-8-yl)pent-4-enamide (5ar)

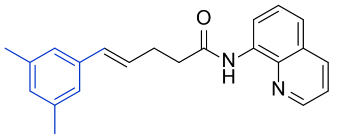

Following general procedure C, the reaction was carried out with alkene **1a** (0.2 mmol, 1.0 equiv.), 3,5-dimethylphenylboronic acid **2r** (0.4 mmol, 2.0 equiv.), Ni(cod)$_2$ (0.02 mmol, 0.0055 g, 0.1 equiv.), CsOPiv (0.3 mmol, 0.0702 g, 1.5 equiv.), (*E*)-3-(3,4,5-trimethoxyphenyl)acrylaldehyde **A18** (0.2 mmol, 0.0444 g, 1.0 equiv.), and *n*-Bu$_3$P (0.04 mmol, 0.0081 g, 0.2 equiv.) in anhydrous *t*-AmylOH (1 mL) at 70 °C for 48 h. (*E*)-5-(3,5-Dimethylphenyl)-*N*-(quinolin-8-yl)pent-4-enamide **(5ar)** was isolated by column chromatography (PE/EtOAc = 10/1) as a light brown solid in 92 % yield. **M.P.**: 50–51 ºC. **^{1}H NMR (400 MHz, CDCl$_3$):** δ 9.88 (s, 1H), 8.82 (d, *J* = 7.3 Hz, 1H), 8.76 (dd, *J* = 4.1, 1.4 Hz, 1H), 8.14 (dd, *J* = 8.2, 1.3 Hz, 1H), 7.53 (dt, *J* = 16.3, 8.1 Hz, 2H), 7.43 (dd, *J* = 8.3, 4.2 Hz, 1H), 6.99 (s, 2H), 6.85 (s, 1H), 6.48 (d, *J* = 15.8 Hz, 1H), 6.35–6.28 (m, 1H), 2.84–2.69 (m, 4H), 2.29 (s, 6H). **^{13}C NMR (100 MHz, CDCl$_3$):** δ 171.00, 148.18, 138.39, 137.96, 137.40, 136.43, 134.56, 131.41, 128.93, 128.31, 128.01, 127.49, 124.11, 121.65, 121.53, 116.58, 37.94, 29.02, 21.34. **HRMS (ESI)** m/z Calcd. for $C_{22}H_{22}N_2O$ [M+H]$^+$ 331.1810, Found 331.1808.

(*E*)-5-(1*H*-Indol-5-yl)-*N*-(quinolin-8-yl)pent-4-enamide (5as)

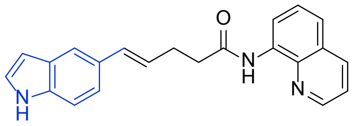

Following general procedure C, the reaction was carried out with alkene **1a** (0.2 mmol, 1.0 equiv.), 1*H*-indol-5-ylboronic acid **2s** (0.4 mmol, 2.0 equiv.), Ni(cod)$_2$ (0.02 mmol, 0.0055 g, 0.1 equiv.), CsOPiv (0.3 mmol, 0.0702 g, 1.5 equiv.), (*E*)-3-(3,4,5-trimethoxyphenyl)acrylaldehyde **A18** (0.2 mmol, 0.0444 g, 1.0 equiv.), and *n*-Bu$_3$P (0.04 mmol, 0.0081 g, 0.2 equiv.) in anhydrous *t*-AmylOH (1.5 mL) at 70 °C for 48 h. (*E*)-5-(1*H*-Indol-5-yl)-*N*-(quinolin-8-yl)pent-4-enamide **(5as)** was isolated by column chromatography (PE/EtOAc = 10/1) as a light brown solid in 71 %

yield. **M.P.**: 95–96 ºC. **^{1}H NMR (400 MHz, CDCl$_3$):** δ 9.91 (s, 1H), 8.82 (d, J = 7.2 Hz, 1H), 8.75 (d, J = 2.9 Hz, 1H), 8.26 (br, 1H), 8.15 (d, J = 8.1 Hz, 1H), 7.59 (s, 1H), 7.57–7.47 (m, 2H), 7.43 (dd, J = 8.2, 4.2 Hz, 1H), 7.31–7.26 (m, 2H), 7.16 (s, 1H), 6.65 (d, J = 15.7 Hz, 1H), 6.50 (s, 1H), 6.36–6.14 (m, 1H), 2.89–2.55 (m, 4H). **^{13}C NMR (100 MHz, CDCl$_3$):** δ 171.36, 148.23, 138.43, 136.50, 135.42, 134.61, 132.35, 129.69, 128.20, 128.06, 127.54, 125.79, 124.70, 121.68, 121.57, 120.50, 118.83, 116.68, 111.18, 102.89, 38.30, 29.19. **HRMS (ESI)** m/z Calcd. for $C_{22}H_{19}N_3O$ $[M+H]^+$ 342.1606, Found 342.1603.

(*E*)-5-(1-Methyl-1H-indol-5-yl)-*N*-(quinolin-8-yl)pent-4-enamide (5at)

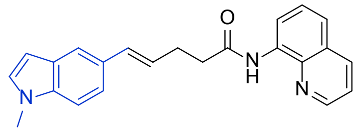

Following general procedure C, the reaction was carried out with alkene **1a** (0.2 mmol, 1.0 equiv.), 1-methyl-1*H*-indol-5-ylboronic acid **2t** (0.4 mmol, 2.0 equiv.), Ni(cod)$_2$ (0.02 mmol, 0.0055 g, 0.1 equiv.), CsOPiv (0.3 mmol, 0.0702 g, 1.5 equiv.), (*E*)-3-(3,4,5-trimethoxyphenyl)acrylaldehyde **A18** (0.2 mmol, 0.0444 g, 1.0 equiv.), and *n*-Bu$_3$P (0.04 mmol, 0.0081 g, 0.2 equiv.) in anhydrous *t*-AmylOH (1.5 mL) at 70 °C for 48 h. (*E*)-5-(1-Methyl-1*H*-indol-5-yl)-*N*-(quinolin-8-yl)pent-4-enamide **(5at)** was isolated by column chromatography (PE/EtOAc = 10/1) as a brown solid in 84 % yield. **M.P.**: 134–135 ºC. **^{1}H NMR (400 MHz, CDCl$_3$):** δ 9.90 (s, 1H), 8.83 (d, J = 7.3 Hz, 1H), 8.76 (dd, J = 4.1, 1.4 Hz, 1H), 8.14 (dd, J = 8.3, 1.4 Hz, 1H), 7.62–7.46 (m, 3H), 7.43 (dd, J = 8.3, 4.2 Hz, 1H), 7.32 (d, J = 8.5 Hz, 1H), 7.23 (d, J = 8.5 Hz, 1H), 7.01 (d, J = 3.0 Hz, 1H), 6.66 (d, J = 15.7 Hz, 1H), 6.44 (d, J = 2.9 Hz, 1H), 6.34–6.16 (m, 1H), 3.75 (s, 3H), 2.87–2.63 (m, 4H). **^{13}C NMR (100 MHz, CDCl$_3$):** δ 171.28, 148.22, 138.45, 136.43, 136.34, 134.65, 132.36, 129.28, 129.17, 128.74, 128.03, 127.52, 125.65, 121.66, 121.50, 120.02, 119.00, 116.60, 109.32, 101.23, 38.31, 32.97, 29.19. **HRMS (ESI)** m/z Calcd. for $C_{23}H_{21}N_3O$ $[M+H]^+$ 356.1763, Found 356.1760.

(*E*)-5-(Benzofuran-5-yl)-*N*-(quinolin-8-yl)pent-4-enamide (5au)

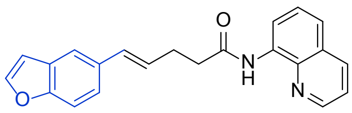

Following general procedure C, the reaction was carried

out with alkene **1a** (0.2 mmol, 1.0 equiv.), benzofuran-5-ylboronic acid **2u** (0.4 mmol, 2.0 equiv.), Ni(cod)$_2$ (0.02 mmol, 0.0055 g, 0.1 equiv.), CsOPiv (0.3 mmol, 0.0702 g, 1.5 equiv.), (*E*)-3-(3,4,5-trimethoxyphenyl)acrylaldehyde **A18** (0.2 mmol, 0.0444 g, 1.0 equiv.), and *n*-Bu$_3$P (0.04 mmol, 0.0081 g, 0.2 equiv.) in anhydrous *t*-AmylOH (1 mL) at 70 °C for 48 h. (*E*)-5-(Benzofuran-5-yl)-*N*-(quinolin-8-yl)pent-4-enamide **(5au)** was isolated by column chromatography (PE/EtOAc = 10/1) as a light yellow solid in 84 % yield. **M.P.**: 80–81 ºC. **^{1}H NMR (400 MHz, CDCl$_3$):** δ 9.88 (s, 1H), 8.82 (dd, *J* = 7.5, 1.2 Hz, 1H), 8.74 (dd, *J* = 4.2, 1.6 Hz, 1H), 8.13 (dd, *J* = 8.3, 1.6 Hz, 1H), 7.58 (d, *J* = 2.2 Hz, 1H), 7.54 (t, J = 7.9 Hz, 2H), 7.49 (dd, *J* = 8.3, 1.4 Hz, 1H), 7.44–7.39 (m, 2H), 7.32 (dd, *J* = 8.6, 1.7 Hz, 1H), 6.71 (dd, *J* = 2.1, 0.7 Hz, 1H), 6.62 (d, *J* = 15.7 Hz, 1H), 6.38–6.20 (m, 1H), 2.85–2.70 (m, 4H). **^{13}C NMR (100 MHz, CDCl$_3$):** δ 171.04, 154.47, 148.19, 145.43, 138.40, 136.45, 134.56, 132.65, 131.39, 128.02, 127.75, 127.59, 127.50, 122.76, 121.66, 121.56, 118.79, 116.60, 111.35, 106.73, 38.02, 29.03. **HRMS (ESI)** m/z Calcd. for $C_{22}H_{18}N_2O_2$ $[M+H]^+$ 343.1447, Found 343.1444.

(*E*)-*N*-(Quinolin-8-yl)-5-(thiophen-3-yl)pent-4-enamide (5av)

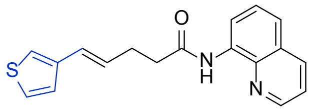

Following general procedure C, the reaction was carried out with alkene **1a** (0.2 mmol, 1.0 equiv.), thiophen-3-ylboronic acid **2v** (0.4 mmol, 2.0 equiv.), Ni(cod)$_2$ (0.02 mmol, 0.0055 g, 0.1 equiv.), CsOPiv (0.3 mmol, 0.0702 g, 1.5 equiv.), (*E*)-3-(3,4,5-trimethoxyphenyl)acrylaldehyde **A18** (0.2 mmol, 0.0444 g, 1.0 equiv.), and *n*-Bu$_3$P (0.04 mmol, 0.0081 g, 0.2 equiv.) in anhydrous *t*-AmylOH (1 mL) at 70 °C for 48 h. (*E*)-*N*-(Quinolin-8-yl)-5-(thiophen-3-yl)pent-4-enamide **(5av)** was isolated by column chromatography (PE/EtOAc = 10/1) as a white solid in 80 % yield. **M.P.**: 105–106 ºC. **^{1}H NMR (400 MHz, CDCl$_3$):** δ 9.90 (s, 1H), 8.84 (d, *J* = 8.1 Hz, 1H), 8.79 (dd, *J* = 4.1, 1.4 Hz, 1H), 8.18 (dd, *J* = 8.2, 1.4 Hz, 1H), 7.56 (dt, *J* = 8.2, 7.5 Hz, 2H), 7.47 (dd, *J* = 8.3, 4.2 Hz, 1H), 7.33–7.18 (m, 2H), 7.11 (d, *J* = 1.9 Hz, 1H), 6.58 (d, *J* = 15.8 Hz, 1H), 6.22 (dt, *J* = 15.8, 6.4 Hz, 1H), 2.92–2.57 (m, 4H). **^{13}C NMR (100 MHz, CDCl$_3$):** δ 170.96, 148.21, 140.12, 138.41, 136.46, 134.56, 128.66,

128.03, 127.51, 125.92, 125.59, 125.09, 121.70, 121.57, 121.15, 116.60, 37.86, 28.85. **HRMS (ESI)** m/z Calcd. for $C_{18}H_{16}N_2OS$ $[M+H]^+$ 309.1062, Found 309.1060.

(4*E*,6*E*)-7-Phenyl-*N*-(quinolin-8-yl)hepta-4,6-dienamide (5aw)

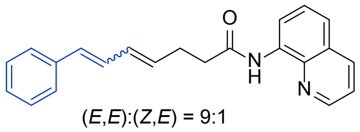

Following general procedure C, the reaction was carried out with alkene **1a** (0.2 mmol, 1.0 equiv.), (*E*)-styrylboronic acid **2w** (0.4 mmol, 2.0 equiv.), Ni(cod)$_2$ (0.02 mmol, 0.0055 g, 0.1 equiv.), CsOPiv (0.3 mmol, 0.0702 g, 1.5 equiv.), (*E*)-3-(3,4,5-trimethoxyphenyl)acrylaldehyde **A18** (0.2 mmol, 0.0444 g, 1.0 equiv.), and *n*-Bu$_3$P (0.04 mmol, 0.0081 g, 0.2 equiv.) in anhydrous *t*-AmylOH (1 mL) at 70 °C for 48 h. The (*E*,*E*)/(*E*,*Z*) ratio of the crude reaction mixture was 9:1, which was determined by ^{1}H NMR analysis. The resulting residue was purified by flash chromatography on silica gel (PE/EtOAc = 10/1) to afford the corresponding product in 74% yield. The ensuing analytical data correspond to the major isomer. **^{1}H NMR (400 MHz, CDCl$_3$):** δ 9.73 (s, 1H),), 8.79–8.58 (m, 2H), 8.04 (d, J = 8.1 Hz, 1H), 7.48–7.36 (m, 2H), 7.33 (dd, J = 8.2, 4.2 Hz, 1H), 7.25 (d, J = 7.5 Hz, 2H), 7.20–7.14 (m, 2H), 7.08 (t, J = 7.2 Hz, 1H), 6.64 (dd, J = 15.6, 10.4 Hz, 1H), 6.35 (d, J = 15.6 Hz, 1H), 6.23 (dd, J = 15.0, 10.4 Hz, 1H), 5.90–5.74 (m, 1H), 2.65–2.53 (m, 4H). **^{13}C NMR (100 MHz, CDCl$_3$):** δ 170.94, 148.26, 138.47, 137.58, 136.50, 134.59, 133.22, 131.94, 131.07, 129.11, 128.68, 128.07, 127.56, 127.38, 126.34, 121.72, 121.59, 116.63, 37.82, 28.81. **HRMS (ESI)** m/z Calcd. for $C_{22}H_{20}N_2O$ $[M+H]^+$ 329.1654, Found 329.1651.

(*E*)-5-Cyclohexenyl-*N*-(quinolin-8-yl)pent-4-enamide (5ax)

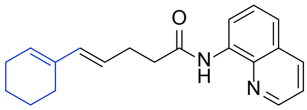

Following general procedure C, the reaction was carried out with alkene **1a** (0.2 mmol, 1.0 equiv.), cyclohexenylboronic acid **2x** (0.4 mmol, 2.0 equiv.), Ni(cod)$_2$ (0.02 mmol, 0.0055 g, 0.1 equiv.), CsOPiv (0.3 mmol, 0.0702 g, 1.5 equiv.), (*E*)-3-(3,4,5-trimethoxyphenyl)acrylaldehyde **A18** (0.2 mmol, 0.0444 g, 1.0 equiv.), and *n*-Bu$_3$P (0.04 mmol, 0.0081 g, 0.2 equiv.) in anhydrous *t*-AmylOH (1 mL) at 70 °C for 48 h. (*E*)-5-Cyclohexenyl-*N*-(quinolin-8-yl)pent-4-enamide **(5ax)** was

isolated by column chromatography (PE/EtOAc = 10/1) as a brown oil in 62 % yield. **^{1}H NMR (400 MHz, CDCl$_3$):** δ 9.82 (s, 1H), 8.94–8.66 (m, 2H), 8.15 (dd, J = 8.3, 1.5 Hz, 1H), 7.57–7.47 (m, 2H), 7.44 (dd, J = 8.3, 4.2 Hz, 1H), 6.16 (d, J = 15.6 Hz, 1H), 5.80–5.49 (m, 2H), 2.79–2.51 (m, 4H), 2.11–2.09 (m, 4H), 1.69–1.60 (m, 2H), 1.60–1.51 (m, 2H). **^{13}C NMR (100 MHz, CDCl$_3$):** δ 171.26, 148.21, 138.49, 136.46, 135.57, 134.96, 134.66, 128.22, 128.06, 127.55, 124.29, 121.68, 121.50, 116.60, 38.35, 28.89, 25.89, 24.69, 22.71, 22.63. **HRMS (ESI)** m/z Calcd. for $C_{20}H_{22}N_2O$ $[M+H]^+$ 307.1810, Found 307.1808.

(*E*)-2-Methyl-5-(naphthalen-2-yl)-*N*-(quinolin-8-yl)pent-4-enamide (5ba)

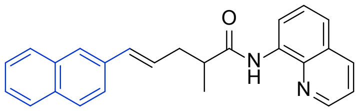

Following general procedure C, the reaction was carried out with alkene **1b** (0.2 mmol, 1.0 equiv.), naphthalen-2-ylboronic acid **2a** (0.4 mmol, 2.0 equiv.), Ni(cod)$_2$ (0.02 mmol, 0.0055 g, 0.1 equiv.), CsOPiv (0.3 mmol, 0.0702 g, 1.5 equiv.), (*E*)-3-(3,4,5-trimethoxyphenyl)acrylaldehyde **A18** (0.2 mmol, 0.0444 g, 1.0 equiv.), and *n*-Bu$_3$P (0.04 mmol, 0.0081 g, 0.2 equiv.) in anhydrous *t*-AmylOH (1 mL) at 70 °C for 48 h. (*E*)-2-Methyl-5-(naphthalen-2-yl)-*N*-(quinolin-8-yl)pent-4-enamide **(5ba)** was isolated by column chromatography (PE/EtOAc = 10/1) as a white solid in 86 % yield. **M.P.**: 64–65 °C. **^{1}H NMR (400 MHz, CDCl$_3$):** δ 9.85 (s, 1H), 8.74 (d, J = 7.5 Hz, 1H), 8.61 (d, J = 4.1 Hz, 1H), 7.99 (d, J = 8.2 Hz, 1H), 7.65–7.58 (m, 3H), 7.52 (s, 1H), 7.42 (dd, J = 8.0, 3.6 Hz, 2H), 7.37 (d, J = 8.2 Hz, 1H), 7.34–7.22 (m, 3H), 6.55 (d, J = 15.7 Hz, 1H), 6.38–6.14 (m, 1H), 2.84–2.56 (m, 2H), 2.56–2.29 (m, 1H), 1.32 (d, J = 6.2 Hz, 3H). **^{13}C NMR (100 MHz, CDCl$_3$):** δ 174.67, 148.23, 138.51, 136.41, 134.93, 134.52, 133.65, 132.83, 132.58, 128.07, 128.00, 127.95, 127.93, 127.67, 127.48, 126.20, 125.80, 125.66, 123.71, 121.63, 121.61, 116.67, 43.12, 37.92, 17.76. **HRMS (ESI)** m/z Calcd. for $C_{25}H_{22}N_2O$ $[M+H]^+$ 367.1810, Found 367.1808.

(*E*)-5-(Naphthalen-2-yl)-2-phenyl-*N*-(quinolin-8-yl)pent-4-enamide (5ca)

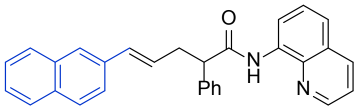

Following general procedure C, the reaction was carried out with alkene **1c** (0.2 mmol, 1.0 equiv.),

naphthalen-2-ylboronic acid **2a** (0.4 mmol, 2.0 equiv.), Ni(cod)$_2$ (0.02 mmol, 0.0055 g, 0.1 equiv.), CsOPiv (0.3 mmol, 0.0702 g, 1.5 equiv.), (*E*)-3-(3,4,5-trimethoxyphenyl)acrylaldehyde **A18** (0.2 mmol, 0.0444 g, 1.0 equiv.), and *n*-Bu$_3$P (0.04 mmol, 0.0081 g, 0.2 equiv.) in anhydrous *t*-AmylOH (1 mL) at 70 °C for 48 h. (*E*)-5-(Naphthalen-2-yl)-2-phenyl-*N*-(quinolin-8-yl)pent-4-enamide **(5ca)** was isolated by column chromatography (PE/EtOAc = 10/1) as a light yellow solid in 89 % yield. **M.P.**: 100–101 ºC. **^{1}H NMR (400 MHz, CDCl$_3$):** *δ* 10.00 (s, 1H), 8.85 (dd, *J* = 7.6, 0.9 Hz, 1H), 8.71 (dd, *J* = 4.2, 1.5 Hz, 1H), 8.07 (dd, *J* = 8.3, 1.5 Hz, 1H), 7.80–7.74 (m, 2H), 7.72 (d, *J* = 8.6 Hz, 1H), 7.65 (s, 1H), 7.58 (d, *J* = 7.3 Hz, 2H), 7.52 (d, *J* = 8.0 Hz, 2H), 7.48–7.39 (m, 5H), 7.39–7.29 (m, 2H), 6.69 (d, *J* = 15.8 Hz, 1H), 6.52–6.16 (m, 1H), 3.96 (t, *J* = 7.5 Hz, 1H), 3.44–3.14 (m, 1H), 3.02–2.65 (m, 1H). **^{13}C NMR (100 MHz, CDCl$_3$):** *δ* 171.46, 148.21, 139.42, 138.47, 136.29, 134.94, 134.51, 133.66, 132.84, 132.47, 129.07, 128.18, 128.07, 128.00, 127.95, 127.93, 127.67, 127.60, 127.37, 126.20, 125.83, 125.66, 123.71, 121.67, 121.60, 116.51, 55.14, 37.22. **HRMS (ESI)** m/z Calcd. for $C_{30}H_{24}N_2O$ $[M+H]^+$ 429.1967, Found 429.1964.

(*E*)-3-Methyl-5-(naphthalen-2-yl)-*N*-(quinolin-8-yl)pent-4-enamide (5da)

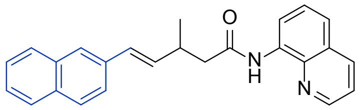

Following general procedure C, the reaction was carried out with alkene **1d** (0.2 mmol, 1.0 equiv.), naphthalen-2-ylboronic acid **2a** (0.4 mmol, 2.0 equiv.), Ni(cod)$_2$ (0.02 mmol, 0.0055 g, 0.1 equiv.), CsOPiv (0.3 mmol, 0.0702 g, 1.5 equiv.), (*E*)-3-(3,4,5-trimethoxyphenyl)acrylaldehyde **A18** (0.2 mmol, 0.0444 g, 1.0 equiv.), and *n*-Bu$_3$P (0.04 mmol, 0.0081 g, 0.2 equiv.) in anhydrous *t*-AmylOH (1 mL) at 70 °C for 48 h. (*E*)-3-Methyl-5-(naphthalen-2-yl)-N-(quinolin-8-yl)pent-4-enamide **(5da)** was isolated by column chromatography (PE/EtOAc = 10/1) as a brown solid in 89 % yield. **M.P.**: 100–101 ºC. **^{1}H NMR (400 MHz, CDCl$_3$):** *δ* 9.75 (s, 1H), 8.70 (d, *J* = 7.0 Hz, 1H), 8.53 (dd, *J* = 4.2, 1.5 Hz, 1H), 7.92 (dd, *J* = 8.3, 1.4 Hz, 1H), 7.66–7.56 (m, 3H), 7.51 (s, 1H), 7.43 (dd, *J* = 8.6, 1.4 Hz, 1H), 7.38 (t, *J* = 7.9 Hz, 1H), 7.33–7.23 (m, 3H), 7.20 (dd, *J* = 8.3, 4.2 Hz, 1H), 6.52 (d, *J* = 15.9 Hz, 1H), 6.27

(dd, J = 15.9, 7.4 Hz, 1H), 3.03–2.97 (m, 1H), 2.60 (dd, J = 14.3, 7.2 Hz, 1H), 2.49 (dd, J = 14.3, 7.1 Hz, 1H), 1.17 (J = 6.7 Hz, 3H). **^{13}C NMR (100 MHz, CDCl$_3$):** δ 170.55, 148.13, 138.34, 136.35, 134.97, 134.86, 134.45, 133.66, 132.80, 129.40, 128.04, 127.95, 127.91, 127.65, 127.40, 126.16, 125.85, 125.61, 123.72, 121.59, 121.56, 116.66, 45.68, 34.58, 20.36. **HRMS (ESI)** m/z Calcd. for $C_{25}H_{22}N_2O$ [M+H]$^+$ 367.1810, Found 367.1808.

(*E*)-5-(Naphthalen-2-yl)-3-phenyl-*N*-(quinolin-8-yl)pent-4-enamide (5ea)

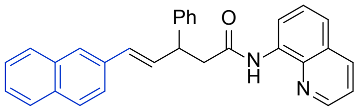

Following general procedure C, the reaction was carried out with alkene **1e** (0.2 mmol, 1.0 equiv.), naphthalen-2-ylboronic acid **2a** (0.4 mmol, 2.0 equiv.), Ni(cod)$_2$ (0.02 mmol, 0.0055 g, 0.1 equiv.), CsOPiv (0.3 mmol, 0.0702 g, 1.5 equiv.), (*E*)-3-(3,4,5-trimethoxyphenyl)acrylaldehyde **A18** (0.2 mmol, 0.0444 g, 1.0 equiv.), and *n*-Bu$_3$P (0.04 mmol, 0.0081 g, 0.2 equiv.) in anhydrous *t*-AmylOH (1 mL) at 70 °C for 48 h. (*E*)-5-(Naphthalen-2-yl)-3-phenyl-*N*-(quinolin-8-yl)pent-4-enamide **(5ea)** was isolated by column chromatography (PE/EtOAc = 10/1) as a white solid in 89 % yield. **M.P.**: 109–110 ºC. **^{1}H NMR (400 MHz, CDCl$_3$):** δ 9.90 (s, 1H), 8.82 (d, J = 7.4 Hz, 1H), 8.73 (d, J = 2.9 Hz, 1H), 8.11 (d, J = 8.2 Hz, 1H), 7.85–7.72 (m, 3H), 7.68 (s, 1H), 7.59 (d, J = 7.8 Hz, 1H), 7.54 (t, J = 7.9 Hz, 1H), 7.51–7.34 (m, 8H), 7.28 (t, J = 7.2 Hz, 1H), 6.78–6.60 (m, 2H), 4.40–4.35 (m, 1H), 3.27–3.05 (m, 2H). **^{13}C NMR (100 MHz, CDCl$_3$):** δ 169.66, 148.01, 143.01, 138.25, 136.46, 134.77, 134.40, 133.65, 132.92, 132.81, 130.72, 128.89, 128.07, 127.96, 127.84, 127.67, 127.47, 126.88, 126.21, 126.14, 125.73, 123.78, 121.59, 121.56, 116.80, 45.50, 44.58. **HRMS (ESI)** m/z Calcd. for $C_{30}H_{24}N_2O$ [M+H]$^+$ 429.1967, Found 429.1964.

(*E*)-3,3-Dimethyl-5-(naphthalen-2-yl)-*N*-(quinolin-8-yl)pent-4-enamide (5fa)

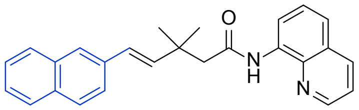

Following general procedure C, the reaction was carried out with alkene **1f** (0.2 mmol, 1.0 equiv.), naphthalen-2-ylboronic acid **2a** (0.4 mmol, 2.0 equiv.), Ni(cod)$_2$ (0.02 mmol, 0.0055 g, 0.1 equiv.), CsOPiv (0.3 mmol, 0.0702 g, 1.5 equiv.), (*E*)-3-(3,4,5-trimethoxyphenyl)acrylaldehyde **A18** (0.2 mmol, 0.0444 g, 1.0 equiv.),

and n-Bu$_3$P (0.04 mmol, 0.0081 g, 0.2 equiv.) in anhydrous t-AmylOH (1 mL) at 70 °C for 48 h. (E)-3,3-dimethyl-5-(naphthalen-2-yl)-N-(quinolin-8-yl) pent-4-enamide **(5fa)** was isolated by column chromatography (PE/EtOAc = 10/1) as a white solid in 87 % yield. **M.P.**: 71–72 ºC. **^{1}H NMR (400 MHz, CDCl$_3$):** δ 9.79 (s, 1H), 8.70 (dd, J = 7.6, 1.1 Hz, 1H), 8.17 (dd, J = 4.2, 1.6 Hz, 1H), 7.89 (dd, J = 8.3, 1.6 Hz, 1H), 7.68–7.63 (m, 1H), 7.60 (t, J = 8.1 Hz, 2H), 7.56 (s, 1H), 7.51 (dd, J = 8.6, 1.5 Hz, 1H), 7.38 (t, J = 8.0 Hz, 1H), 7.33–7.26 (m, 3H), 7.07 (dd, J = 8.3, 4.2 Hz, 1H), 6.50 (s, 2H), 2.58 (s, 2H), 1.29 (s, 6H). **^{13}C NMR (100 MHz, CDCl$_3$):** δ 170.08, 148.07, 139.15, 138.36, 136.15, 135.16, 134.55, 133.69, 132.84, 127.99, 127.96, 127.93, 127.90, 127.68, 127.37, 126.16, 126.03, 125.63, 124.00, 121.48, 121.45, 116.51, 51.92, 36.57, 27.73. **HRMS (ESI)** m/z Calcd. for $C_{26}H_{22}N_2O$ $[M+H]^+$ 381.1967, Found 381.1964.

(E)-2,2-Dimethyl-5-(naphthalen-2-yl)-N-(quinolin-8-yl)pent-4-enamide (5ga)

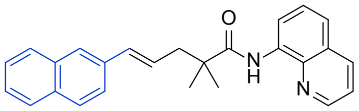

Following general procedure C, the reaction was carried out with alkene **1 g** (0.2 mmol, 1.0 equiv.), naphthalen-2-ylboronic acid **2a** (0.4 mmol, 2.0 equiv.), Ni(cod)$_2$ (0.02 mmol, 0.0055 g, 0.1 equiv.), CsOPiv (0.3 mmol, 0.0702 g, 1.5 equiv.), (E)-3-(3,4,5-trimethoxyphenyl)acrylaldehyde **A18** (0.2 mmol, 0.0444 g, 1.0 equiv.), and PMe$_3$ (0.04 mmol, 0.2 equiv.) in anhydrous t-AmylOH (1 mL) at 70 °C for 48 h. (E)-2,2-dimethyl-5-(naphthalen-2-yl)-N-(quinolin-8-yl)pent-4-enamide **(5ga)** was isolated by column chromatography (PE/EtOAc = 10/1) as a light brown oil in 36 % yield. **^{1}H NMR (400 MHz, CDCl$_3$):** δ 10.31 (s, 1H), 8.84 (d, J = 7.3 Hz, 1H), 8.74 (d, J = 3.3 Hz, 1H), 8.14 (d, J = 8.0 Hz, 1H), 7.76–7.68 (m, 3H), 7.63 (s, 1H), 7.59–7.47 (m, 3H), 7.45–7.35 (m, 3H), 6.66 (d, J = 15.6 Hz, 1H), 6.49–6.26 (m, 1H), 2.70 (d, J = 7.3 Hz, 2H), 1.50 (s, 6H). **^{13}C NMR (100 MHz, CDCl$_3$):** δ 176.26, 148.31, 138.87, 136.52, 135.03, 134.68, 133.70, 133.63, 132.89, 128.09, 127.98, 127.71, 127.61, 126.66, 126.23, 125.83, 125.70, 123.87, 121.64, 121.53, 116.65, 44.77, 44.39, 25.65. **HRMS (ESI)** m/z Calcd. for $C_{26}H_{24}N_2O$ $[M+H]^+$ 381.1967, Found 381.1965.

(*E*)-5-(Naphthalen-2-yl)-*N*-(quinolin-8-yl)hex-4-enamide (5ha)

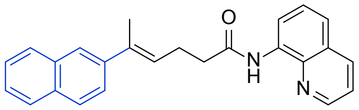

Following general procedure C, the reaction was carried out with alkene **1h** (0.2 mmol, 1.0 equiv.), naphthalen-2-ylboronic acid **2a** (0.4 mmol, 2.0 equiv.), Ni(cod)$_2$ (0.02 mmol, 0.0055 g, 0.1 equiv.), CsOPiv (0.3 mmol, 0.0702 g, 1.5 equiv.), (*E*)-3-(3,4,5-trimethoxyphenyl)acrylaldehyde **A18** (0.2 mmol, 0.0444 g, 1.0 equiv.), and *n*-Bu$_3$P (0.04 mmol, 0.0081 g, 0.2 equiv.) in anhydrous *t*-AmylOH (1 mL) at 70 °C for 48 h. (*E*)-5-(Naphthalen-2-yl)-*N*-(quinolin-8-yl)hex-4-enamide **(5ha)** was isolated by column chromatography (PE/EtOAc = 10/1) as a light yellow oil in 91 % yield. **^{1}H NMR (400 MHz, CDCl$_3$):** δ 9.89 (s, 1H), 8.82 (d, *J* = 7.4 Hz, 1H), 8.72 (d, *J* = 3.9 Hz, 1H), 8.14 (d, *J* = 8.2 Hz, 1H), 7.80–7.74 (m, 4H), 7.61–7.48 (m, 3H), 7.48–7.37 (m, 3H), 6.08–6.00 (m, 1H), 2.96–2.62 (m, 4H), 2.21 (s, 3H). **^{13}C NMR (100 MHz, CDCl$_3$):** δ 171.28, 148.24, 140.91, 138.47, 136.54, 136.46, 134.66, 133.55, 132.58, 128.16, 128.06, 127.69, 127.58, 126.94, 126.12, 125.61, 124.55, 124.27, 121.70, 121.57, 116.61, 38.09, 25.18, 16.14. **HRMS (ESI)** m/z Calcd. for $C_{25}H_{22}N_2O$ $[M+H]^+$ 367.1810, Found 367.1808.

5-(Naphthalen-2-yl)-*N*-(quinolin-8-yl)hex-4-enamide (5ia)

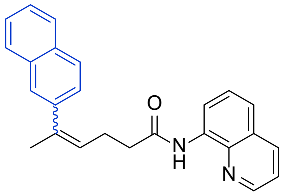

Following general procedure C, the reaction was carried out with (*Z*)-alkene **1i** containing 9% E-isomer (0.2 mmol, 1.0 equiv.), naphthalen-2-ylboronic acid **2a** (0.4 mmol, 2.0 equiv.), Ni(cod)$_2$ (0.02 mmol, 0.0055 g, 0.1 equiv.), CsOPiv (0.3 mmol, 0.0702 g, 1.5 equiv.), (*E*)-3-(3,4,5-trimethoxyphenyl)acrylaldehyde **A18** (0.2 mmol, 0.0444 g, 1.0 equiv.), and *n*-Bu$_3$P (0.04 mmol, 0.0081 g, 0.2 equiv.) in anhydrous *t*-AmylOH (1 mL) at 70 °C for 48 h. The *Z*:*E* ratio of the crude reaction mixture was 91:9, which was determined by ^{1}H NMR analysis. The resulting residue was purified by flash chromatography on silica gel (PE/EtOAc = 10/1) to afford **5ia** as mixture of *E*- and *Z*-isomers (*Z*/*E* = 91:9) in 85% yield. A small amount of the mixture was further purified by column chromatography (PE/EtOAc = 10/1) to obtain analytically pure (*Z*)-5-(naphthalen-2-yl)-*N*-(quinolin-8-yl)hex-4-enamide as a light yellow oil. NMR data of the Z isomer: **^{1}H NMR (400 MHz, CDCl$_3$):** δ 9.77 (s, 1H),

8.78 (d, J = 7.4 Hz, 1H), 8.71 (d, J = 4.1 Hz, 1H), 8.13 (d, J = 8.2 Hz, 1H), 7.84–7.68 (m, 3H), 7.65 (s, 1H), 7.55–7.40 (m, 5H), 7.35 (d, J = 8.4 Hz, 1H), 5.78–5.56 (m, 1H), 2.67–2.49 (m, 4H), 2.14 (s, 3H). **^{13}C NMR (100 MHz, $CDCl_3$):** δ 171.22, 148.13, 139.29, 138.36, 138.10, 136.38, 134.57, 133.37, 132.37, 127.98, 127.96, 127.81, 127.68, 127.50, 126.55, 126.48, 126.05, 125.93, 125.69, 121.64, 121.45, 116.48, 38.51, 25.81, 25.47. **HRMS (ESI)** m/z Calcd. for $C_{25}H_{22}N_2O$ $[M+H]^+$ 367.1810, Found 367.1806.

(*E*)-5-(Naphthalen-2-yl)-*N*-(quinolin-8-yl)hept-4-enamide (5ja)

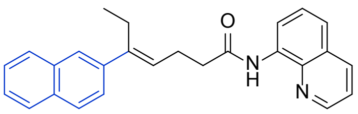

Following general procedure C, the reaction was carried out with alkene **1j** (0.2 mmol, 1.0 equiv.), naphthalen-2-ylboronic acid **2a** (0.4 mmol, 2.0 equiv.), Ni(cod)$_2$ (0.02 mmol, 0.0055 g, 0.1 equiv.), CsOPiv (0.3 mmol, 0.0702 g, 1.5 equiv.), (*E*)-3-(3,4,5-trimethoxyphenyl)acrylaldehyde **A18** (0.2 mmol, 0.0444 g, 1.0 equiv.), and *n*-Bu$_3$P (0.04 mmol, 0.0081 g, 0.2 equiv.) in anhydrous *t*-AmylOH (1 mL) at 70 °C for 48 h. (*E*)-5-(Naphthalen-2-yl)-*N*-(quinolin-8-yl)hept-4-enamide **(5ja)** was isolated by column chromatography (PE/EtOAc = 10/1) as a light yellow oil in 92 % yield. **^{1}H NMR (400 MHz, $CDCl_3$):** δ 9.91 (s, 1H), 8.85 (d, J = 7.4 Hz, 1H), 8.75 (d, J = 2.9 Hz, 1H), 8.14 (d, J = 7.8 Hz, 1H), 7.81–7.75 (m, 4H), 7.59–7.48 (m, 3H), 7.48–7.36 (m, 3H), 5.89 (t, J = 6.5 Hz, 1H), 2.82–2.75 (m, 4H), 2.70 (q, J = 7.5 Hz, 2H), 1.05 (t, J = 7.5 Hz, 3H). **^{13}C NMR (100 MHz, $CDCl_3$):** δ 171.30, 148.22, 143.43, 140.07, 138.43, 136.44, 134.58, 133.56, 132.57, 128.07, 128.03, 127.69, 127.56, 127.52, 126.53, 126.04, 125.54, 125.22, 124.87, 121.67, 121.58, 116.62, 38.37, 24.81, 23.14, 13.77. **HRMS (ESI)** m/z Calcd. for $C_{26}H_{24}N_2O$ $[M+H]^+$ 381.1967, Found 381.1964.

(*E*)-5-(Naphthalen-2-yl)-*N*-(quinolin-8-yl)dec-4-enamide (5ka)

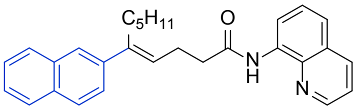

Following general procedure C, the reaction was carried out with alkene **1k** (0.2 mmol, 1.0 equiv.), naphthalen-2-ylboronic acid **2a** (0.4 mmol, 2.0 equiv.), Ni(cod)$_2$ (0.02 mmol, 0.0055 g, 0.1 equiv.), CsOPiv (0.3 mmol, 0.0702 g, 1.5 equiv.),

(*E*)-3-(3,4,5-trimethoxyphenyl)acrylaldehyde **A18** (0.2 mmol, 0.0444 g, 1.0 equiv.), and *n*-Bu_3P (0.04 mmol, 0.0081 g, 0.2 equiv.) in anhydrous *t*-AmylOH (1 mL) at 70 °C for 48 h. (*E*)-5-(Naphthalen-2-yl)-*N*-(quinolin-8-yl)dec-4-enamide **(5ka)** was isolated by column chromatography (PE/EtOAc = 10/1) as a light yellow oil in 89 % yield. **^{1}H NMR (400 MHz, $CDCl_3$):** *δ* 9.92 (s, 1H), 8.86 (d, *J* = 7.2 Hz, 1H), 8.76 (dd, *J* = 4.1, 1.3 Hz, 1H), 8.14 (dd, *J* = 8.2, 1.3 Hz, 1H), 7.82–7.75 (m, 4H), 7.60–7.48 (m, 3H), 7.48–7.37 (m, 3H), 5.89 (t, *J* = 6.7 Hz, 1H), 2.88–2.72 (m, 4H), 2.72–2.59 (m, 2H), 1.53–1.36 (m, 2H), 1.32–1.22 (m, 4H), 0.85 (t, *J* = 7.0 Hz, 3H). **^{13}C NMR (100 MHz, $CDCl_3$):** *δ* 171.31, 148.20, 142.16, 140.48, 138.42, 136.43, 134.57, 133.55, 132.55, 128.07, 128.03, 127.67, 127.57, 127.51, 127.14, 126.02, 125.52, 125.29, 124.90, 121.66, 121.57, 116.62, 38.40, 31.92, 29.97, 28.56, 25.00, 22.62, 14.15. **HRMS (ESI)** m/z Calcd. for $C_{29}H_{30}N_2O$ $[M+H]^+$ 423.2436, Found 423.2433.

(*E*)-5-(Naphthalen-2-yl)-5-phenyl-N-(quinolin-8-yl)pent-4-enamide (5la)

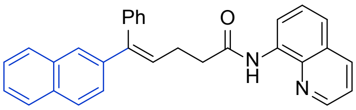

Following general procedure C, the reaction was carried out with alkene **1l** (0.2 mmol, 1.0 equiv.), naphthalen-2-ylboronic acid **2a** (0.4 mmol, 2.0 equiv.), $Ni(cod)_2$ (0.04 mmol, 0.011 g, 0.2 equiv.), CsOPiv (0.3 mmol, 0.0702 g 1.5 equiv.), (*E*)-3-(3,4,5-trimethoxyphenyl)acrylaldehyde **A18** (0.2 mmol, 0.0444 g, 1.0 equiv.), and *n*-Bu_3P (0.08 mmol, 0.0162 g, 0.4 equiv.) in anhydrous *t*-AmylOH (1 mL) at 120 °C for 72 h. (*E*)-5-(Naphthalen-2-yl)-5-phenyl-*N*-(quinolin-8-yl) pent-4-enamide (**5la**) was isolated by column chromatography (PE/EtOAc = 10/1) as a white solid in 32 % yield. **M.P.**: 53–54 ºC. **^{1}H NMR (400 MHz, $CDCl_3$):** *δ* 9.75 (s, 1H), 8.69 (d, *J* = 7.4 Hz, 1H), 8.62 (dd, *J* = 4.2, 1.5 Hz, 1H), 8.03 (dd, *J* = 8.3, 1.4 Hz, 1H), 7.69–7.63 (m, 1H), 7.61 (d, *J* = 8.6 Hz, 1H), 7.59–7.54 (m, 1H), 7.46 (s, 1H), 7.42 (d, *J* = 7.6 Hz, 1H), 7.37 (ddd, *J* = 8.6, 4.4, 1.4 Hz, 2H), 7.29 (ddd, *J* = 12.7, 9.2, 6.3 Hz, 5H), 7.25–7.19 (m, 1H), 7.17–7.12 (m, 2H), 6.24 (t, *J* = 6.9 Hz, 1H), 2.71–2.49 (m, 4H). **^{13}C NMR (100 MHz, $CDCl_3$):** *δ* 171.01, 148.11, 143.24, 139.89, 139.84, 138.27, 136.59, 134.51, 133.39, 132.68, 130.02, 128.47, 128.25, 128.21, 128.04, 127.66, 127.57, 127.31, 126.54, 126.11, 125.82, 125.53, 121.66, 121.56, 116.71, 38.29, 26.13.

HRMS (ESI) m/z Calcd. for $C_{30}H_{24}N_2O$ $[M+H]^+$ 429.1967, Found 429.1964.

(*E*)-3-Methyl-5-(naphthalen-2-yl)-*N*-(quinolin-8-yl)hex-4-enamide (5ma)

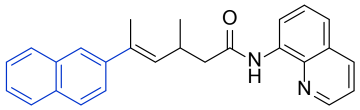

Following general procedure C, the reaction was carried out with alkene **1m** (0.2 mmol, 1.0 equiv.), naphthalen-2-ylboronic acid **2a** (0.4 mmol, 2.0 equiv.), Ni(cod)$_2$ (0.02 mmol, 0.0055 g, 0.1 equiv.), CsOPiv (0.3 mmol, 0.0702 g, 1.5 equiv.), (*E*)-3-(3,4,5-trimethoxyphenyl)acrylaldehyde **A18** (0.2 mmol, 0.0444 g, 1.0 equiv.), and *n*-Bu$_3$P (0.04 mmol, 0.0081 g, 0.2 equiv.) in anhydrous *t*-AmylOH (1 mL) at 70 °C for 48 h. (*E*)-3-Methyl-5-(naphthalen-2-yl)-*N*-(quinolin-8-yl) hex-4-enamide **(5ma)** was isolated by column chromatography (PE/EtOAc = 10/1) as a brown oil in 88 % yield. **^{1}H NMR (400 MHz, CDCl$_3$):** δ 9.91 (s, 1H), 8.83 (d, J = 13.2 Hz, 1H), 8.63 (d, J = 5.5 Hz, 1H), 8.10 (dd, J = 8.2, 1.2 Hz, 1H), 7.82–7.76 (m, 1H), 7.73 (d, J = 8.1 Hz, 3H), 7.60–7.51 (m, 2H), 7.48 (d, J = 8.2 Hz, 1H), 7.46–7.39 (m, 2H), 7.35 (dd, J = 8.2, 4.2 Hz, 1H), 5.88 (d, J = 9.4 Hz, 1H), 3.42–3.32 (m, 1H), 2.68 (d, J = 7.0 Hz, 2H), 2.20 (s, 3H), 1.28 (d, J = 6.7 Hz, 3H). **^{13}C NMR (100 MHz, CDCl$_3$):** δ 170.88, 148.16, 140.95, 138.40, 136.35, 135.33, 134.59, 133.45, 133.19, 132.52, 128.09, 127.98, 127.55, 127.53, 127.48, 126.04, 125.55, 124.62, 124.33, 121.59, 116.60, 46.26, 31.33, 21.27, 16.26. **HRMS (ESI)** m/z Calcd. for $C_{26}H_{24}N_2O$ $[M+H]^+$ 381.1967, Found 381.1964.

(*E*)-5-(Naphthalen-2-yl)-3-phenyl-*N*-(quinolin-8-yl)hex-4-enamide (5na)

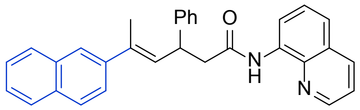

Following general procedure C, the reaction was carried out with alkene **1n** (0.2 mmol, 1.0 equiv.), naphthalen-2-ylboronic acid **2a** (0.4 mmol, 2.0 equiv.), Ni(cod)$_2$ (0.02 mmol, 0.0055 g, 0.1 equiv.), CsOPiv (0.3 mmol, 0.0702 g, 1.5 equiv.), (*E*)-3-(3,4,5-trimethoxyphenyl)acrylaldehyde **A18** (0.2 mmol, 0.0444 g, 1.0 equiv.), and *n*-Bu$_3$P (0.04 mmol, 0.0081 g, 0.2 equiv.) in anhydrous *t*-AmylOH (1 mL) at 70 °C for 48 h. (*E*)-5-(Naphthalen-2-yl)-3-phenyl-*N*-(quinolin-8-yl)hex-4-enamide **(5na)** was isolated by column chromatography (PE/EtOAc = 10/1) as a white solid in 85 % yield. **M.P.**: 67–68 ºC. **^{1}H NMR (400 MHz, CDCl$_3$):** δ 9.89 (s, 1H), 8.83 (d, J

= 6.8 Hz, 1H), 8.67 (dd, J = 4.1, 1.4 Hz, 1H), 8.11 (dd, J = 8.2, 1.3 Hz, 1H), 7.82–7.76 (m, 1H), 7.72 (d, J = 6.8 Hz, 3H), 7.59–7.51 (m, 2H), 7.51–7.41 (m, 5H), 7.41–7.34 (m, 3H), 7.30–7.22 (m, 1H), 6.24 (d, J = 10.1 Hz, 1H), 4.57 (dd, J = 16.5, 7.7 Hz, 1H), 3.23–2.99 (m, 2H), 2.27 (s, 3H). **^{13}C NMR (100 MHz, $CDCl_3$):** δ 169.92, 148.11, 144.05, 140.86, 138.35, 136.66, 136.32, 134.51, 133.39, 132.56, 130.77, 128.89, 128.09, 127.94, 127.56, 127.52, 127.46, 126.63, 126.06, 125.62, 124.66, 124.50, 121.58, 116.60, 46.08, 41.91, 16.61. **HRMS (ESI)** m/z Calcd. for $C_{31}H_{26}N_2O$ $[M+H]^+$ 443.2123, Found 443.2120.

4-Methyl-5-(naphthalen-2-yl)-*N*-(quinolin-8-yl)pent-4-enamide (5oa)

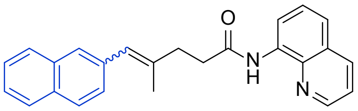

Following general procedure C, the reaction was carried out with alkene **1o** (0.2 mmol, 1.0 equiv.), naphthalen-2-ylboronic acid **2a** (0.4 mmol, 2.0 equiv.), $Ni(cod)_2$ (0.02 mmol, 0.0055 g, 0.1 equiv.), CsOPiv (0.3 mmol, 0.0702 g, 1.5 equiv.), (*E*)-3-(3,4,5-trimethoxyphenyl)acrylaldehyde **A18** (0.2 mmol, 0.0444 g, 1.0 equiv.), and *n*-Bu_3P (0.04 mmol, 0.0081 g, 0.2 equiv.) in anhydrous *t*-AmylOH (1 mL) at 70 °C for 48 h. The *E*:*Z* ratio of the crude reaction mixture was 63:37, which was determined by ^{1}H NMR analysis. The resulting residue was purified by flash chromatography on silica gel (PE/EtOAc = 10/1) to afford **5oa** as a mixture of *E*- and *Z*-isomers in 84% yield. A small amount of the mixture was further purified by column chromatography (PE/EtOAc = 10/1) to obtain analytically pure samples of both isomers. Pure (*E*)-4-methyl-5-(naphthalen-2-yl)-*N*-(quinolin-8-yl)pent-4-enamide was obtained as a white solid, **M.P.**: 80–81 ºC and the pure (*Z*)-4-methyl-5-(naphthalen-2-yl)-*N*-(quinolin-8-yl)pent-4-enamide was obtained as a light brown oil. NMR data of the *E* isomer: **^{1}H NMR (400 MHz, $CDCl_3$):** δ 9.93 (s, 1H), 8.84 (d, J = 7.4 Hz, 1H), 8.77 (dd, J = 4.1, 1.4 Hz, 1H), 8.15 (d, J = 7.6 Hz, 1H), 7.78 (dd, J = 13.5, 8.5 Hz, 3H), 7.66 (s, 1H), 7.59–7.48 (m, 2H), 7.44 (dt, J = 10.4, 4.0 Hz, 3H), 7.37 (d, J = 8.3 Hz, 1H), 6.57 (s, 1H), 2.95–2.81 (m, 2H), 2.81–2.70 (m, 2H), 2.04 (s, 3H). **^{13}C NMR (100 MHz, $CDCl_3$):** δ 171.26, 148.23, 138.44, 137.88, 136.49,

135.83, 134.62, 133.42, 132.01, 128.05, 127.91, 127.65, 127.63, 127.56, 127.53, 127.44, 126.08, 126.04, 125.59, 121.69, 121.58, 116.64, 37.02, 36.37, 18.15. **HRMS (ESI)** m/z Calcd. for $C_{25}H_{22}N_2O$ $[M+H]^+$ 367.1810, Found 367.1808.

NMR data of the *Z* isomer: **^{1}H NMR (400 MHz, CDCl$_3$):** *δ* 9.81 (s, 1H), 8.77 (d, *J* = 7.2 Hz, 1H), 8.68 (dd, *J* = 4.1, 1.3 Hz, 1H), 8.13 (dd, J = 8.2, 1.3 Hz, 1H), 7.82–7.71 (m, 3H), 7.69 (s, 1H), 7.51 (dt, *J* = 8.2, 7.5 Hz, 2H), 7.45–7.33 (m, 4H), 6.53 (s, 1H), 2.98–2.83 (m, 2H), 2.77 (dd, *J* = 9.2, 6.0 Hz, 2H), 2.04 (s, 3H). **^{13}C NMR (100 MHz, CDCl$_3$):** *δ* 171.09, 148.15, 138.36, 137.93, 136.43, 135.64, 134.56, 133.54, 132.08, 128.03, 128.01, 127.81, 127.62, 127.53, 127.34, 127.17, 127.08, 126.01, 125.56, 121.68, 121.53, 116.54, 36.76, 28.78, 24.18. **HRMS (ESI)** m/z Calcd. for $C_{25}H_{22}N_2O$ $[M+H]^+$ 367.1810, Found 367.1807.

5-(Naphthalen-2-yl)-2-phenyl-*N*-(quinolin-8-yl)hex-4-enamide (5pa)

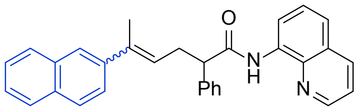

Following general procedure C, the reaction was carried out with alkene **1p** (0.2 mmol, 1.0 equiv.), naphthalen-2-ylboronic acid **2a** (0.4 mmol, 2.0 equiv.), Ni(cod)$_2$ (0.02 mmol, 0.0055 g, 0.1 equiv.), CsOPiv (0.3 mmol, 0.0702 g, 1.5 equiv.), (*E*)-3-(3,4,5-trimethoxyphenyl)acrylaldehyde **A18** (0.2 mmol, 0.0444 g, 1.0 equiv.), and *n*-Bu$_3$P (0.04 mmol, 0.0081 g, 0.2 equiv.) in anhydrous *t*-AmylOH (1 mL) at 120 °C for 48 h. The *E*:*Z* ratio of the crude reaction mixture was 84:16, which was determined by ^{1}H NMR analysis. The resulting residue was purified by flash chromatography on silica gel (PE/EtOAc = 10/1) to afford **5pa** as a mixture of *E*- and *Z*-isomers in 78% yield. A small amount of the mixture was further purified by column chromatography (PE/EtOAc = 10/1) to yield analytically pure (*E*)-5-(naphthalen-2-yl)-2-phenyl-*N*-(quinolin-8-yl)hex-4-enamide as a white solid, **M.P.**: 122–123 °C. NMR data of the *E* isomer: **^{1}H NMR (400 MHz, CDCl$_3$):** *δ* 9.98 (s, 1H), 8.83 (d, *J* = 7.5 Hz, 1H), 8.70 (d, *J* = 4.0 Hz, 1H), 8.10 (d, *J* = 7.9 Hz, 1H), 7.81–7.68 (m, 4H), 7.61–7.35 (m, 10H), 7.32 (t, *J* = 7.3 Hz, 1H), 5.97 (t, *J* = 7.1 Hz, 1H), 3.94 (t, *J* = 7.5 Hz, 1H), 3.33–3.26 (m, 1H), 2.98–2.90 (m, 1H), 2.16 (s, 3H). **^{13}C NMR (100 MHz, CDCl$_3$):** *δ* 171.73, 148.19, 140.98, 139.64, 138.46, 136.96, 136.39,

134.58, 133.47, 132.52, 129.02, 128.27, 128.12, 127.98, 127.61, 127.56, 127.54, 127.46, 126.06, 125.81, 125.56, 124.56, 124.25, 121.64, 121.62, 116.56, 54.92, 33.13, 16.24. **HRMS (ESI)** m/z Calcd. for $C_{31}H_{26}N_2O$ $[M+H]^+$ 443.2123, Found 443.2121.

5-(Naphthalen-2-yl)-*N*-(quinolin-8-yl)hex-4-enamide (5qa)

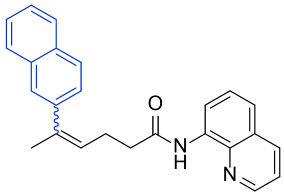

Following general procedure D, the reaction was carried out with alkene **1q** (0.3 mmol, 1.5 equiv.), naphthalen-2-ylboronic acid (0.2 mmol, 1.0 equiv.), Ni(cod)$_2$ (0.02 mmol, 0.0055 g, 0.1 equiv.), CsOPiv (0.3 mmol, 0.0702 g, 1.5 equiv.), and PPhMe$_2$ (0.04 mmol, 0.0055 g, 0.2 equiv.) in anhydrous *t*-AmylOH (1 mL) at 70 °C for 48 h. The *Z*:*E* ratio of the crude reaction mixture was 92:8, which was determined by ^{1}H NMR analysis. The resulting residue was purified by flash chromatography on silica gel (PE/EtOAc = 10/1) to afford **5qa** as a mixture of *E*- and *Z*-isomers in 83% yield. A small amount of the mixgture was further purified by column chromatography (PE/EtOAc = 10/1) to obtain analytically pure (*Z*)-5-(naphthalen-2-yl)-*N*-(quinolin-8-yl)hex-4-enamide as a colorless oil. NMR data of the *Z* isomer: **^{1}H NMR (400 MHz, CDCl$_3$):** *δ* 9.77 (s, 1H), 8.78 (d, *J* = 7.3 Hz, 1H), 8.71 (d, *J* = 5.1 Hz, 1H), 8.13 (d, *J* = 9.2 Hz, 1H), 7.85–7.72 (m, 3H), 7.65 (s, 1H), 7.55–7.40 (m, 5H), 7.35 (d, *J* = 9.3 Hz, 1H), 5.78–5.56 (m, 1H), 2.67–2.49 (m, 4H), 2.14 (s, 3H). **^{13}C NMR (100 MHz, CDCl$_3$):** *δ* 171.23, 148.14, 139.31, 138.37, 138.12, 136.39, 134.58, 133.38, 132.38, 127.97, 127.82, 127.68, 127.51, 126.55, 126.49, 126.06, 125.93, 125.70, 121.65, 121.46, 116.49, 38.53, 25.81, 25.48. **HRMS (ESI)** m/z Calcd. for $C_{25}H_{22}N_2O$ $[M+H]^+$ 367.1810, Found 367.1807.

Methyl 4-(6-oxo-6-(quinolin-8-ylamino)hex-2-en-2-yl)benzoate (5qb)

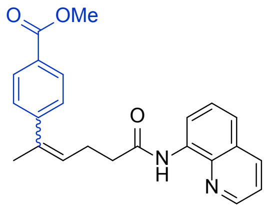

Following general procedure D, the reaction was carried out with alkene **1q** (0.3 mmol, 1.5 equiv.), 4-acetylphenylboronic acid (0.2 mmol, 1.0 equiv.), Ni(cod)$_2$ (0.02 mmol, 0.0055 g, 0.1 equiv.), CsOPiv (0.3 mmol, 0.0702 g, 1.5 equiv.), and PPhMe$_2$ (0.04 mmol, 0.0055 g, 0.2 equiv.) in anhydrous *t*-AmylOH (1 mL) at 70 °C for 48 h. The *Z*:*E* ratio of the crude reaction mixture was 81:19, which was determined by ^{1}H

NMR analysis. The resulting residue was purified by flash chromatography on silica gel (PE/EtOAc = 7/1) to afford **5qb** as a mixture of *E*- and *Z*-isomers in 80% yield. A small amount of the mixture was further purified by column chromatography (PE/EtOAc = 7/1) to yield analytically pure (*Z*)-methyl 4-(6-oxo-6-(quinolin-8-ylamino)hex-2-en-2-yl)benzoate as a light yellow oil. NMR data of the *Z* isomer: **^{1}H NMR (400 MHz, $CDCl_3$):** δ 9.76 (s, 1H), 8.77 (d, J = 6.6 Hz, 2H), 8.16 (d, J = 8.0 Hz, 1H), 7.97 (d, J = 8.1 Hz, 2H), 7.64–7.36 (m, 3H), 7.27 (d, J = 8.1 Hz, 2H), 5.65 (t, J = 6.9 Hz, 1H), 3.91 (s, 3H), 2.59 (t, J = 7.0 Hz, 2H), 2.51 (t, J = 7.1 Hz, 2H), 2.05 (s, 3H). **^{13}C NMR (100 MHz, $CDCl_3$):** δ 170.97, 167.02, 148.15, 146.75, 138.32, 137.31, 136.49, 134.51, 129.91, 129.64, 128.50, 128.02, 127.52, 126.65, 121.65, 121.51, 116.56, 52.10, 38.28, 25.39, 25.34. **HRMS (ESI)** m/z Calcd. for $C_{23}H_{22}N_2O_3$ $[M+H]^+$ 375.1709, Found 375.1706.

N-(Quinolin-8-yl)-5-(4-(trifluoromethyl)phenyl)hex-4-enamide (5qc)

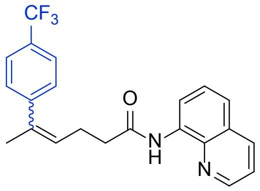

Following general procedure D, the reaction was carried out with alkene **1q** (0.3 mmol, 1.5 equiv.), 4-(trifluoromethyl)phenylboronic acid (0.2 mmol, 1.0 equiv.), $Ni(cod)_2$ (0.02 mmol, 0.0055 g, 0.1 equiv.), CsOPiv (0.3 mmol, 0.0702 g, 1.5 equiv.), and $PPhMe_2$ (0.04 mmol, 0.0055 g, 0.2 equiv.) in anhydrous *t*-AmylOH (1 mL) at 70 °C for 48 h. The *Z*:*E* ratio of the crude reaction mixture was 90:10, which was determined by ^{1}H NMR analysis. The resulting residue was purified by flash chromatography on silica gel (PE/EtOAc = 10/1) to afford **5qc** as a mixture of *E*- and *Z*-isomers in 75% yield. A small amount of the mixture was further purified by column chromatography (PE/EtOAc = 10/1) to obtain analytically pure (*Z*)-*N*-(quinolin-8-yl)-5-(4-(trifluoromethyl)phenyl)hex-4-enamide as a light yellow oil. NMR data of the Z isomer: **^{1}H NMR (400 MHz, $CDCl_3$):** δ 9.76 (s, 1H), 8.76 (dd, J = 4.3, 1.4 Hz, 2H), 8.15 (dd, J = 8.3, 1.5 Hz, 1H), 7.59–7.46 (m, 4H), 7.44 (dd, J = 8.3, 4.2 Hz, 1H), 7.29 (d, J = 8.0 Hz, 2H), 5.64 (td, J = 7.2, 1.2 Hz, 1H), 2.59 (t, J = 7.5 Hz, 2H), 2.50–2.45 (m, 2H), 2.03 (d, J = 1.0 Hz, 3H). **^{13}C NMR (100 MHz, $CDCl_3$):** $\delta\delta$ 170.97, 148.21, 145.58, 138.33, 136.95, 136.50, 134.49, 128.85 (q, J =

32.3 Hz), 128.35, 128.03, 127.52, 126.79, 125.26 (q, J = 3.8 Hz), 124.33 (q, J = 272.1 Hz), 121.73, 121.57, 116.51, 38.23, 25.48, 25.33.. **^{19}F NMR (376 MHz, $CDCl_3$):** δ -67.10. **HRMS (ESI)** m/z Calcd. for $C_{22}H_{19}F_3N_2O$ $[M+H]^+$ 385.1528, Found 385.1525.

5-(Benzofuran-5-yl)-*N*-(quinolin-8-yl)hex-4-enamide (5qd)

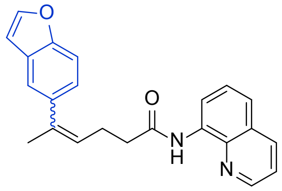

Following general procedure D, the reaction was carried out with alkene **1q** (0.3 mmol, 1.5 equiv.), benzofuran-5-ylboronic acid (0.2 mmol, 1.0 equiv.), $Ni(cod)_2$ (0.02 mmol, 0.0055 g, 0.1 equiv.), CsOPiv (0.3 mmol, 0.0702 g, 1.5 equiv.), and $PPhMe_2$ (0.04 mmol, 0.0055 g, 0.2 equiv.) in anhydrous *t*-AmylOH (1 mL) at 70 °C for 48 h. The *Z*:*E* ratio of the crude reaction mixture was 92:8, which was determined by ^{1}H NMR analysis. The resulting residue was purified by flash chromatography on silica gel (PE/EtOAc = 10/1) to afford **5qd** as a mixture of *E*- and *Z*-isomers in 40 % yield. A small amount of the mixture was further purified by column chromatography (PE/EtOAc = 10/1) to obtain analytically pure (*Z*)-5-(benzofuran-5-yl)-*N*-(quinolin-8-yl)hex-4-enamide as a light yellow oil. **^{1}H NMR (400 MHz, $CDCl_3$):** δ 9.77 (s, 1H), 8.94–8.49 (m, 2H), 8.16 (dd, J = 8.3, 1.4 Hz, 1H), 7.58 (d, J = 2.2 Hz, 1H), 7.56–7.47 (m, 2H), 7.47–7.36 (m, 3H), 7.12 (dd, J = 8.4, 1.7 Hz, 1H), 6.74–6.63 (m, 1H), 5.61 (t, J = 6.9 Hz, 1H), 2.70–2.40 (m, 4H), 2.08 (d, J = 1.0 Hz, 3H). **^{13}C NMR (100 MHz, $CDCl_3$):** δ 171.36, 153.97, 148.04, 145.26, 138.40, 136.66, 136.59, 134.57, 128.08, 127.63, 127.43, 125.41, 124.54, 121.65, 121.49, 120.38, 116.74, 111.10, 106.72, 38.56, 26.36, 25.47. **HRMS (ESI)** m/z Calcd. for $C_{23}H_{20}N_2O_2$ $[M+H]^+$ 357.1603, Found 357.1600.

(*E*)-5-(2-Methoxy-4-methylphenyl)-*N*-(quinolin-8-yl)hex-4-enamide (5hz)

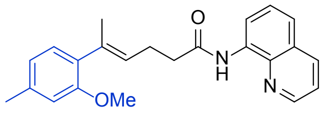

Following general procedure C, the reaction was carried out with alkene **1h** (0.2 mmol, 1.0 equiv.), 2-methoxy-4-methylphenylboronic acid **2z** (0.4 mmol, 2.0 equiv.), $Ni(cod)_2$ (0.04 mmol, 0.011 g, 0.2 equiv.), CsOPiv (0.3 mmol, 0.0702 g, 1.5 equiv.),

(*E*)-3-(3,4,5-trimethoxyphenyl)acrylaldehyde **A18** (0.2 mmol, 0.0444 g, 1.0 equiv.), and PMe_3 (0.08 mmol, 0.4 equiv.) in anhydrous *t*-AmylOH (1 mL) at 120 °C for 48 h. (*E*)-5-(2-Methoxy-4-methylphenyl)-*N*-(quinolin-8-yl)hex-4-enamide (**5hz**) was isolated by column chromatography (PE / EtOAc = 10/1) as a light yellow oil in 88 % yield. **^{1}H NMR (400 MHz, $CDCl_3$):** *δ* 9.88 (s, 1H), 8.83–8.78 (m, 2H), 8.15 (d, *J* = 7.2 Hz, 1H), 7.57–7.47 (m, 2H), 7.44 (dd, *J* = 8.2, 4.2 Hz, 1H), 7.02 (d, *J* = 7.4 Hz, 1H), 6.70–6.61 (m, 2H), 3.75 (s, 3H), 2.78–2.65 (m, 4H), 2.33 (s, 3H), 2.04 (s, 3H). **^{13}C NMR (100 MHz, $CDCl_3$):** *δ* 171.46, 156.54, 148.14, 138.38, 138.00, 136.75, 136.44, 134.63, 131.81, 129.59, 128.00, 127.52, 127.38, 121.64, 121.45, 121.08, 116.56, 111.72, 55.38, 38.16, 24.71, 21.53, 17.33. **HRMS (ESI)** m/z Calcd. for $C_{23}H_{24}N_2O_2$ $[M+H]^+$ 361.1916, Found 361.1914.

Synthesis of 6

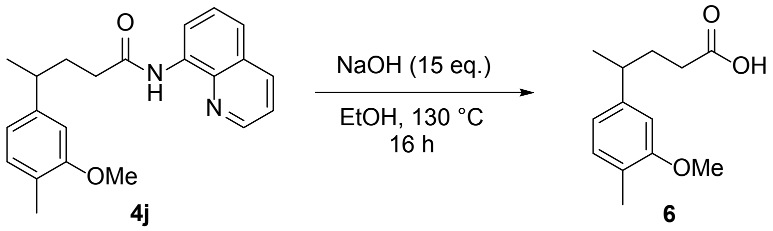

To an oven-dried 25-mL Schlenk tube equipped with a Teflon-coated magnetic stir bar were successively added 4-(3-methoxy-4-methylphenyl)-*N*-(quinolin-8-yl)pentanamide **4j** (0.2 mmol, 0.070 g), NaOH (15 mmol, 0.12 g), and EtOH (4 mL). After stirring at 130 ºC for 16 h, the reaction was allowed to cool to rt, diluted with EtOAc (15 mL), and washed with HCl (1 M, 3 × 8 mL). The organic layers were combined, dried over Na_2SO_4, concentrated under reduced pressure, and purified by flash column chromatography (DCM/HOAc = 100/1) to give **6** (0.038 g, 85%) as a white solid. **^{1}H NMR (400 MHz, $CDCl_3$):** *δ* 7.06 (d, *J* = 7.6 Hz, 1H), 6.69 (d, *J* = 7.6 Hz, 1H), 6.65 (s, 1H), 3.84 (s, 3H), 2.74–2.67 (m, 1H), 2.26 (t, *J* = 7.4 Hz, 2H), 2.20 (s, 3H), 2.01–1.84 (m, 2H), 1.29 (d, *J* = 6.9 Hz, 3H). **^{13}C NMR (100 MHz, $CDCl_3$):** *δ* 180.35, 157.87, 145.10, 130.69, 124.55, 118.74, 108.92, 55.36, 39.48, 33.09, 32.46, 22.44, 15.98.

Synthesis of 7

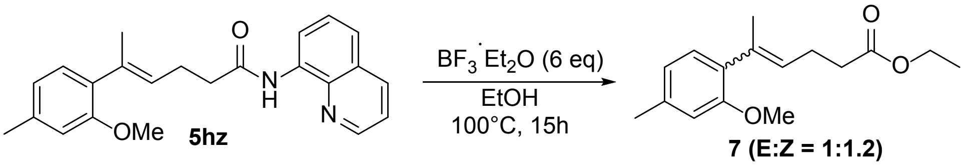

5hz → **7 (E:Z = 1:1.2)**

To an oven-dried 25-mL Schlenk tube equipped with a Teflon-coated magnetic stir bar were successively added (*E*)-5-(2-methoxy-4-methylphenyl)-*N*-(quinolin-8-yl) hex-4-enamide **5hz** (0.072 g, 0.2 mmol, 1 equiv.), anhydrous EtOH (2.0 mL), and $BF_3 \cdot Et_2O$ (0.17 g, 1.2 mmol, 6 equiv.) under a N_2 atmosphere. The resulting mixture was heated at 100 °C for 15 h. The reaction was allowed to cool to rt, and Et_3N (2 mmol, 10 equiv) was added dropwise while stirring. The mixture was concentrated under reduced pressure, and the residue was purified by flash column chromatography on silica gel (PE/EtOAc = 20/1) to afford **7** (0.047 g, 91%) as a colorless oil.[7] The ratio of (*E*)-**7**/(Z)-**7** was 1:1.2, as determined by ^{1}H NMR.

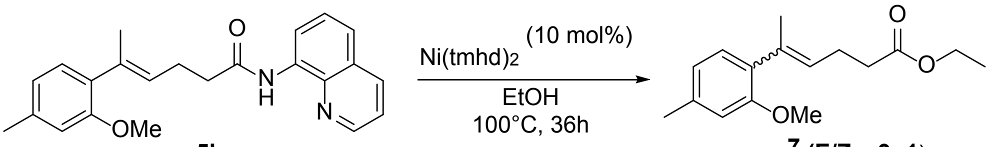

5hz → **7 (E/Z = 9 :1)**

To an oven-dried 25-mL Schlenk tube equipped with a Teflon-coated magnetic stir bar were successively added (*E*)-5-(2-methoxy-4-methylphenyl)-*N*-(quinolin-8-yl) hex-4-enamide **5hz** (0.072 g, 0.2 mmol, 1 equiv.), Ni(tmhd)$_2$ (0.02 mmol, 10 mol %), and anhydrous EtOH (2.0 mL) under a N_2 atmosphere. The resulting mixture was heated at 100 °C for 36 h. After cooling to rt, the mixture was concentrated under reduced pressure, and the residue was purified by flash column chromatography on silica gel (PE/EtOAc = 20/1) to afford **7** (0.030 g, 57%) as a colorless oil.[8] The ratio of (*E*)-**7**/(Z)-**7** was 9:1, as determined by ^{1}H NMR. NMR data of the *E* isomer: **^{1}H NMR (400 MHz, CDCl$_3$):** δ 6.98 (d, J = 7.5 Hz, 1H), 6.71 (d, J = 7.5 Hz, 1H), 6.67 (s, 1H), 5.40 (t, J = 6.8 Hz, 1H), 4.15 (q, J = 7.2 Hz, 2H), 3.80 (s, 3H), 2.53–2.48 (m, 2H), 2.46–2.40 (m, 2H), 2.34 (s, 3H), 1.98 (s, 3H), 1.27 (t, J = 7.2 Hz, 3H). **^{13}C NMR (100 MHz, CDCl$_3$):** δ 173.50, 156.60, 138.09, 136.39, 131.89, 129.57, 127.39, 121.18, 111.83, 60.43, 55.48, 34.43, 24.11, 21.57, 17.24, 14.39.

Mechanistic experiments on the γ-selective hydroarylation

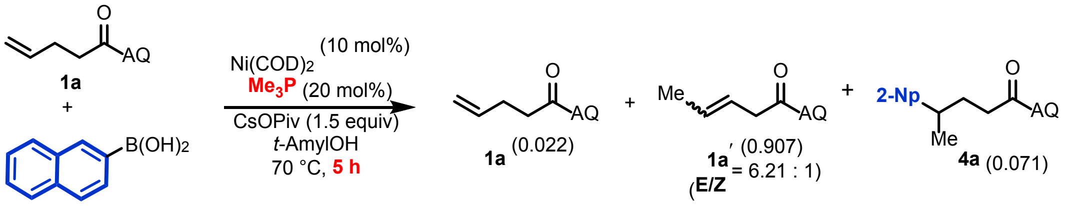

In an argon-filled glovebox, an oven-dried 25-mL Schlenk tube equipped with a Teflon-coated magnetic stir bar was charged successively with alkene **1a** (0.2 mmol, 1.0 equiv.), arylboronic acid **2a** (0.4 mmol, 2.0 equiv.), $Ni(cod)_2$ (0.02 mmol, 0.0055 g, 0.1 equiv.), CsOPiv (0.3 mmol, 0.0702 g, 1.5 equiv.), anhydrous *t*-AmylOH (1 mL), and PMe_3 (0.04 mmol, 10% in toluene, 0.2 equiv.). The tube was sealed with a Teflon screw cap, moved out of the glovebox, and placed on a hotplate pre-heated to 70 °C with vigorous stirring for 5 h. Then, the reaction mixture was cooled to rt and diluted with EtOAc. The organic layer was washed with brine solution and was then dried over anhydrous $MgSO_4$. The reaction mixture was filtered through a short pad of Celite, and the solvent was evaporated under vacuum to give the crude product, which was analyzed by ^{1}H NMR (**Supplementary Figure 2**).

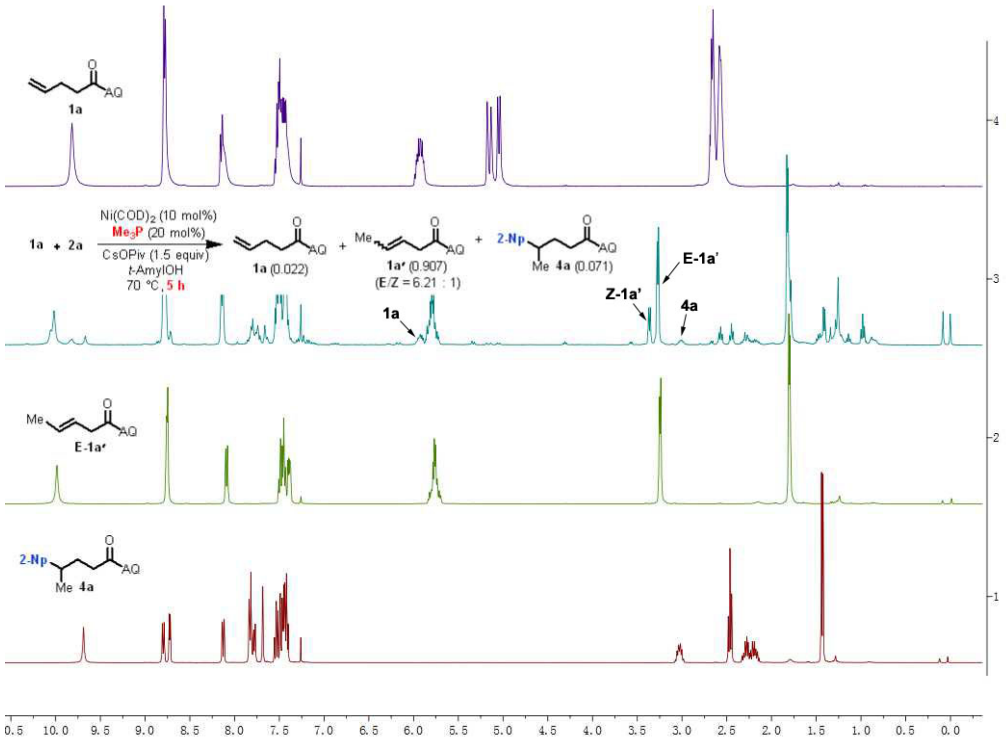

Supplementary Figure 2. The ^{1}H NMR monitoring of hydroarylation reaction for 5 h.

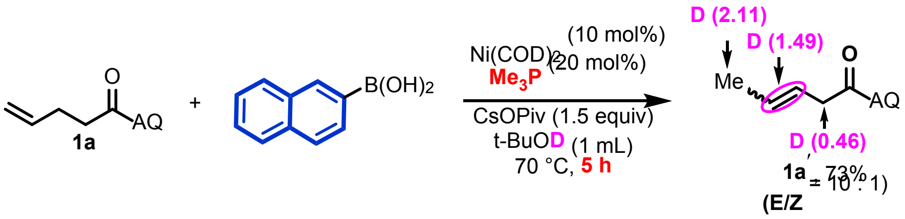

In an argon-filled glovebox, an oven-dried 25-mL Schlenk tube equipped with a Teflon-coated magnetic stir bar was charged successively with alkene **1a** (0.2 mmol, 1.0 equiv.), arylboronic acid **2a** (0.4 mmol, 2.0 equiv.), Ni(cod)$_2$ (0.02 mmol, 0.0055 g, 0.1 equiv.), CsOPiv (0.3 mmol, 0.0702 g, 1.5 equiv.), anhydrous *t*-BuOD (1 mL), and PMe$_3$ (0.04 mmol, 10% in toluene, 0.2 equiv.). The tube was sealed with a Teflon screw cap, moved out of the glovebox, and placed on a hotplate pre-heated to 70 °C with vigorous stirring for 5 h. Then, the reaction mixture was cooled to rt and diluted with EtOAc. The organic layer was washed with brine solution and was then dried over anhydrous MgSO$_4$. The reaction mixture was filtered through a short pad of Celite, and the solvent was evaporated under vacuum to give the crude product. The resulting residue was purified by flash chromatography on silica gel (hexane/ EtOAc = 10/1) to afford the isomer **1a'**. The ratio of deuterium incorporation was determined by ^{1}H NMR (**Supplementary Figure 3**).

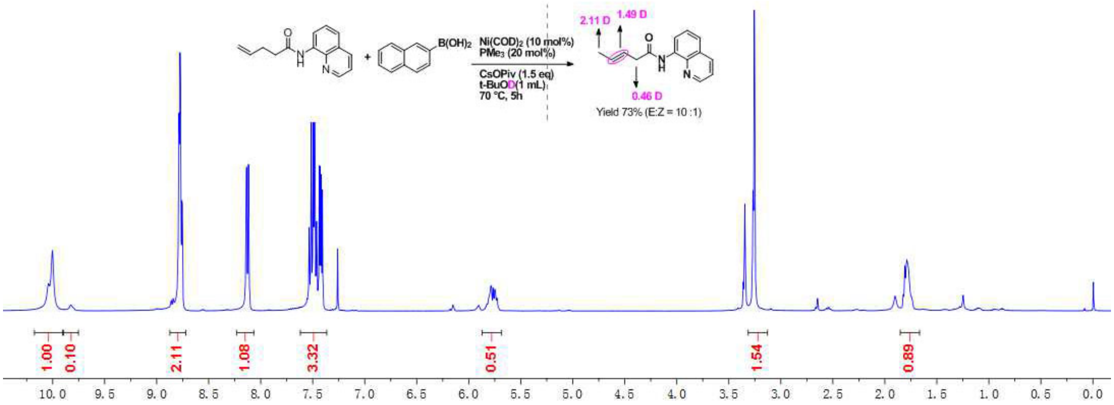

Supplementary Figure 3. ^{1}H NMR spectra to check the ratio of deuterium incorporation

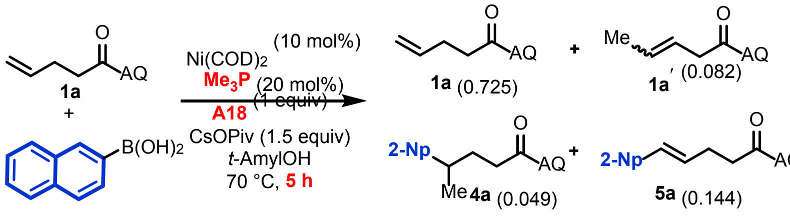

In an argon-filled glovebox, an oven-dried 25-mL Schlenk tube equipped with a Teflon-coated magnetic stir bar was charged successively with alkene **1a** (0.2 mmol, 1.0 equiv.), arylboronic acid **2a** (0.4 mmol, 2.0 equiv.), Ni(cod)$_2$ (0.02 mmol, 0.0055 g, 0.1 equiv.), CsOPiv (0.3 mmol, 0.0702 g, 1.5 equiv.), (*E*)-3-(3,4,5-trimethoxyphenyl)acrylaldehyde **A18** (0.2 mmol, 0.0444 g, 1.0 equiv.), anhydrous *t*-AmylOH (1 mL), and PMe$_3$ (0.04 mmol, 10% in toluene, 0.2 equiv.). The tube was sealed with a Teflon screw cap, moved out of the glovebox, and placed on a hotplate pre-heated to 70 °C with vigorous stirring for 5 h. Then, the reaction mixture was cooled to rt and diluted with EtOAc. The organic layer was washed with brine solution and was then dried over anhydrous MgSO$_4$. The reaction mixture was filtered through a short pad of Celite, and the solvent was evaporated under vacuum to give the crude product, which was analyzed by ^{1}H NMR (**Supplementary Figure 4**).

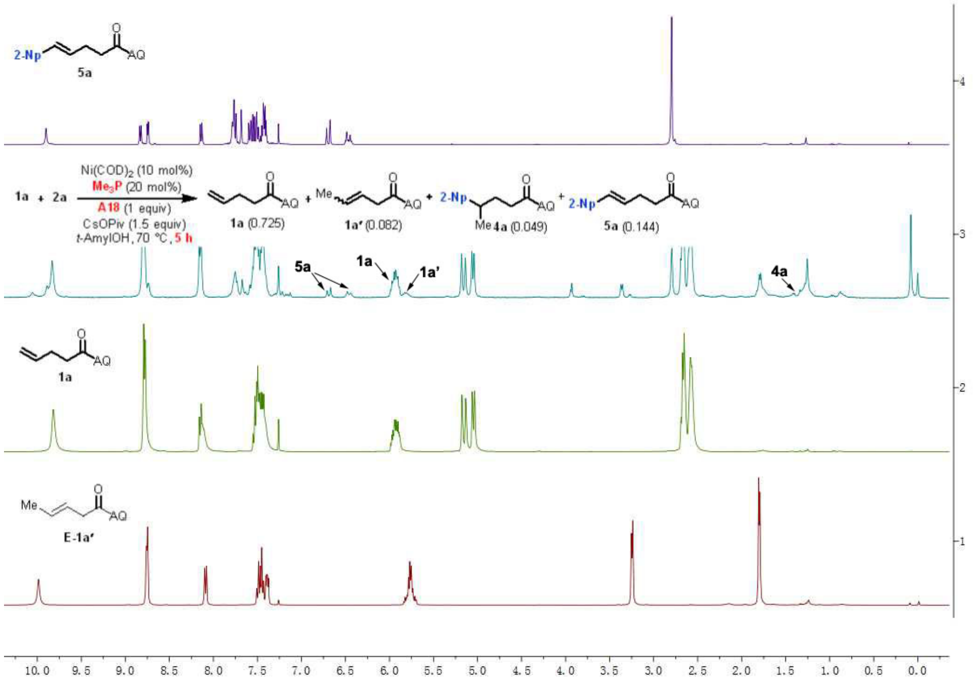

Supplementary Figure 4. The ^{1}H NMR spectra of the reaction in presence of **A18** acceptor for 5 h.

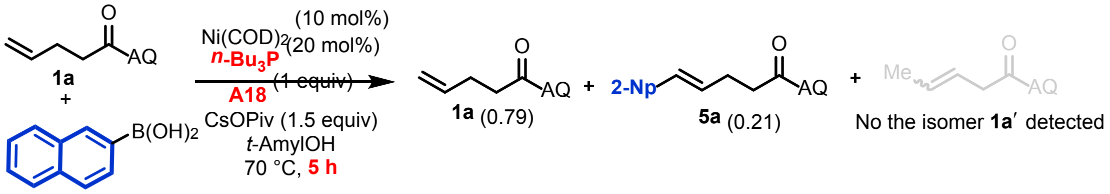

In an argon-filled glovebox, an oven-dried 25-mL Schlenk tube equipped with a Teflon-coated magnetic stir bar was charged successively with alkene **1a** (0.2 mmol, 1.0 equiv.), arylboronic acid **2a** (0.4 mmol, 2.0 equiv.), Ni(cod)$_2$ (0.02 mmol, 0.0055 g, 0.1 equiv.), CsOPiv (0.3 mmol, 0.0702 g, 1.5 equiv.), (*E*)-3-(3,4,5-trimethoxyphenyl)acrylaldehyde **A18** (0.2 mmol, 0.0444 g, 1.0 equiv.), anhydrous *t*-AmylOH (1 mL), and *n*-Bu$_3$P (0.04 mmol, 0.0081 g, 0.2 equiv.). The tube was sealed with a Teflon screw cap, moved out of the glovebox, and placed on a hotplate pre-heated to 70 °C with vigorous stirring for 5 h. Then, the reaction mixture was cooled to rt and diluted with EtOAc. The organic layer was washed with brine solution and was then dried over anhydrous MgSO$_4$. The reaction mixture was filtered through a short pad of Celite, and the solvent was evaporated under vacuum to give the crude product, which was analyzed by ^{1}H NMR (**Supplementary Figure 5**).

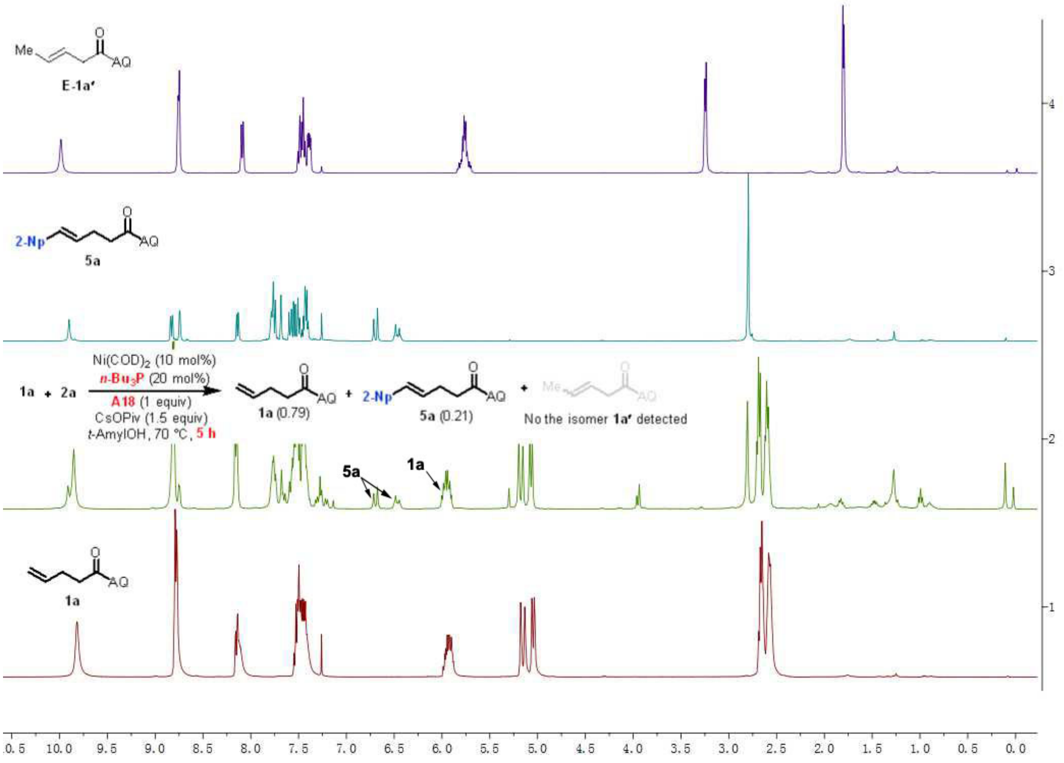

Supplementary Figure 5. ^{1}H NMR spectra of Heck coupling for 5 h.

Mechanistic experiments on the oxidative Heck reaction

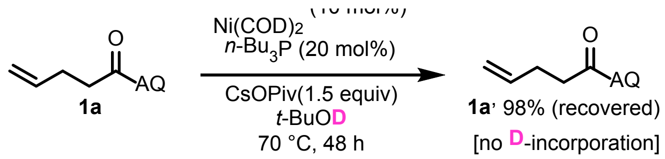

In an argon-filled glovebox, an oven-dried 25-mL Schlenk tube equipped with a Teflon-coated magnetic stir bar was successively charged with alkene **1a** (0.2 mmol, 1.0 equiv.), Ni(cod)$_2$ (0.02 mmol, 0.0055 g 0.1 equiv.), CsOPiv (0.3 mmol, 0.0702 g, 1.5 equiv.), *t*-BuO**D** (1 mL), and *n*-Bu$_3$P (0.04 mmol, 0.0081 g, 0.2 equiv.). The tube was sealed with a Teflon screw cap, moved out of the glovebox, and placed on a hotplate pre-heated to 70 °C with vigorous stirring. After 48 h, the reaction mixture was cooled to rt and diluted with EtOAc. The organic layer was washed with brine solution and was then dried over anhydrous MgSO$_4$. The reaction mixture was filtered through a short pad of Celite, and the solvent was evaporated under vacuum to give the crude mixture. The resulting residue was purified by flash chromatography on silica gel (hexane/ EtOAc = 10/1) to recover **1a** (98%).

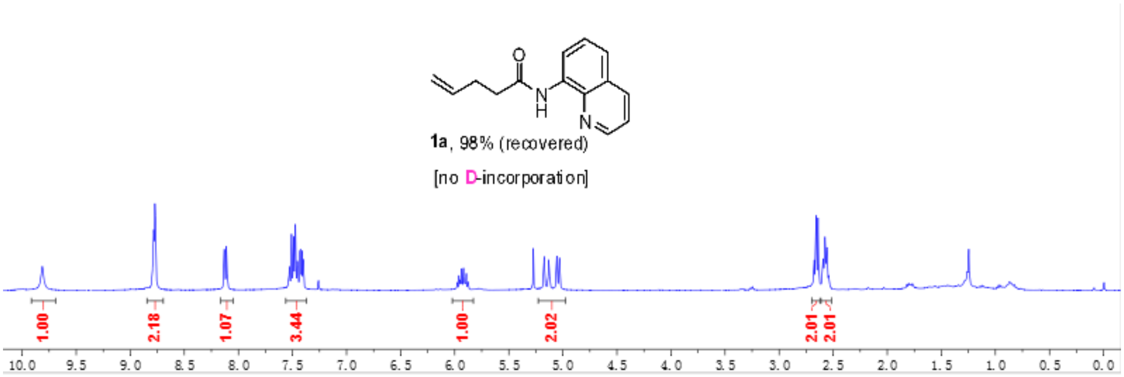

Supplementary Figure 6. ^{1}H NMR spectra of the Heck coupling in the absence of aryl boronic acid.

Synthesis of 1a-d_2

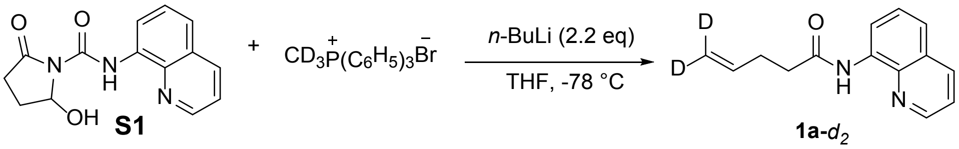

To a suspension of methyl triphenylphosphonium bromide (1.6 g, 4.38 mmol) in anhydrous THF (10 mL) at –78 °C under argon was added *n*-BuLi (1.3mL, 2.5 M in

hexanes, 3.2 mmol), and the resulting solution was stirred for 30 min at –78 °C. A solution of compound **S1** (0.33 g, 1.46mmol) in THF (10 mL) was then added, then warmed to rt and stirred for 12 h. The reaction was quenched with brine (20 mL) and extracted with ethyl acetate (2 × 30 mL). The combined organic layer was washed with brine (30 mL), dried over Na_2SO_4. The solution was concentrated under vacuum, and the resulting residue was purified by flash chromatography on silica gel (PE/EtOAc = 10/1) to afford the **1a-*d*₂** in 81 % yield. **^{1}H NMR (400 MHz, $CDCl_3$):** δ 9.82 (s, 1H), 8.84–8.72 (m, 2H), 8.13 (d, *J* = 8.1 Hz, 1H), 7.58–7.35 (m, 3H), 6.02–5.81 (m, 1H), 5.14 (d, *J* = 17.1 Hz, 0.2H), 5.03 (d, *J* = 10.2 Hz, 0.2H), 2.66 (dd, *J* = 7.9, 6.3 Hz, 2H), 2.59–2.54 (m, 2H).

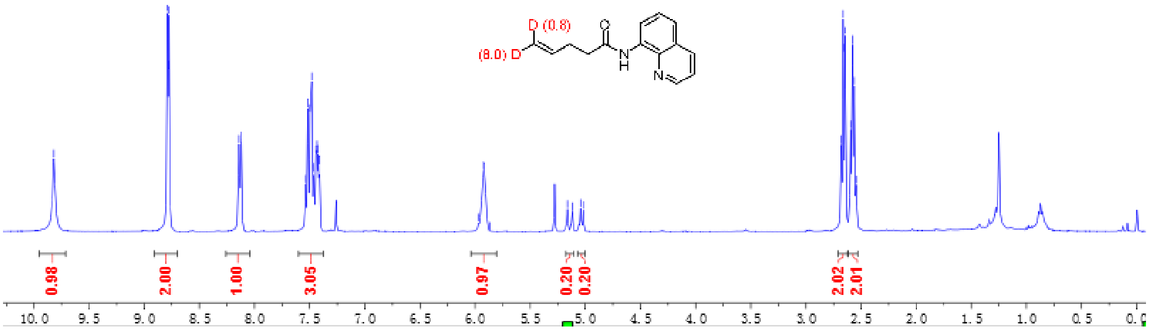

Supplementary Figure 7. ^{1}H NMR spectra of the **1a-*d*₂** compound.

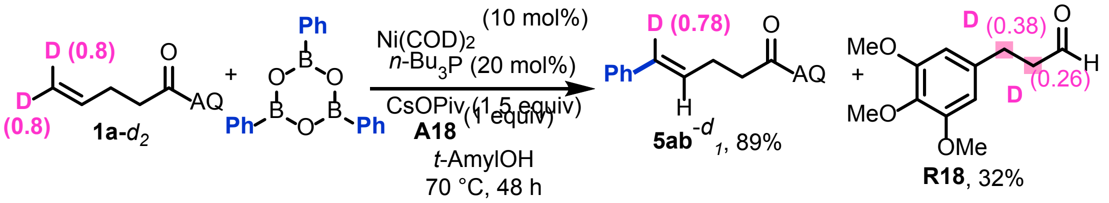

In an argon-filled glove-box, an oven-dried 25 mL Schlenk tube equipped with a Teflon-coated magnetic stir bar were subsequently added alkene **1a-*d*₂** (0.2 mmol, 1.0 equiv.), 2,4,6-Triphenylboroxine (0.13 mmol, 41.6 mg), $Ni(cod)_2$ (0.02 mmol, 0.0055 g, 0.1 equiv.), CsOPiv (0.3 mmol, 0.0702 g, 1.5 equiv.), (*E*)-3-(3,4,5-trimethoxyphenyl)acrylaldehyde **A18** (0.2 mmol, 0.0444 g, 1.0 equiv.), anhydrous *t*-AmylOH (1 mL), and *n*-Bu_3P (0.04 mmol, 0.0081 g, 0.2 equiv.). The tube was sealed with a Teflon screw cap, moved out of the glovebox, and placed on a

hotplate pre-heated to 70 °C with vigorous stirring. After 48 h, the reaction mixture was cooled to rt and diluted with EtOAc. The organic layer was washed with brine solution and was then dried over anhydrous $MgSO_4$. The reaction mixture was filtered through a short pad of Celite, and the solvent was evaporated under vacuum to give the crude product. The resulting residue was purified by flash chromatography on silica gel to afford **5ab-*d*$_1$** in 89% yield, and reduced **R18** was obtained as colorless oil in 32% yield.

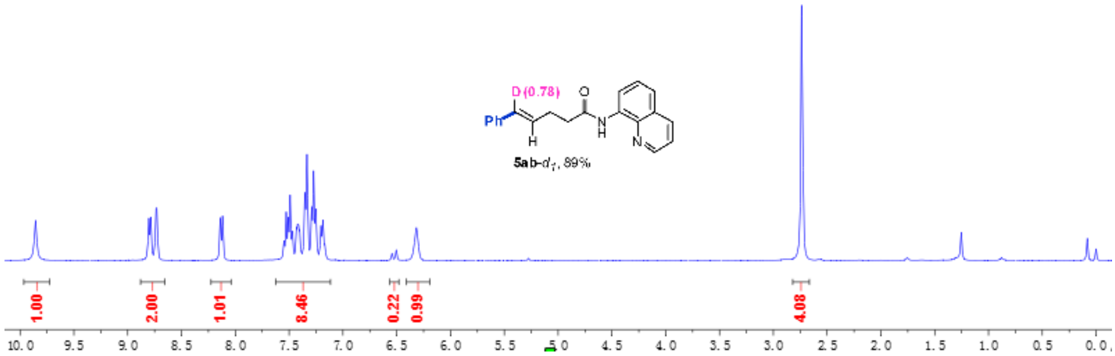

Supplementary Figure 8. ^{1}H NMR spectra of the **5ab-*d*$_1$** compound.

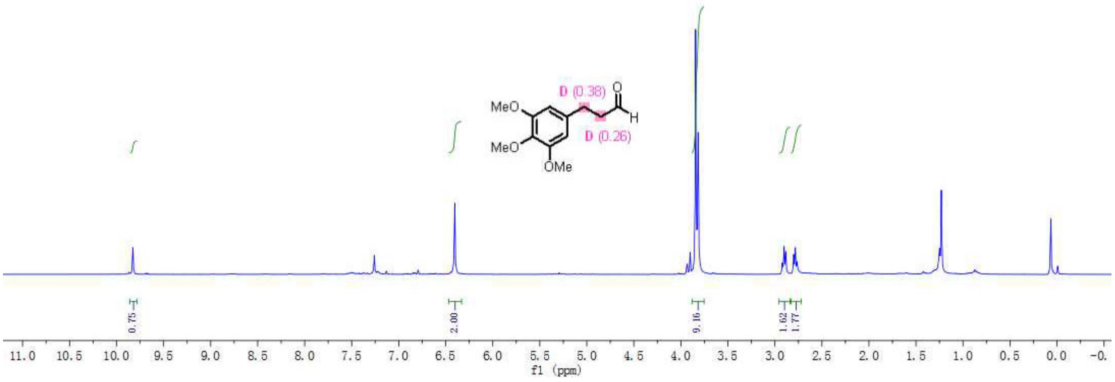

Supplementary Figure 9. ^{1}H NMR spectra of the **R18** compound by using the **1a-*d*$_2$** as the alkene substrate

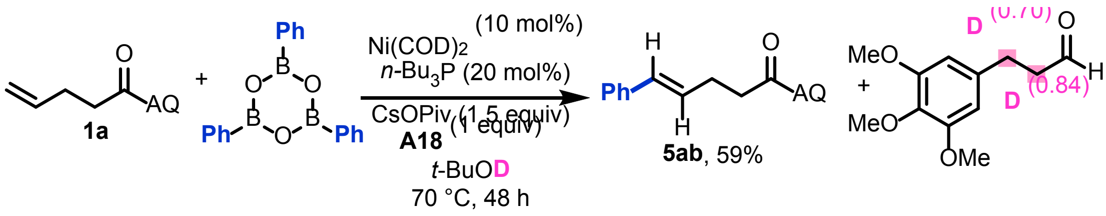

In an argon-filled glovebox, an oven-dried 25-mL Schlenk tube equipped with a Teflon-coated magnetic stir bar was successively charged with alkene **1a** (0.2 mmol,

1.0 equiv.), 2,4,6-triphenylboroxine (0.13 mmol, 41.6 mg), Ni(cod)$_2$ (0.02 mmol, 0.0055 g, 0.1 equiv.), CsOPiv (0.3 mmol, 0.0702 g, 1.5 equiv.), (*E*)-3-(3,4,5-trimethoxyphenyl)acrylaldehyde **A18** (0.2 mmol, 0.0444 g, 1.0 equiv.), *t*-BuOD (1 mL), and *n*-Bu$_3$P (0.04 mmol, 0.0081 g, 0.2 equiv.). The tube was sealed with a Teflon screw cap, moved out of the glovebox, and placed on a hotplate pre-heated to 70 °C with vigorous stirring. After 48 h, the reaction mixture was cooled to rt and diluted with EtOAc. The organic layer was washed with brine solution and was then dried over anhydrous $MgSO_4$. The reaction mixture was filtered through a short pad of Celite, and the solvent was evaporated under vacuum to give the crude product. The resulting residue was purified by flash chromatography on silica gel to afford **5ab** in 59% yield, and reduced **R18** was obtained as colorless oil in 21% yield.

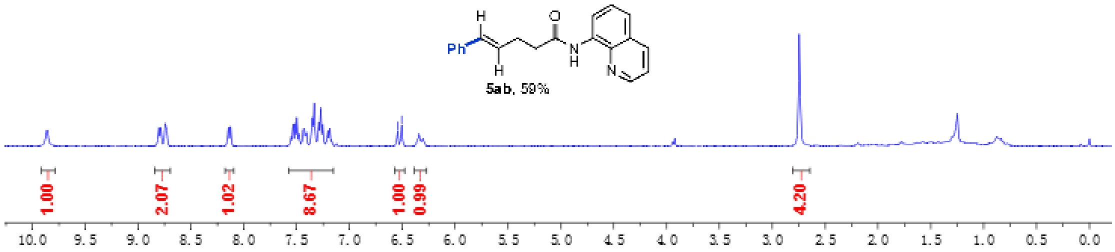

Supplementary Figure 10. ^{1}H NMR spectra of the **5ab** compound.

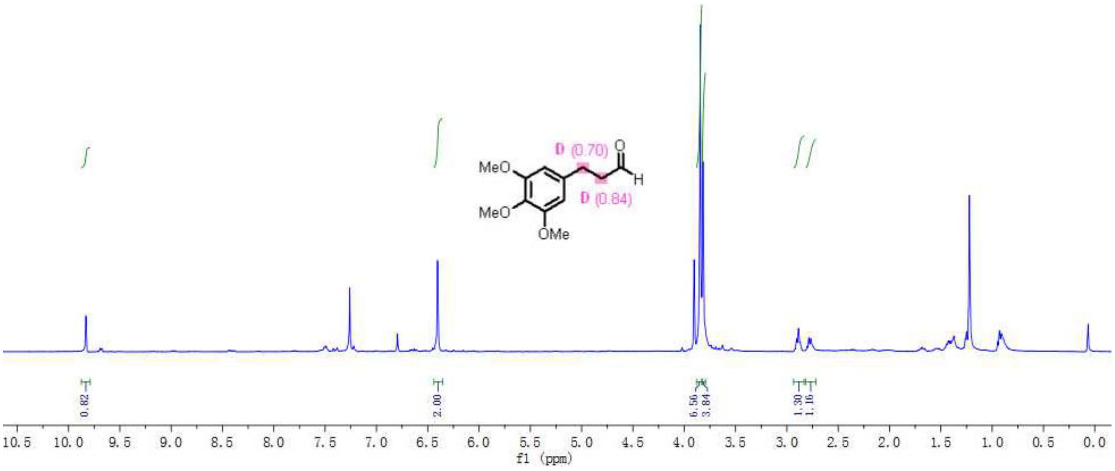

Supplementary Figure 11. ^{1}H NMR spectra of the **R18** compound by using *t*-BuOD as the reaction solvent.

The evidence on producing RO-Ni-H species

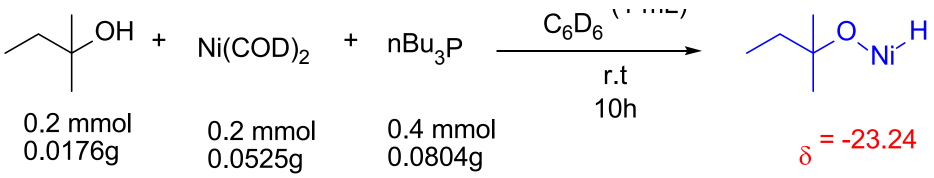

General procedure: In an argon-filled glovebox, a 8-mL glass vial equipped with a Teflon-coated magnetic stir bar was charged successively with $Ni(COD)_2$ (0.2 mmol, 0.0525g), C_6D_6 (1 mL), anhydrous 2-methyl-2-butanol (0.2 mmol, 0.0176g), and $n$$Bu_3P$ (0.4 mmol, 0.0804 g). The vials were sealed with a PTFE cap, and the solution was vigorously stirred at rt for 10 h. The reaction mixture was filtered through a PTFE membrane (0.22 um), and analyzed by ^{1}H NMR (400 MHz) spectroscopy. A signal (δ = -23.24) consistent with formation of a nickel–hydride species was observed.[9]

Dependence of the reaction rate on concentration of compound 1a

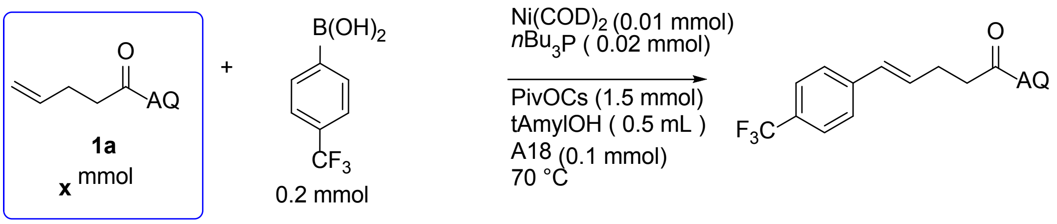

General procedure: In an argon-filled glovebox, a group of 8-mL glass vials (5 in total, each equipped with a Teflon-coated magnetic stir bar) were charged successively with alkene **1a** (x mmol), (4-CF_3)-$C_6H_4B(OH)_2$ (0.2 mmol), $Ni(cod)_2$ (0.01 mmol, 0.00275 g), CsOPiv (0.15 mmol, 0.035 g), (*E*)-3-(3,4,5-trimethoxyphenyl)acrylaldehyde **A18** (0.1 mmol, 0.022 g), anhydrous *t*-AmylOH (0.5 mL), and *n*-Bu_3P (0.02 mmol, 0.0041 g). The vials were sealed with PTFE caps, moved out of the glovebox, and placed on a hotplate pre-heated to 70 °C and stirred vigorously for the indicated time. Every 45 min, one reaction vial was removed from the hotplate, cooled to rt, and quenched with EtOAc. The reaction mixture was filtered through a short pad of Celite, and the solvent was evaporated under vacuum to give the crude

reaction mixture. Yields were determined by ^{1}H NMR spectroscopy with an internal standard (phenyl(trifluoromethyl)sulfane).

The initial rates at different initial concentrations of compound **1a** (from 0.1 mmol mL^{-1} to 0.4 mmol mL^{-1}) were measured (**Supplementary Figure 12**). Then, the rates were plotted against the initial concentration of compound **1a** (**Supplementary Figure 13**).

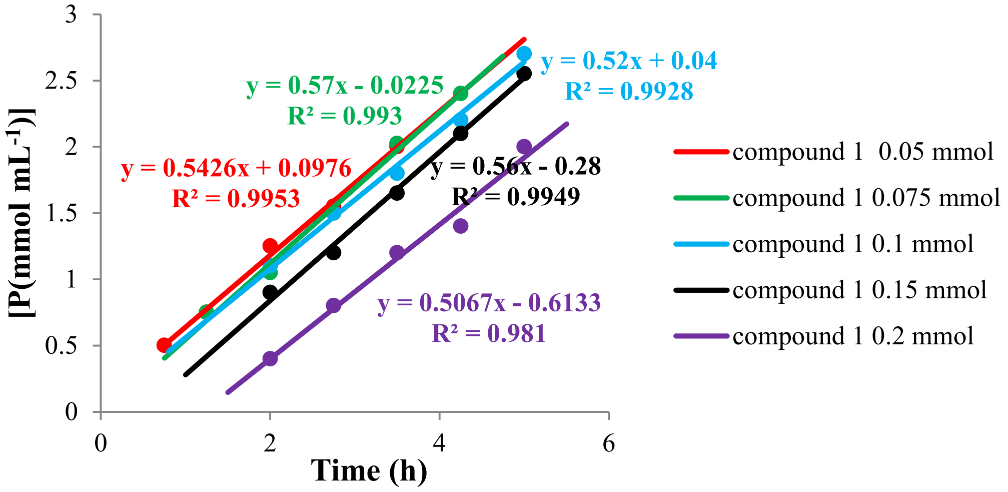

Supplementary Figure 12. Reaction profiles for compound **1a**.

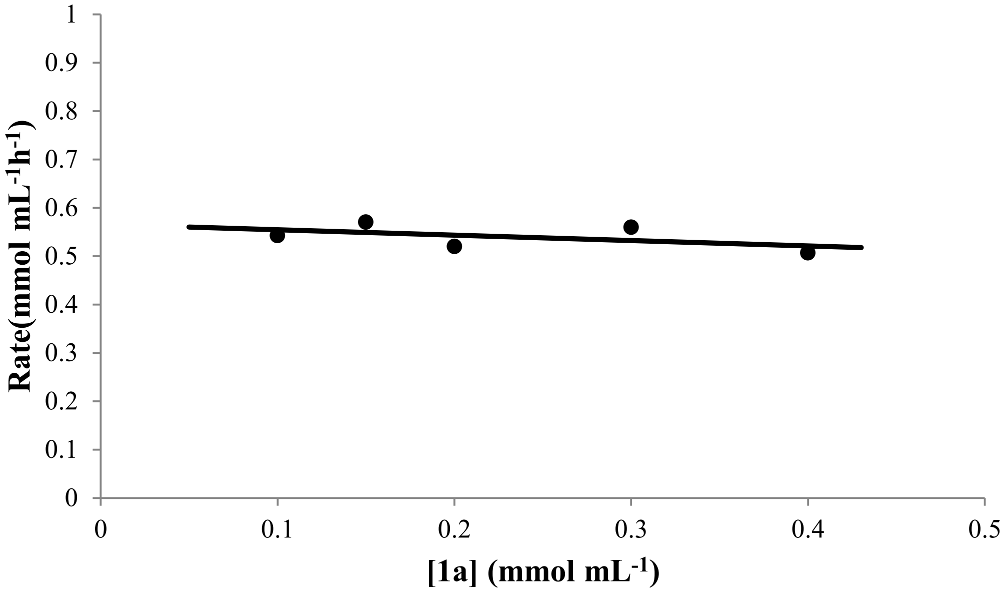

Supplementary Figure 13. Initial reaction rate dependence on concentration of alkene **1a**.

Dependence of the reaction rate on concentration of $(4\text{-}CF_3)C_6H_4B(OH)_2$

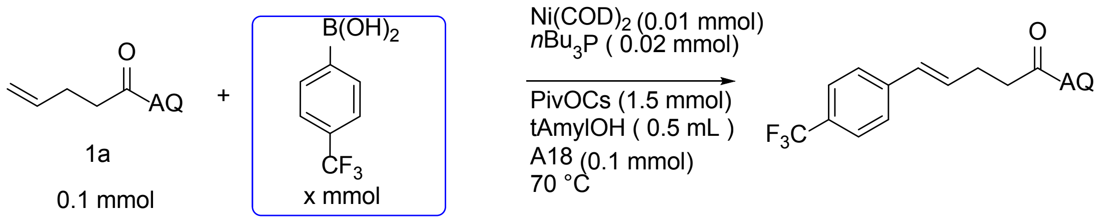

General procedure: In an argon-filled glovebox, a group of 8-mL glass vials (5 in total, each equipped with a Teflon-coated magnetic stir bar) were charged successively with alkene **1a** (0.1 mmol, 0.0226 g), (4-CF_3)$C_6H_4B(OH)_2$ (x mmol), $Ni(cod)_2$ (0.01 mmol, 0.00275 g), CsOPiv (0.15 mmol, 0.035 g), (*E*)-3-(3,4,5-trimethoxyphenyl)acrylaldehyde **A18** (0.1 mmol, 0.022 g), anhydrous *t*-AmylOH (0.5 mL), and *n*-Bu_3P (0.02 mmol, 0.0041 g). The vials were sealed with PTFE caps, moved out of the glovebox, and placed on a hotplate pre-heated to 70 °C and stirred vigorously for the indicated time. Every 45 min, one reaction vial was removed from the hotplate, cooled to rt, and quenched with EtOAc. The reaction mixture was filtered through a short pad of Celite, and the solvent was evaporated under vacuum to give the crude reaction mixture. Yields were determined by ^1H NMR spectroscopy with an internal standard (phenyl(trifluoromethyl)sulfane).

Kinetic profiles of different initial concentrations of (4-CF_3)$C_6H_4B(OH)_2$ (from 0.2 mmol mL^{-1} to 0.8 mmol L^{-1}) were collected (**Supplementary Figure 14**). The rate was plotted against the concentration of (4-CF_3)$C_6H_4B(OH)_2$ (**Supplementary Figure 15**).

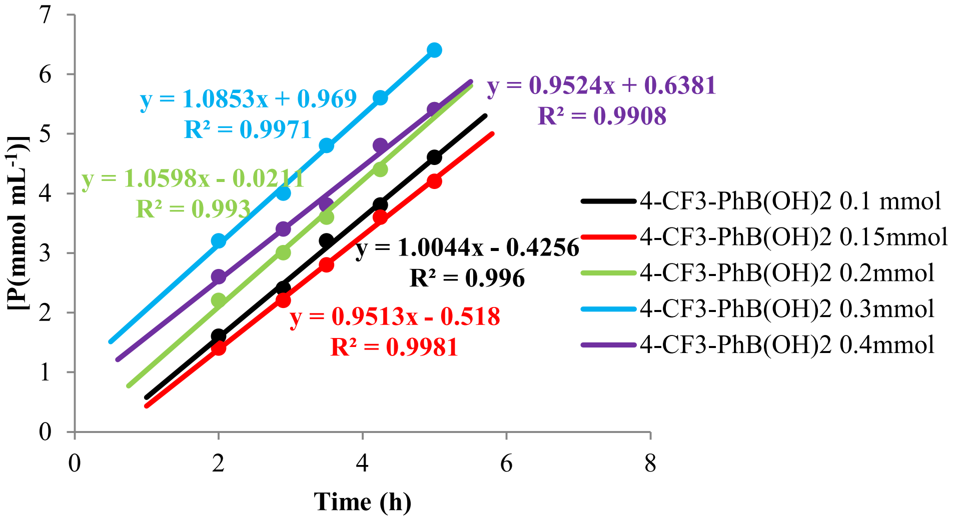

Supplementary Figure 14. Reaction profiles for (4-CF_3)$C_6H_4B(OH)_2$.

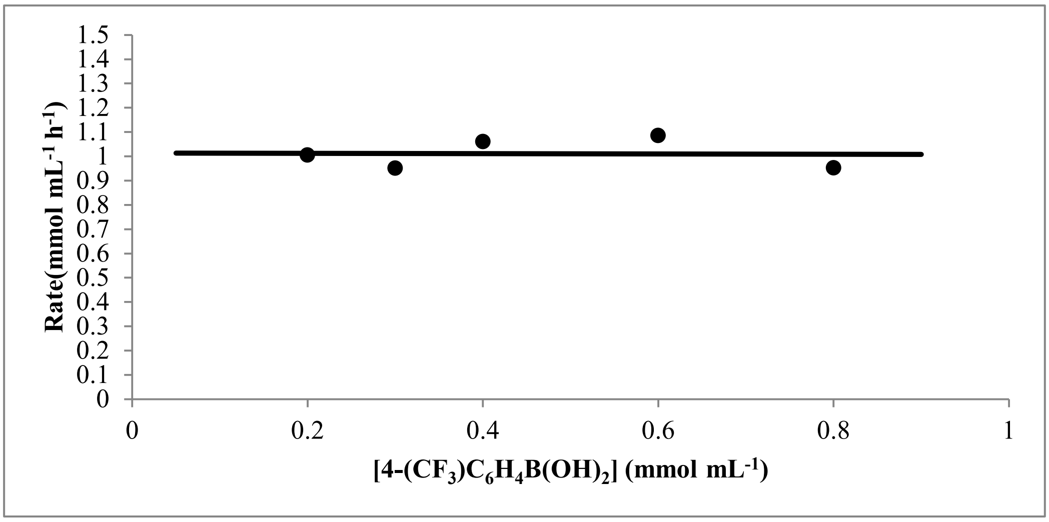

Supplementary Figure 15. Initial reaction rate dependence on concentration of $(4\text{-}CF_3)C_6H_4B(OH)_2$.

Dependence of the reaction rate on concentration of A18

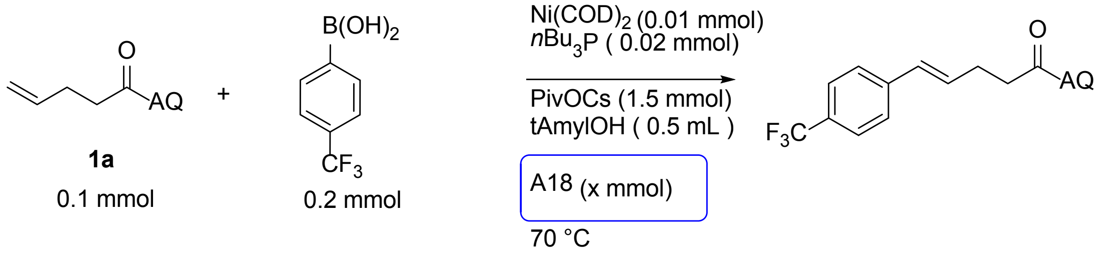

General procedure: In an argon-filled glovebox, a group of 8-mL glass vials (5 in total, each equipped with a Teflon-coated magnetic stir bar) were charged successively with alkene **1a** (0.1 mmol, 0.0226 g), $(4\text{-}CF_3)C_6H_4B(OH)_2$ (0.2 mmol, 0.0344 g), $Ni(cod)_2$ (0.01 mmol, 0.0275 g), CsOPiv (0.15 mmol, 0.035 g), (*E*)-3-(3,4,5-trimethoxyphenyl)acrylaldehyde **A18** (x mmol), anhydrous *t*-AmylOH (0.5 mL), and *n*-Bu_3P (0.02 mmol, 0.0041 g). The vials were sealed with PTFE caps, moved out of the glovebox, and placed on a hotplate pre-heated to 70 °C and stirred vigorously for the indicated time. Every 45 min, one reaction vial was removed from the hotplate, cooled to rt, and quenched with EtOAc. The reaction mixture was filtered through a short pad of Celite, and the solvent was evaporated under vacuum to give the crude reaction mixture. Yields were determined by ^1H NMR spectroscopy with an internal standard (phenyl(trifluoromethyl)sulfane).

Kinetic profiles of different initial concentrations of **A18** (from 0.1 mmol mL^{-1} to 0.4 mmol L^{-1}) were collected (**Supplementary Figure 16**). The rate was plotted against the concentration of $(4\text{-}CF_3)C_6H_4B(OH)_2$ (**Supplementary Figure 17**).

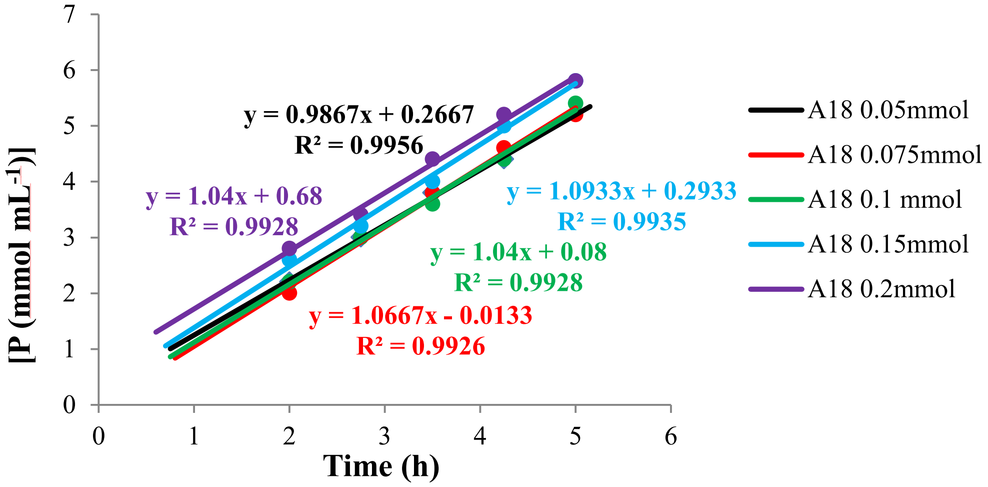

Supplementary Figure 16. Reaction profiles for **A18** .

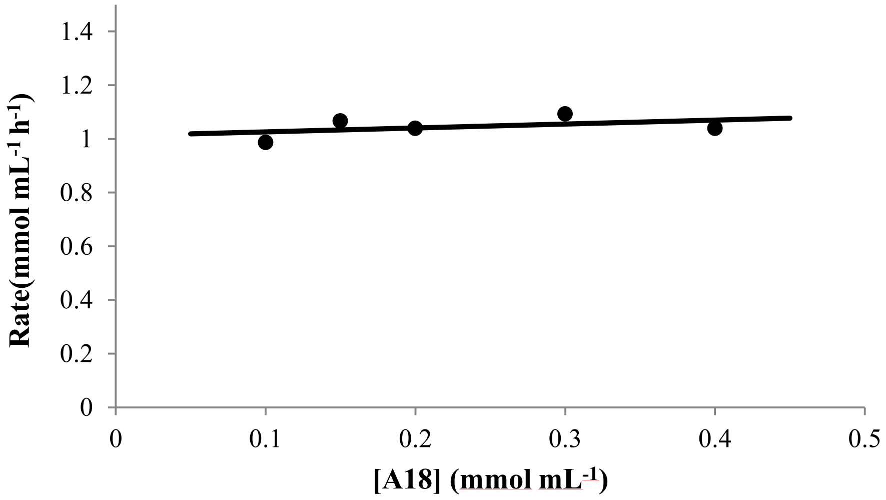

Supplementary Figure 17. Initial reaction rate dependence on concentration of **A18** .

Dependence of the reaction rate on concentration of $Ni(COD)_2$

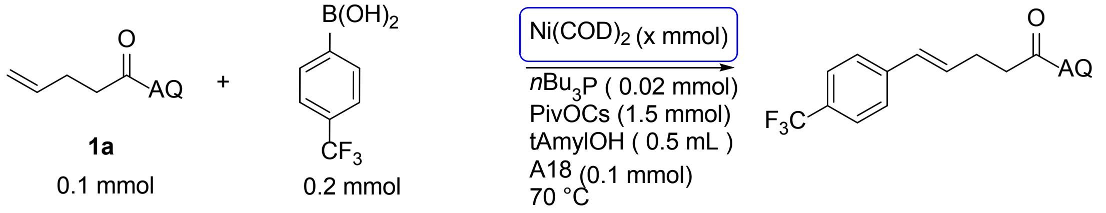

General procedure: In an argon-filled glovebox, a group of 8-mL glass vials (5 in total, each equipped with a Teflon-coated magnetic stir bar) were charged successively with alkene **1a**

(0.1 mmol, 0.0226 g), (4-CF_3)$C_6H_4B(OH)_2$ (0.2 mmol, 0.0344 g), Ni(cod)$_2$ (x mmol), CsOPiv (0.15 mmol, 0.035 g), (*E*)-3-(3,4,5-trimethoxyphenyl)acrylaldehyde **A18** (0.1 mmol, 0.022 g), anhydrous *t*-AmylOH (0.5 mL), and *n*-Bu_3P (0.02 mmol, 0.0041 g). The vials were sealed with PTFE caps, moved out of the glovebox, and placed on a hotplate pre-heated to 70 °C and stirred vigorously for the indicated time. Every 45 min, one reaction vial was removed from the hotplate, cooled to rt, and quenched with EtOAc. The reaction mixture was filtered through a short pad of Celite, and the solvent was evaporated under vacuum to give the crude reaction mixture. Yields were determined by ^{1}H NMR spectroscopy with an internal standard (phenyl(trifluoromethyl)sulfane).

Kinetic profiles of different initial concentrations of Ni(COD)$_2$ (from 0.01 mmol mL^{-1} to 0.04 mmol L^{-1}) were collected (**Supplementary Figure 18**). The rate was plotted against the concentration of Ni(COD)$_2$ (**Supplementary Figure 19**).

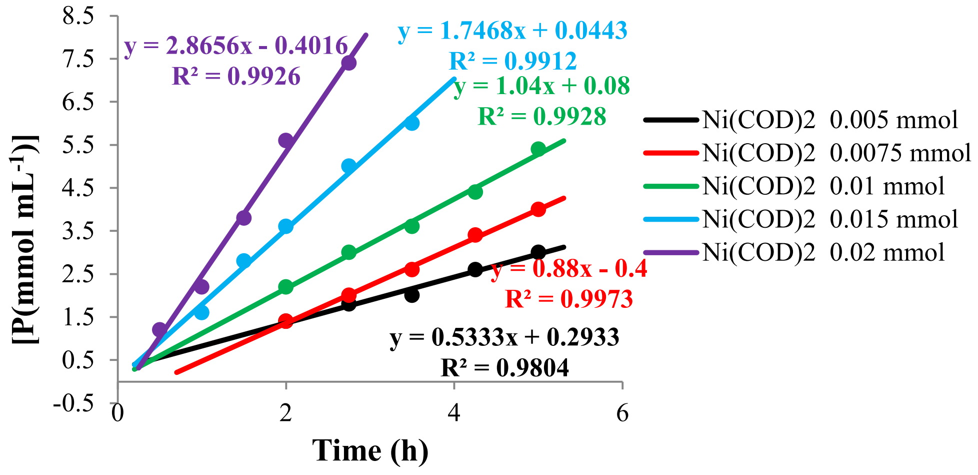

Supplementary Figure 18. Reaction profiles for Ni(COD)$_2$.

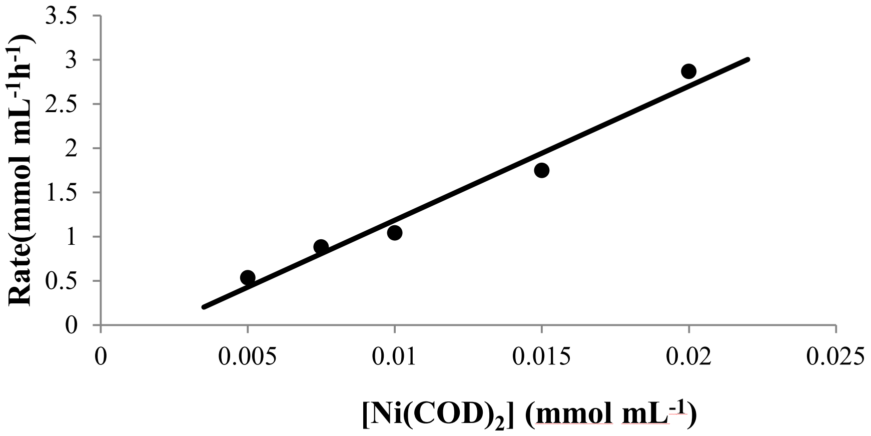

Supplementary Figure 19. Initial reaction rate dependence on concentration of $Ni(COD)_2$.

X-ray Crystal Structure Data

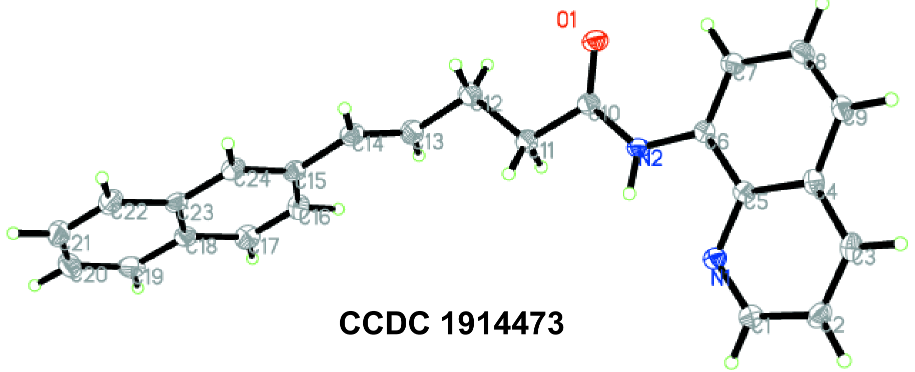

Supplementary Figure 20. ORTEP plot of compound **5a**. All H atoms have been omitted for clarity. The crystal was kept at 113.15 K during data collection.

Supplementary Table 7 Crystal data and structure refinement for **5a**.

Identification code	**5a**
Empirical formula	$C_{24}H_{20}N_2O$
Formula weight	352.42
Temperature/K	113.15
Crystal system	orthorhombic
Space group	$P2_12_12_1$
a/Å	5.8681(2)
b/Å	8.2806(4)
c/Å	37.3551(18)
α/°	90
β/°	90
γ/°	90
Volume/Å^3	1815.12(14)

Z	4
ρ_{calc}g/cm^3	1.290
μ/mm^{-1}	0.079
F(000)	744.0
Crystal size/mm^3	0.2 × 0.18 × 0.12
Radiation	MoKα (λ = 0.71073)
2Θ range for data collection/°	4.362 to 52.738
Index ranges	-7 ≤ h ≤ 7, -10 ≤ k ≤ 10, -46 ≤ l ≤ 46
Reflections collected	15887
Independent reflections	3694 [R_{int} = 0.0676, R_{sigma} = 0.0518]
Data/restraints/parameters	3694/1/248
Goodness-of-fit on F^2	1.074
Final R indexes [I>=2σ (I)]	R_1 = 0.0461, wR_2 = 0.0946
Final R indexes [all data]	R_1 = 0.0556, wR_2 = 0.1000
Largest diff. peak/hole / e Å^{-3}	0.15/-0.15
Flack parameter	0(3)

Supplementary Table 8. Fractional Atomic Coordinates (×10^4) and Equivalent Isotropic Displacement Parameters (Å^2×10^3) for 5a. U_{eq} is defined as 1/3 of of the trace of the orthogonalised U_{IJ}tensor.

**Atom**	***x***	***y***	***z***	**U(eq)**
O(1)	4420(3)	2564(2)	838.7(5)	41.2(5)
N(1)	1463(4)	8068(2)	762.9(5)	29.7(5)
N(2)	3483(4)	5227(3)	841.4(5)	29.3(5)
C(1)	412(5)	9470(3)	747.4(6)	34.2(6)
C(2)	-1684(5)	9722(3)	572.5(6)	35.6(6)
C(3)	-2659(5)	8459(3)	397.2(6)	33.0(6)
C(4)	-1594(4)	6932(3)	395.4(6)	28.1(6)
C(5)	476(4)	6793(3)	590.9(6)	25.7(5)
C(6)	1571(4)	5261(3)	617.4(6)	26.3(5)
C(7)	686(5)	3958(3)	437.0(6)	31.9(6)
C(8)	-1336(5)	4130(3)	236.2(6)	35.4(6)
C(9)	-2462(5)	5554(3)	217.3(6)	32.6(6)
C(10)	4768(4)	3935(3)	945.1(6)	29.8(6)
C(11)	6643(5)	4374(3)	1204.6(7)	31.9(6)
C(12)	7892(6)	2929(4)	1358.9(8)	47.3(8)
C(13)	9663(5)	3418(4)	1626.7(7)	41.3(7)
C(14)	9561(5)	3100(3)	1970.5(7)	38.0(7)
C(15)	11185(4)	3565(3)	2250.4(6)	31.4(6)
C(16)	13218(5)	4429(4)	2174.7(7)	38.9(7)

C(17)	14729(5)	4814(4)	2435.6(7)	41.9(7)
C(18)	14329(5)	4378(3)	2795.7(7)	34.4(6)
C(19)	15900(5)	4754(4)	3073.1(8)	46.2(8)
C(20)	15445(6)	4305(4)	3419.1(8)	51.6(9)
C(21)	13432(6)	3501(4)	3500.7(8)	51.2(8)
C(22)	11898(6)	3117(4)	3242.0(7)	43.6(7)
C(23)	12307(5)	3539(3)	2881.3(6)	32.0(6)
C(24)	10772(5)	3139(3)	2600.6(7)	34.6(6)

Supplementary Table 9. Anisotropic Displacement Parameters ($Å^2 \times 10^3$) for 5a. The Anisotropic displacement factor exponent takes the form: $-2\pi^2[h^2a^{*2}U_{11}+2hka^*b^*U_{12}+\cdots]$.

**Atom**	**U_{11}**	**U_{22}**	**U_{33}**	**U_{23}**	**U_{13}**	**U_{12}**
O(1)	43.4(12)	26.7(11)	53.5(11)	-4.3(9)	-8.9(10)	5.1(10)
N(1)	30.4(11)	26.7(12)	32.1(11)	-1.3(9)	-0.9(9)	2.3(10)
N(2)	29.7(11)	23.5(12)	34.7(11)	-4.8(9)	-2.9(9)	1.7(10)
C(1)	37.2(15)	30.8(15)	34.5(13)	-1.0(11)	-1.7(12)	2.5(13)
C(2)	40.0(15)	32.3(16)	34.4(13)	2.7(12)	1.0(12)	12.0(14)
C(3)	27.3(13)	43.1(17)	28.7(12)	5.0(12)	0.5(11)	4.6(13)
C(4)	25.9(12)	35.8(15)	22.5(11)	2.3(10)	2.9(10)	-1.2(12)
C(5)	25.6(12)	28.6(14)	23.0(11)	-0.5(10)	3.8(10)	-2.4(11)
C(6)	24.8(12)	27.6(14)	26.5(11)	0.4(10)	1.7(10)	0.8(12)
C(7)	36.3(14)	28.5(14)	30.8(12)	-2.3(11)	1.6(11)	-2.5(13)
C(8)	39.7(15)	34.9(16)	31.5(13)	-1.2(11)	-3.1(12)	-9.6(14)
C(9)	30.2(13)	39.3(16)	28.4(12)	3.5(11)	-3.3(11)	-6.3(13)
C(10)	29.4(14)	28.6(15)	31.5(12)	-1.4(11)	2.8(11)	2.7(12)
C(11)	29.8(13)	32.0(15)	33.9(12)	0.7(11)	1.5(11)	2.3(13)
C(12)	58(2)	36.7(18)	47.7(16)	-7.7(13)	-16.9(15)	12.7(16)
C(13)	40.7(16)	39.8(17)	43.3(15)	-2.0(13)	-5.9(13)	9.4(15)
C(14)	34.8(15)	31.7(15)	47.4(16)	2.3(12)	-5.4(13)	0.6(14)
C(15)	28.1(14)	28.6(15)	37.5(13)	1.9(11)	-0.8(11)	3.5(12)
C(16)	35.9(16)	44.8(17)	36.0(14)	3.8(13)	4.8(12)	0.1(15)
C(17)	34.7(15)	42.9(18)	48.2(16)	3.7(14)	2.8(13)	-5.3(14)
C(18)	36.0(15)	28.5(14)	38.7(13)	-3.3(12)	-0.6(12)	6.5(13)
C(19)	43.4(18)	35.3(17)	60.1(18)	-8.7(14)	-11.6(15)	3.4(15)
C(20)	61(2)	48(2)	45.7(17)	-10.8(15)	-19.8(16)	12.2(19)
C(21)	76(2)	42.2(19)	35.6(15)	1.3(13)	-2.7(16)	18(2)
C(22)	52.2(19)	38.2(18)	40.6(15)	5.0(13)	6.1(14)	6.6(16)
C(23)	37.2(15)	24.1(14)	34.9(13)	0.4(11)	1.9(11)	7.0(13)
C(24)	31.0(14)	30.6(15)	42.2(14)	4.1(12)	3.8(12)	1.9(13)

Supplementary Table 10. Bond Lengths for **5a**.

Atom	Atom	Length/Å	Atom	Atom	Length/Å
O(1)	C(10)	1.220(3)	C(11)	C(12)	1.517(4)
N(1)	C(1)	1.316(3)	C(12)	C(13)	1.498(4)
N(1)	C(5)	1.365(3)	C(13)	C(14)	1.312(4)
N(2)	C(6)	1.400(3)	C(14)	C(15)	1.466(3)
N(2)	C(10)	1.366(3)	C(15)	C(16)	1.420(4)
C(1)	C(2)	1.408(4)	C(15)	C(24)	1.376(3)
C(2)	C(3)	1.360(4)	C(16)	C(17)	1.356(4)
C(3)	C(4)	1.411(3)	C(17)	C(18)	1.412(4)
C(4)	C(5)	1.422(3)	C(18)	C(19)	1.422(4)
C(4)	C(9)	1.416(3)	C(18)	C(23)	1.412(4)
C(5)	C(6)	1.426(3)	C(19)	C(20)	1.371(4)
C(6)	C(7)	1.374(3)	C(20)	C(21)	1.390(5)
C(7)	C(8)	1.411(4)	C(21)	C(22)	1.358(4)
C(8)	C(9)	1.353(4)	C(22)	C(23)	1.412(4)
C(10)	C(11)	1.511(4)	C(23)	C(24)	1.421(4)

Supplementary Table 11 Bond Angles for **5a**.

Atom	Atom	Atom	Angle/°	Atom	Atom	Atom	Angle/°
C(1)	N(1)	C(5)	117.6(2)	C(10)	C(11)	C(12)	113.9(2)
C(10)	N(2)	C(6)	128.9(2)	C(13)	C(12)	C(11)	112.1(2)
N(1)	C(1)	C(2)	124.1(3)	C(14)	C(13)	C(12)	124.6(3)
C(3)	C(2)	C(1)	118.5(3)	C(13)	C(14)	C(15)	128.0(3)
C(2)	C(3)	C(4)	120.3(2)	C(16)	C(15)	C(14)	122.4(2)
C(3)	C(4)	C(5)	116.6(2)	C(24)	C(15)	C(14)	119.7(2)
C(3)	C(4)	C(9)	124.4(2)	C(24)	C(15)	C(16)	117.8(2)
C(9)	C(4)	C(5)	118.9(2)	C(17)	C(16)	C(15)	121.7(2)
N(1)	C(5)	C(4)	122.8(2)	C(16)	C(17)	C(18)	121.1(3)
N(1)	C(5)	C(6)	117.7(2)	C(17)	C(18)	C(19)	122.0(3)
C(4)	C(5)	C(6)	119.5(2)	C(23)	C(18)	C(17)	118.8(2)
N(2)	C(6)	C(5)	114.9(2)	C(23)	C(18)	C(19)	119.2(2)
C(7)	C(6)	N(2)	125.5(2)	C(20)	C(19)	C(18)	120.1(3)
C(7)	C(6)	C(5)	119.6(2)	C(19)	C(20)	C(21)	120.1(3)
C(6)	C(7)	C(8)	119.9(3)	C(22)	C(21)	C(20)	121.3(3)
C(9)	C(8)	C(7)	121.8(2)	C(21)	C(22)	C(23)	120.5(3)
C(8)	C(9)	C(4)	120.1(2)	C(18)	C(23)	C(22)	118.7(3)
O(1)	C(10)	N(2)	123.0(2)	C(18)	C(23)	C(24)	118.7(2)

O(1)	C(10)	C(11)	123.7(2)	C(22)	C(23)	C(24)	122.6(3)
N(2)	C(10)	C(11)	113.3(2)	C(15)	C(24)	C(23)	122.0(2)

Supplementary Table 12. Torsion Angles for **5a**.

**A**	**B**	**C**	**D**	**Angle/°**	**A**	**B**	**C**	**D**	**Angle/°**
O(1)	C(10)	C(11)	C(12)	-7.1(4)	C(10)	N(2)	C(6)	C(7)	-4.3(4)
N(1)	C(1)	C(2)	C(3)	-2.4(4)	C(10)	C(11)	C(12)	C(13)	-177.4(2)
N(1)	C(5)	C(6)	N(2)	4.2(3)	C(11)	C(12)	C(13)	C(14)	112.7(3)
N(1)	C(5)	C(6)	C(7)	-177.5(2)	C(12)	C(13)	C(14)	C(15)	-178.4(3)
N(2)	C(6)	C(7)	C(8)	176.2(2)	C(13)	C(14)	C(15)	C(16)	-2.0(5)
N(2)	C(10)	C(11)	C(12)	172.6(2)	C(13)	C(14)	C(15)	C(24)	179.0(3)
C(1)	N(1)	C(5)	C(4)	0.8(3)	C(14)	C(15)	C(16)	C(17)	-178.3(3)
C(1)	N(1)	C(5)	C(6)	-177.9(2)	C(14)	C(15)	C(24)	C(23)	179.0(2)
C(1)	C(2)	C(3)	C(4)	0.7(4)	C(15)	C(16)	C(17)	C(18)	-0.5(4)
C(2)	C(3)	C(4)	C(5)	1.5(3)	C(16)	C(15)	C(24)	C(23)	0.0(4)
C(2)	C(3)	C(4)	C(9)	-179.2(2)	C(16)	C(17)	C(18)	C(19)	179.3(3)
C(3)	C(4)	C(5)	N(1)	-2.4(3)	C(16)	C(17)	C(18)	C(23)	-0.4(4)
C(3)	C(4)	C(5)	C(6)	176.4(2)	C(17)	C(18)	C(19)	C(20)	-179.8(3)
C(3)	C(4)	C(9)	C(8)	-178.8(2)	C(17)	C(18)	C(23)	C(22)	-179.4(3)
C(4)	C(5)	C(6)	N(2)	-174.6(2)	C(17)	C(18)	C(23)	C(24)	1.1(4)
C(4)	C(5)	C(6)	C(7)	3.7(3)	C(18)	C(19)	C(20)	C(21)	-0.9(5)
C(5)	N(1)	C(1)	C(2)	1.6(4)	C(18)	C(23)	C(24)	C(15)	-0.9(4)
C(5)	C(4)	C(9)	C(8)	0.4(3)	C(19)	C(18)	C(23)	C(22)	0.8(4)
C(5)	C(6)	C(7)	C(8)	-2.0(4)	C(19)	C(18)	C(23)	C(24)	-178.6(2)
C(6)	N(2)	C(10)	O(1)	2.5(4)	C(19)	C(20)	C(21)	C(22)	1.2(5)
C(6)	N(2)	C(10)	C(11)	-177.2(2)	C(20)	C(21)	C(22)	C(23)	-0.5(5)
C(6)	C(7)	C(8)	C(9)	-0.6(4)	C(21)	C(22)	C(23)	C(18)	-0.5(4)
C(7)	C(8)	C(9)	C(4)	1.4(4)	C(21)	C(22)	C(23)	C(24)	178.9(3)
C(9)	C(4)	C(5)	N(1)	178.3(2)	C(22)	C(23)	C(24)	C(15)	179.6(3)
C(9)	C(4)	C(5)	C(6)	-2.9(3)	C(23)	C(18)	C(19)	C(20)	-0.1(4)
C(10)	N(2)	C(6)	C(5)	174.0(2)	C(24)	C(15)	C(16)	C(17)	0.7(4)

Supplementary Table 13. Hydrogen Atom Coordinates (Å × 10^4) and Isotropic Displacement Parameters (Å^2 × 10^3) for **5a**.

**Atom**	***x***	***y***	***z***	**U(eq)**
H(2)	3750(50)	6180(20)	940(6)	35
H(1)	1111.28	10368.67	861.27	41
H(2A)	-2400.5	10750.04	576.94	43
H(3)	-4064.81	8606.59	275.33	40

H(7)	1434.73	2941.54	447.39	38
H(8)	-1922.59	3222.38	110.88	42
H(9)	-3838.13	5628.76	84.31	39
H(11A)	5976.58	5005.44	1403.86	38
H(11B)	7757.52	5075.87	1080.48	38
H(12A)	6778.48	2200.26	1475.32	57
H(12B)	8631.33	2323.7	1161.98	57
H(13)	10949.79	3998.47	1541.71	50
H(14)	8277.17	2493.54	2047.48	46
H(16)	13527.33	4745.95	1935.11	47
H(17)	16079.74	5384.81	2375.2	50
H(19)	17267.54	5317.15	3018.63	55
H(20)	16508.92	4542.91	3603.38	62
H(21)	13122.52	3215.19	3742.13	61
H(22)	10539.04	2559.92	3304.24	52
H(24)	9420.65	2559.79	2656.4	42

Crystal structure determination of 5a

Crystal Data for $C_{24}H_{20}N_2O$ (M =352.42 g mol^{-1}): orthorhombic, space group $P2_12_12_1$ (no. 19), a = 5.8681(2) Å, b = 8.2806(4) Å, c = 37.3551(18) Å, V = 1815.12(14) Å^3, Z = 4, T = 113.15 K, μ (MoK α) = 0.079 mm^{-1}, $Dcalc$ = 1.290 g cm^{-3}, 15887 reflections measured (4.362° ≤ 2Θ ≤ 52.738°), 3694 unique (R_{int} = 0.0676, R_{sigma} = 0.0518) which were used in all calculations. The final R_1 was 0.0461 (I > 2 σ (I)) and wR_2 was 0.1000 (all data).

DFT Calculations

All density functional theory (DFT) calculations were conducted with the Gaussian 16 software package.[10] Geometries were optimized at the M06/6-31G(d)-Lanl2DZ (Ni,Cs) level of theory with the SMD solvation model in 2-methyl-2-propanol. Based on the optimized structures,[11-13] vibrational frequencies were calculated at the same level of theory to evaluate its zero-point vibrational energy (ZPVE) and thermal corrections at 343.15 K. Saddle points were connected to minima in the usual way with intrinsic reaction coordinate (IRC) calculations. The single-point energies were computed with ωB97XD functional[14] and 6-311++G(2df,2p) basis set for C,H,O,N,P and def2-TZVPP[15] for Ni and Cs including solvation energy which were evaluated by a self-consistent reaction field (SCRF) using SMD model. The 3D diagrams of

molecules were generated using CYLView.[16] The interaction between Cs^+ and AQ directing group has been presented in literature.[17]

Comparison of the different oxidative addition processes

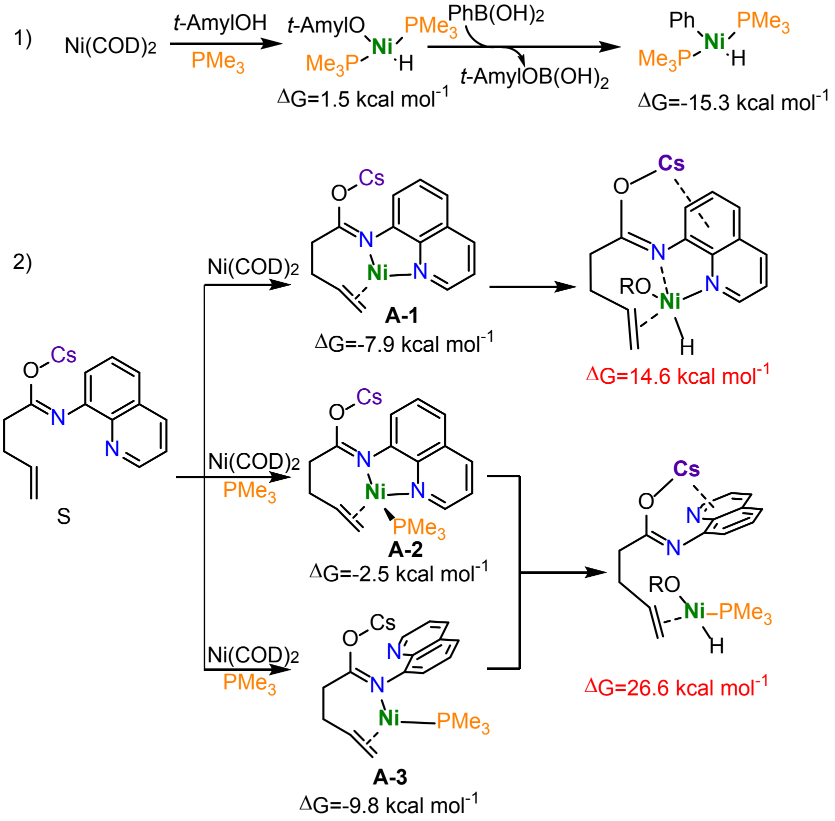

Supplementary Figure 21. DFT-computed free energy profile for the different oxidative addition processes under our reaction condition.

Hydroarylation cycle

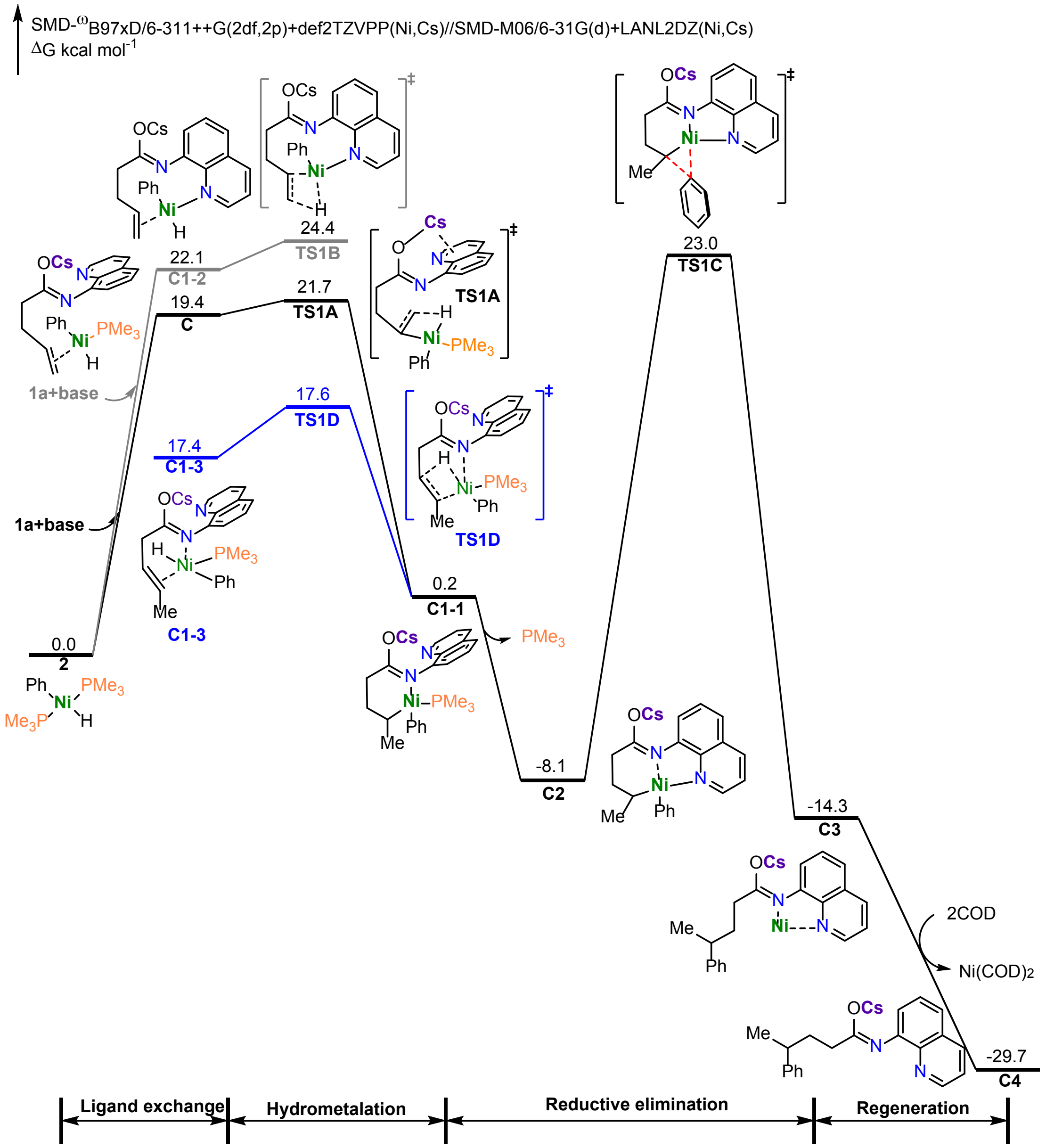

Supplementary Figure 22 DFT-computed free energy profile for the Ni-catalyzed hydroarylation of arylboronic acids and electronically unbiased olefins.

Oxidative Heck cycle

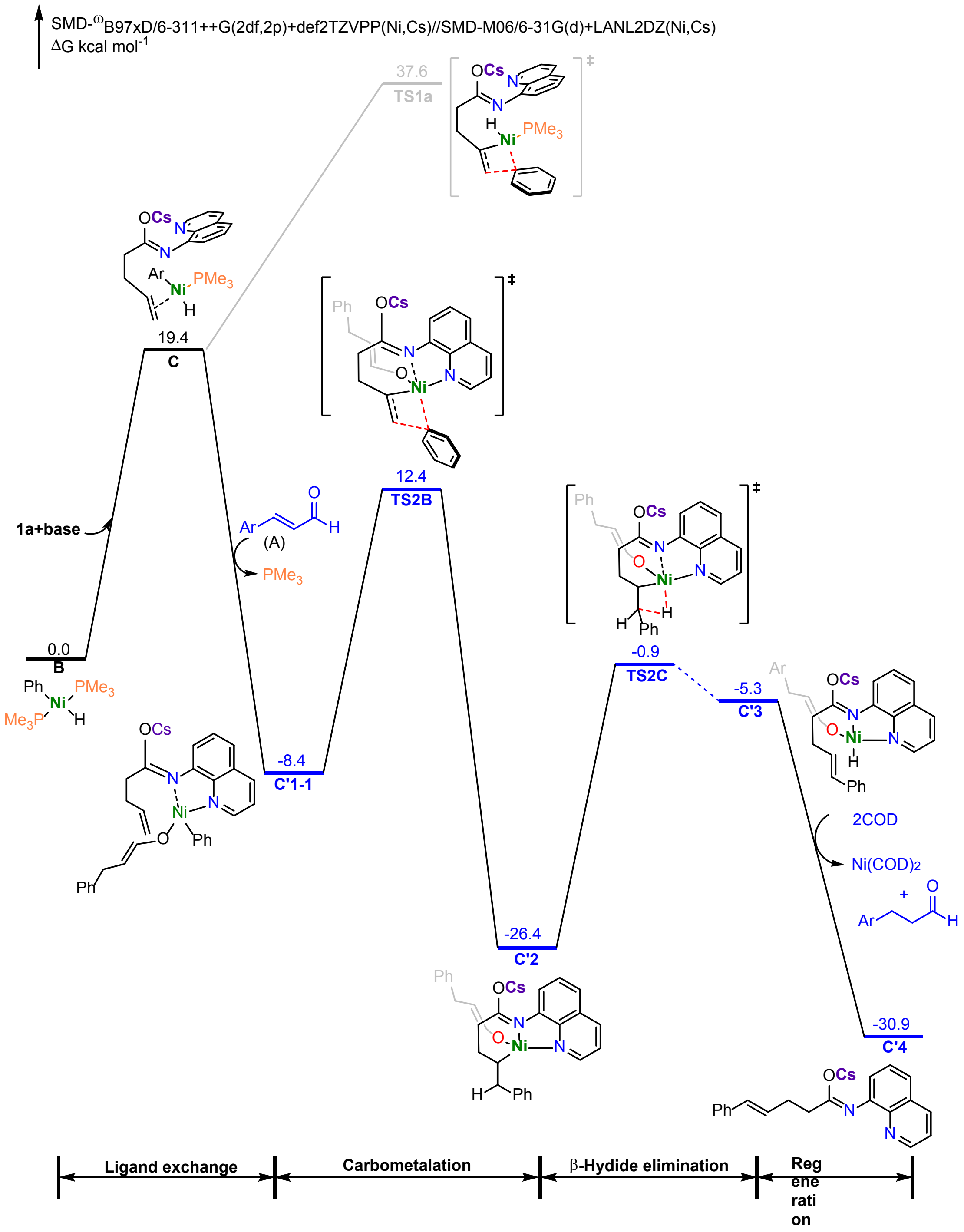

Supplementary Figure 23 DFT-computed free energy profile for the Ni-catalyzed oxidative heck coupling of arylboronic acids and electronically unbiased olefins.

Supplementary Figure 24 ^{1}H NMR (400 MHz, $CDCl_3$) spectrum for synthetic intermediate

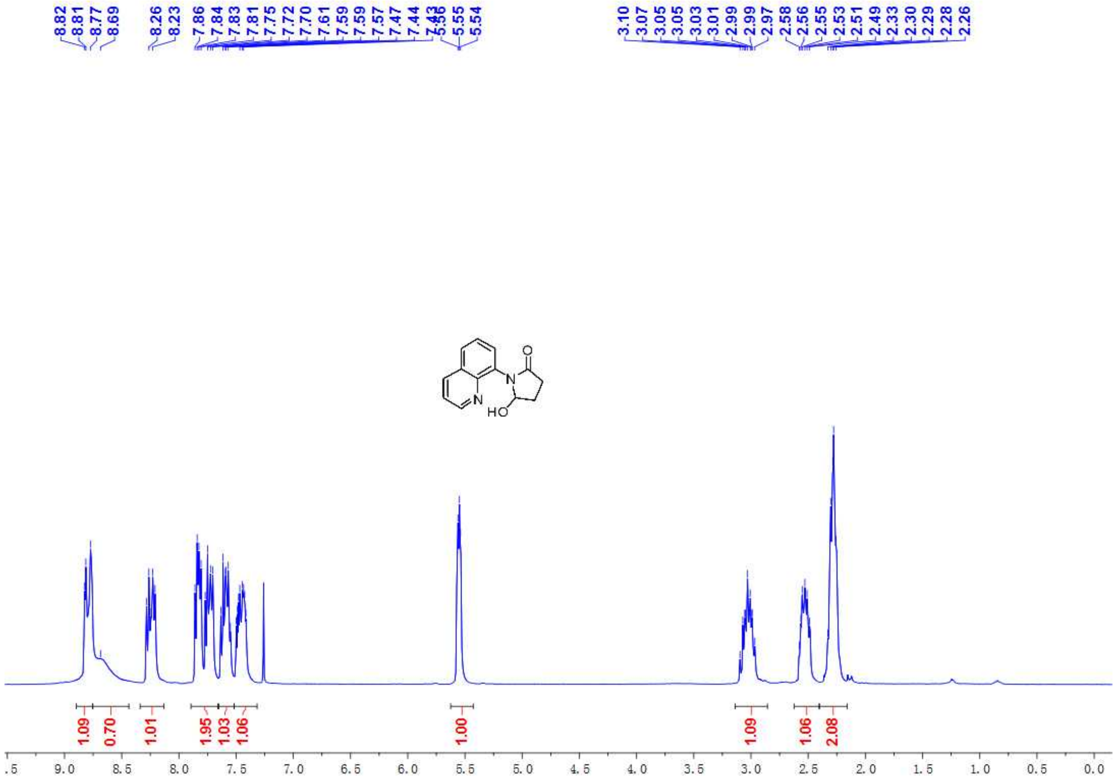

Supplementary Figure 25 ^{13}C NMR (100 MHz, $CDCl_3$) spectrum for synthetic intermediate

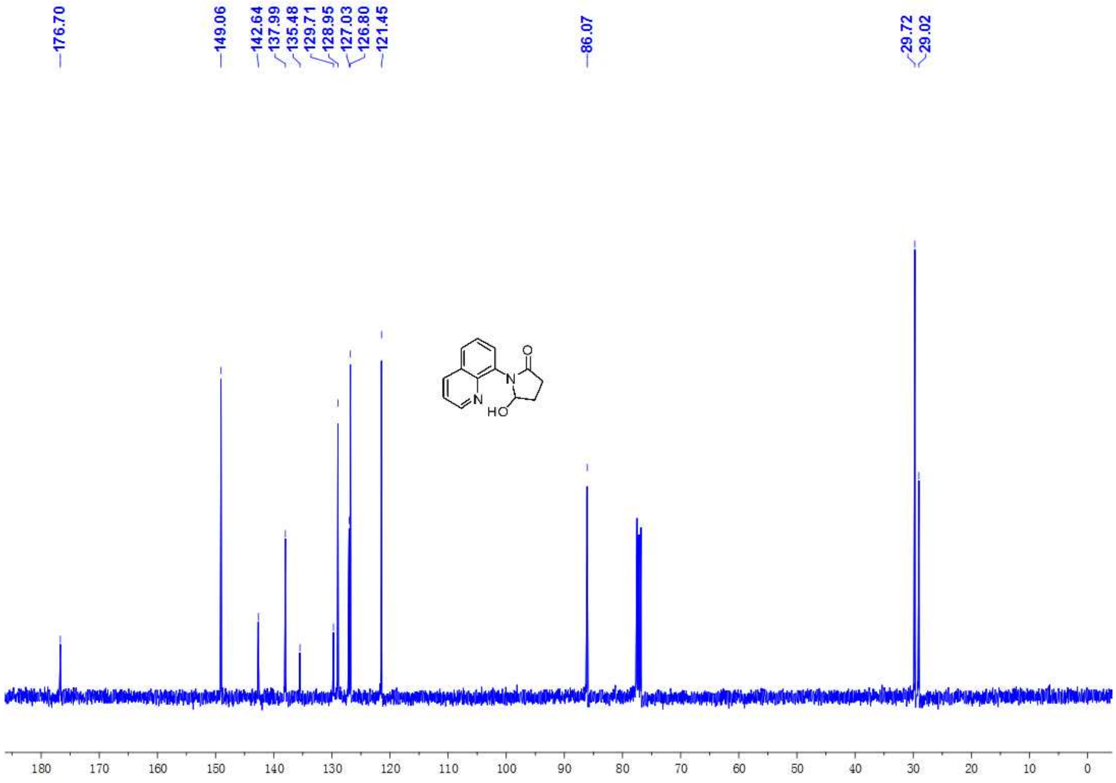

Supplementary Figure 26 ^{1}H NMR (400 MHz, $CDCl_3$) spectrum for 1t

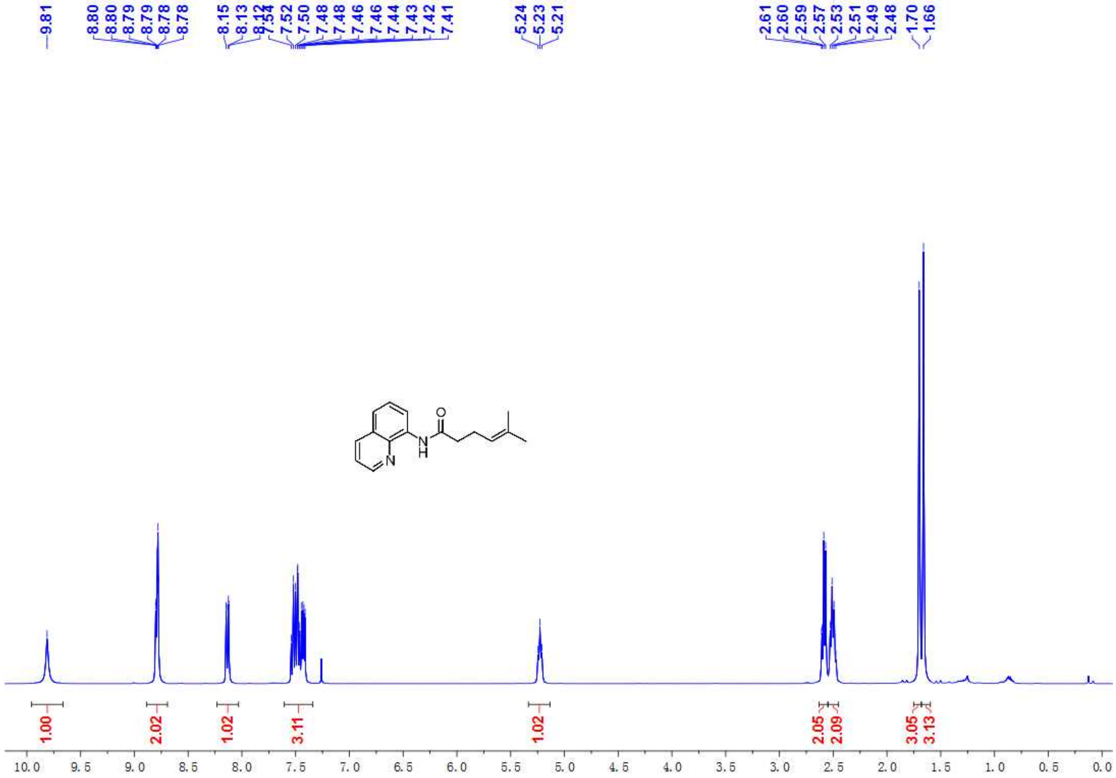

Supplementary Figure 27 ^{13}C NMR (100 MHz, $CDCl_3$) spectrum for 1t

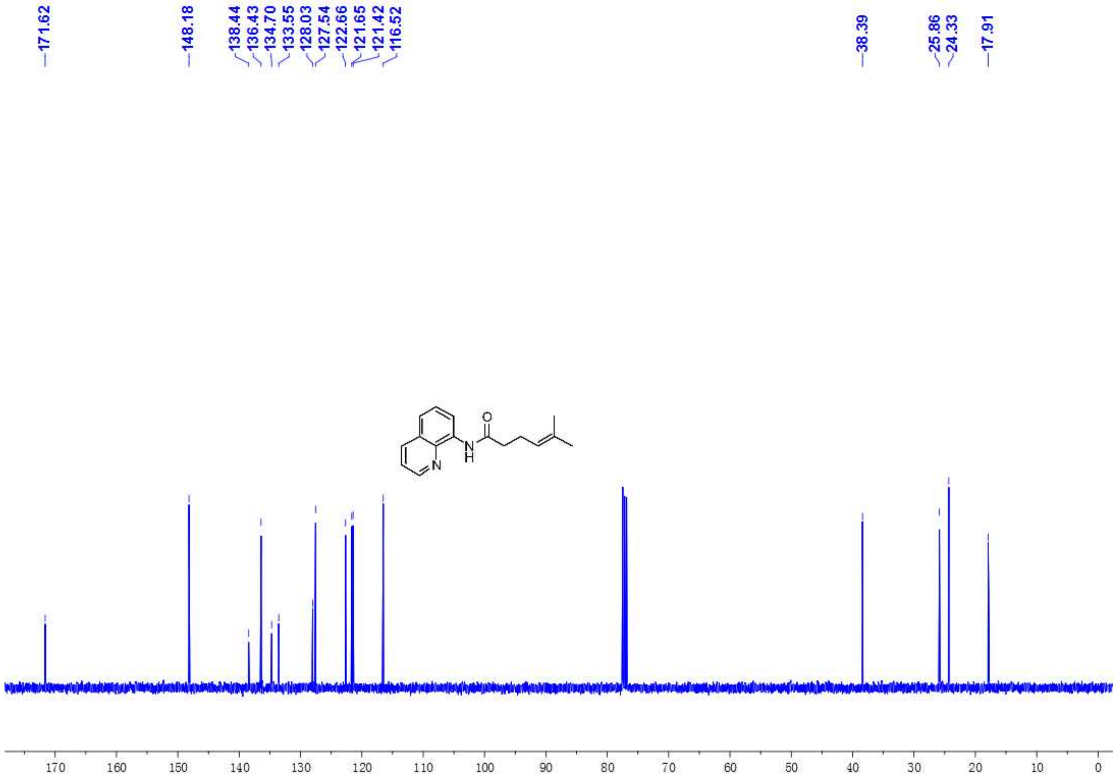

Supplementary Figure 28 ^{1}H NMR (400 MHz, $CDCl_3$) spectrum for 4a

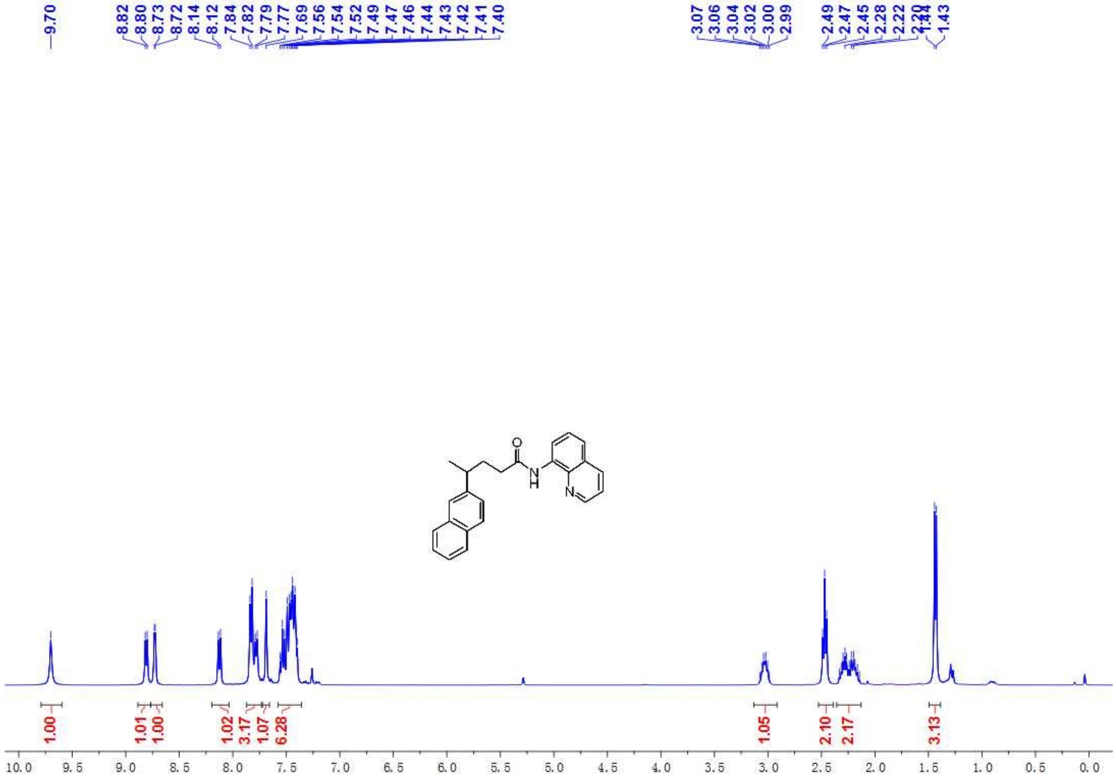

Supplementary Figure 29 ^{13}C NMR (100 MHz, $CDCl_3$) spectrum for 4a

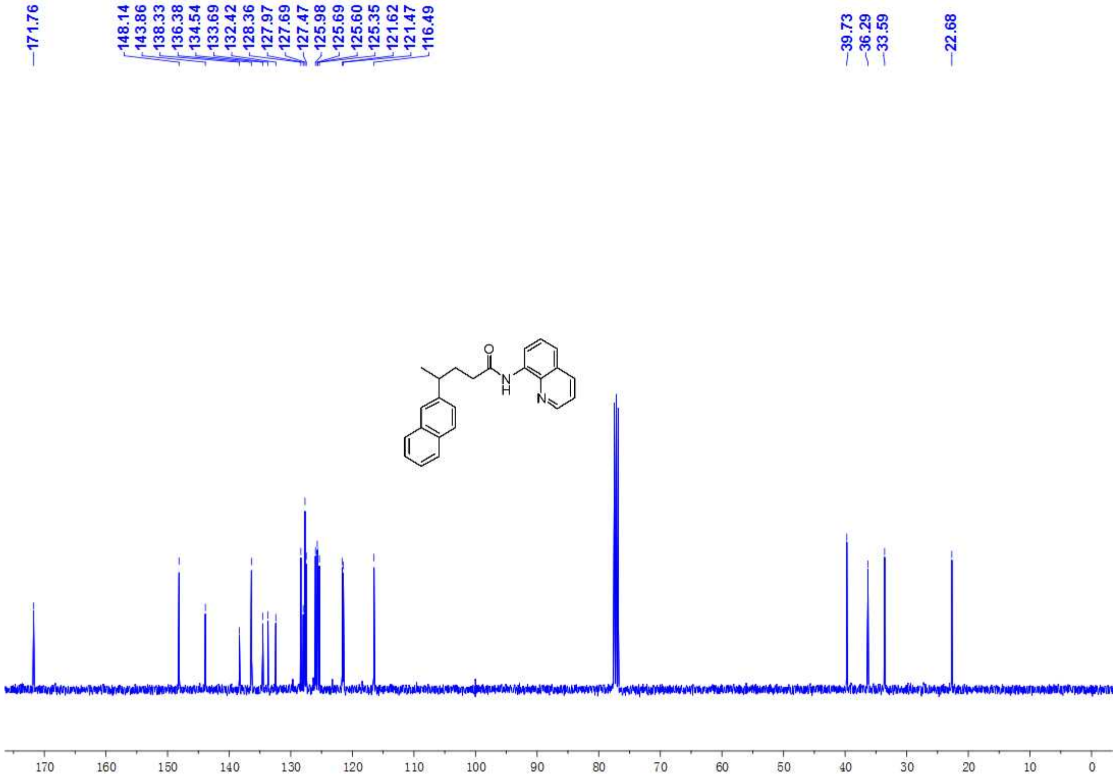

Supplementary **Figure 30 ^{1}H NMR (400 MHz, $CDCl_3$) spectrum for 4b**

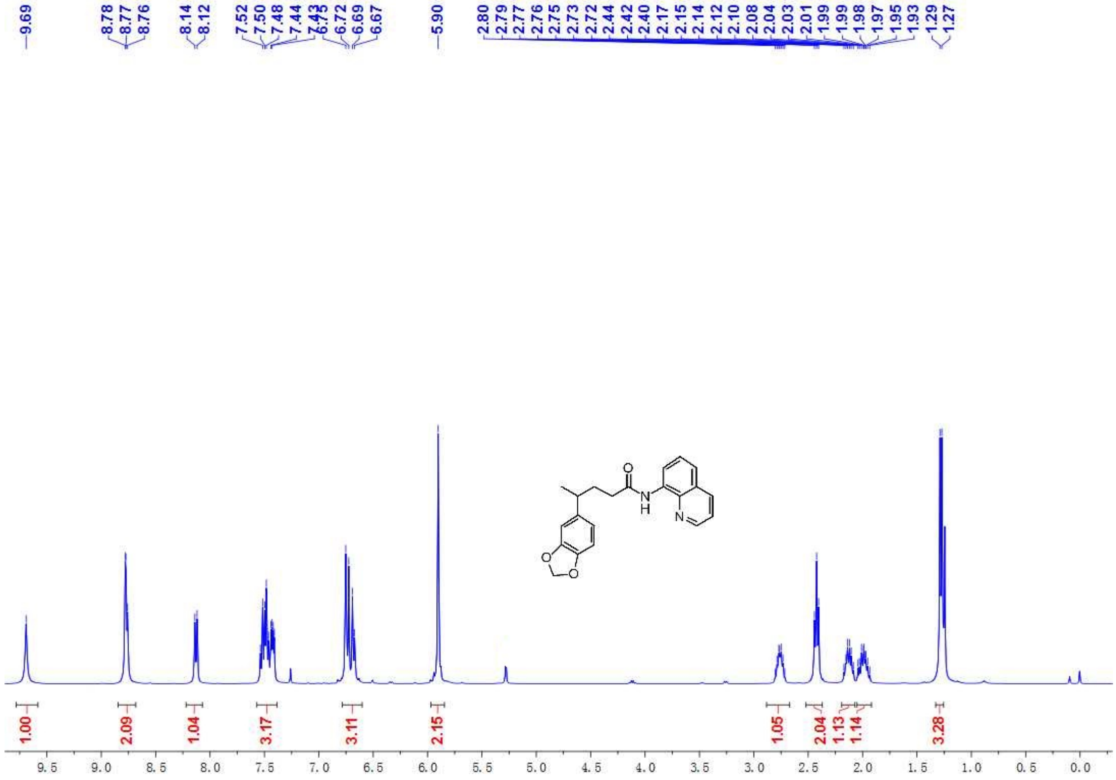

Supplementary **Figure 31 ^{13}C NMR (100 MHz, $CDCl_3$) spectrum for 4b**

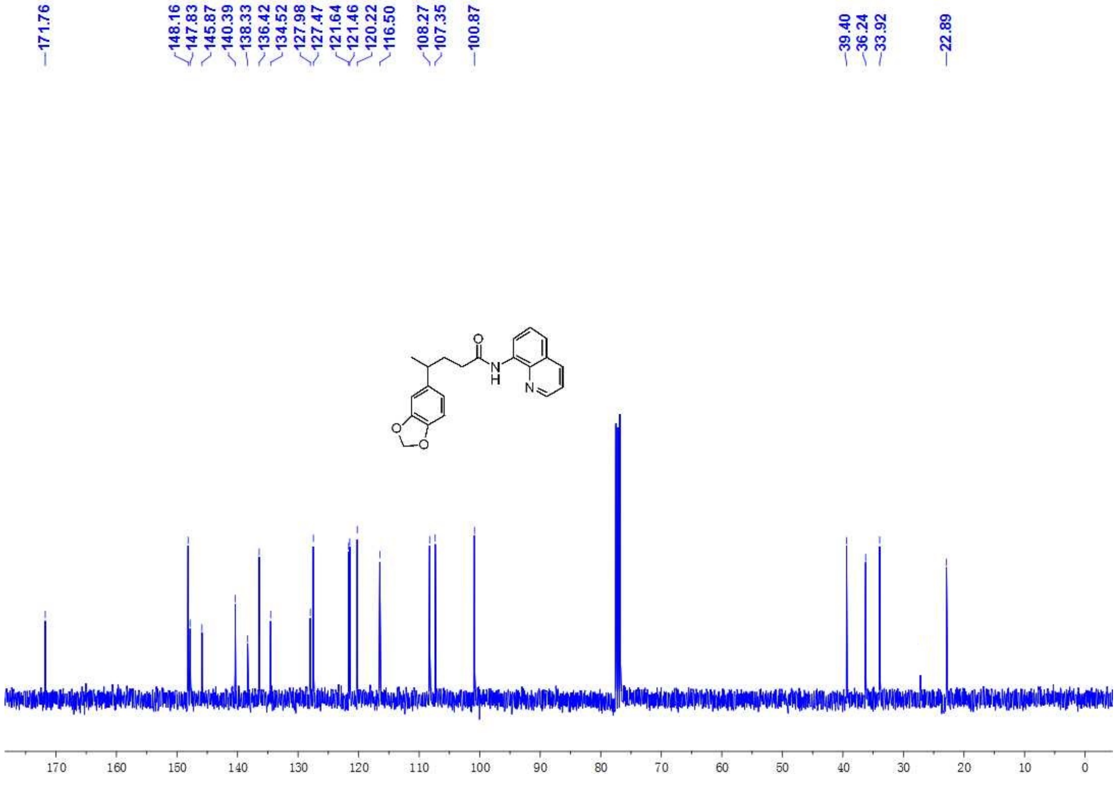

Supplementary Figure 32 ^{1}H NMR (400 MHz, $CDCl_3$) spectrum for 4c

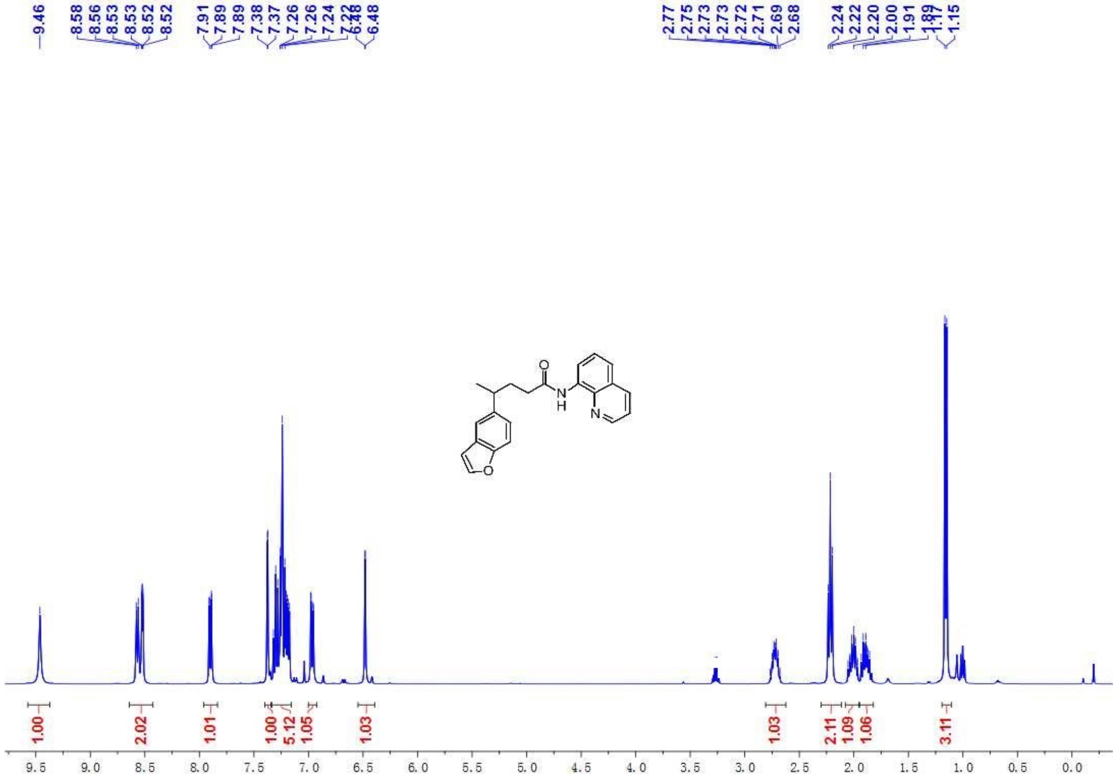

Supplementary Figure 33 ^{13}C NMR (100 MHz, $CDCl_3$) spectrum for 4c

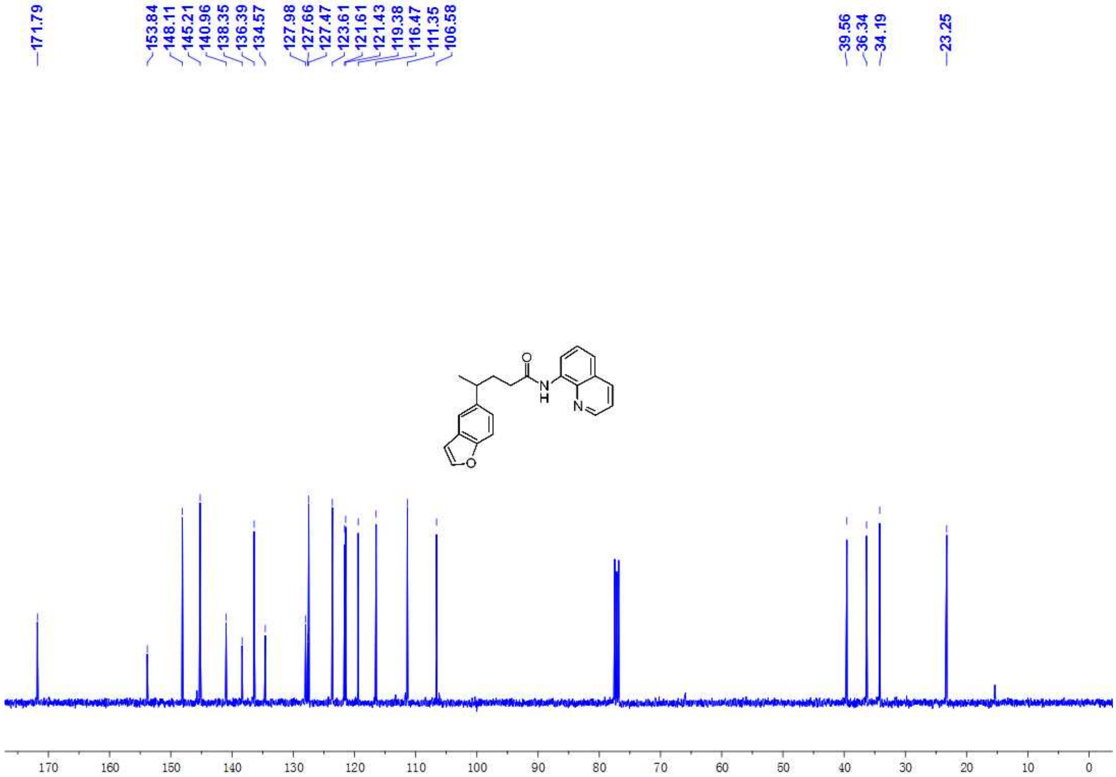

Supplementary Figure 34 ^{1}H NMR (400 MHz, $CDCl_3$) spectrum for 4d

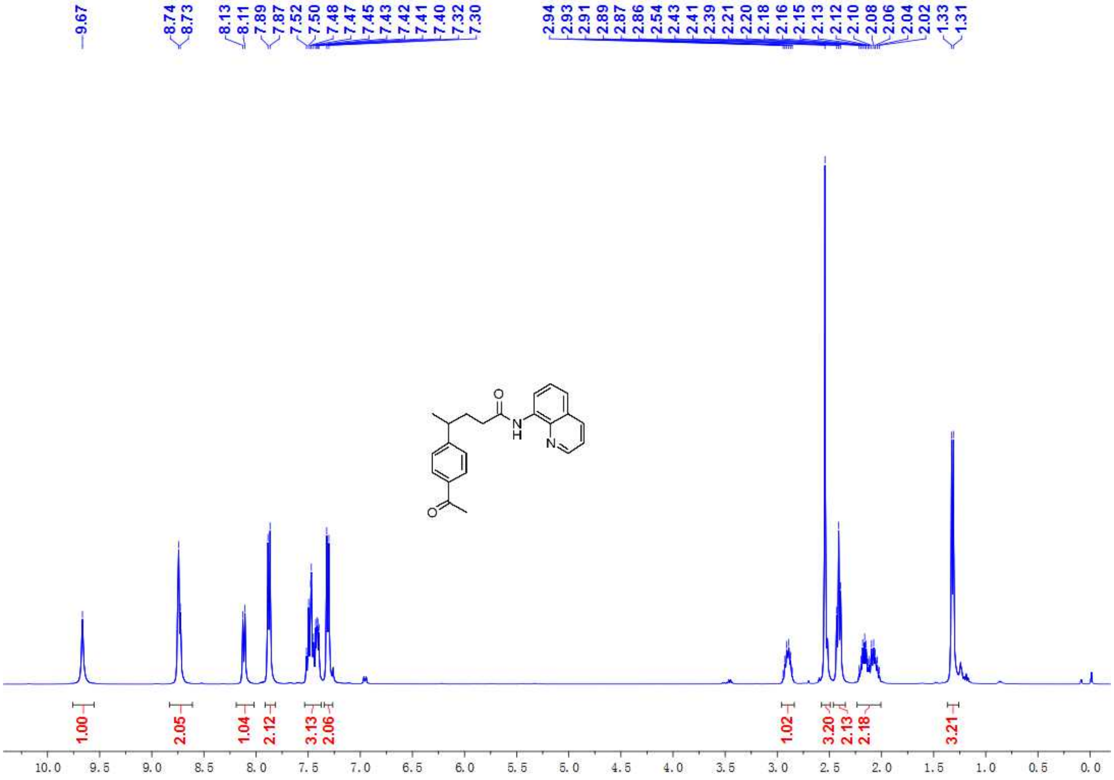

Supplementary Figure 35 ^{13}C NMR (100 MHz, $CDCl_3$) spectrum for 4d

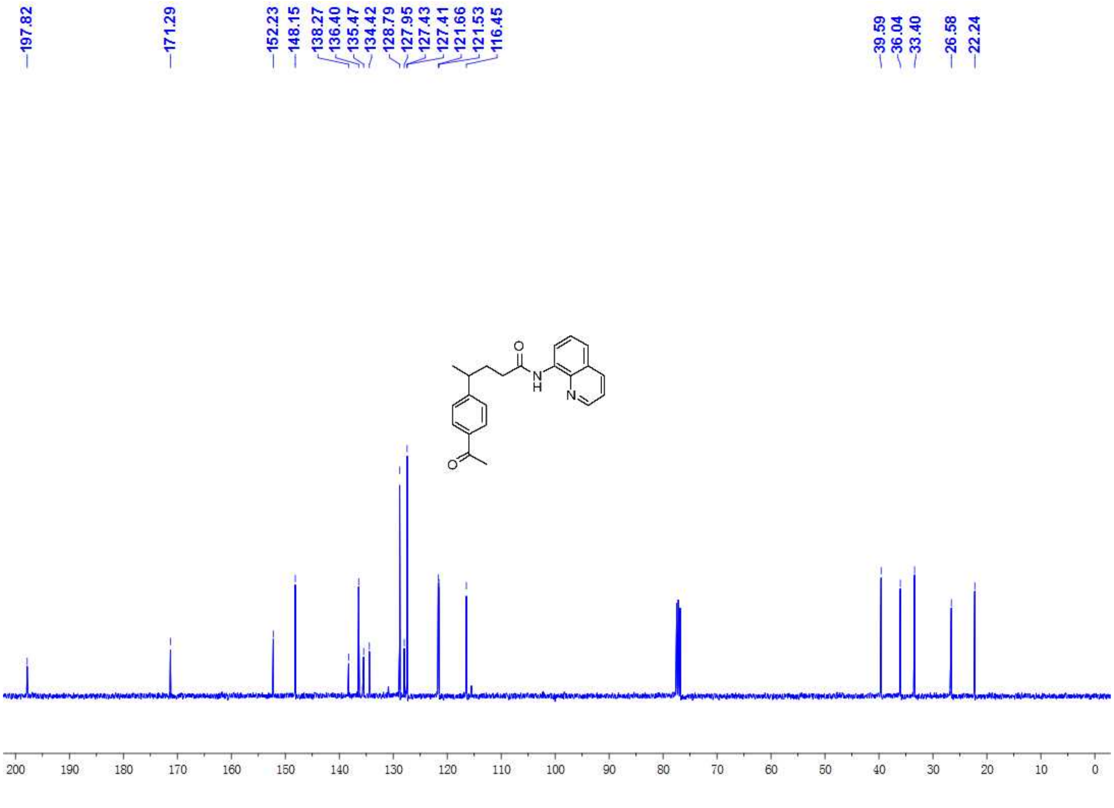

Supplementary Figure 36 ^{1}H NMR (400 MHz, $CDCl_3$) spectrum for 4e

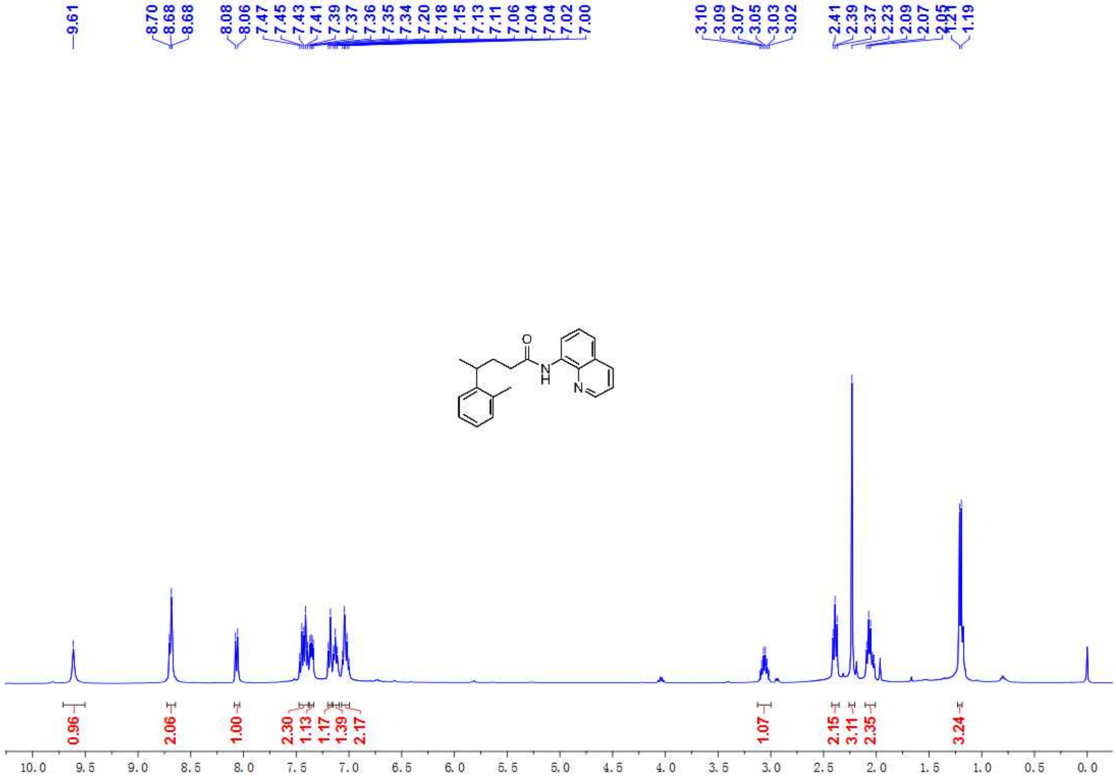

Supplementary Figure 37 ^{13}C NMR (100 MHz, $CDCl_3$) spectrum for 4e

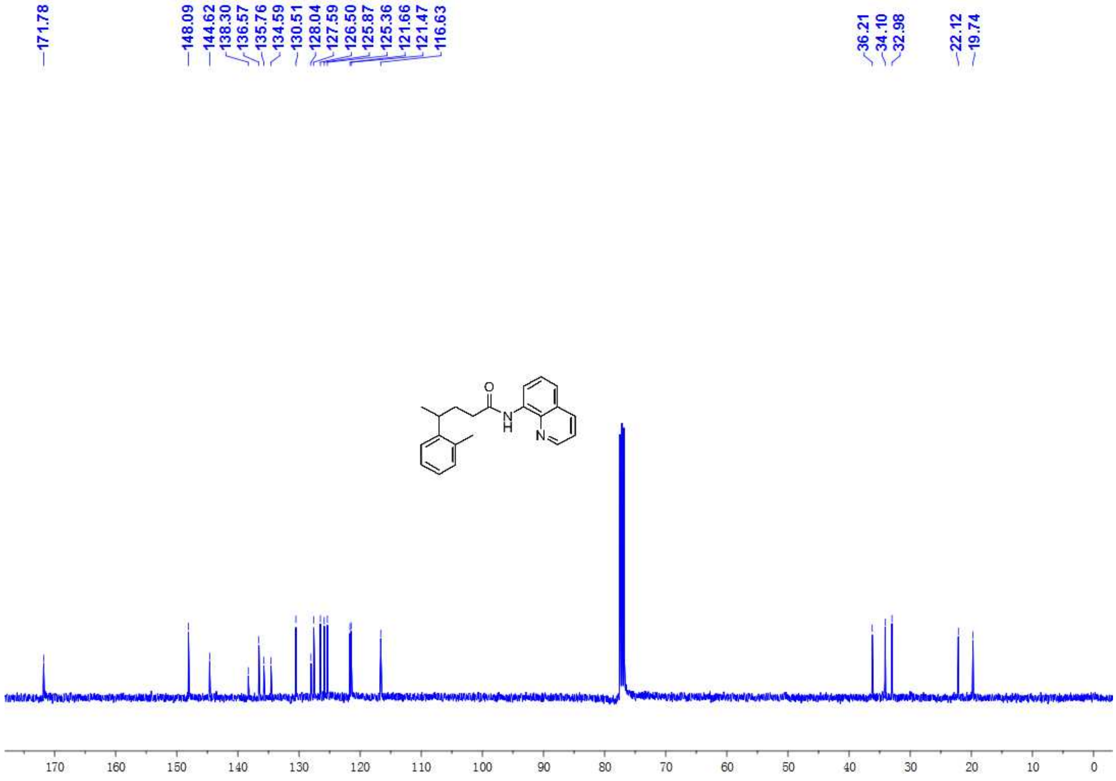

Supplementary Figure 38 ^{1}H NMR (400 MHz, $CDCl_3$) spectrum for 4f

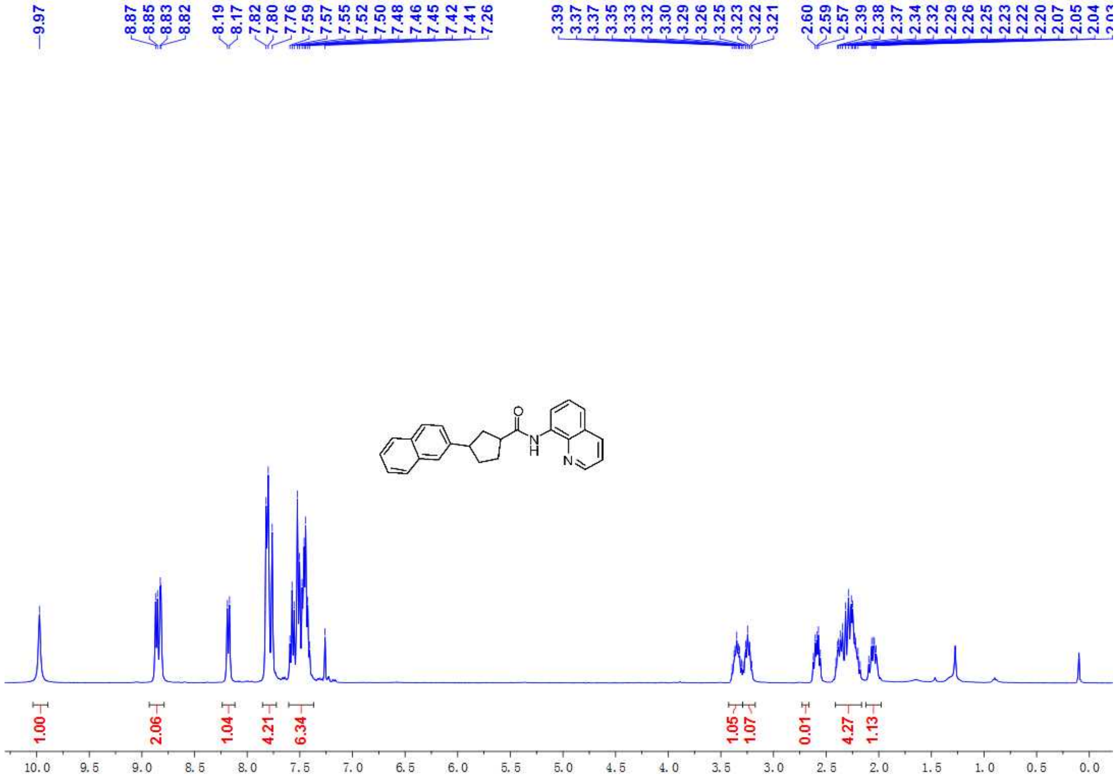

Supplementary Figure 39 ^{13}C NMR (100 MHz, $CDCl_3$) spectrum for 4f

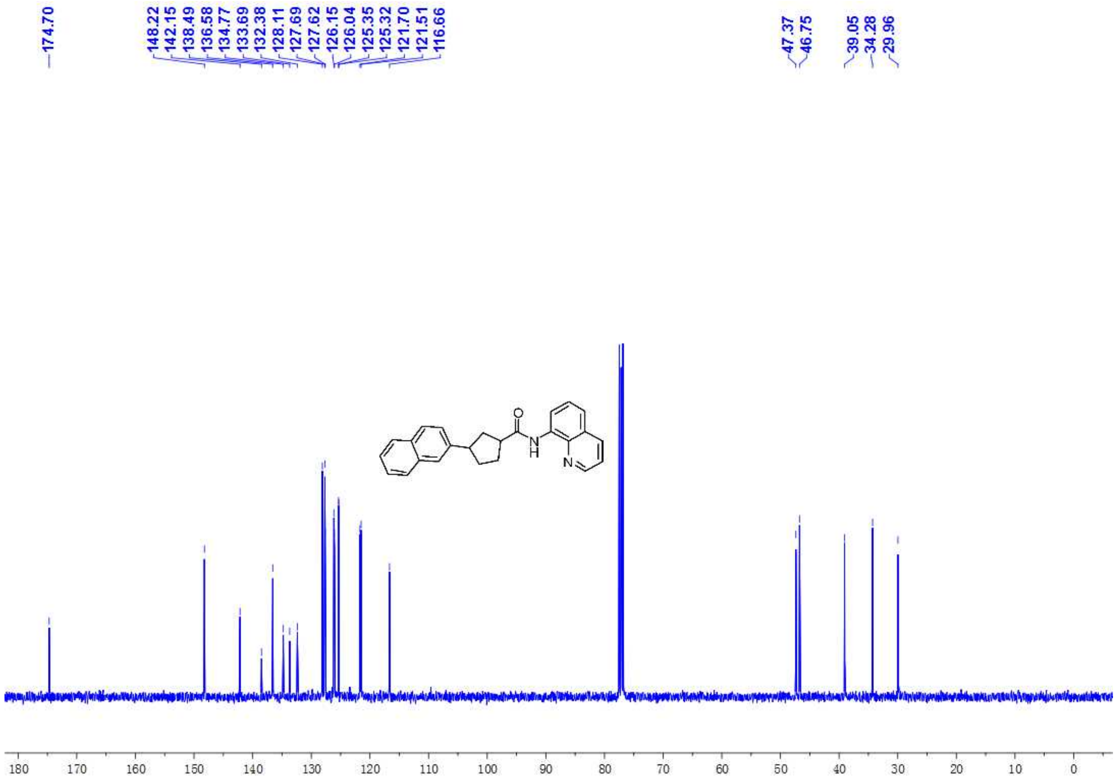

Supplementary Figure 40 ^{1}H NMR (400 MHz, $CDCl_3$) spectrum for 4g

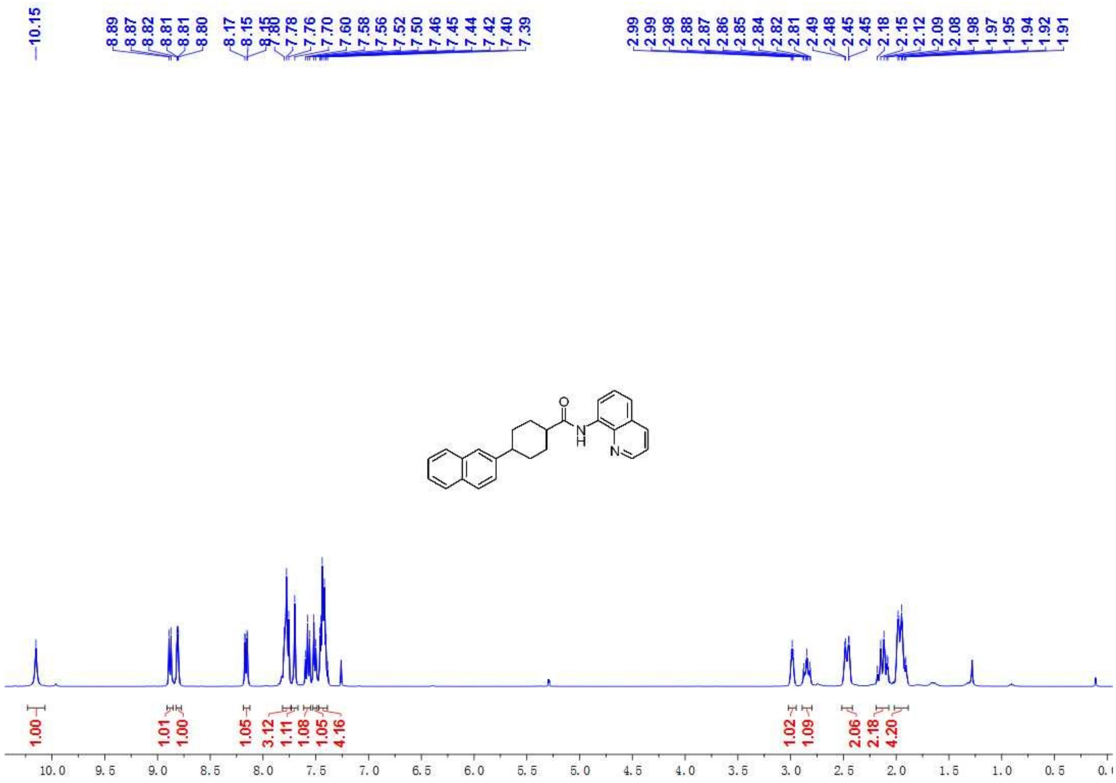

Supplementary Figure 41 ^{13}C NMR (100 MHz, $CDCl_3$) spectrum for 4g

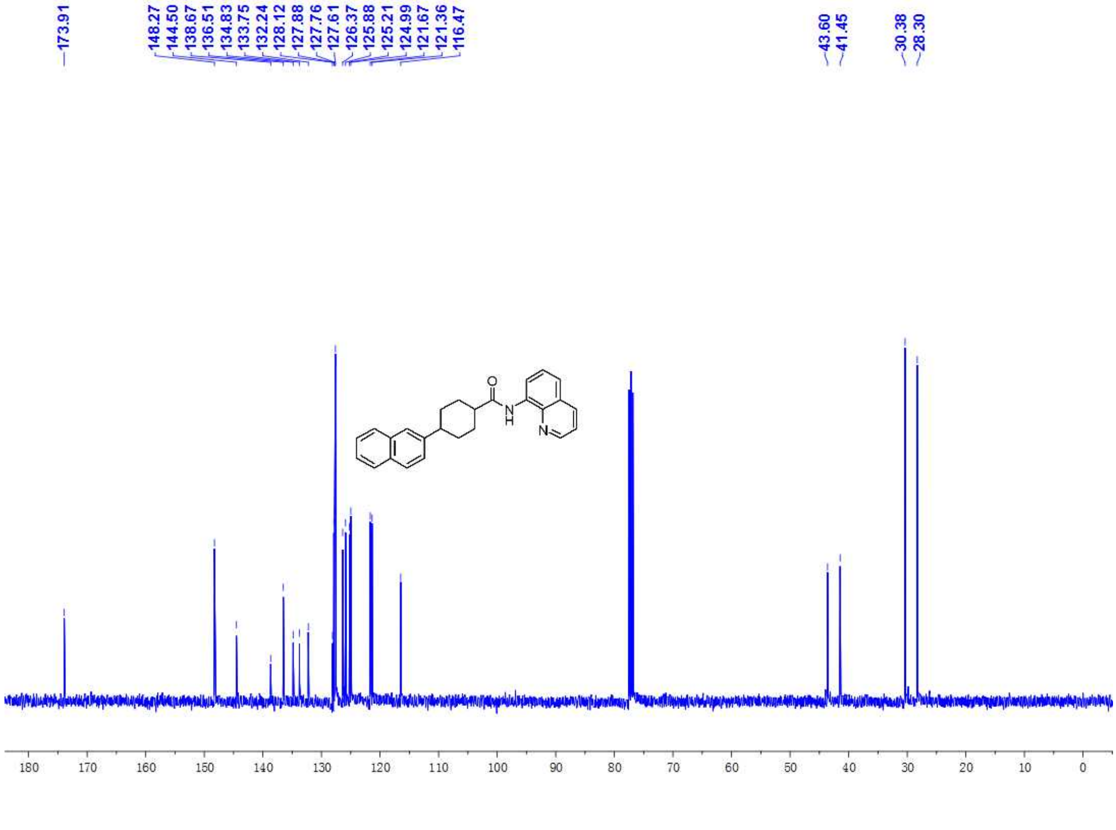

Supplementary Figure 42 ^{1}H NMR (400 MHz, $CDCl_3$) spectrum for 4h

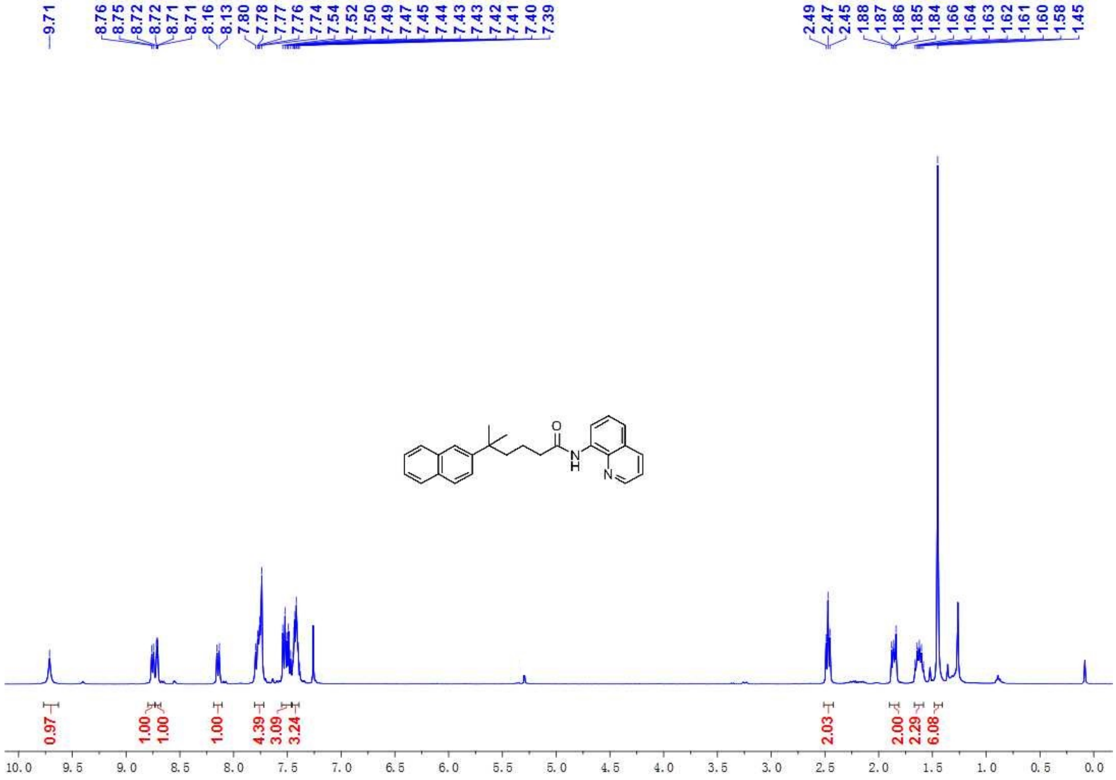

Supplementary Figure 43 ^{13}C NMR (100 MHz, $CDCl_3$) spectrum for 4h

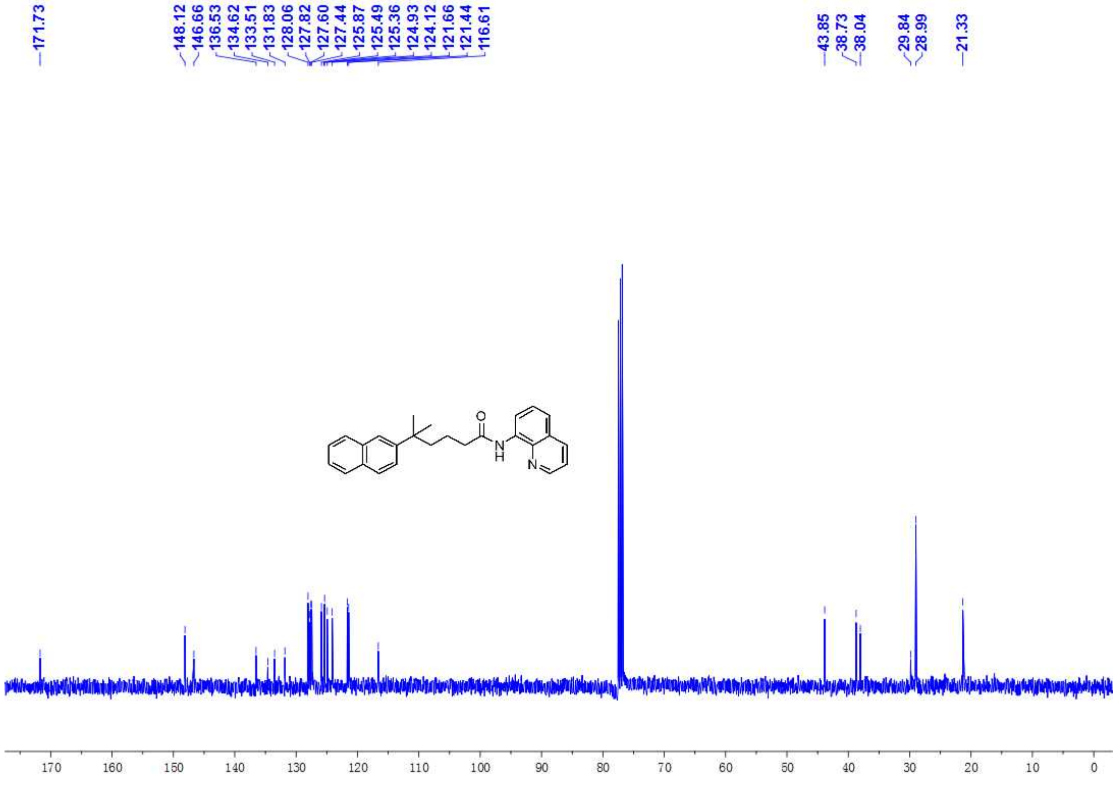

Supplementary Figure 44 ^{1}H NMR (400 MHz, $CDCl_3$) spectrum for 4i

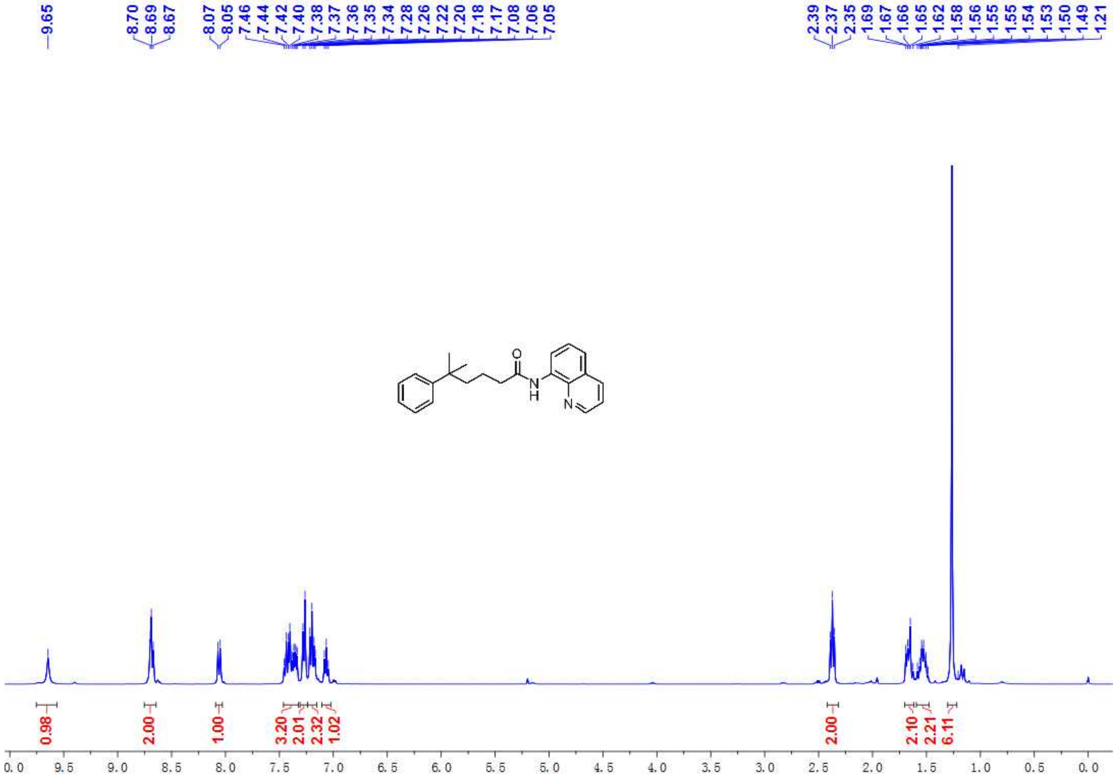

Supplementary Figure 45 ^{13}C NMR (100 MHz, $CDCl_3$) spectrum for 4i

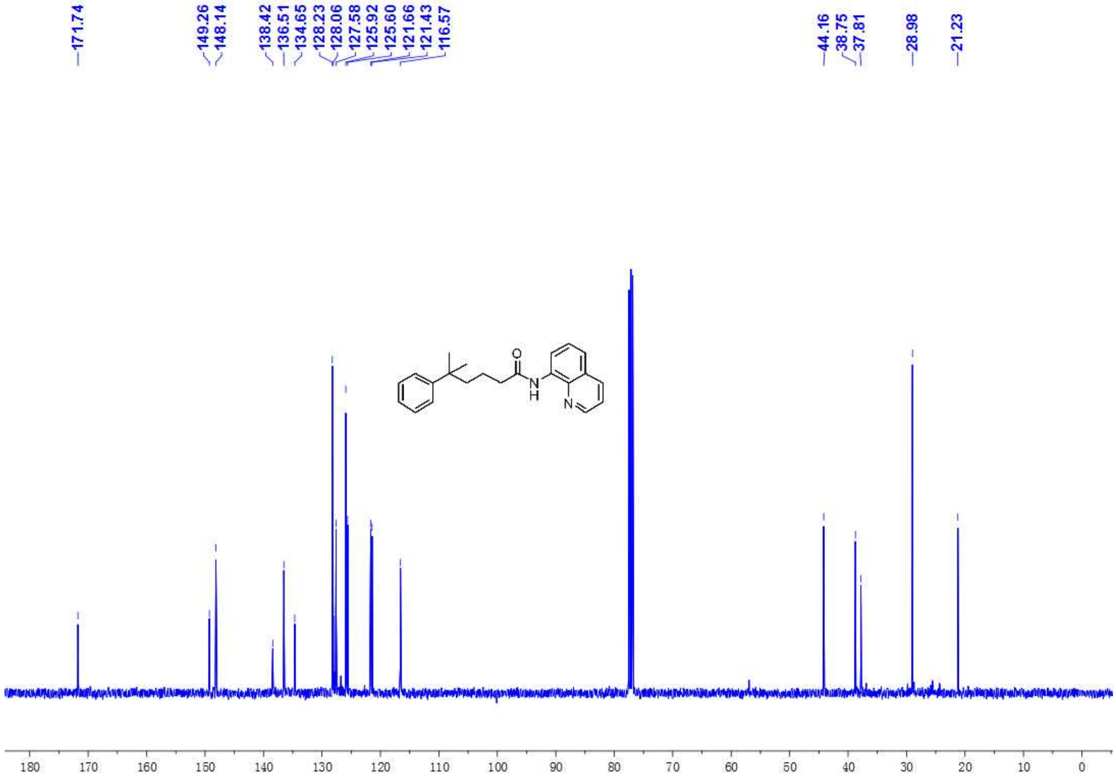

Supplementary Figure 46 ^{1}H NMR (400 MHz, $CDCl_3$) spectrum for 5a

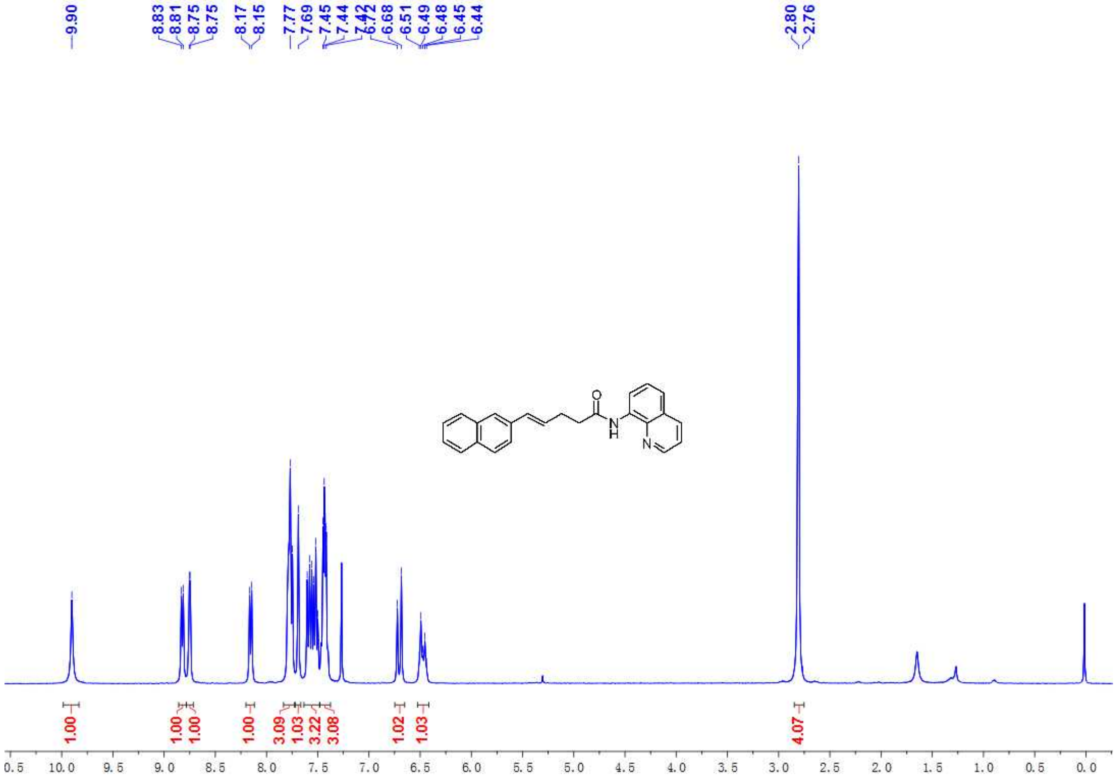

Supplementary Figure 47 ^{13}C NMR (100 MHz, $CDCl_3$) spectrum for 5a

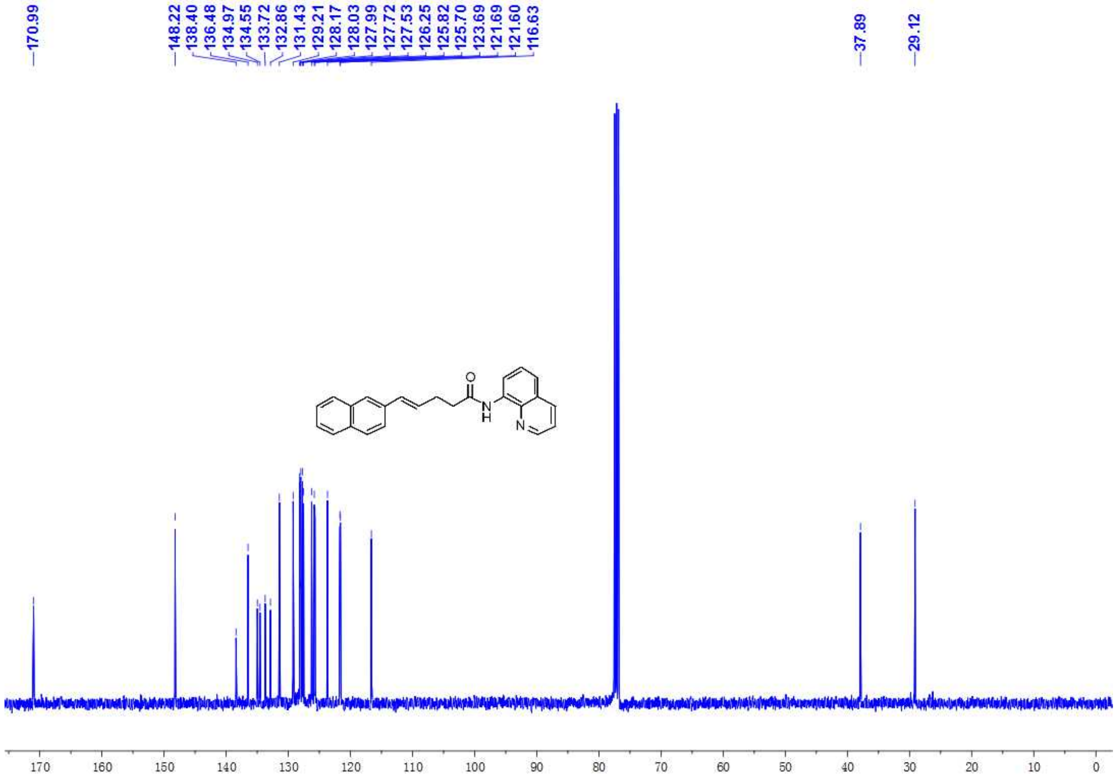

Supplementary Figure 48 ^{1}H NMR (400 MHz, $CDCl_3$) spectrum for 5ab

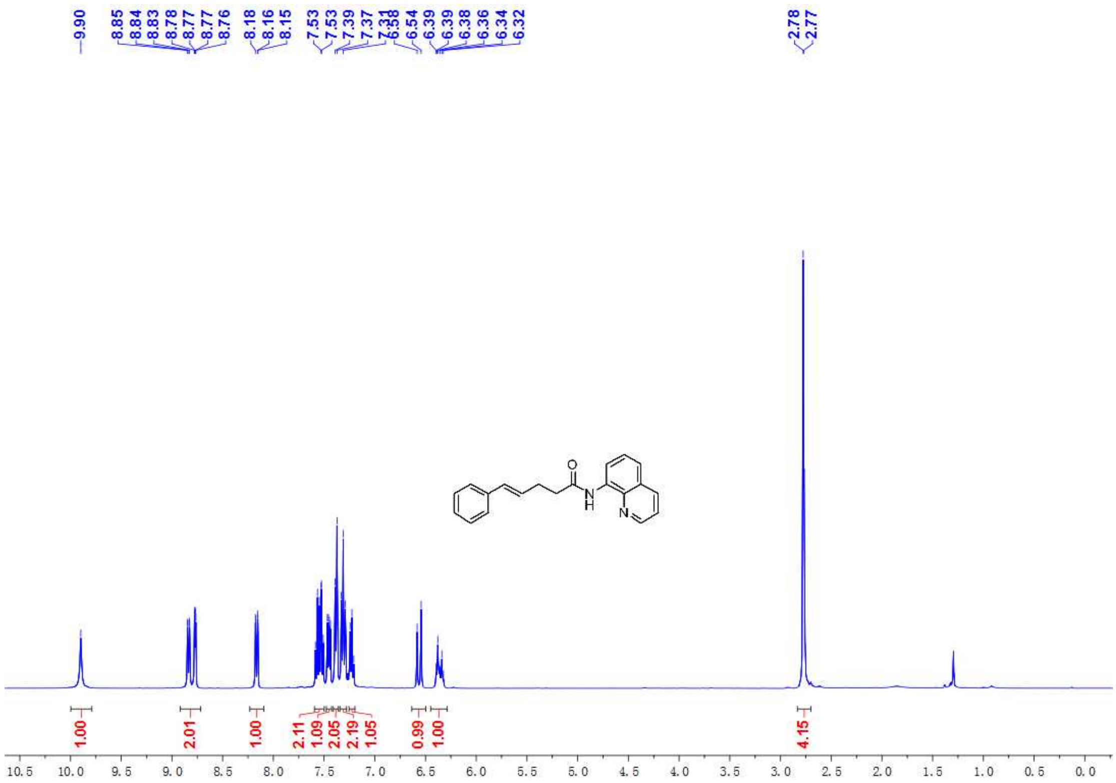

Supplementary Figure 49 ^{13}C NMR (100 MHz, $CDCl_3$) spectrum for 5ab

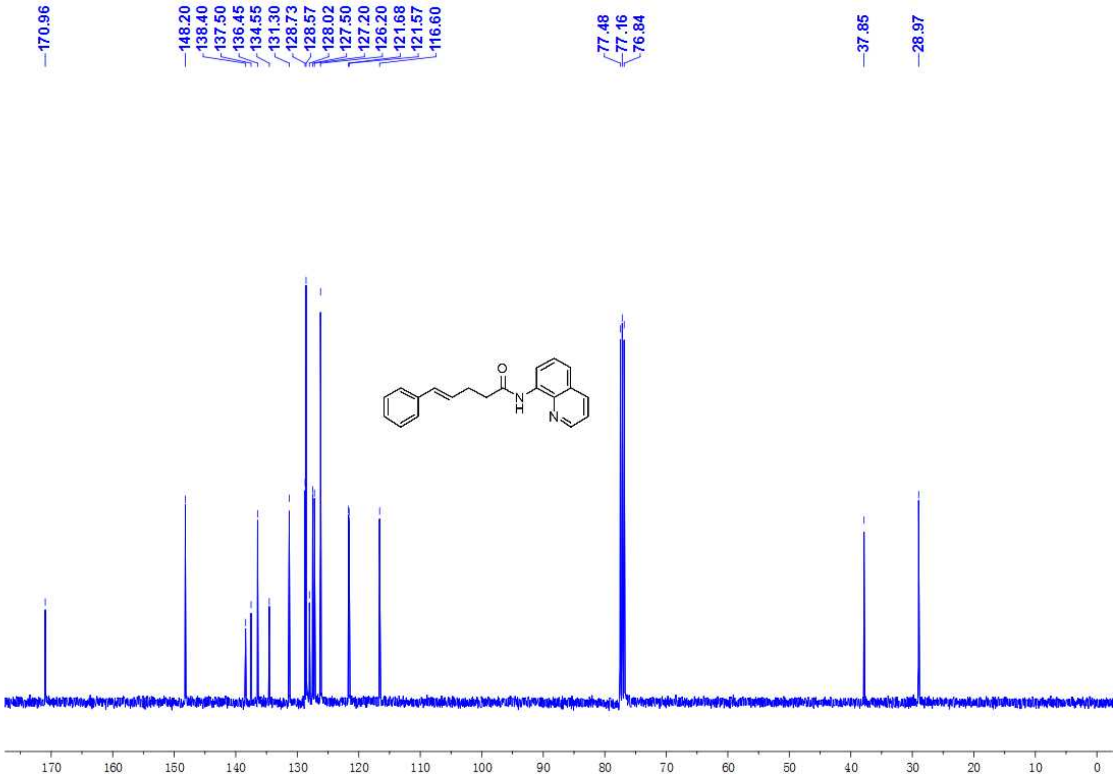

Supplementary Figure 50 ^{1}H NMR (400 MHz, $CDCl_3$) spectrum for 5ac

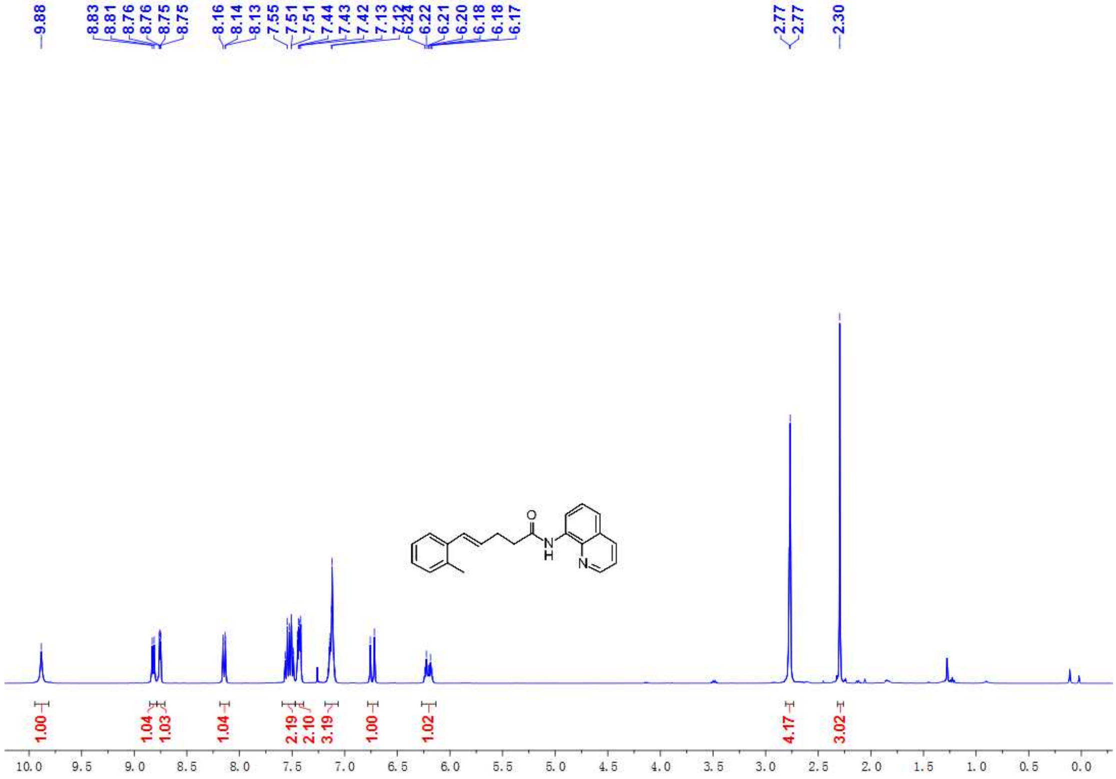

Supplementary Figure 51 ^{13}C NMR (100 MHz, $CDCl_3$) spectrum for 5ac

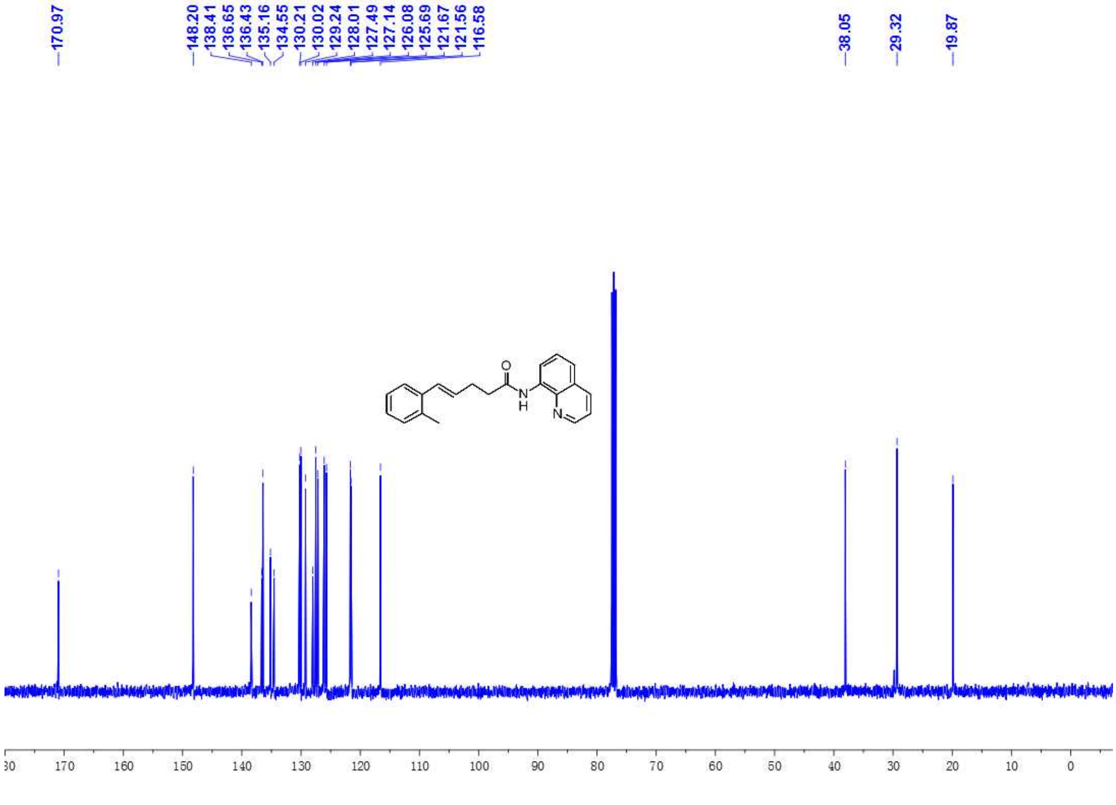

Supplementary Figure 52 ^{1}H NMR (400 MHz, $CDCl_3$) spectrum for 5ad

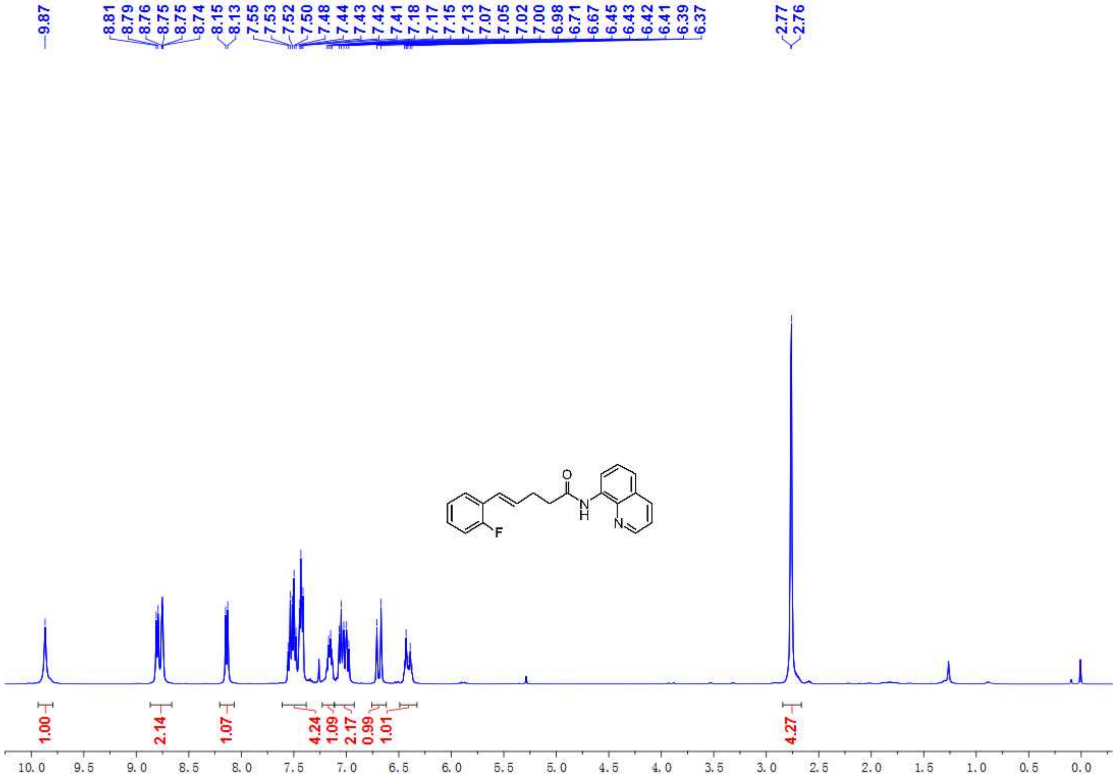

Supplementary Figure 53 ^{13}C NMR (100 MHz, $CDCl_3$) spectrum for 5ad

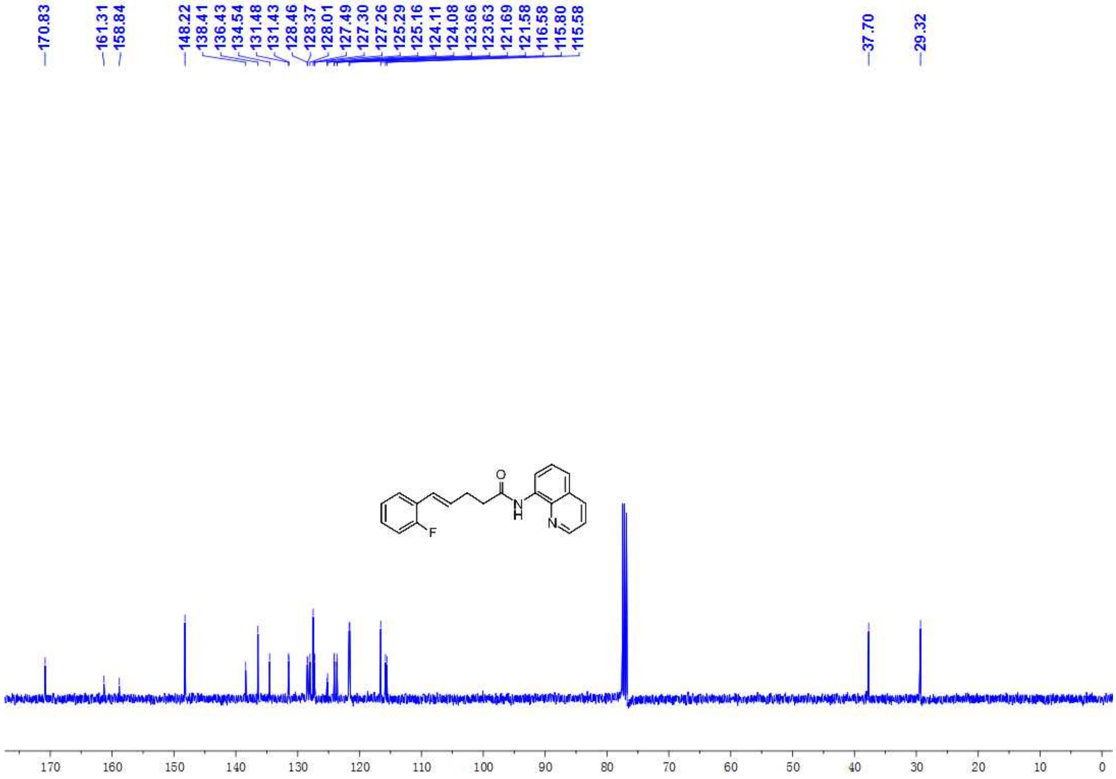

Supplementary Figure 54 ^{19}F NMR (376 MHz, $CDCl_3$) spectrum for 5ad

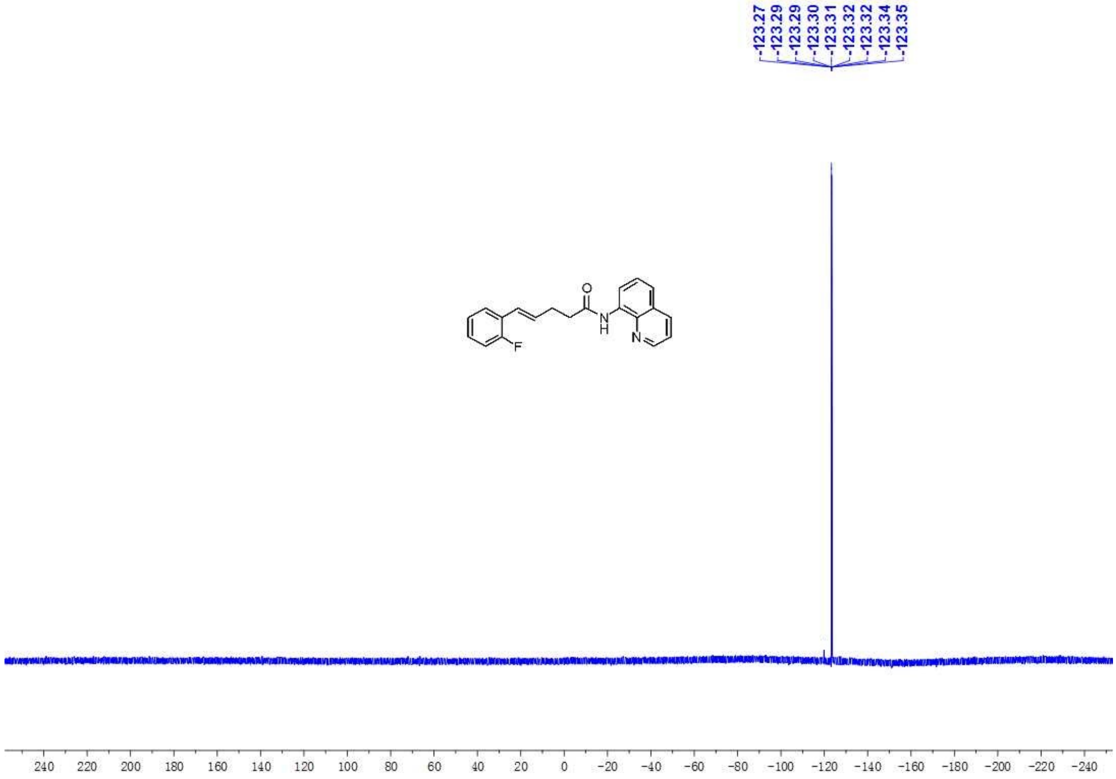

Supplementary Figure 55 ^{1}H NMR (400 MHz, $CDCl_3$) spectrum for 5ae

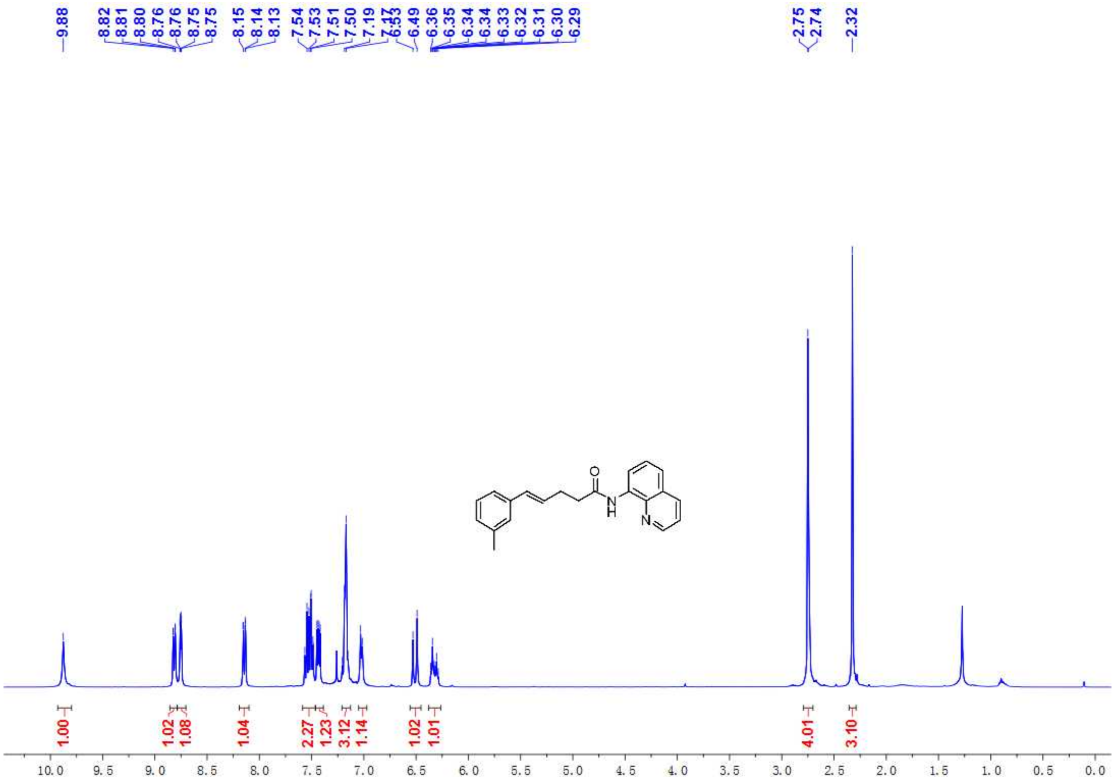

Supplementary Figure 56 ^{13}C NMR (100 MHz, $CDCl_3$) spectrum for 5ae

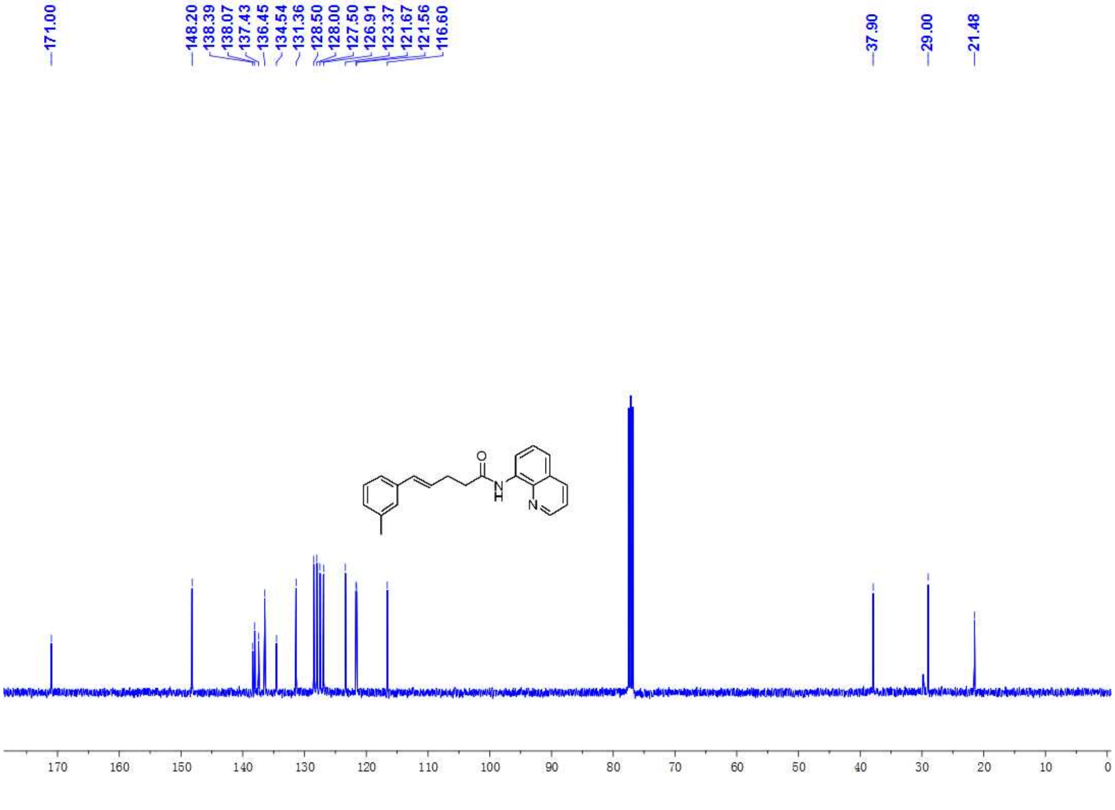

Supplementary Figure 57 ^{1}H NMR (400 MHz, $CDCl_3$) spectrum for 5af

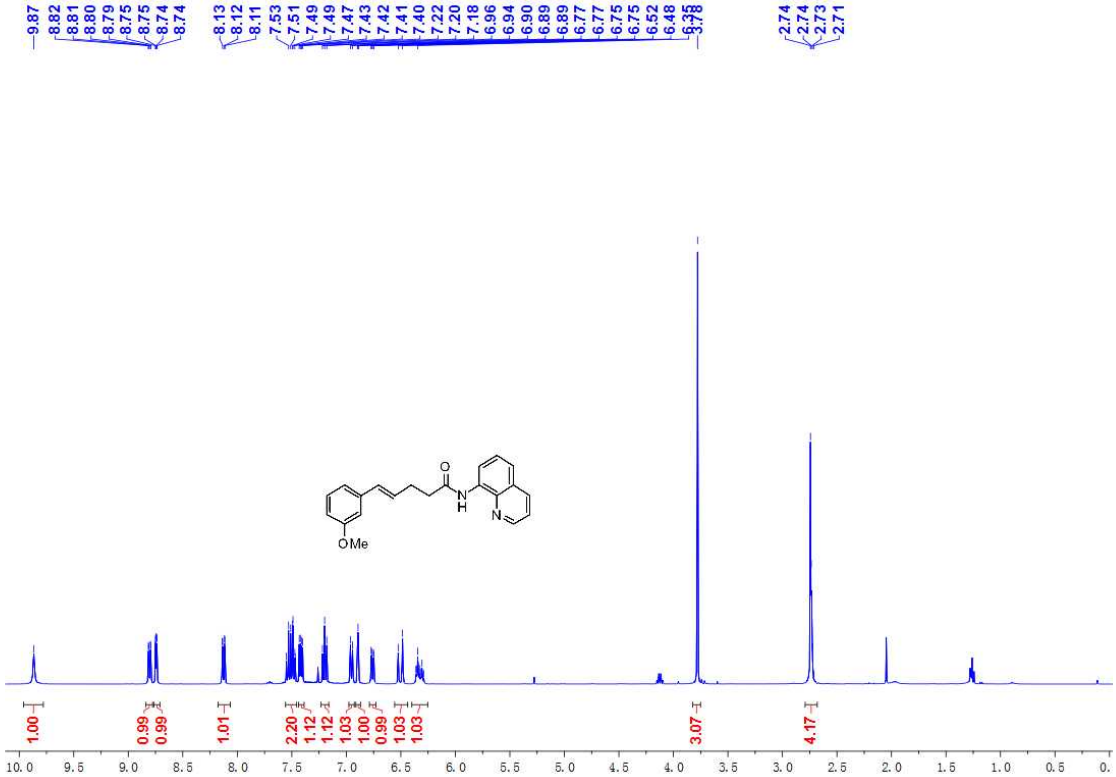

Supplementary Figure 58 ^{13}C NMR (100 MHz, $CDCl_3$) spectrum for 5af

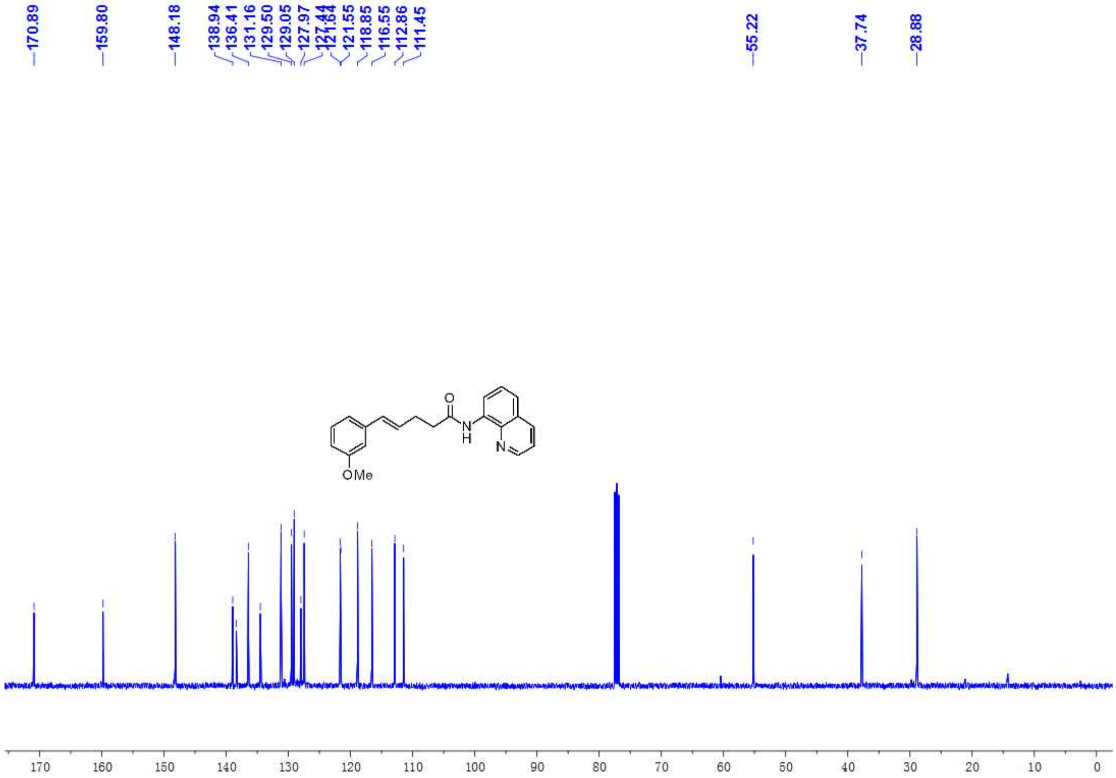

Supplementary Figure 59 ^{1}H NMR (400 MHz, $CDCl_3$) spectrum for 5ag

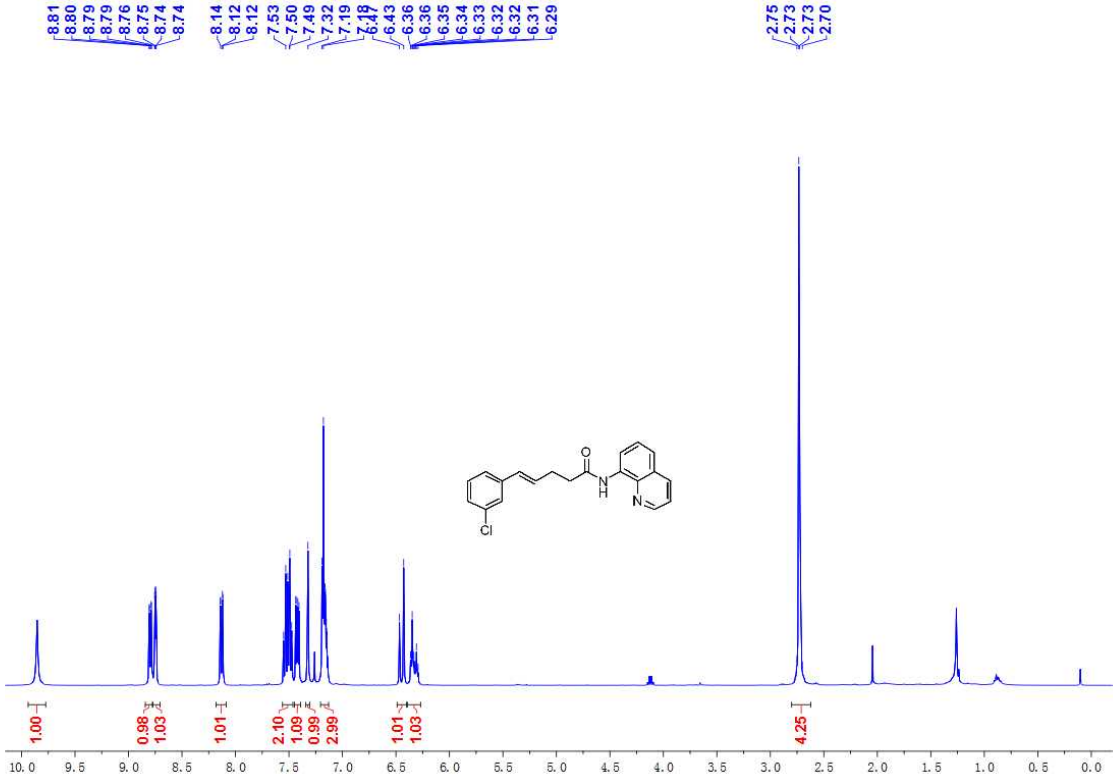

Supplementary Figure 60 ^{13}C NMR (100 MHz, $CDCl_3$) spectrum for 5ag

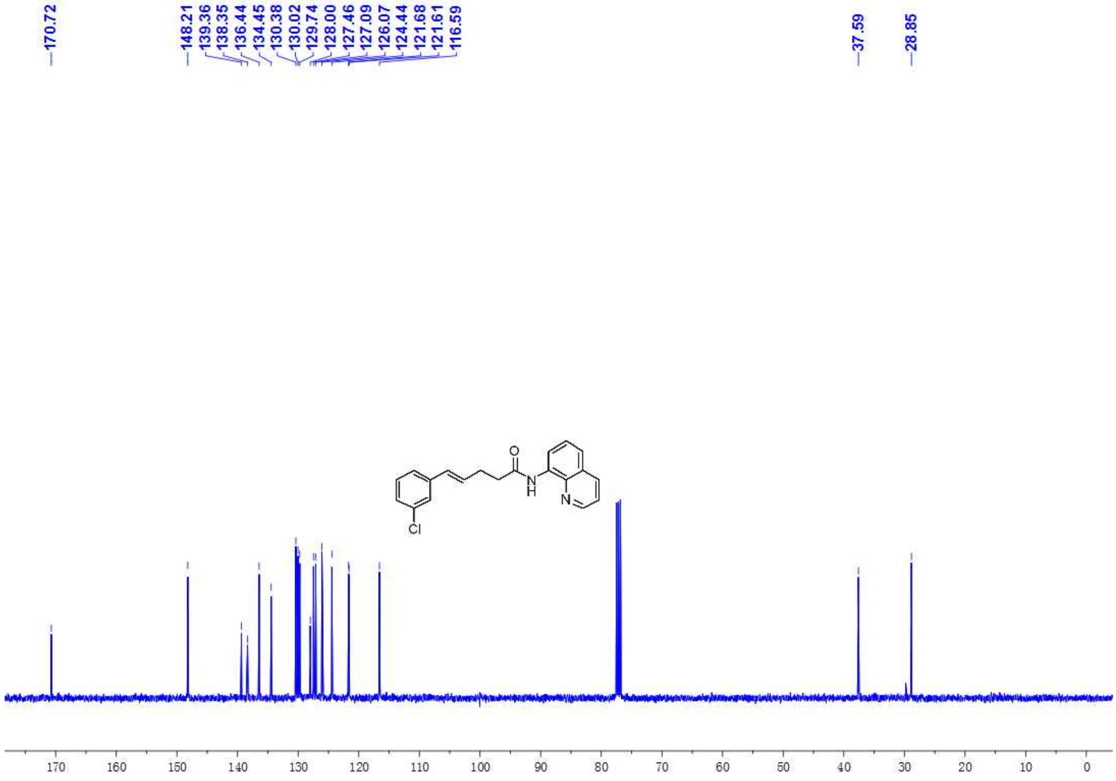

Supplementary Figure 61 ^{1}H NMR (400 MHz, $CDCl_3$) spectrum for 5ah

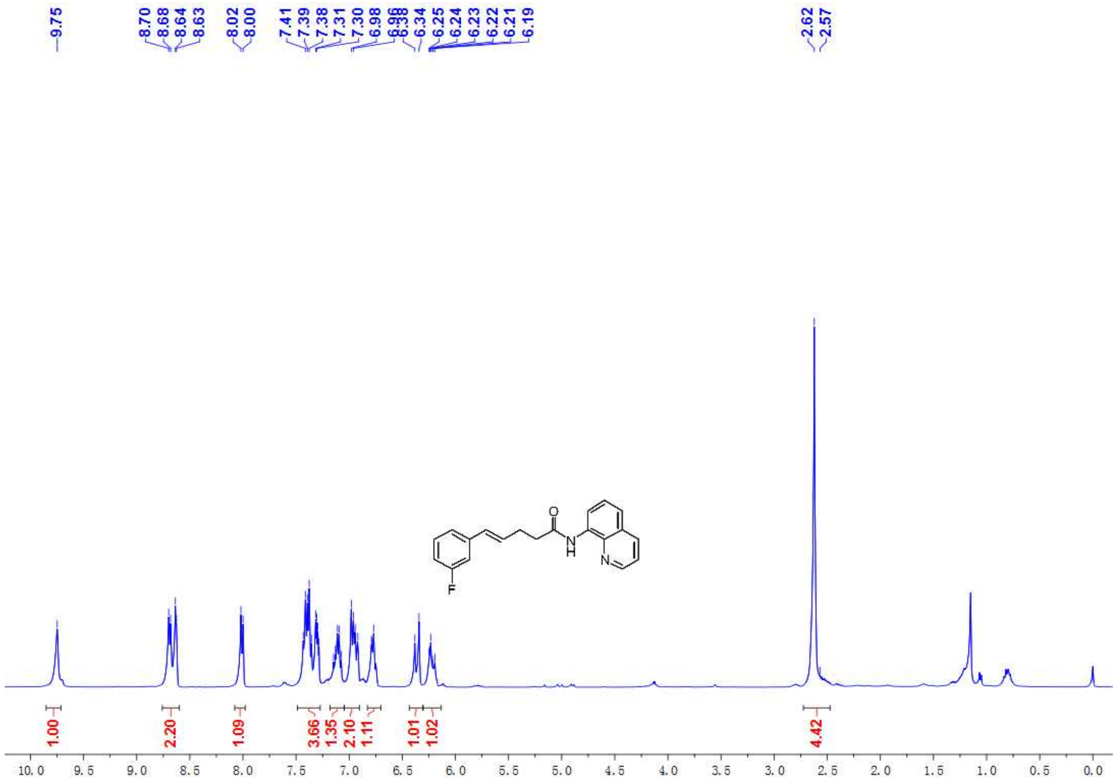

Supplementary Figure 62 ^{13}C NMR (100 MHz, $CDCl_3$) spectrum for 5ah

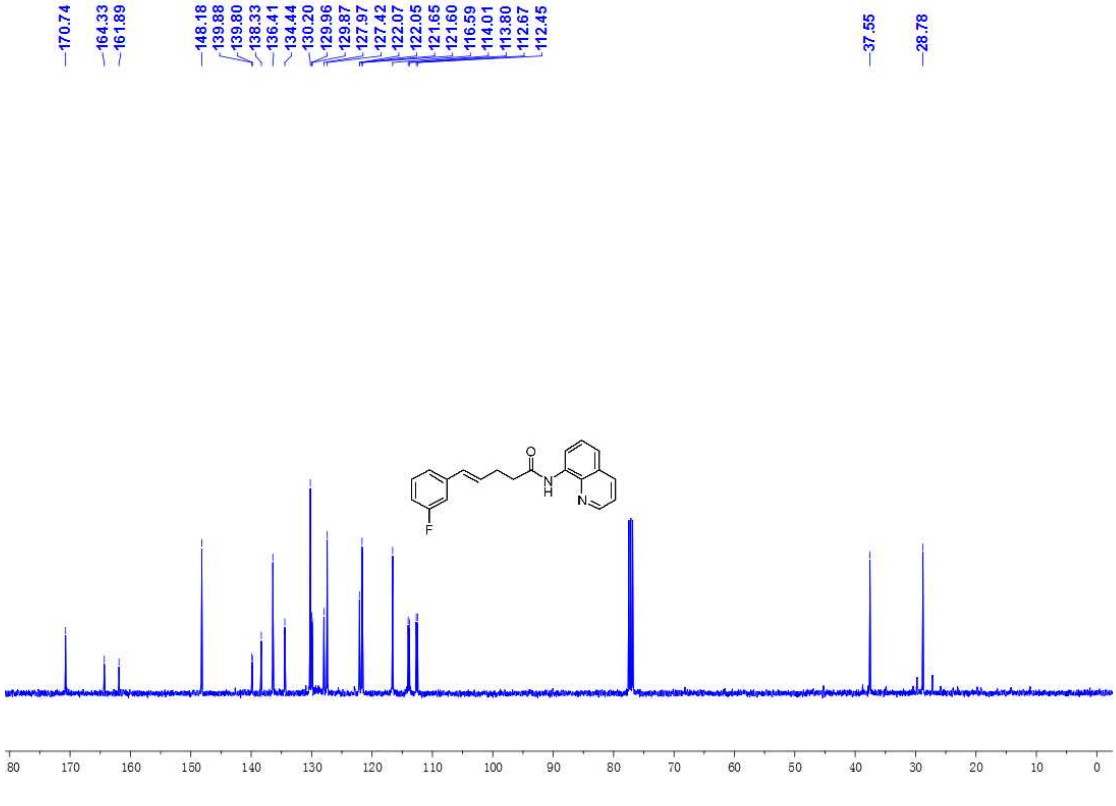

Supplementary Figure 63 ^{19}F NMR (376 MHz, $CDCl_3$) spectrum for 5ah

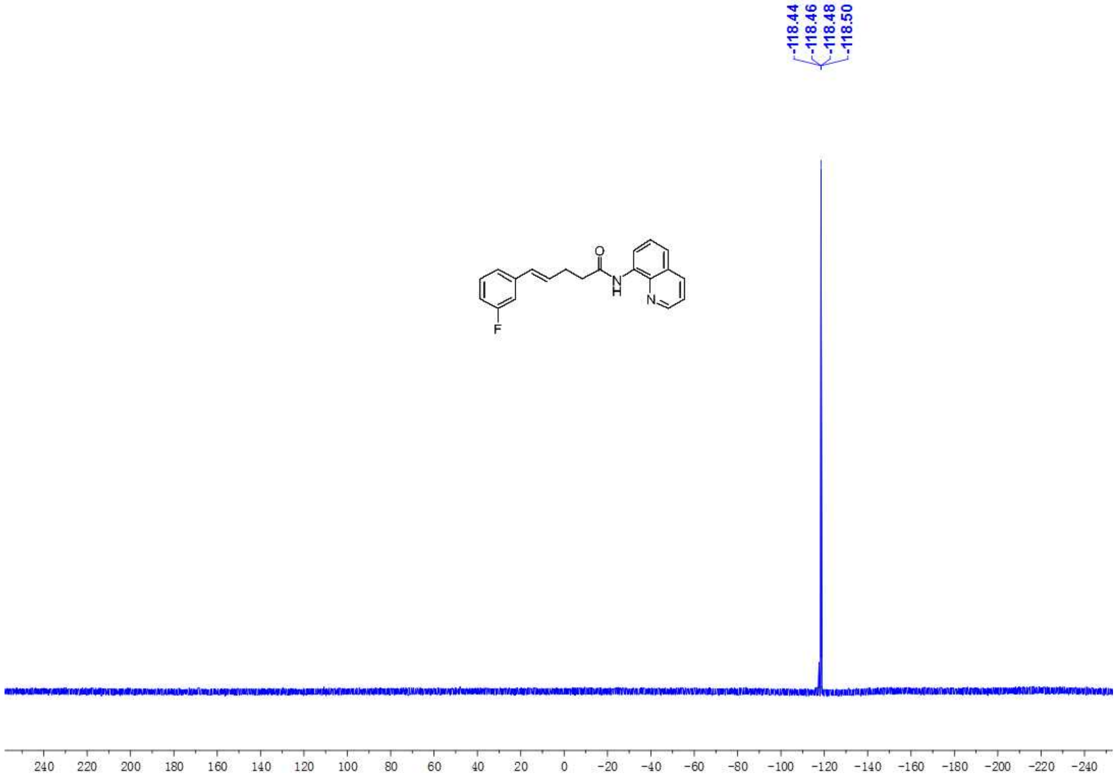

Supplementary Figure 64 ^{1}H NMR (400 MHz, $CDCl_3$) spectrum for 5ai

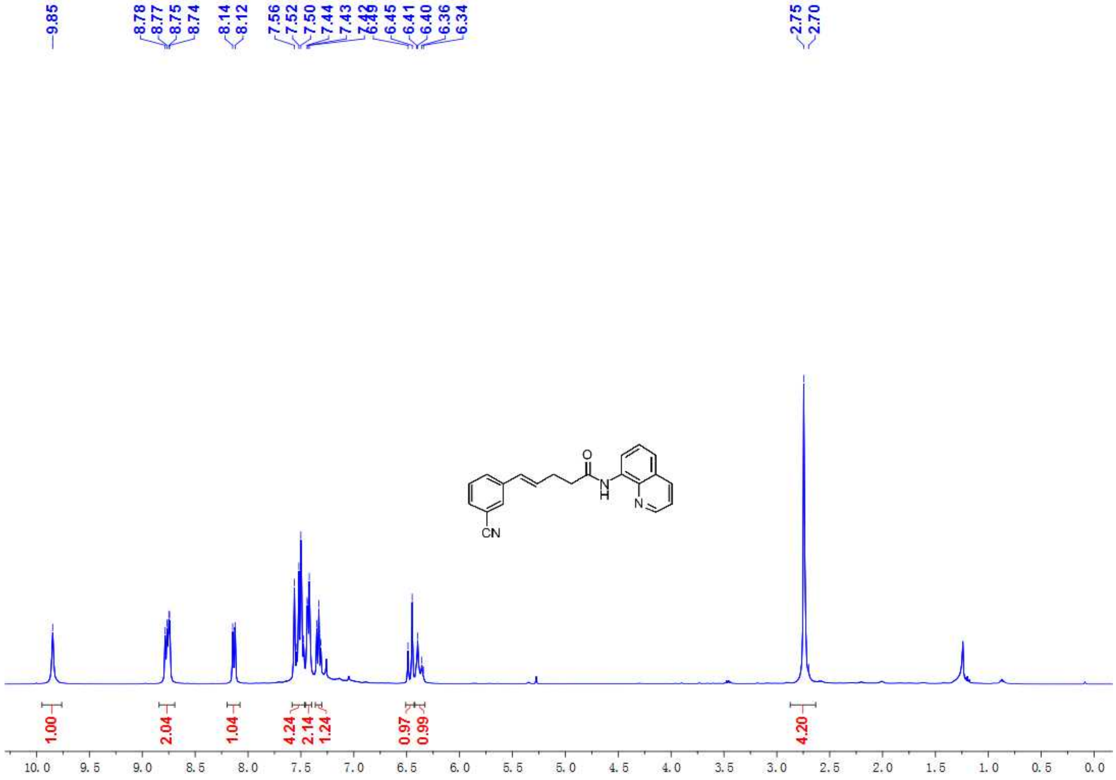

Supplementary Figure 65 ^{13}C NMR (100 MHz, $CDCl_3$) spectrum for 5ai

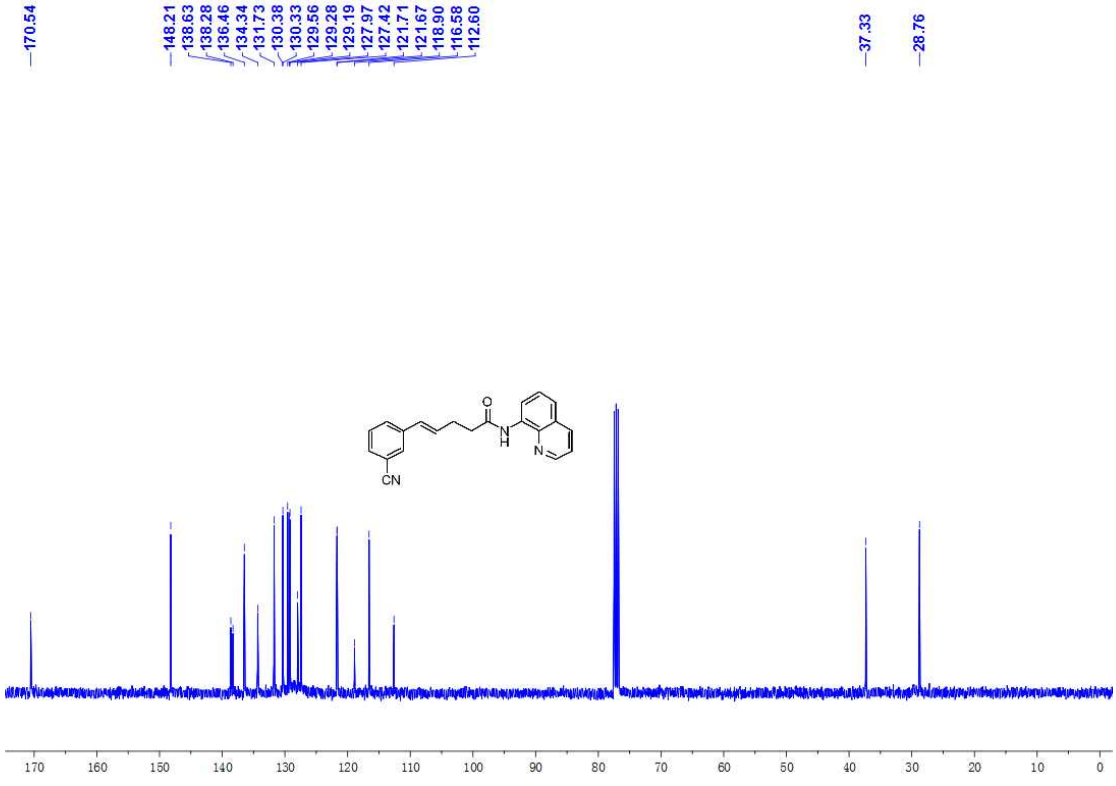

Supplementary Figure 66 ^{1}H NMR (400 MHz, $CDCl_3$) spectrum for 5aj

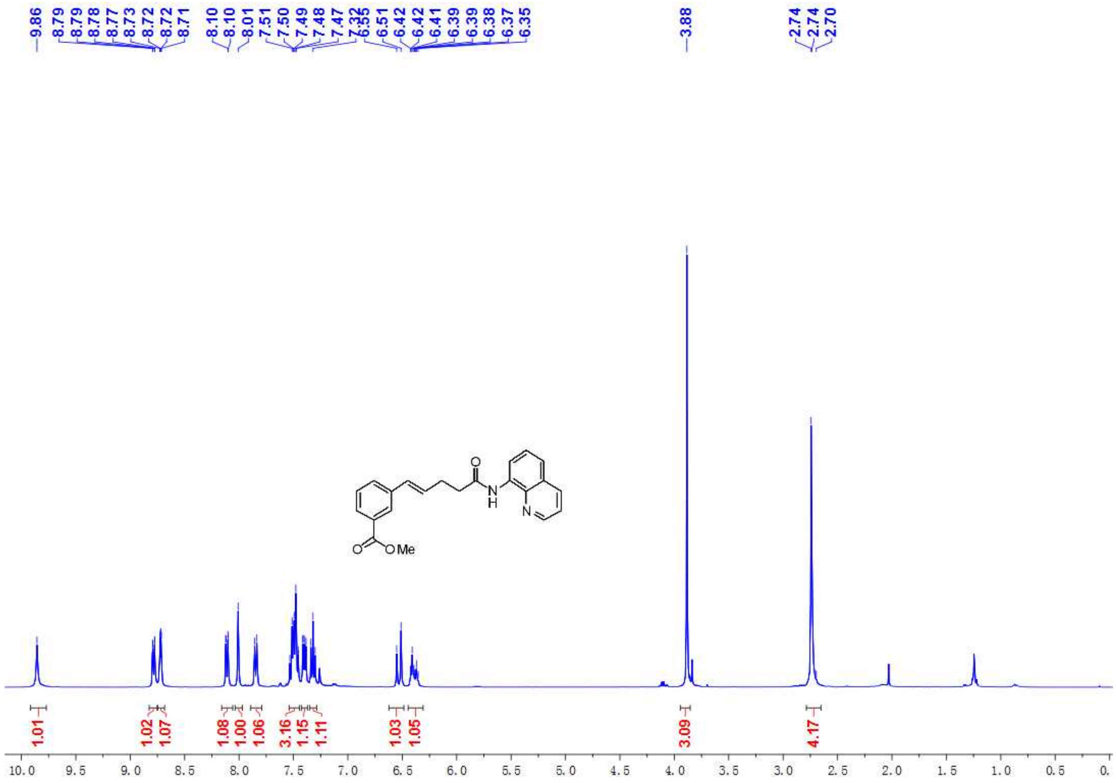

Supplementary Figure 67 ^{13}C NMR (100 MHz, $CDCl_3$) spectrum for 5aj

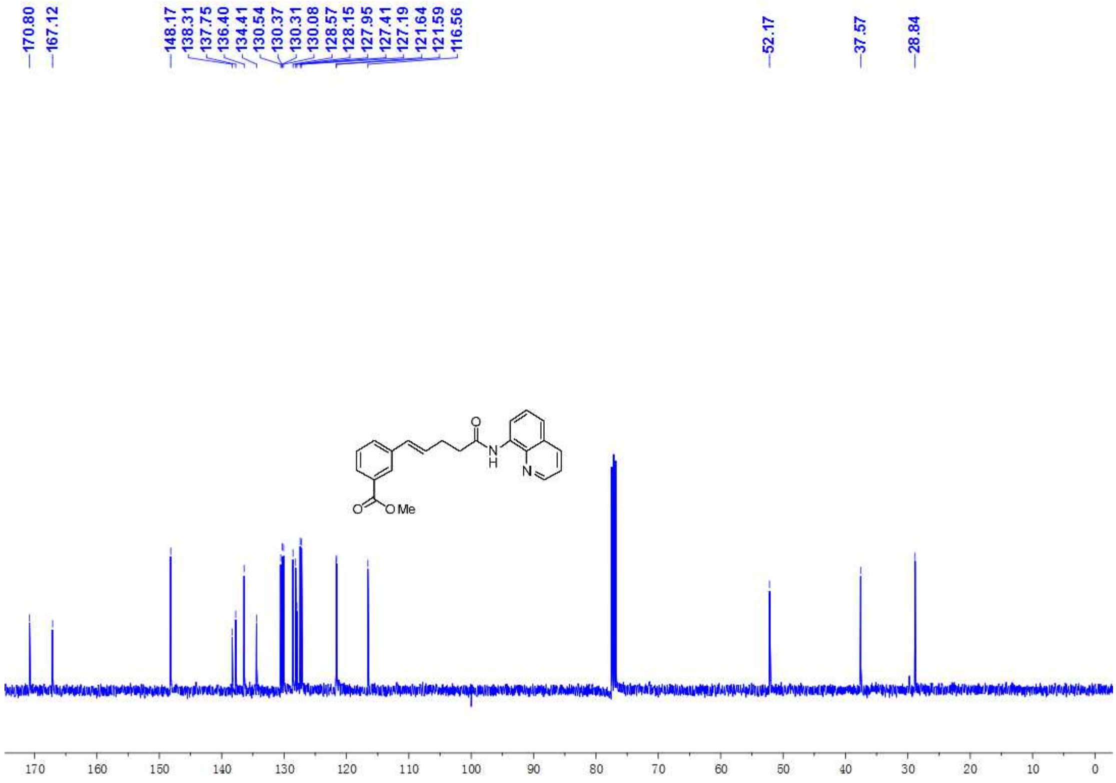

Supplementary Figure 68 ^{1}H NMR (400 MHz, $CDCl_3$) spectrum for 5ak

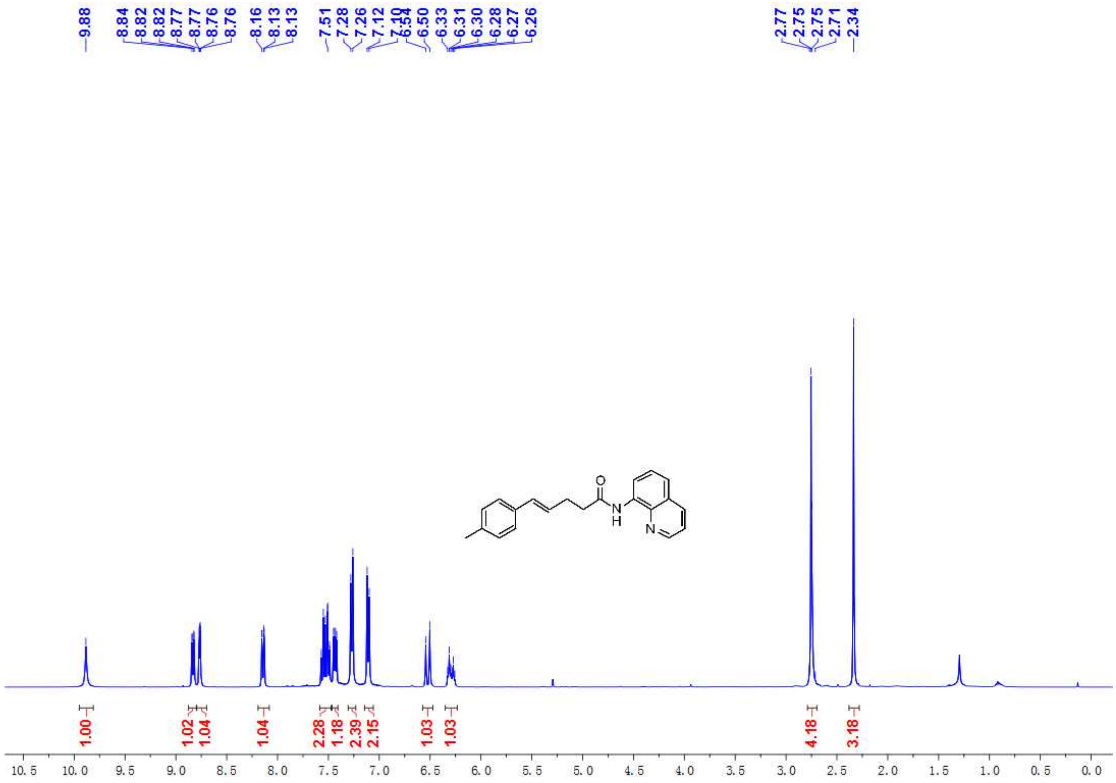

Supplementary Figure 69 ^{13}C NMR (100 MHz, $CDCl_3$) spectrum for 5ak

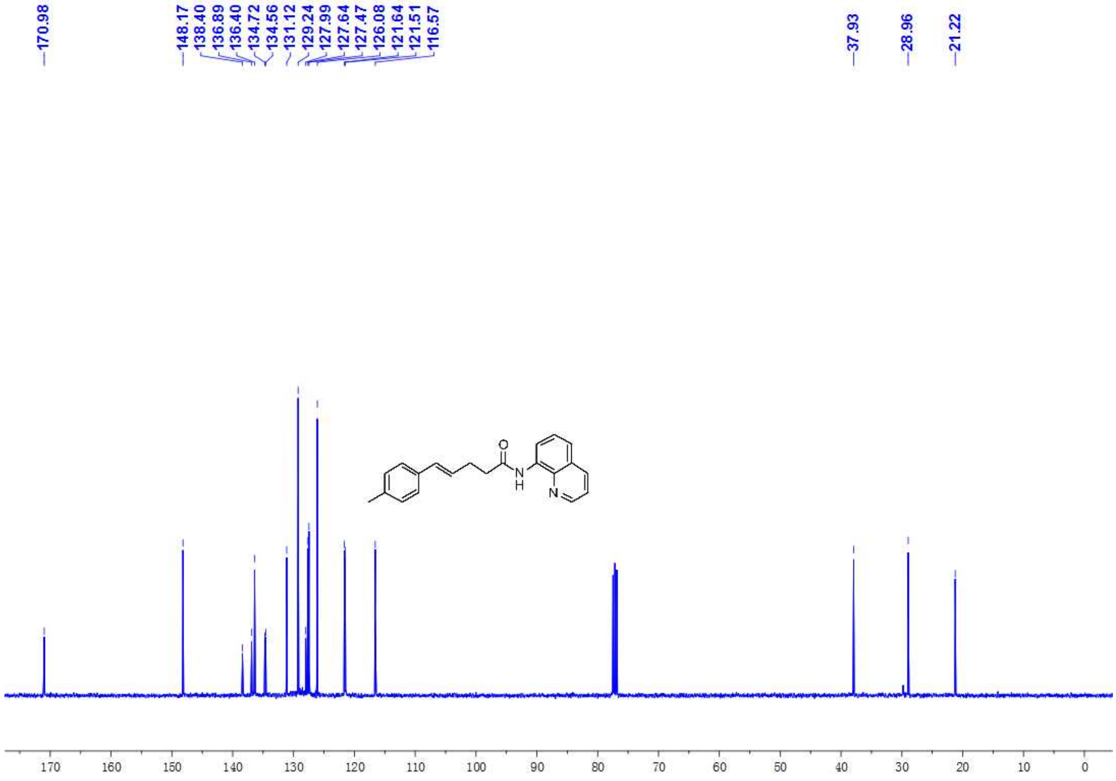

Supplementary Figure 70 ^{1}H NMR (400 MHz, $CDCl_3$) spectrum for 5al

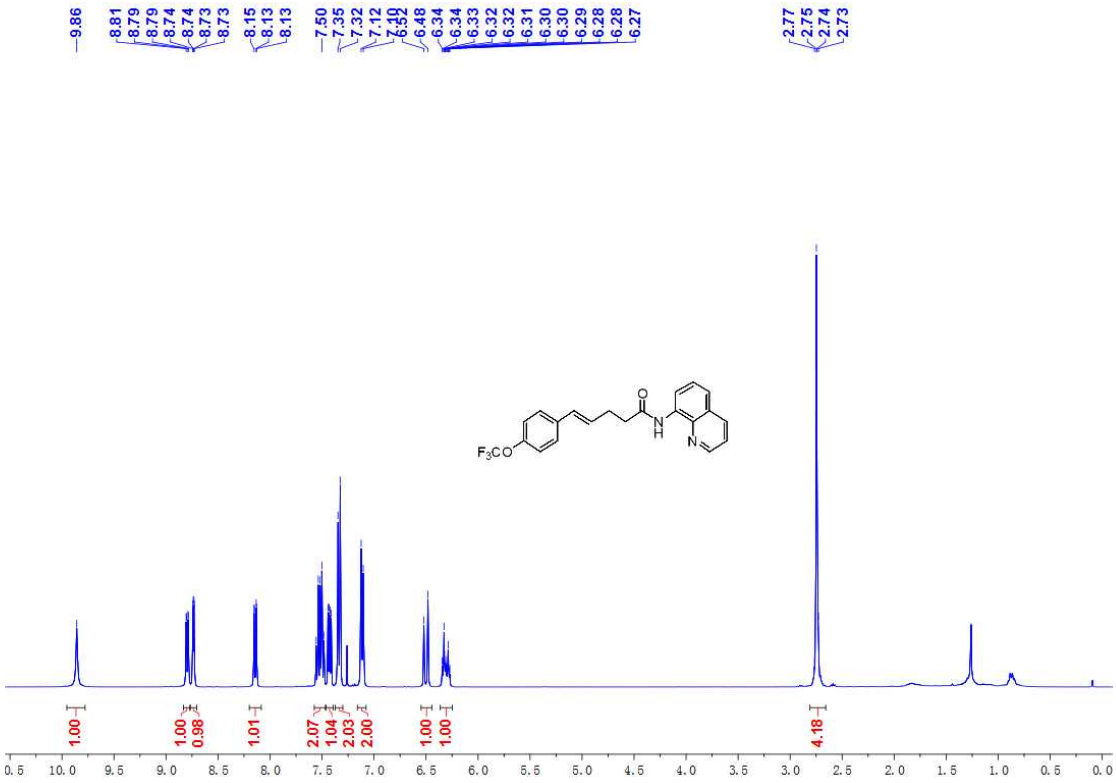

Supplementary Figure 71 ^{13}C NMR (100 MHz, $CDCl_3$) spectrum for 5al

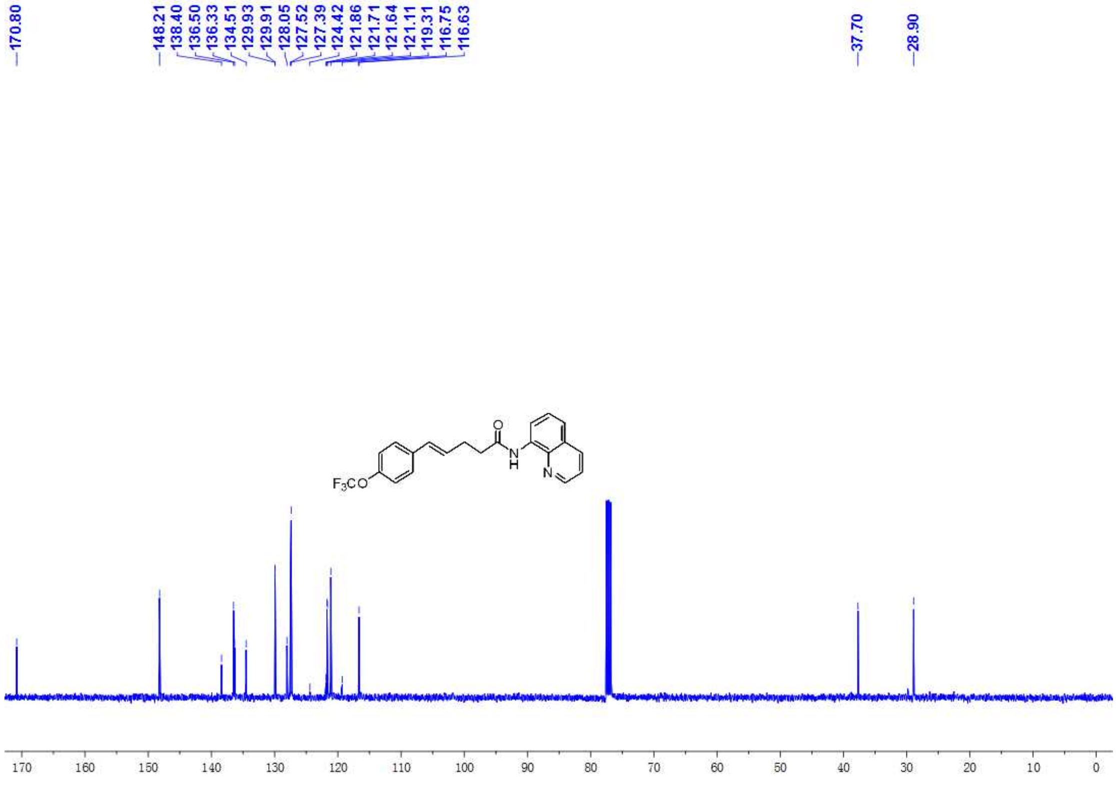

Supplementary Figure 72 ^{19}F NMR (376 MHz, $CDCl_3$) spectrum for 5al

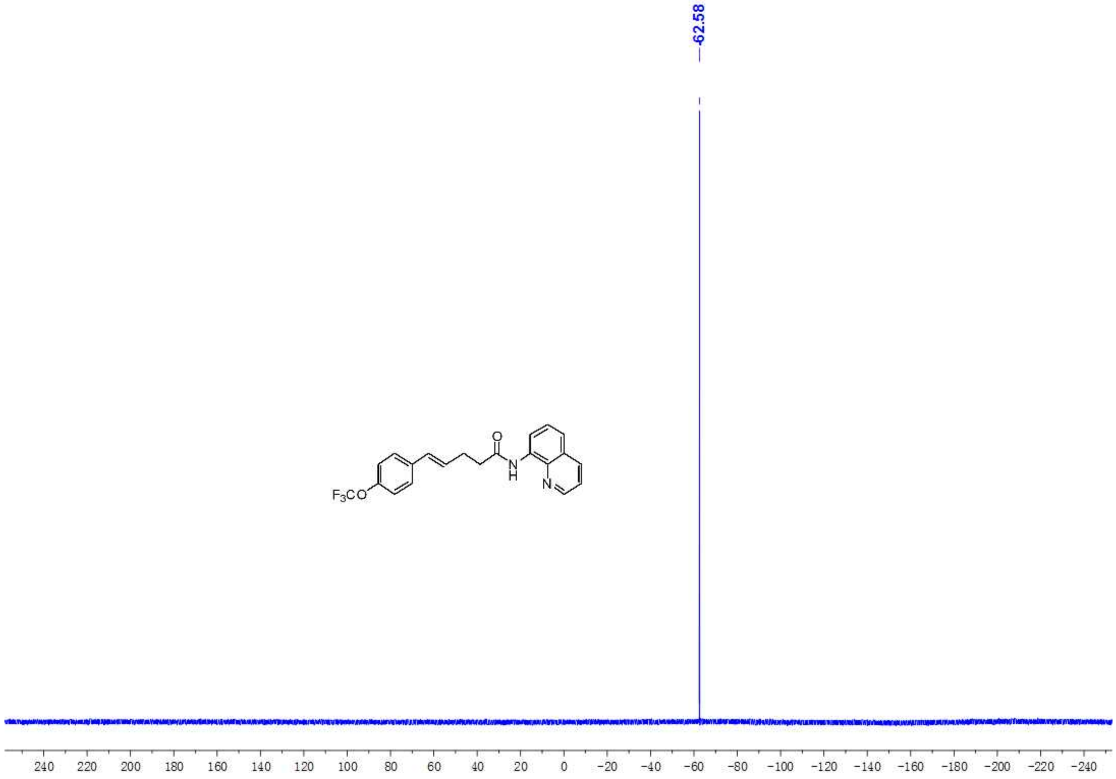

Supplementary Figure 73 ^{1}H NMR (400 MHz, $CDCl_3$) spectrum for 5am

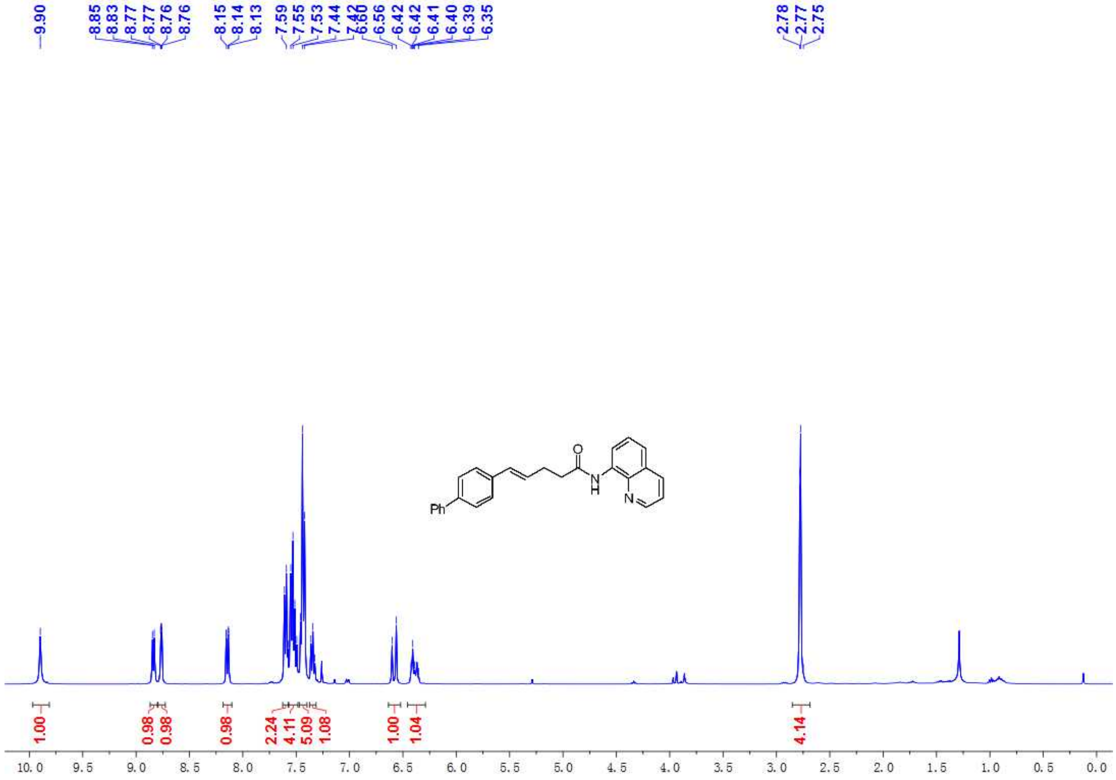

Supplementary Figure 74 ^{13}C NMR (100 MHz, $CDCl_3$) spectrum for 5am

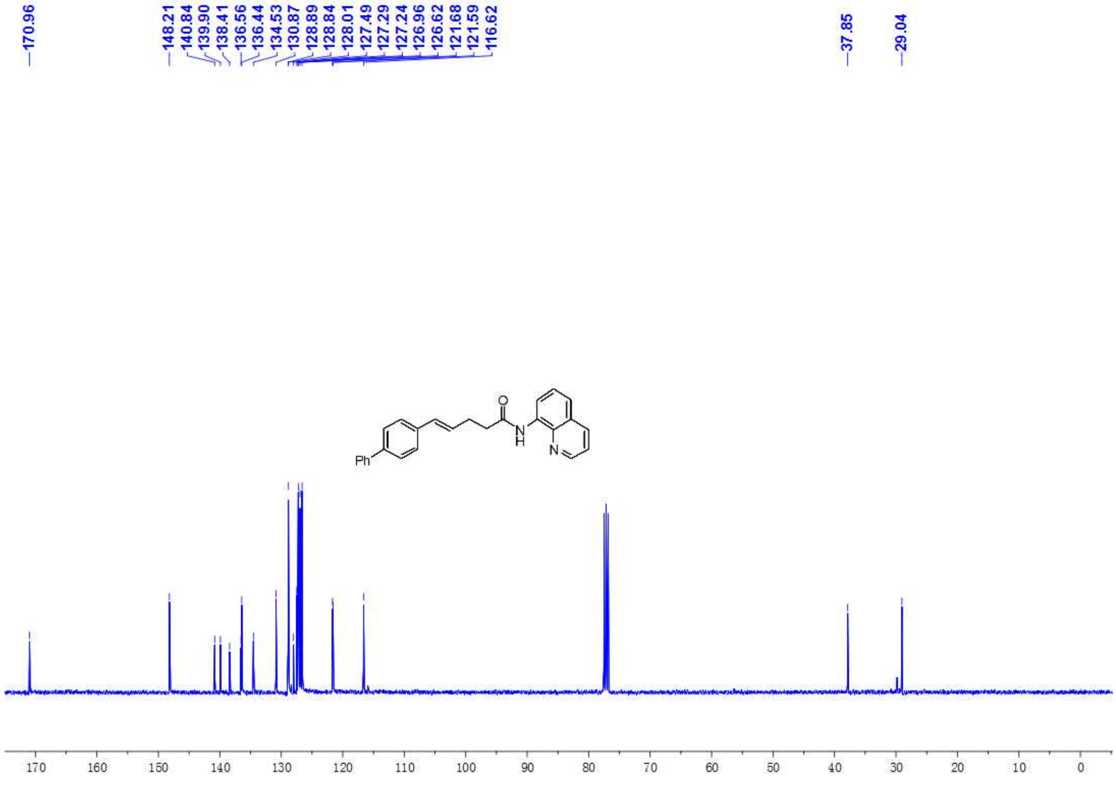

Supplementary Figure 75 ^{1}H NMR (400 MHz, $CDCl_3$) spectrum for 5an

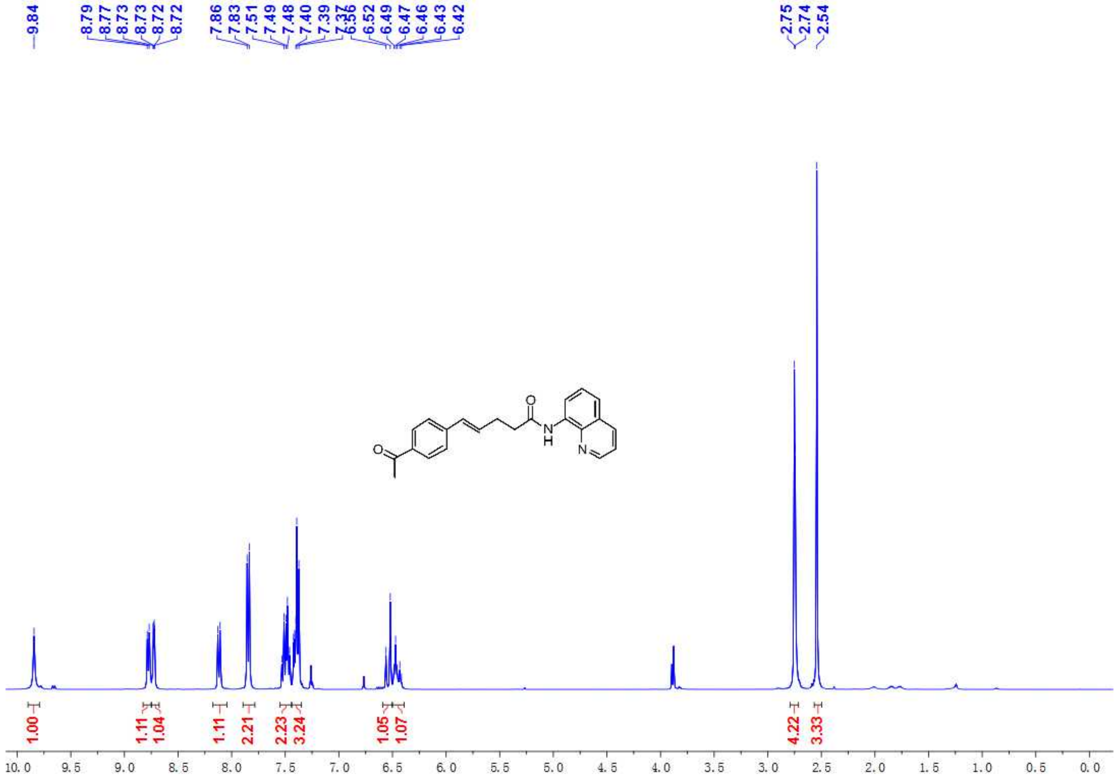

Supplementary Figure 76 ^{13}C NMR (100 MHz, $CDCl_3$) spectrum for 5an

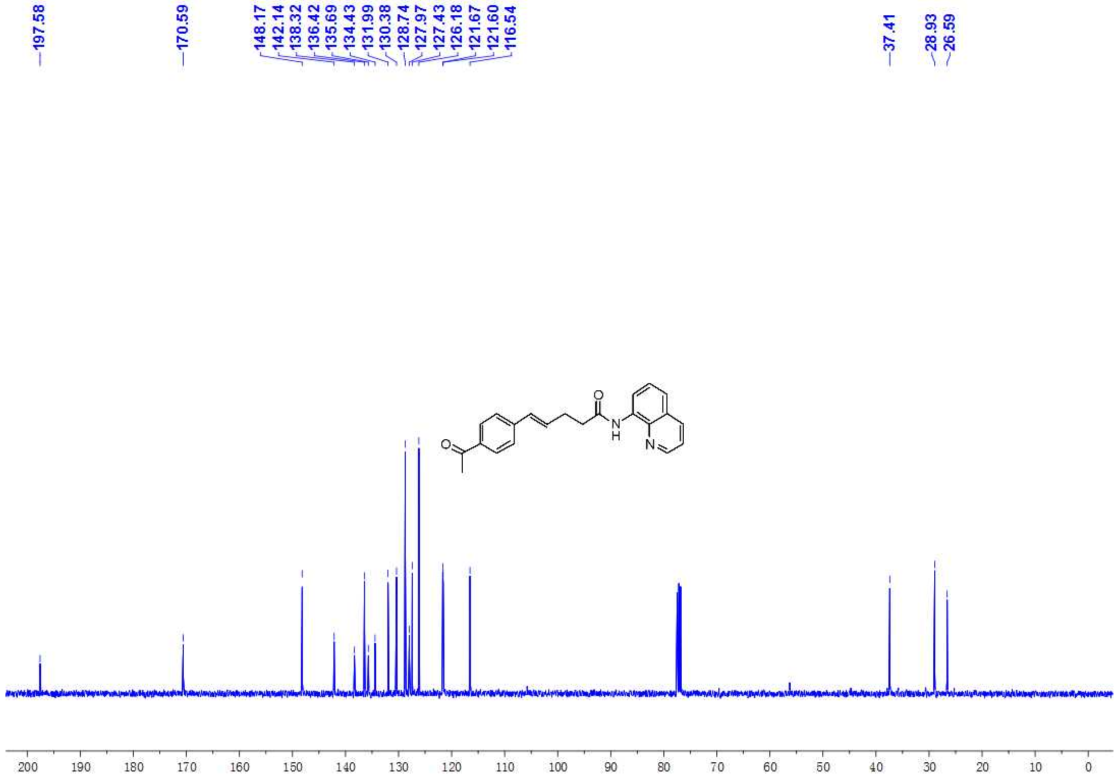

Supplementary Figure 77 ^1H NMR (400 MHz, $CDCl_3$) spectrum for **5ao**

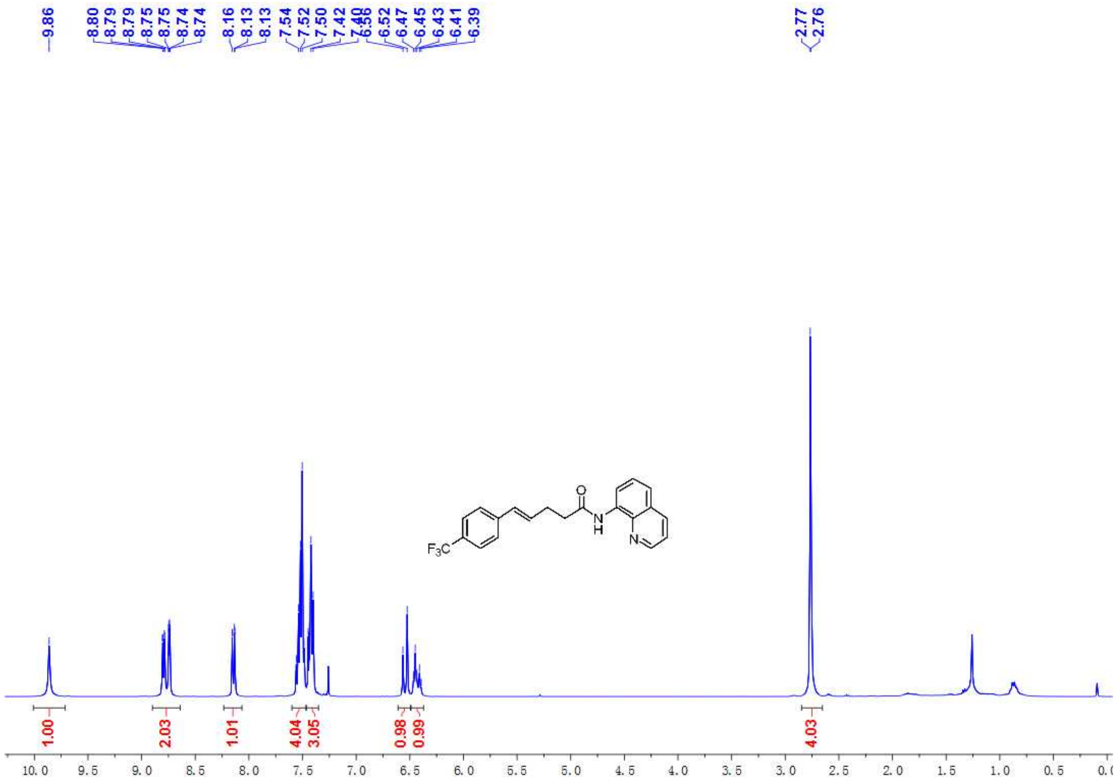

Supplementary Figure 78 ^{13}C NMR (100 MHz, $CDCl_3$) spectrum for **5ao**

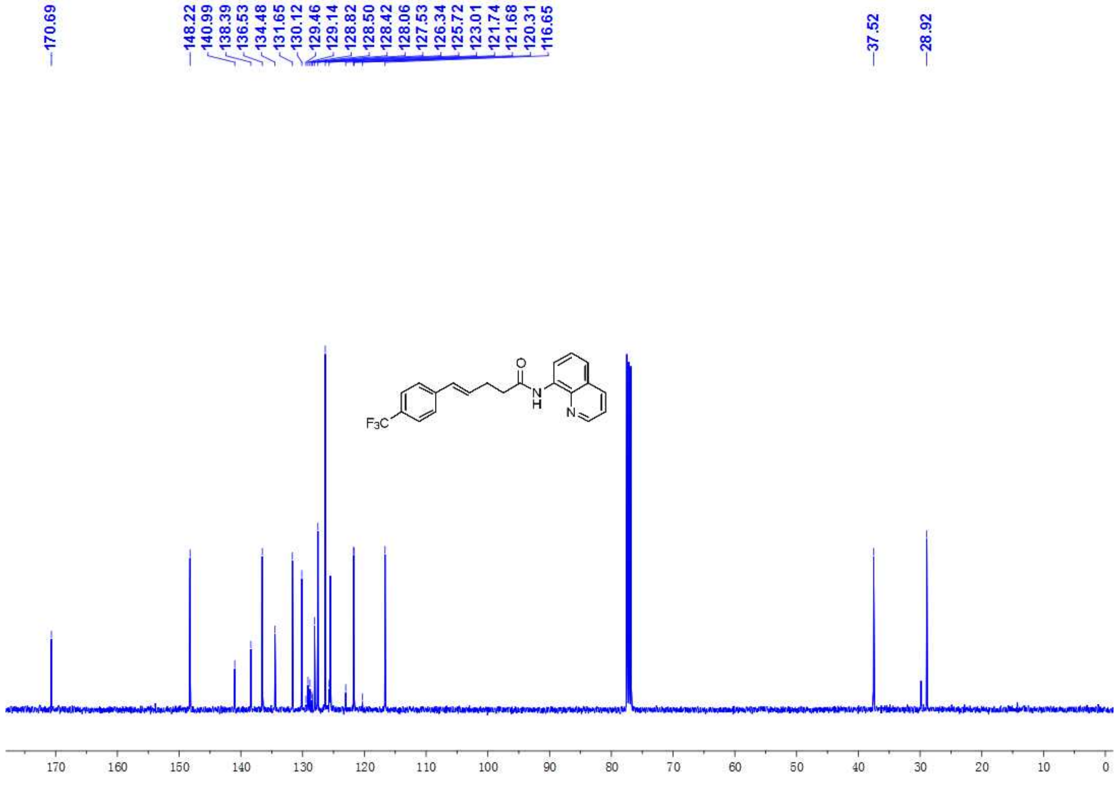

Supplementary Figure 79 ^{19}F NMR (376 MHz, $CDCl_3$) spectrum for 5ao

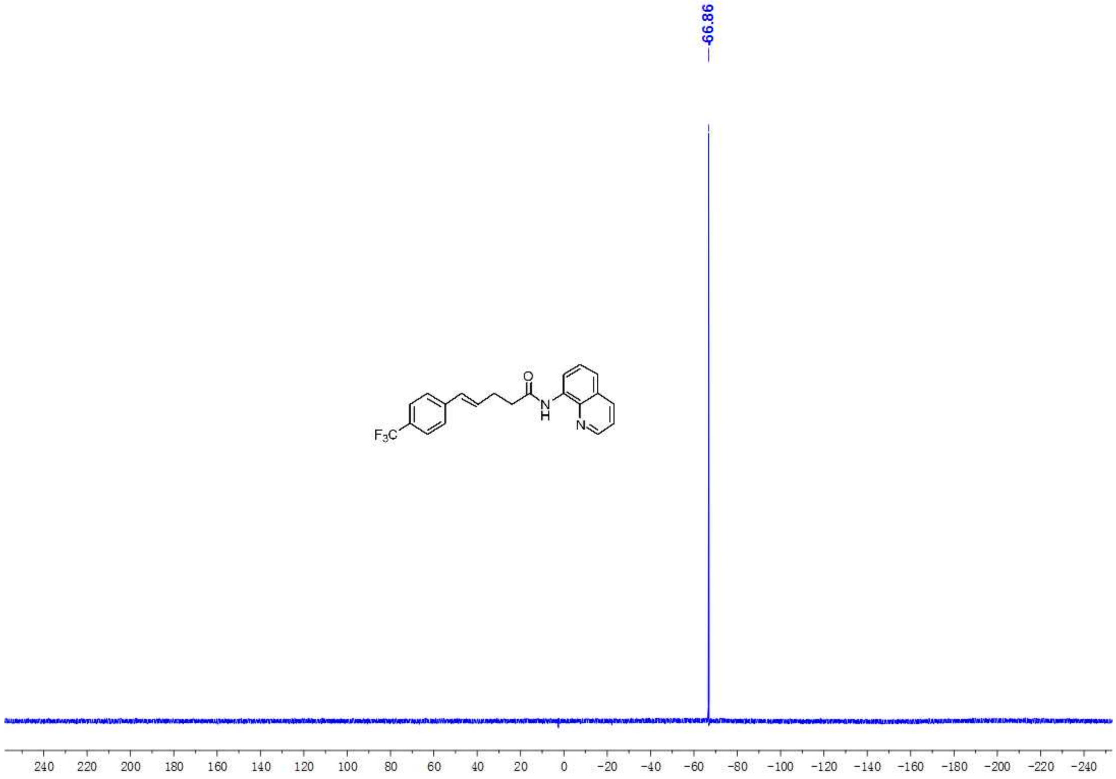

Supplementary Figure 80 ^{1}H NMR (400 MHz, $CDCl_3$) spectrum for 5ap

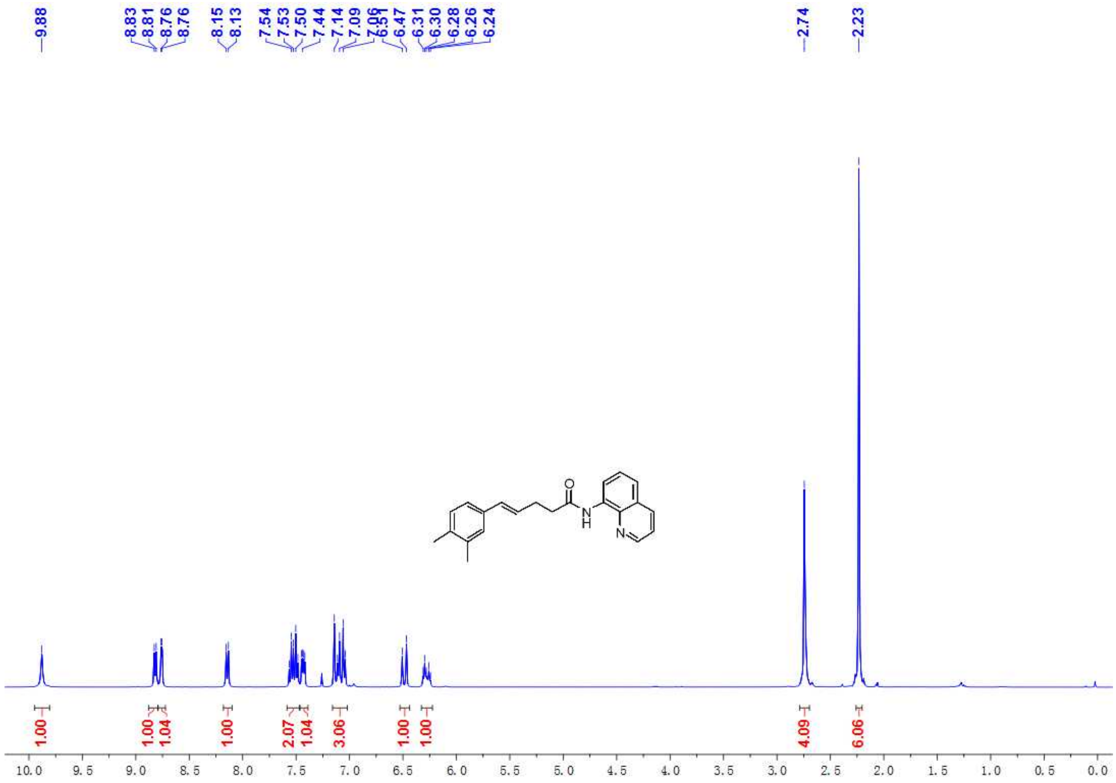

Supplementary Figure 81 ^{13}C NMR (100 MHz, $CDCl_3$) spectrum for 5ap

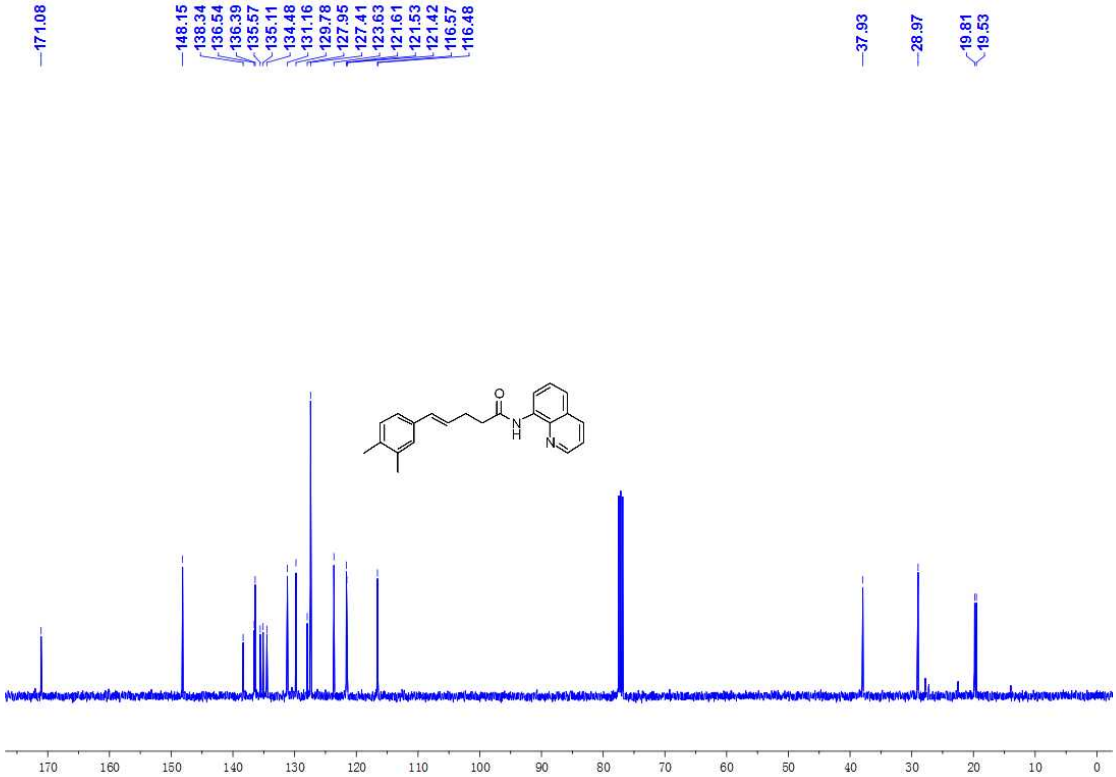

Supplementary Figure 82 ^{1}H NMR (400 MHz, $CDCl_3$) spectrum for 5aq

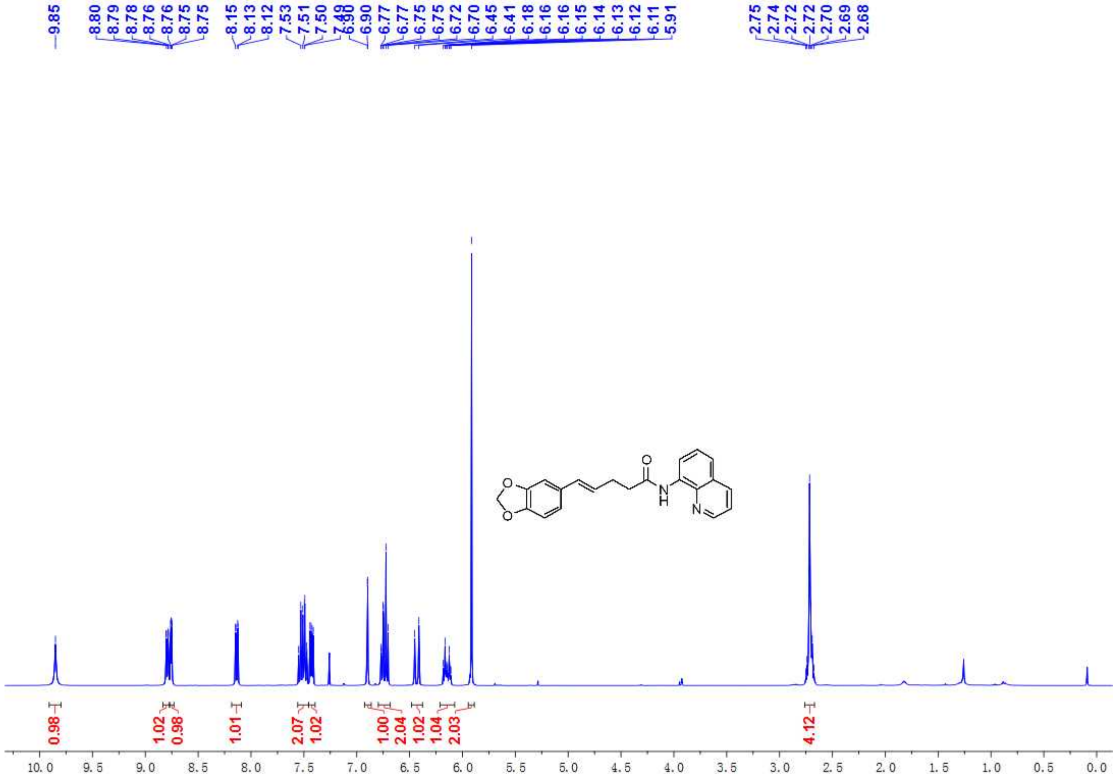

Supplementary Figure 83 ^{13}C NMR (100 MHz, $CDCl_3$) spectrum for 5aq

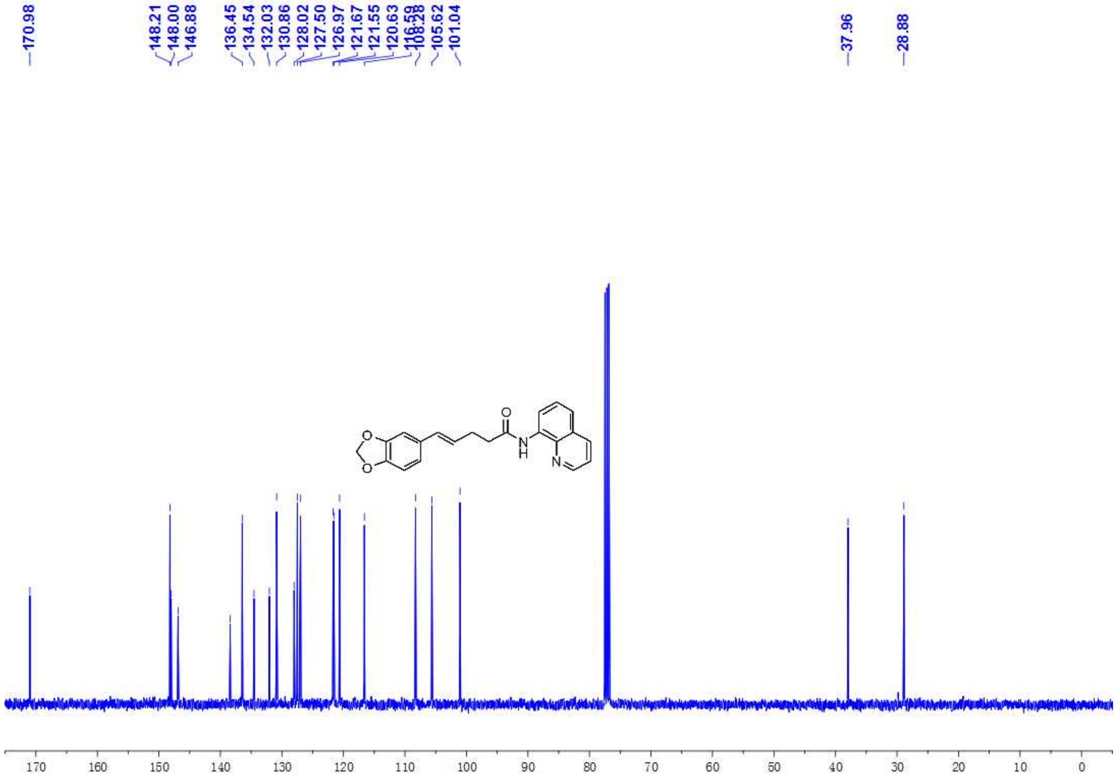

Supplementary Figure 84 ^{1}H NMR (400 MHz, $CDCl_3$) spectrum for 5ar

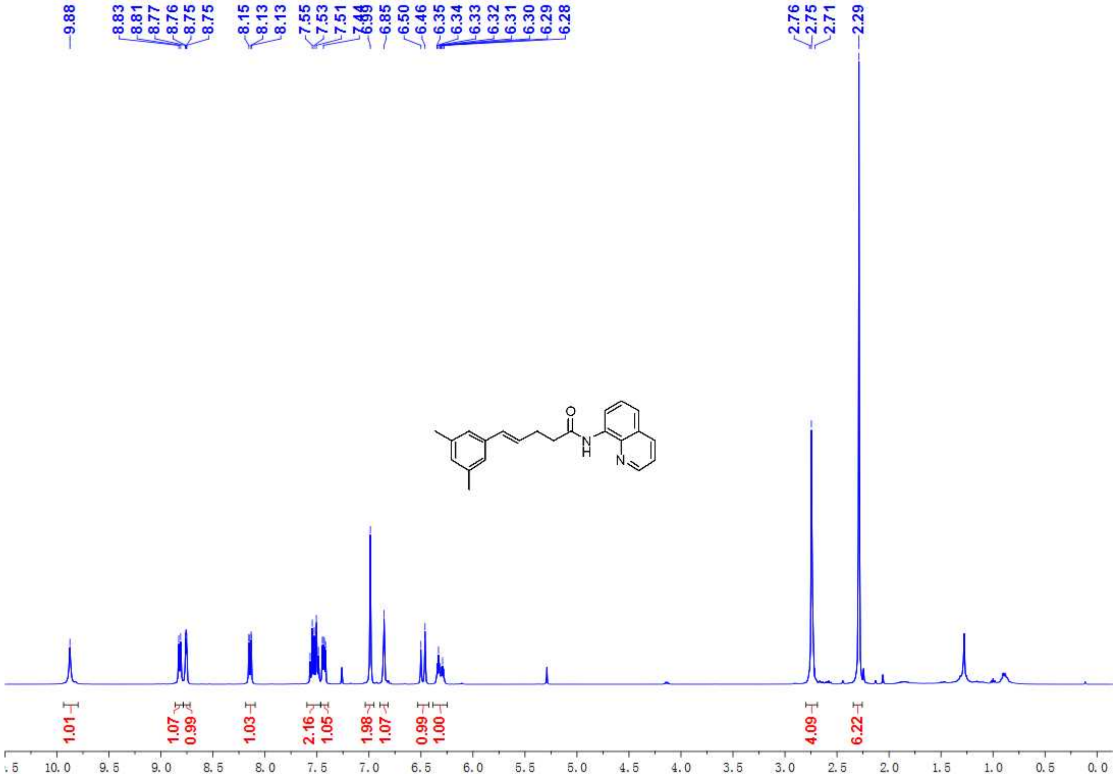

Supplementary Figure 85 ^{13}C NMR (100 MHz, $CDCl_3$) spectrum for 5ar

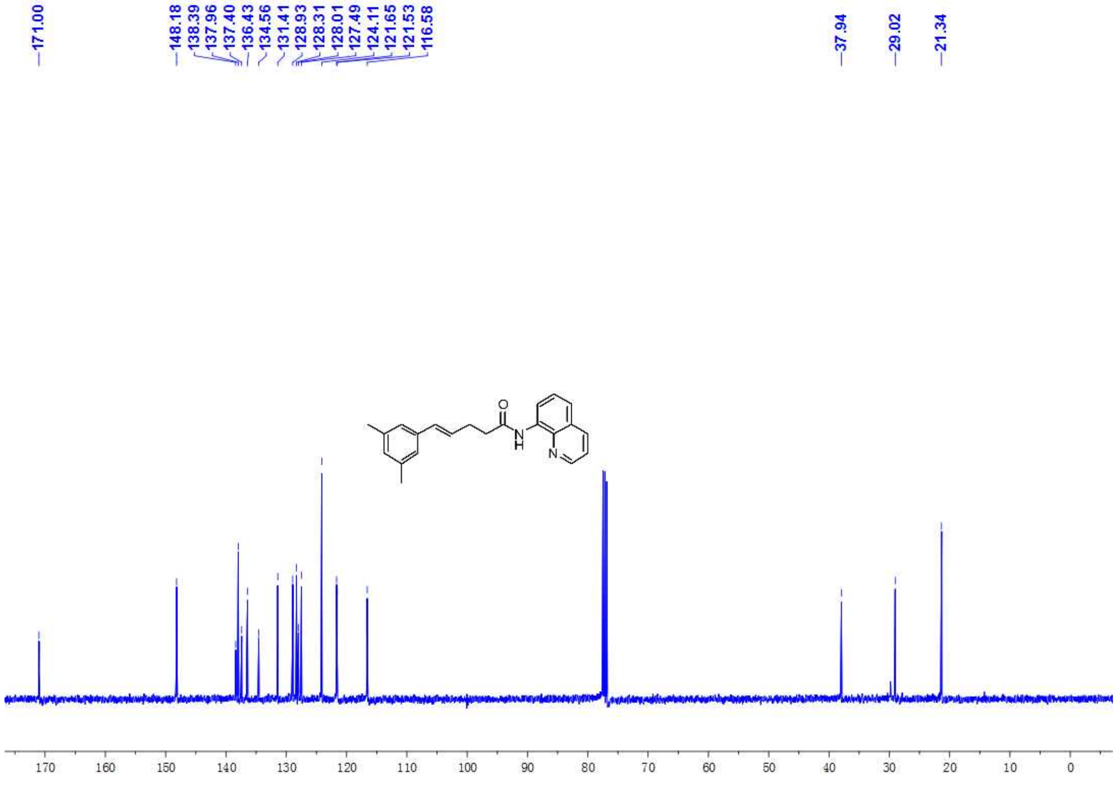

Supplementary Figure 86 ^{1}H NMR (400 MHz, $CDCl_3$) spectrum for 5as

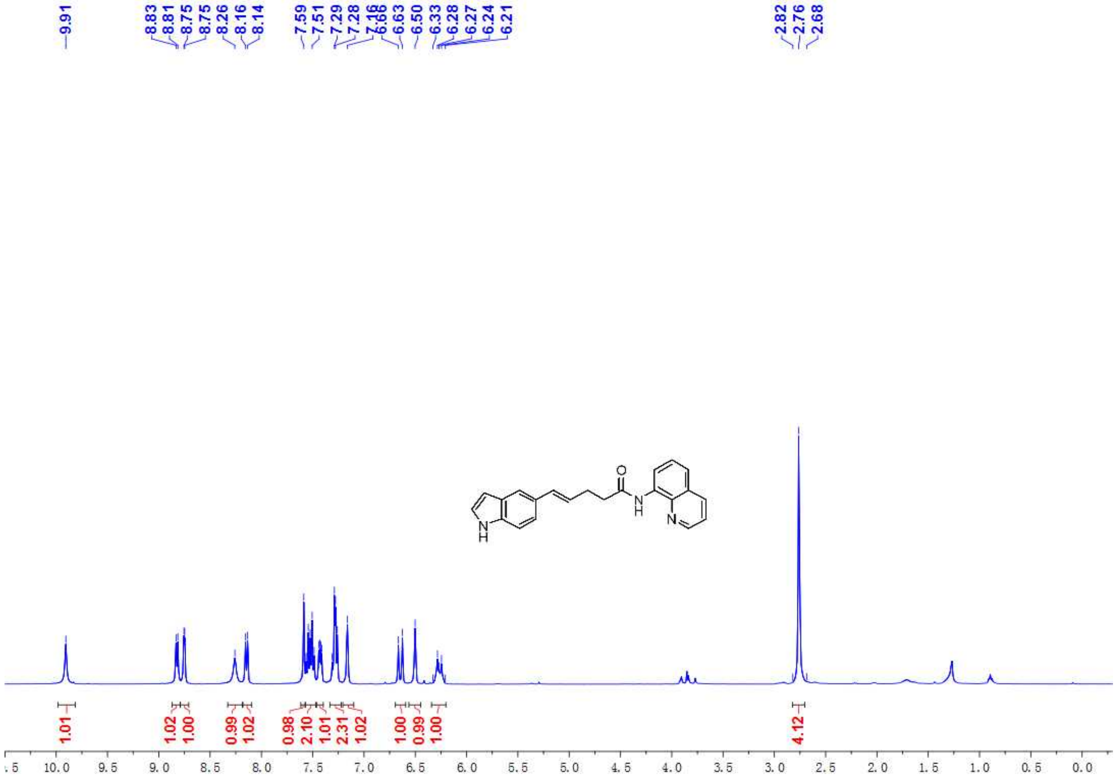

Supplementary Figure 87 ^{13}C NMR (100 MHz, $CDCl_3$) spectrum for 5as

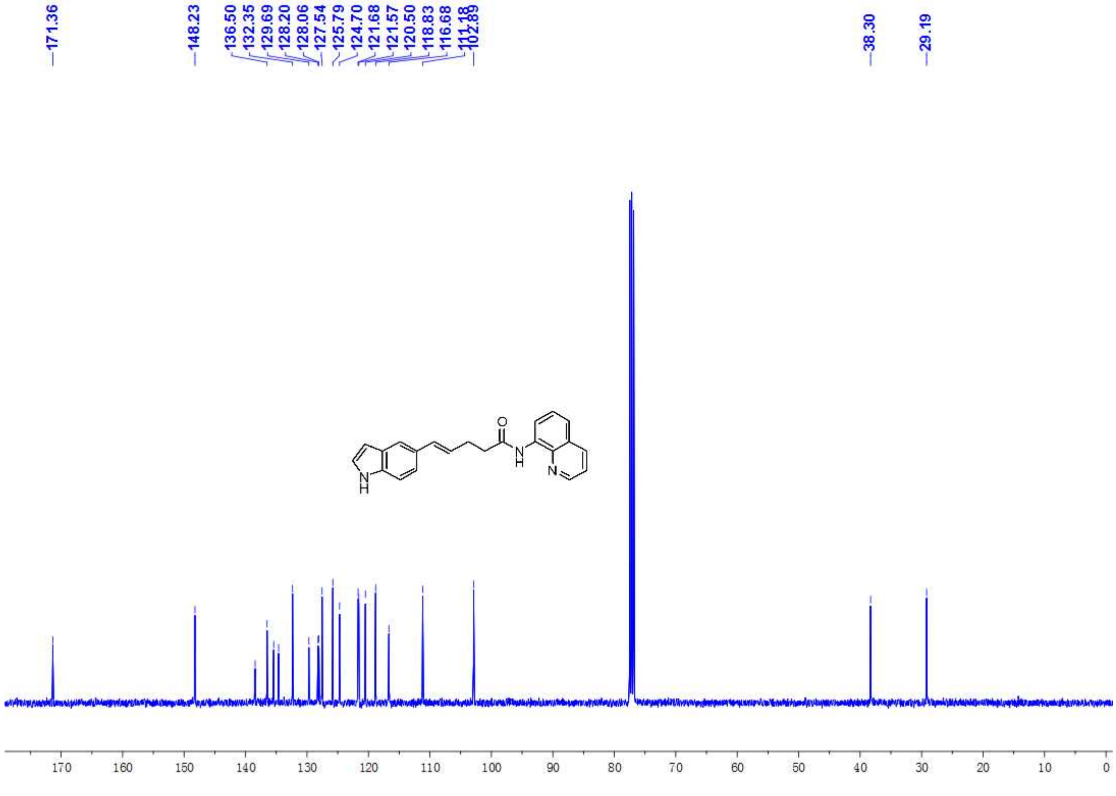

Supplementary Figure 88 ^{1}H NMR (400 MHz, $CDCl_3$) spectrum for 5at

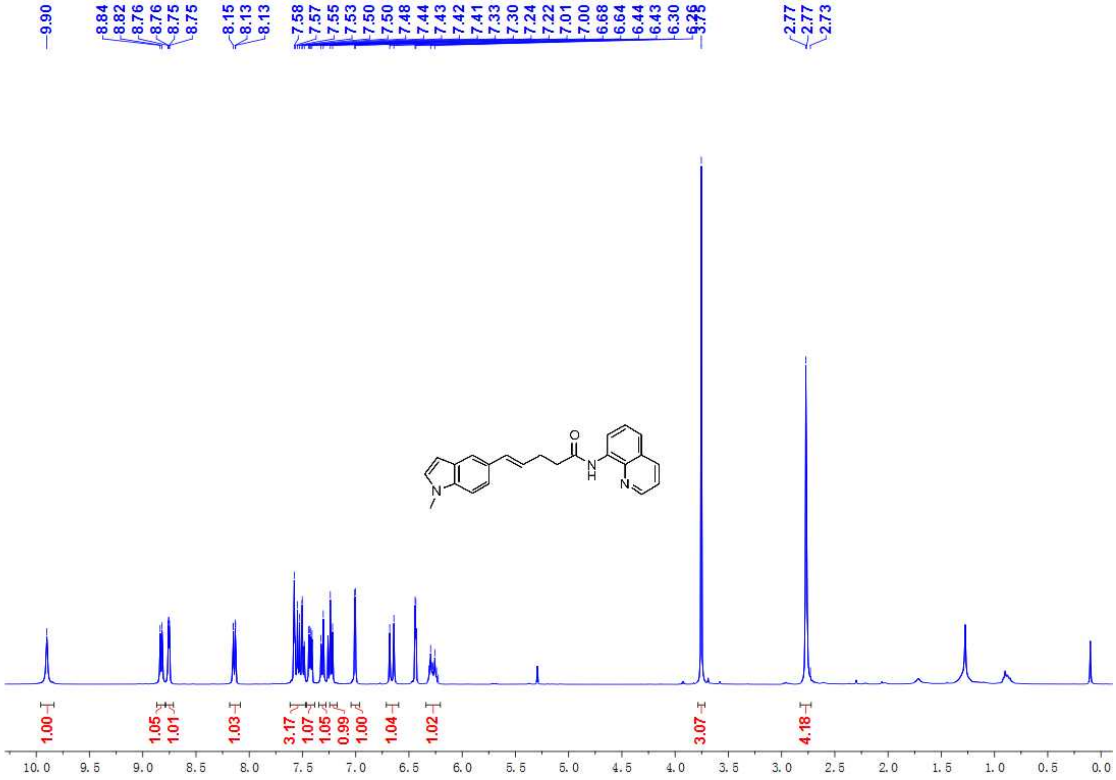

Supplementary Figure 89 ^{13}C NMR (100 MHz, $CDCl_3$) spectrum for 5at

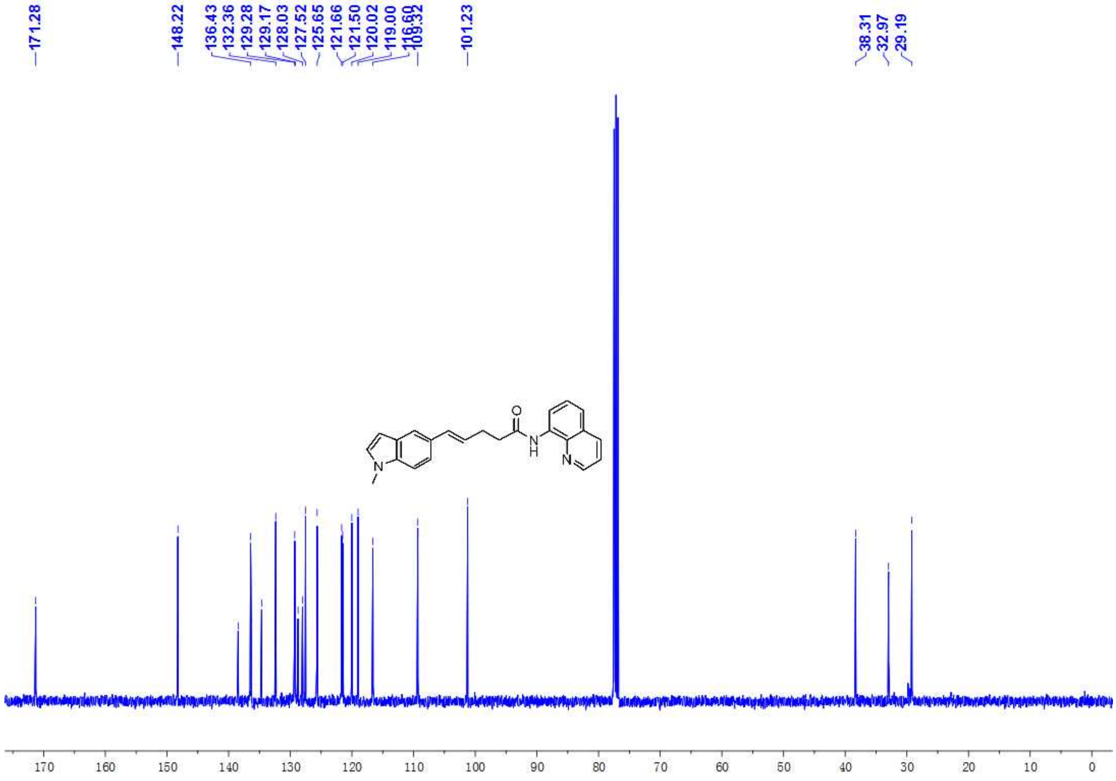

Supplementary Figure 90 ^{1}H NMR (400 MHz, $CDCl_3$) spectrum for 5au

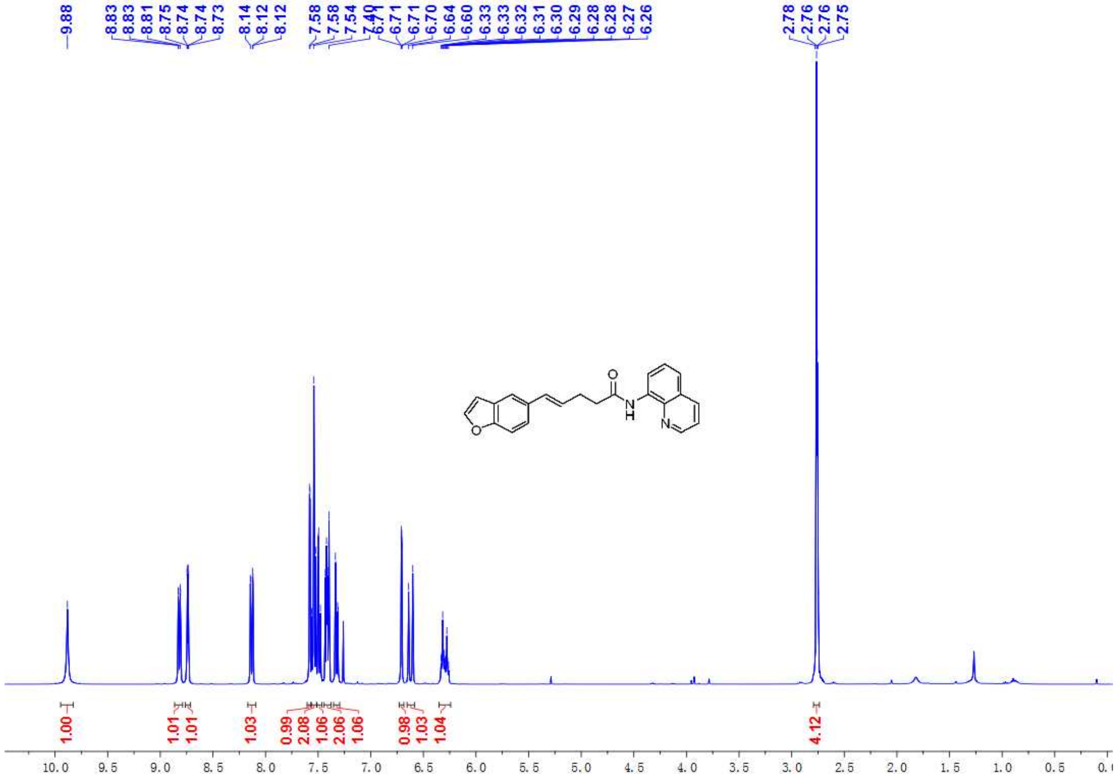

Supplementary Figure 91 ^{13}C NMR (100 MHz, $CDCl_3$) spectrum for 5au

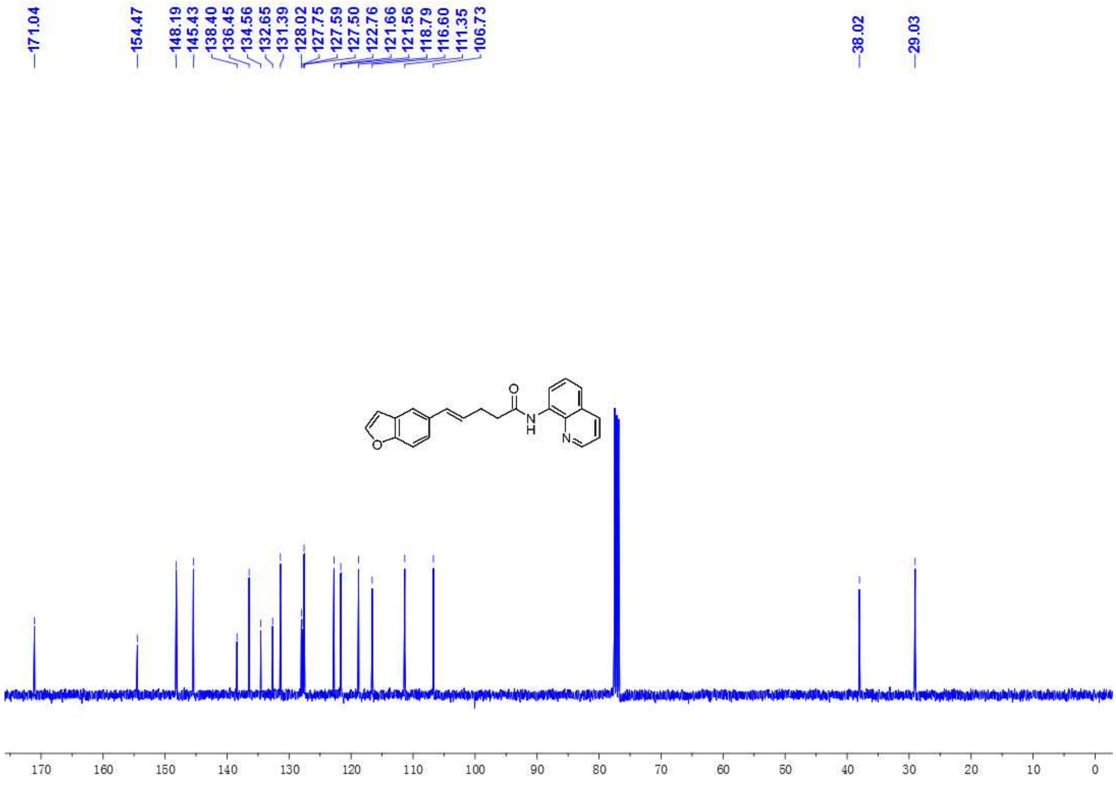

Supplementary Figure 92 ^{1}H NMR (400 MHz, $CDCl_3$) spectrum for 5av

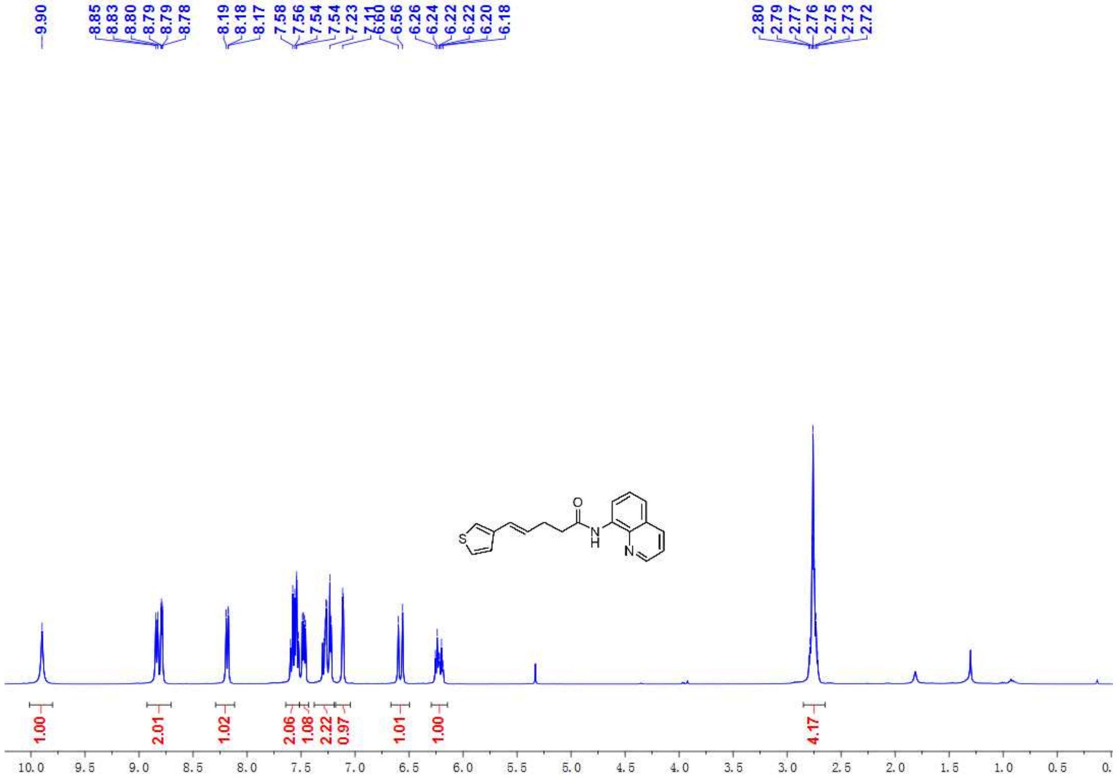

Supplementary Figure 93 ^{13}C NMR (100 MHz, $CDCl_3$) spectrum for 5av

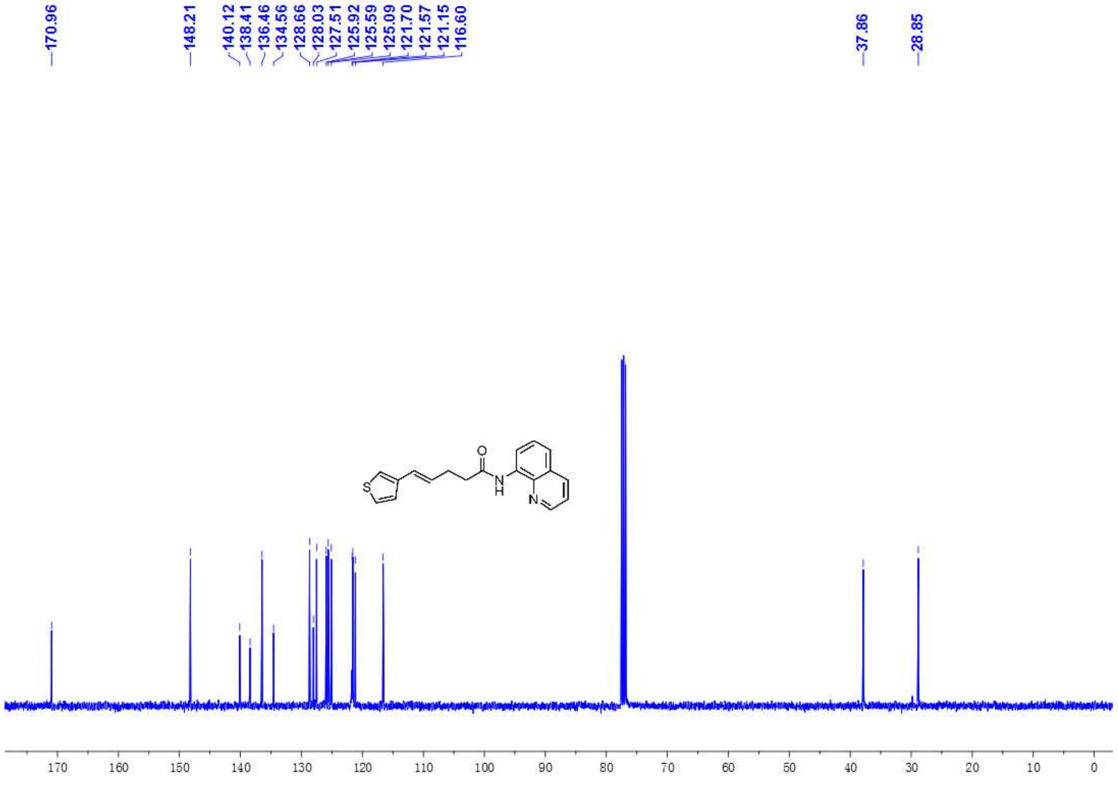

Supplementary Figure 94 ^{1}H NMR (400 MHz, $CDCl_3$) spectrum for 5aw

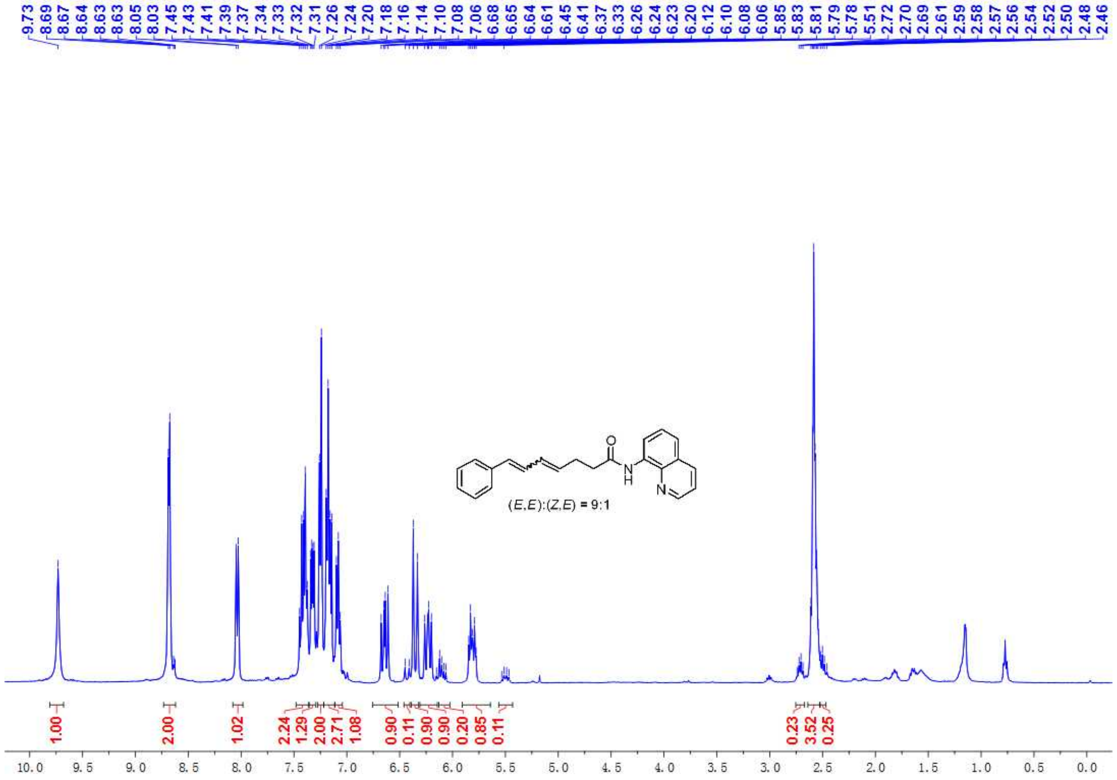

Supplementary Figure 95 ^{13}C NMR (100 MHz, $CDCl_3$) spectrum for 5aw

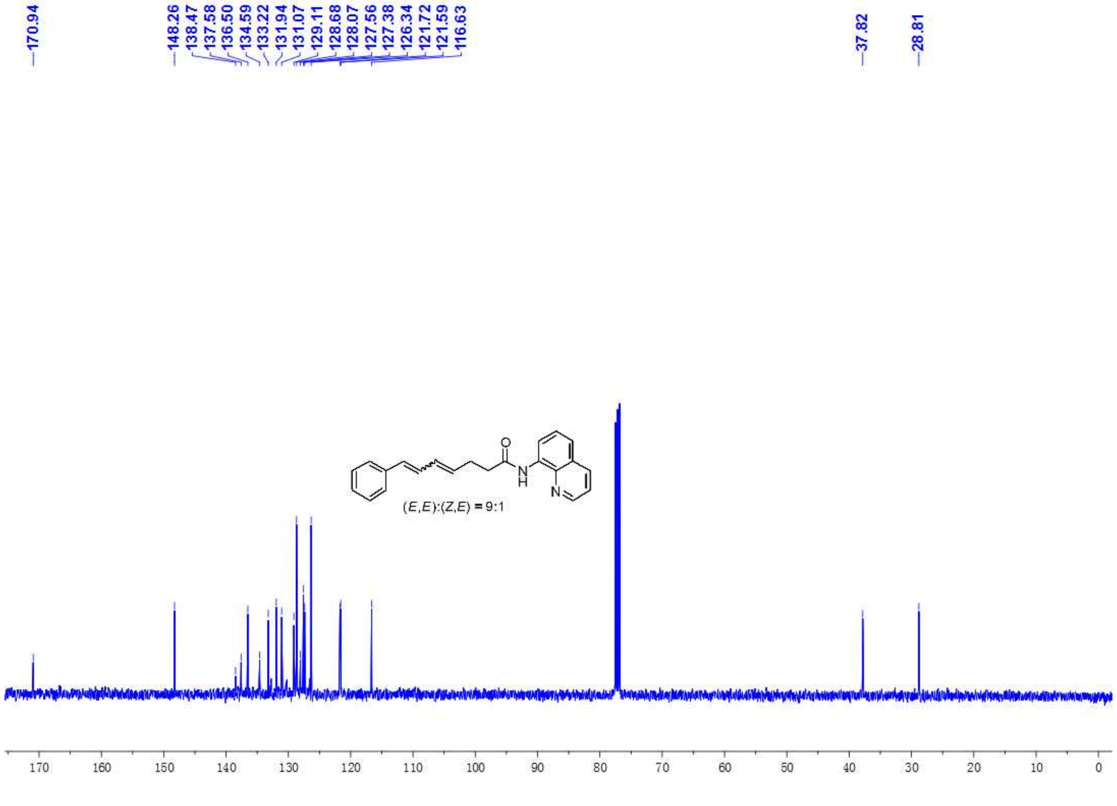

Supplementary Figure 96 ^{1}H NMR (400 MHz, $CDCl_3$) spectrum for 5ax

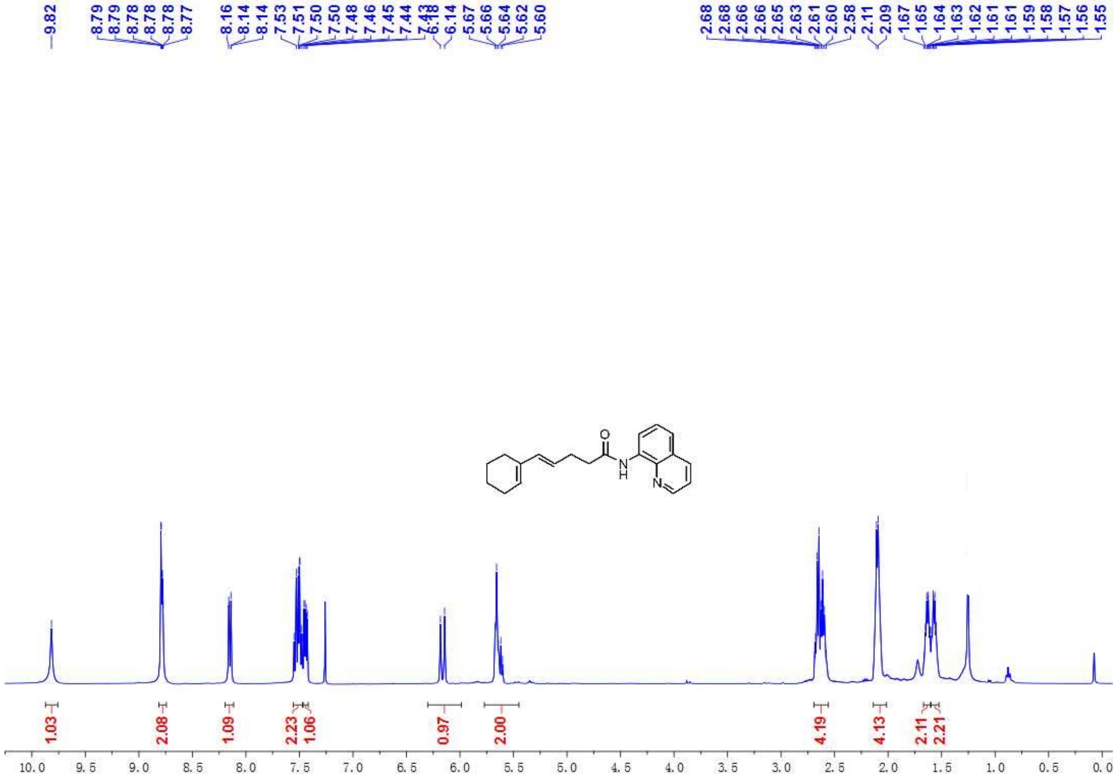

Supplementary Figure 97 ^{13}C NMR (100 MHz, $CDCl_3$) spectrum for 5ax

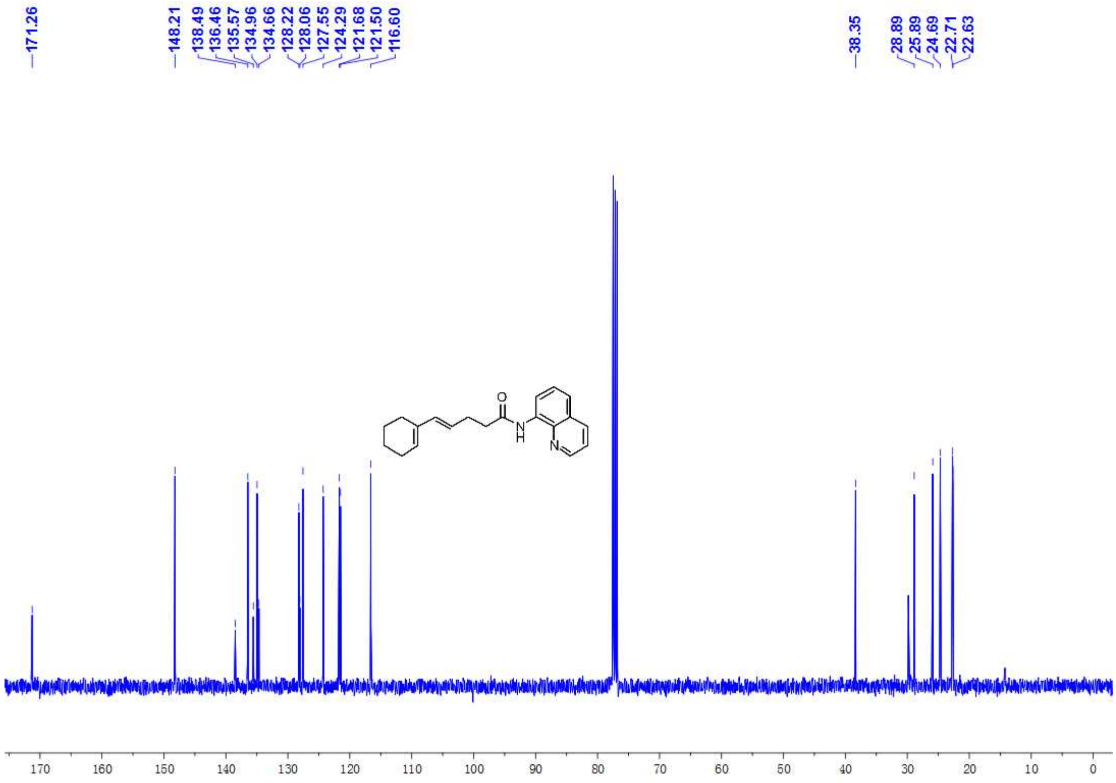

Supplementary Figure 98 ^{1}H NMR (400 MHz, $CDCl_3$) spectrum for 5ba

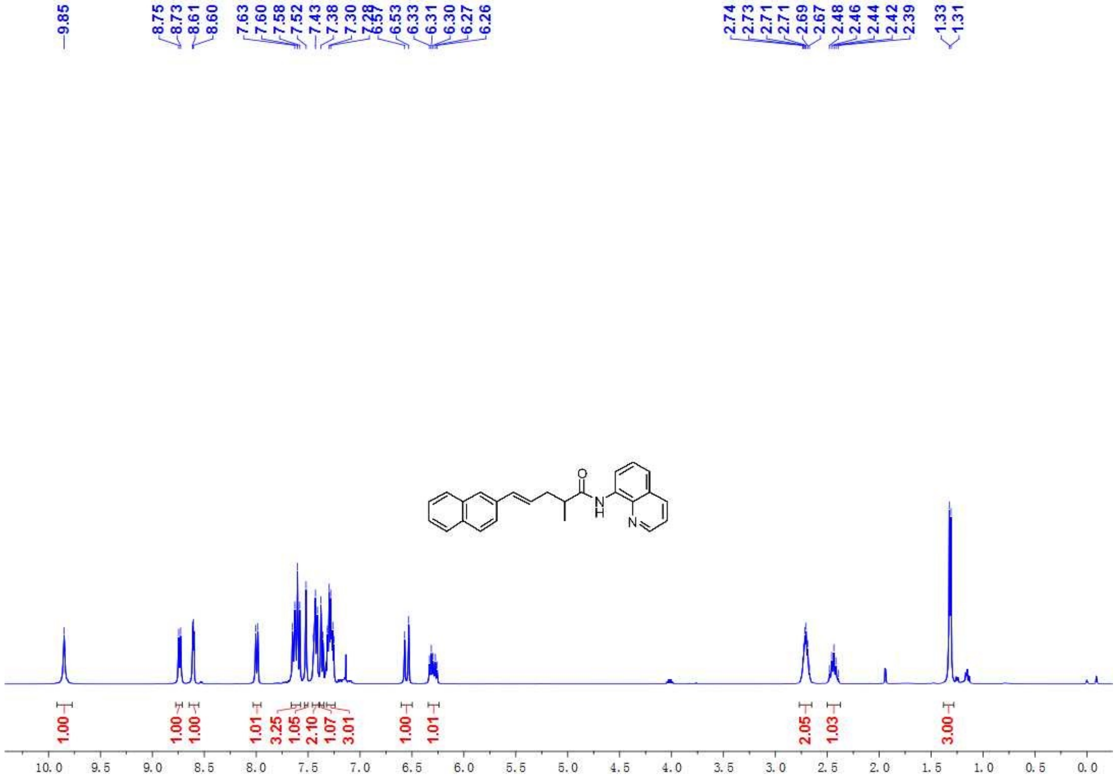

Supplementary Figure 99 ^{13}C NMR (100 MHz, $CDCl_3$) spectrum for 5ba

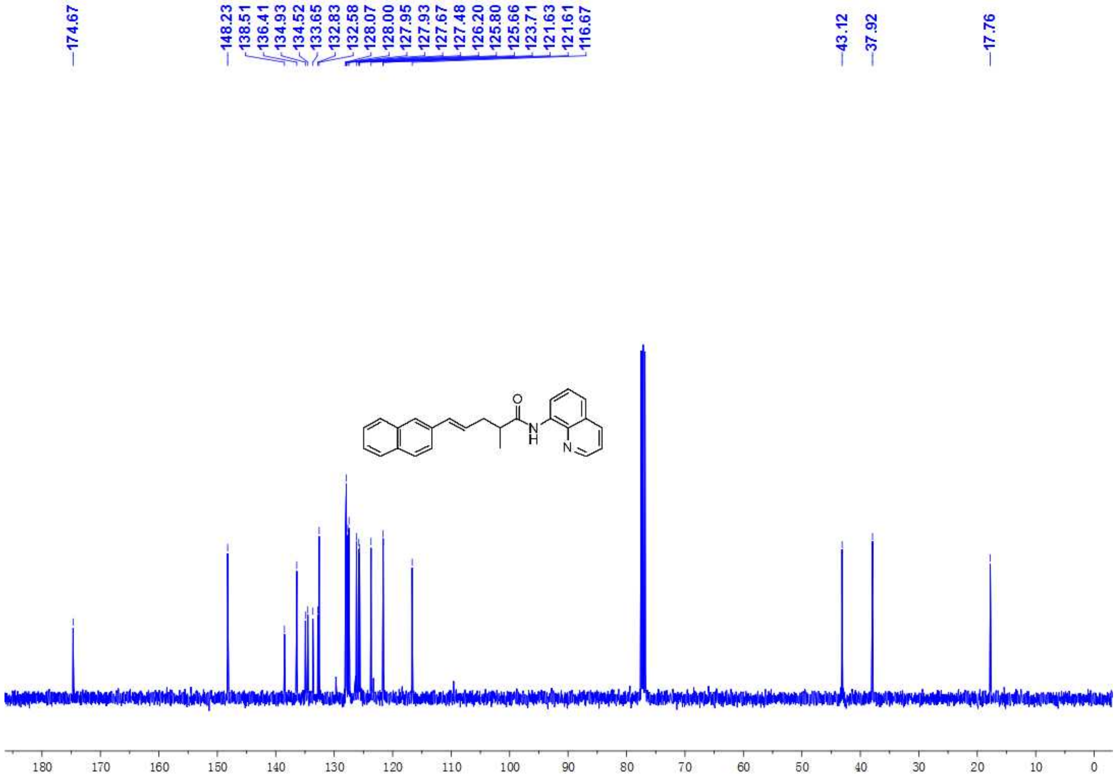

Supplementary Figure 100 ^{1}H NMR (400 MHz, $CDCl_3$) spectrum for 5ca

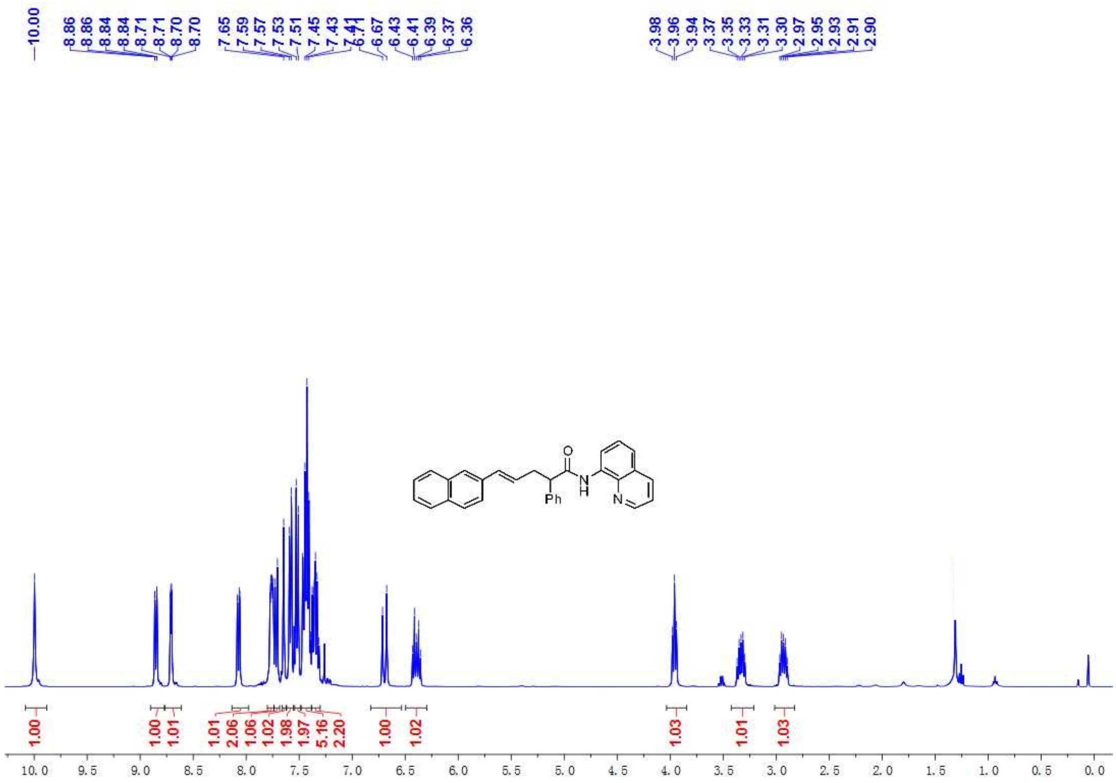

Supplementary Figure 101 ^{13}C NMR (100 MHz, $CDCl_3$) spectrum for 5ca

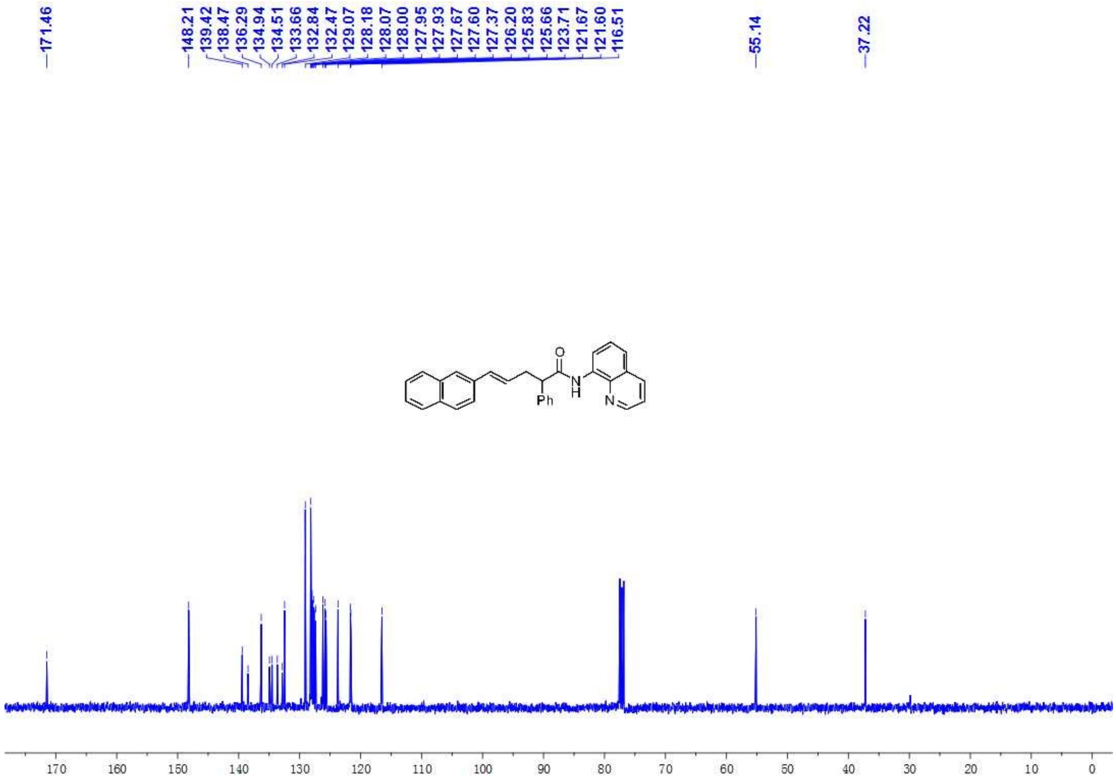

Supplementary Figure 102 ^{1}H NMR (400 MHz, $CDCl_3$) spectrum for 5da

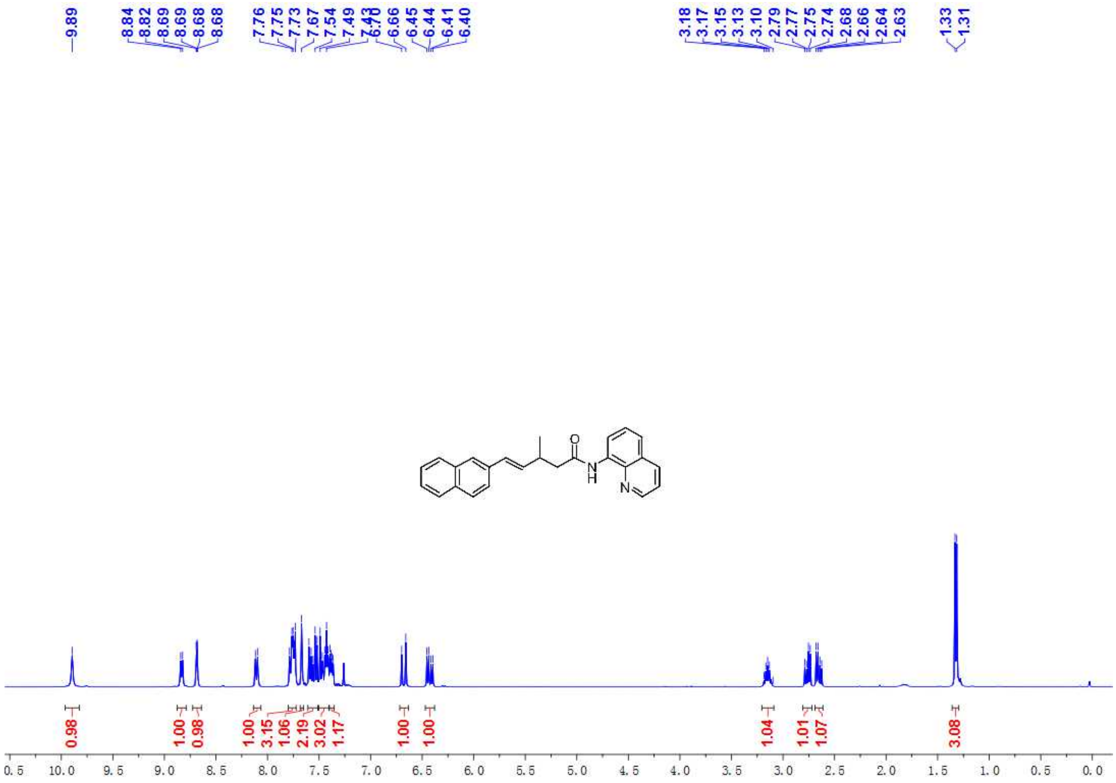

Supplementary Figure 103 ^{13}C NMR (100 MHz, $CDCl_3$) spectrum for 5da

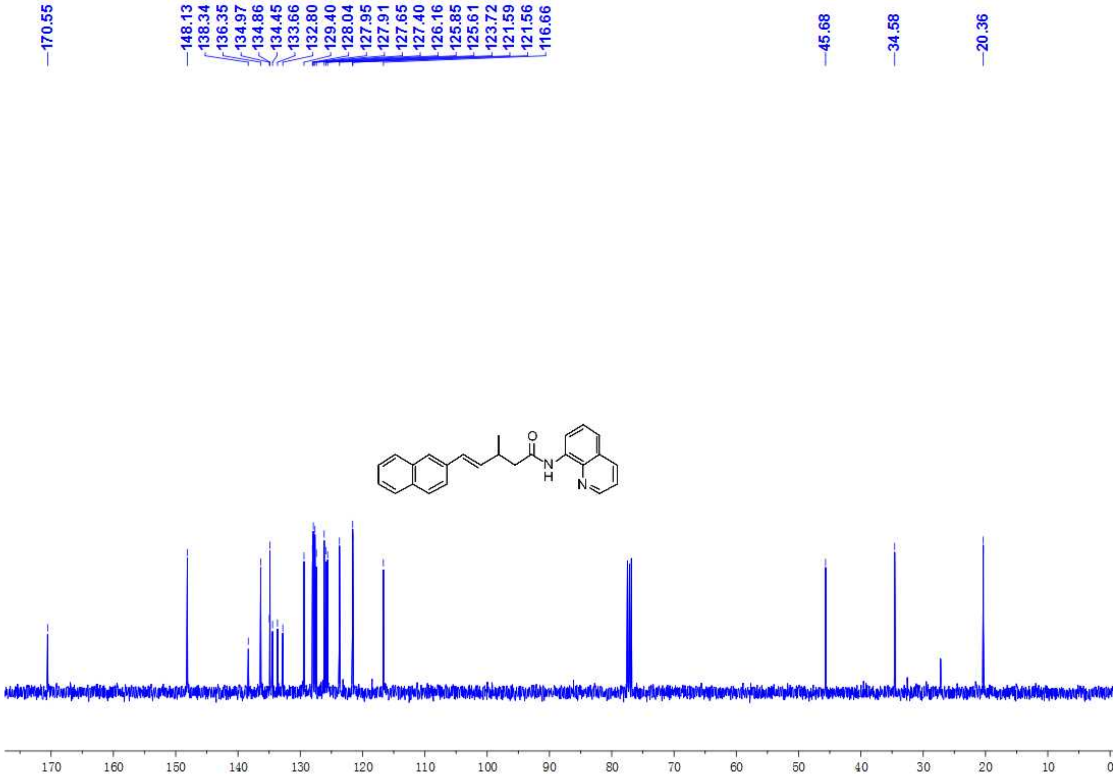

Supplementary Figure 104 ^{1}H NMR (400 MHz, CDCl$_3$) spectrum for 5ea

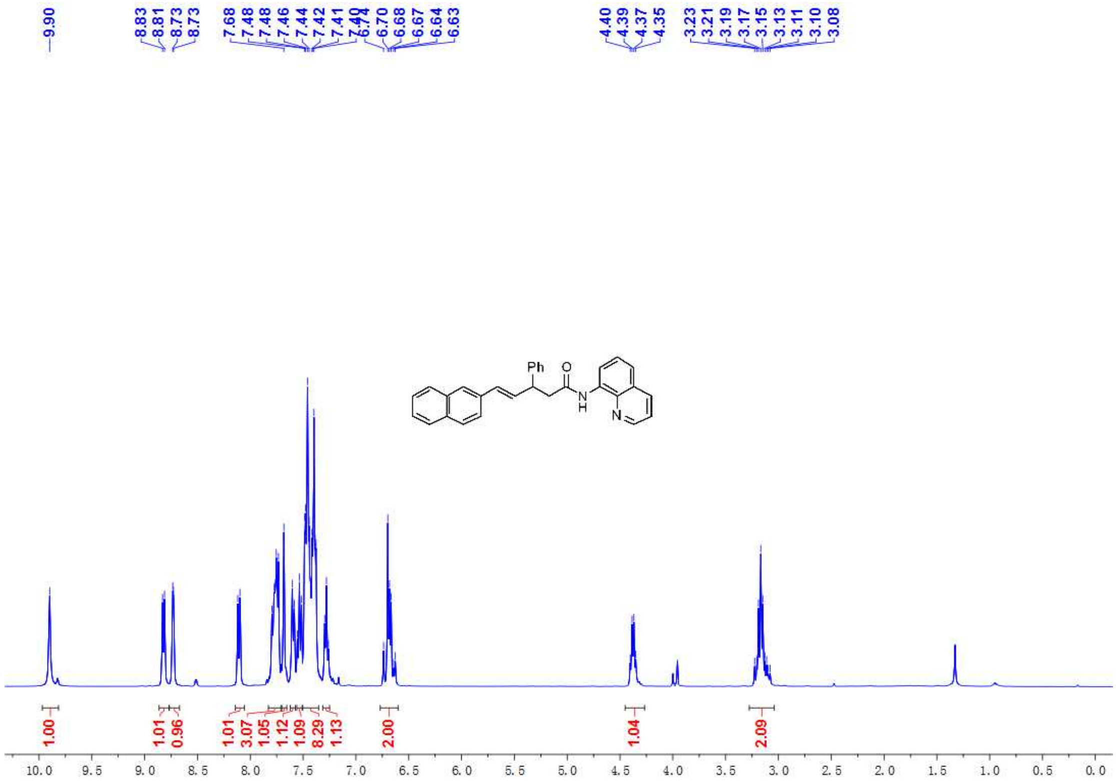

Supplementary Figure 105 ^{13}C NMR (100 MHz, CDCl$_3$) spectrum for 5ea

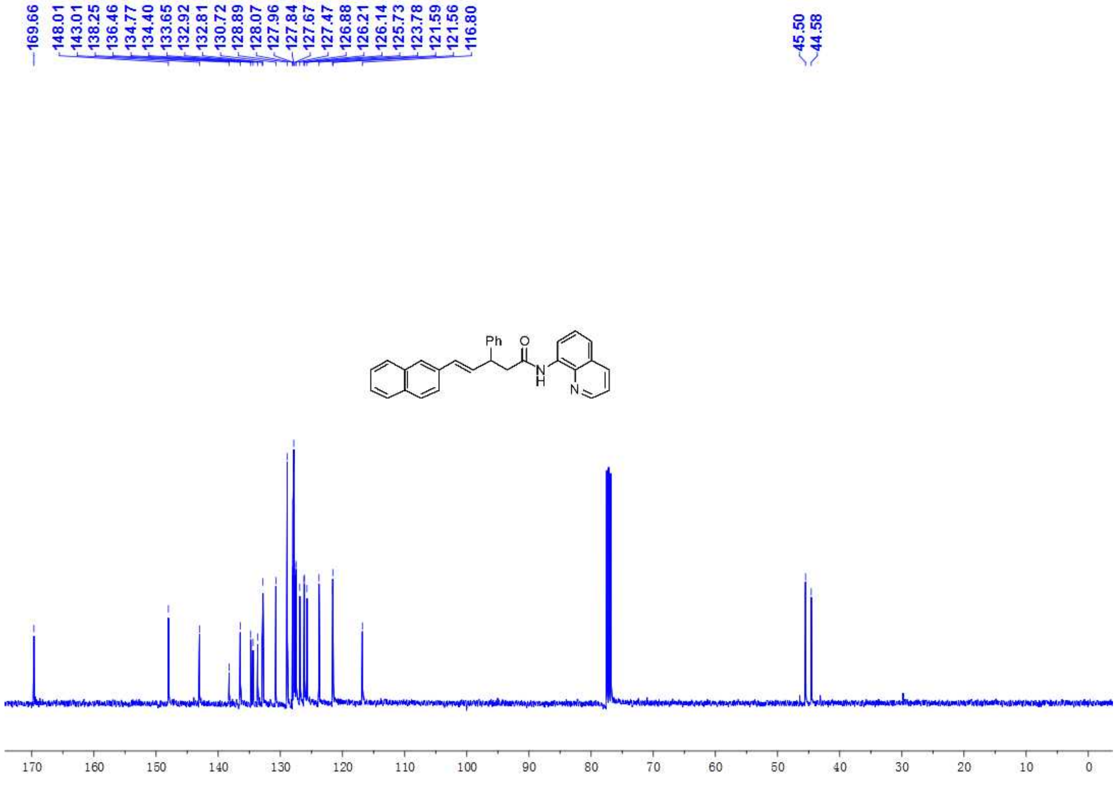

Supplementary Figure 106 ^{1}H NMR (400 MHz, $CDCl_3$) spectrum for 5fa

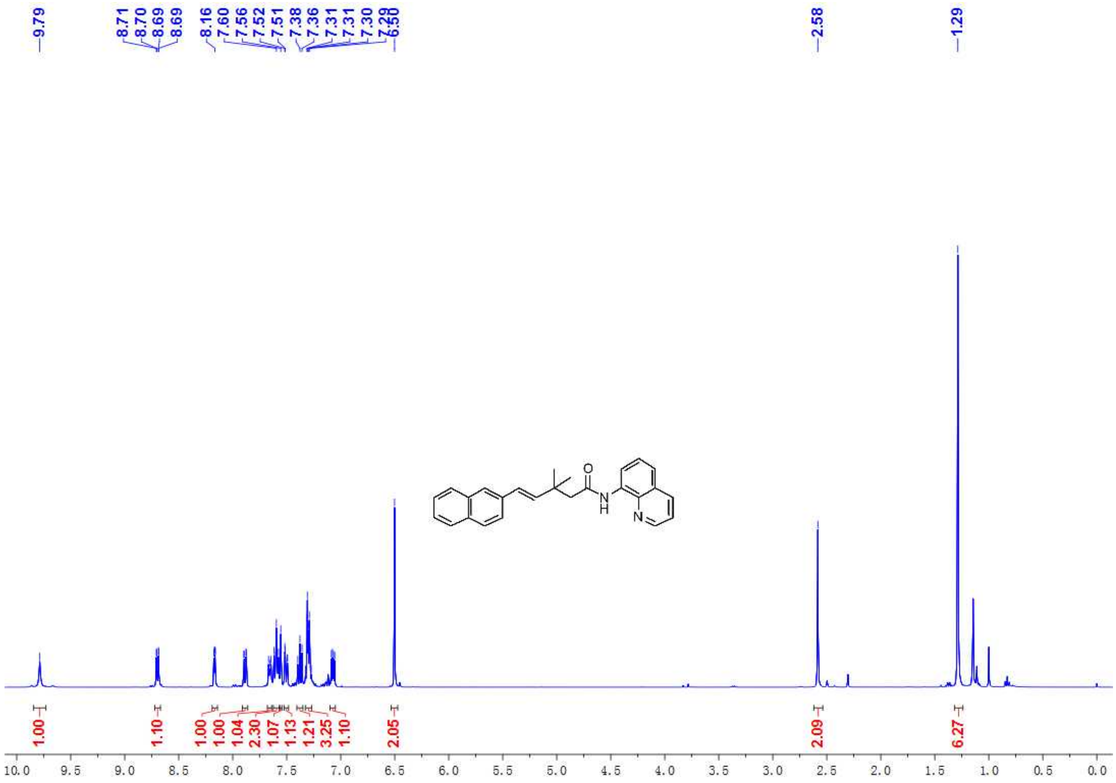

Supplementary Figure 107 ^{13}C NMR (100 MHz, $CDCl_3$) spectrum for 5fa

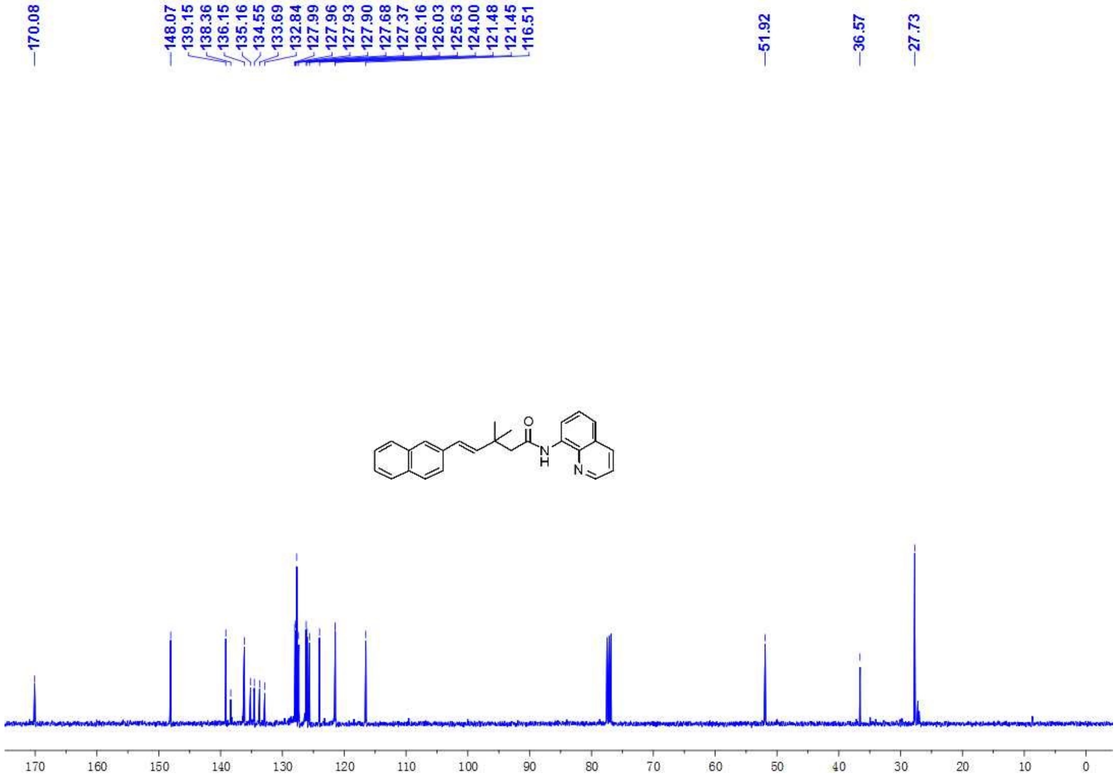

Supplementary Figure 108 ^{1}H NMR (400 MHz, $CDCl_3$) spectrum for 5ga

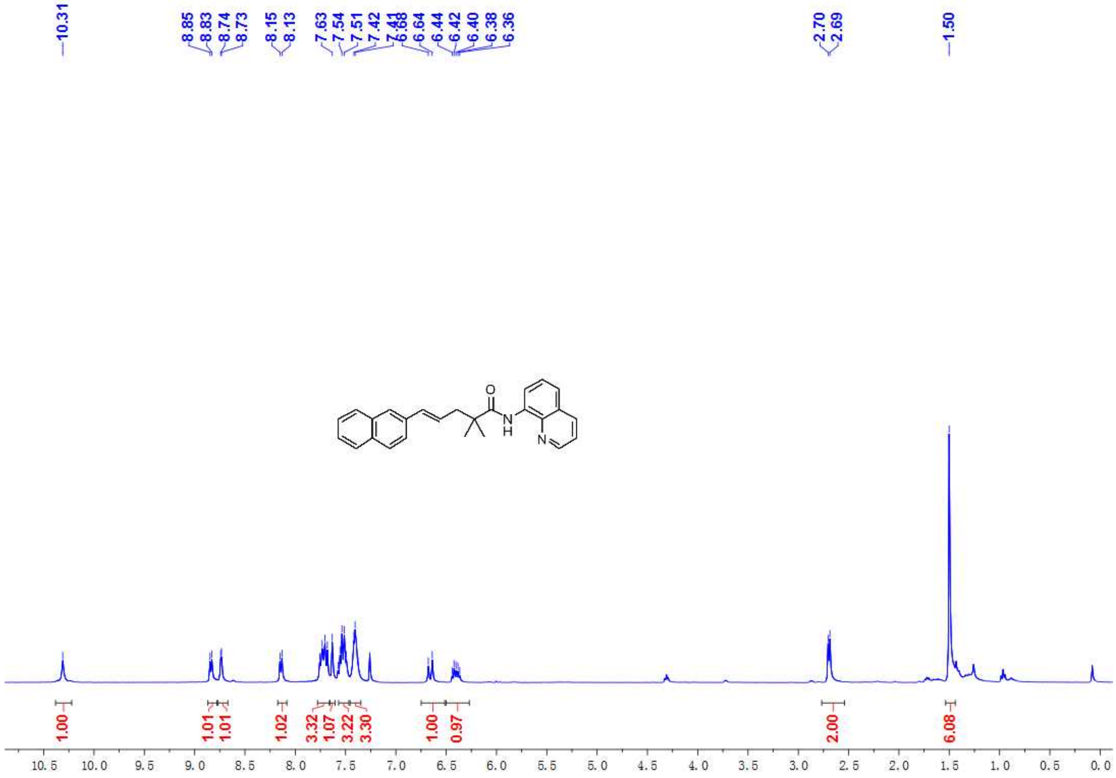

Supplementary Figure 109 ^{13}C NMR (100 MHz, $CDCl_3$) spectrum for 5ga

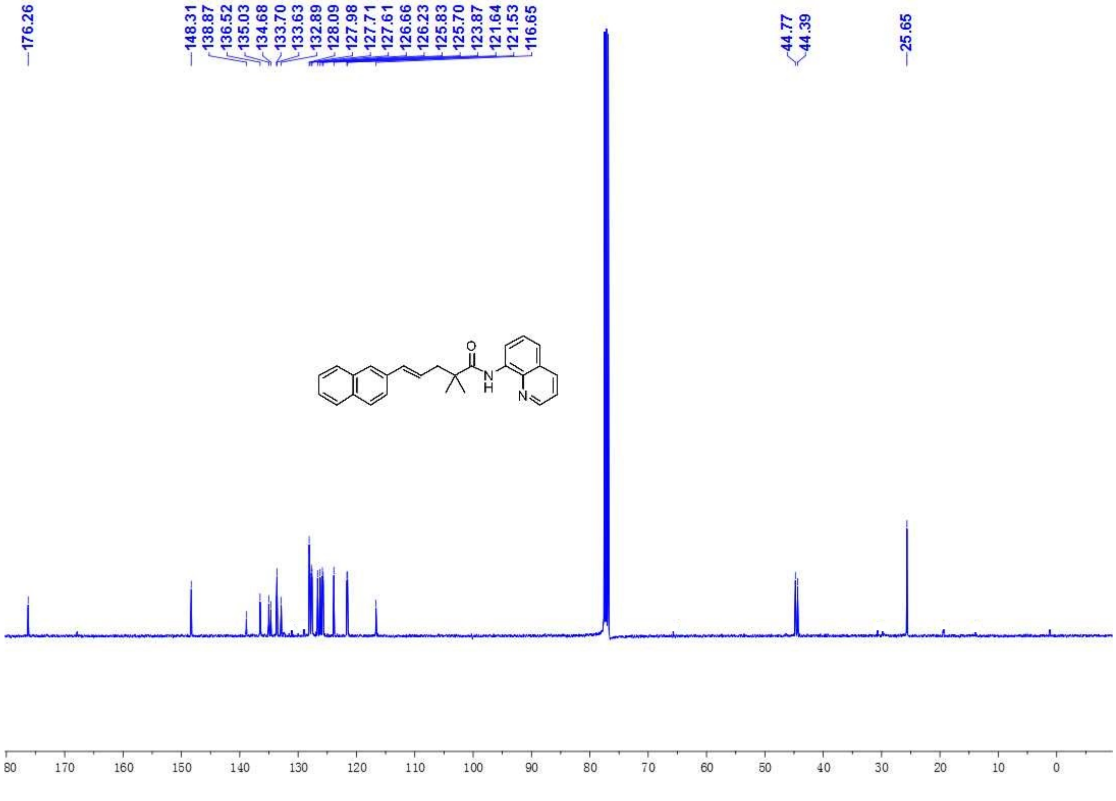

Supplementary Figure 110 ^{1}H NMR (400 MHz, $CDCl_3$) spectrum for 5ha

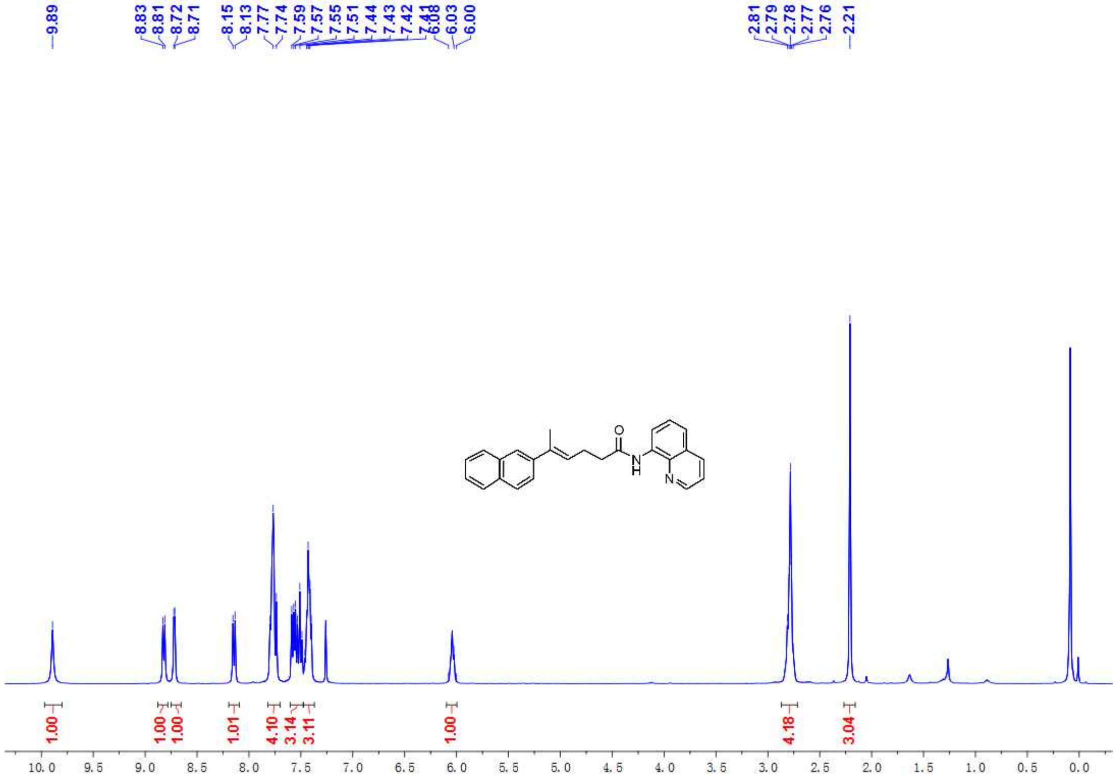

Supplementary Figure 111 ^{13}C NMR (100 MHz, $CDCl_3$) spectrum for 5ha

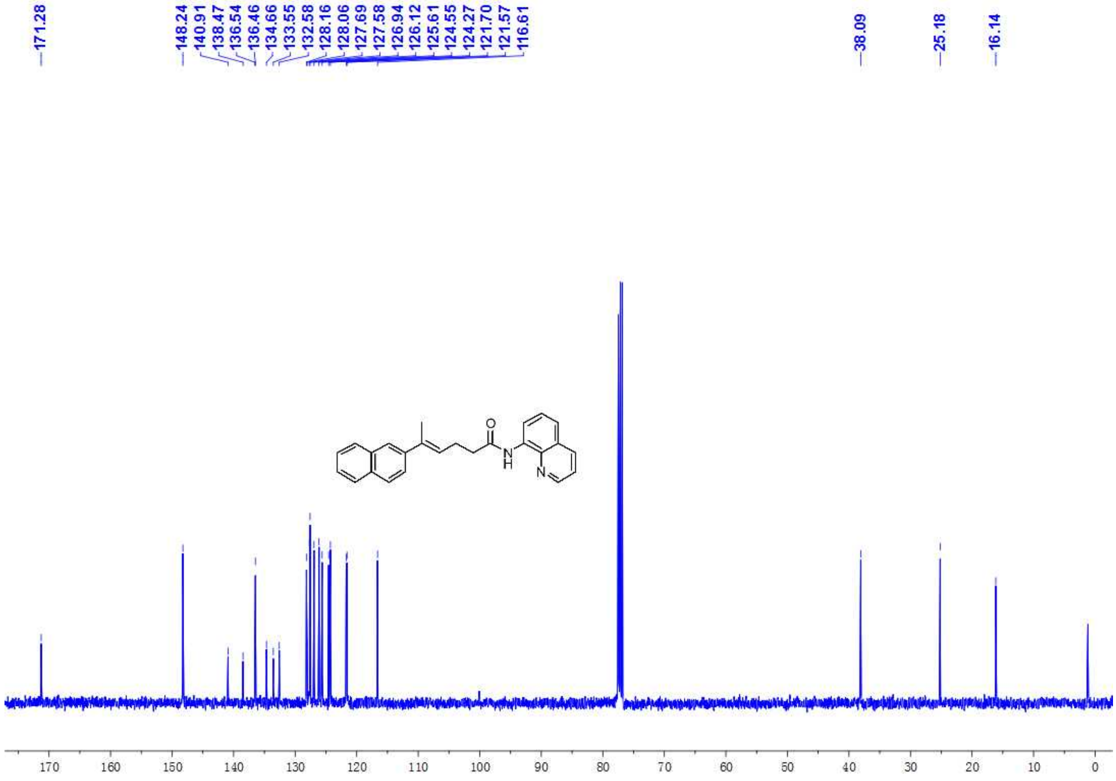

Supplementary Figure 112 ^{1}H NMR (400 MHz, $CDCl_3$) spectrum for 5ia

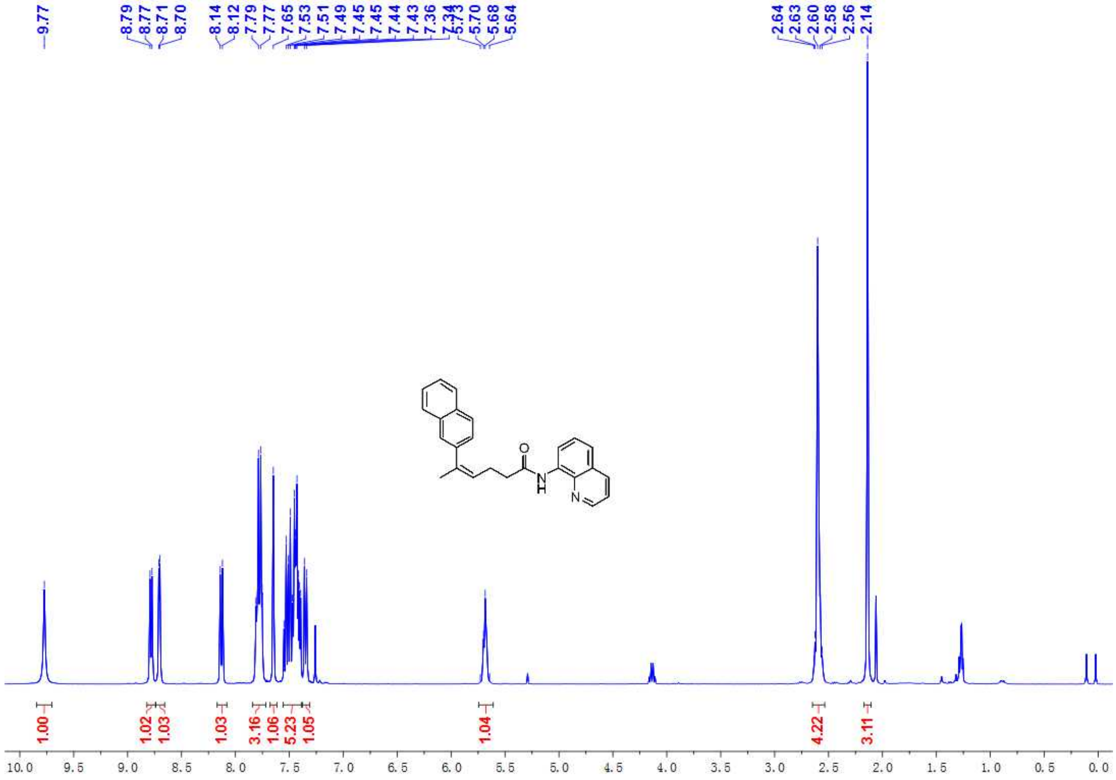

Supplementary Figure 113 ^{13}C NMR (100 MHz, $CDCl_3$) spectrum for 5ia

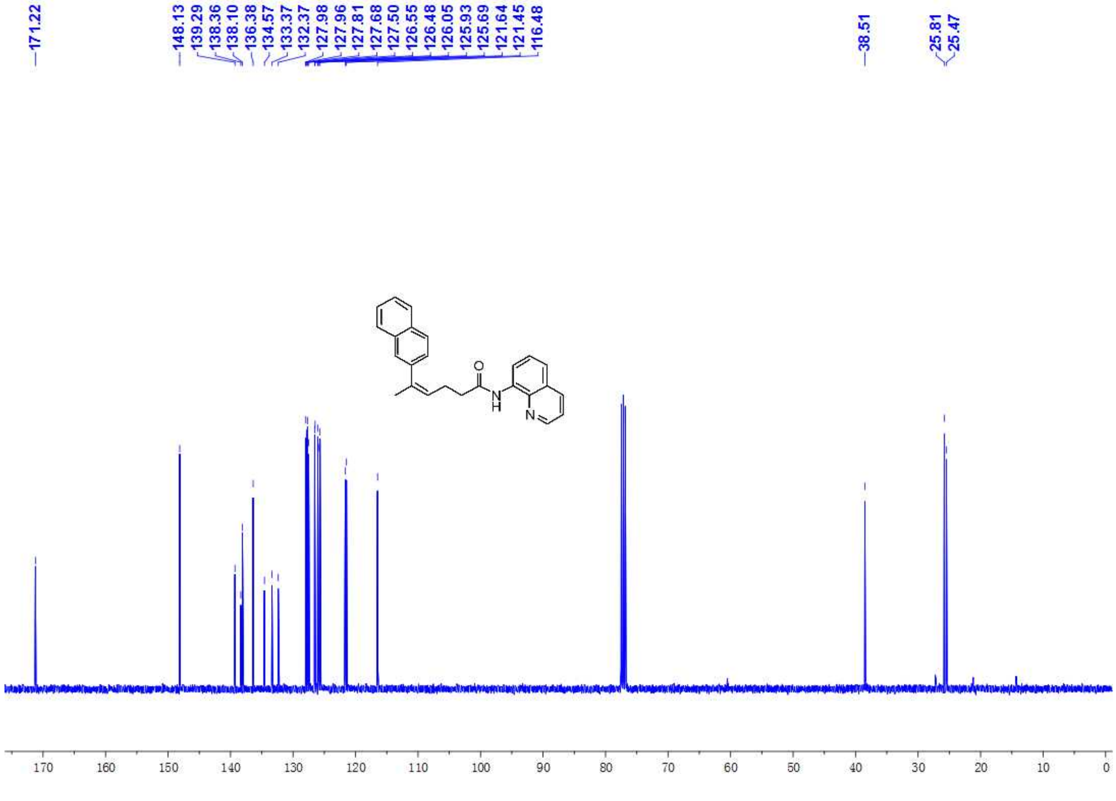

Supplementary Figure 114 ^{1}H NMR (400 MHz, $CDCl_3$) spectrum for 5ja

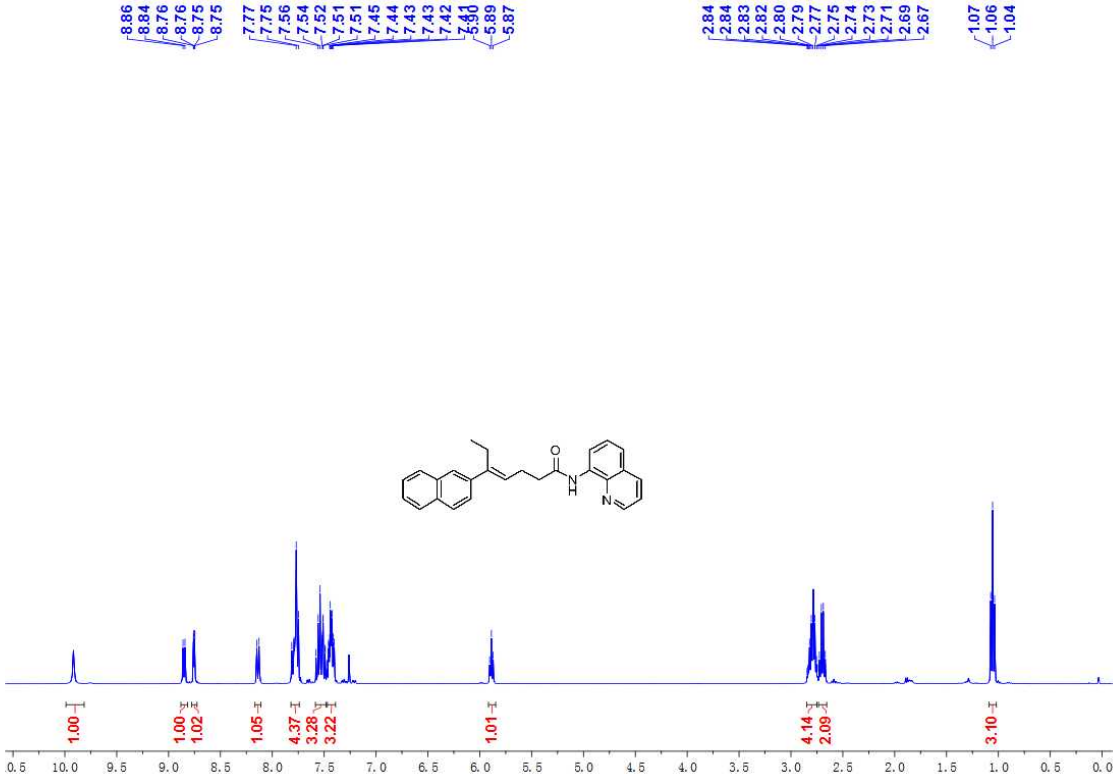

Supplementary Figure 115 ^{13}C NMR (100 MHz, $CDCl_3$) spectrum for 5ja

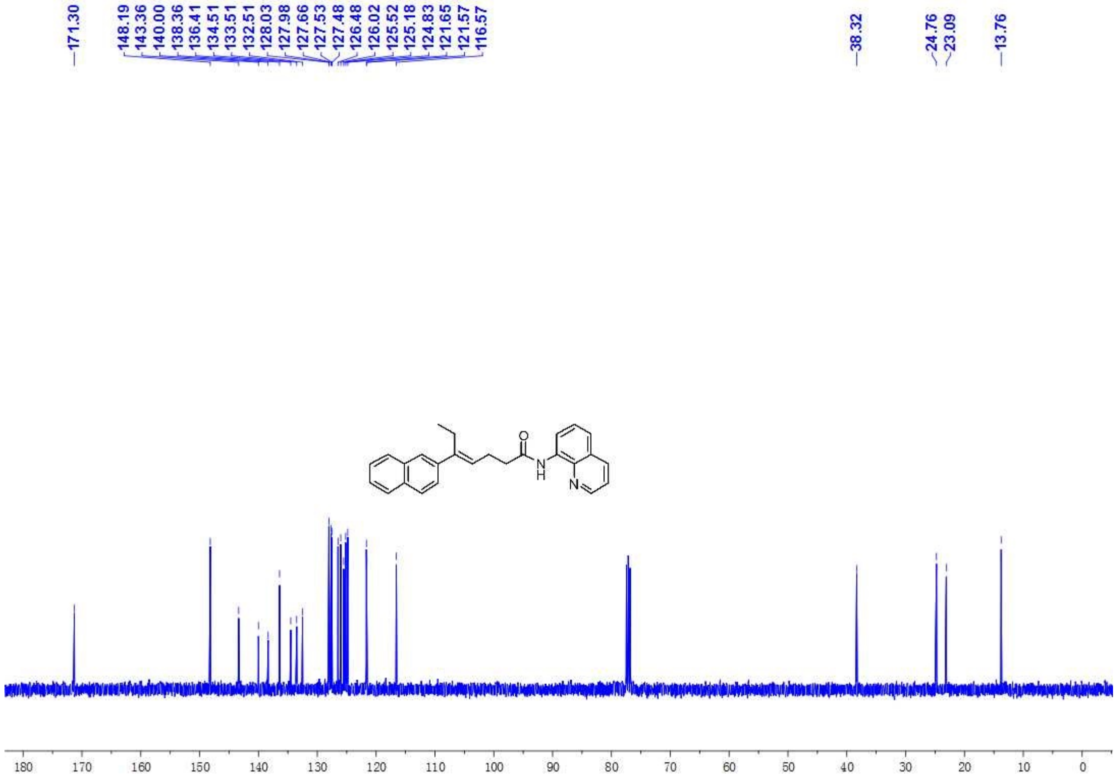

Supplementary Figure 116 ^{1}H NMR (400 MHz, $CDCl_3$) spectrum for 5ka

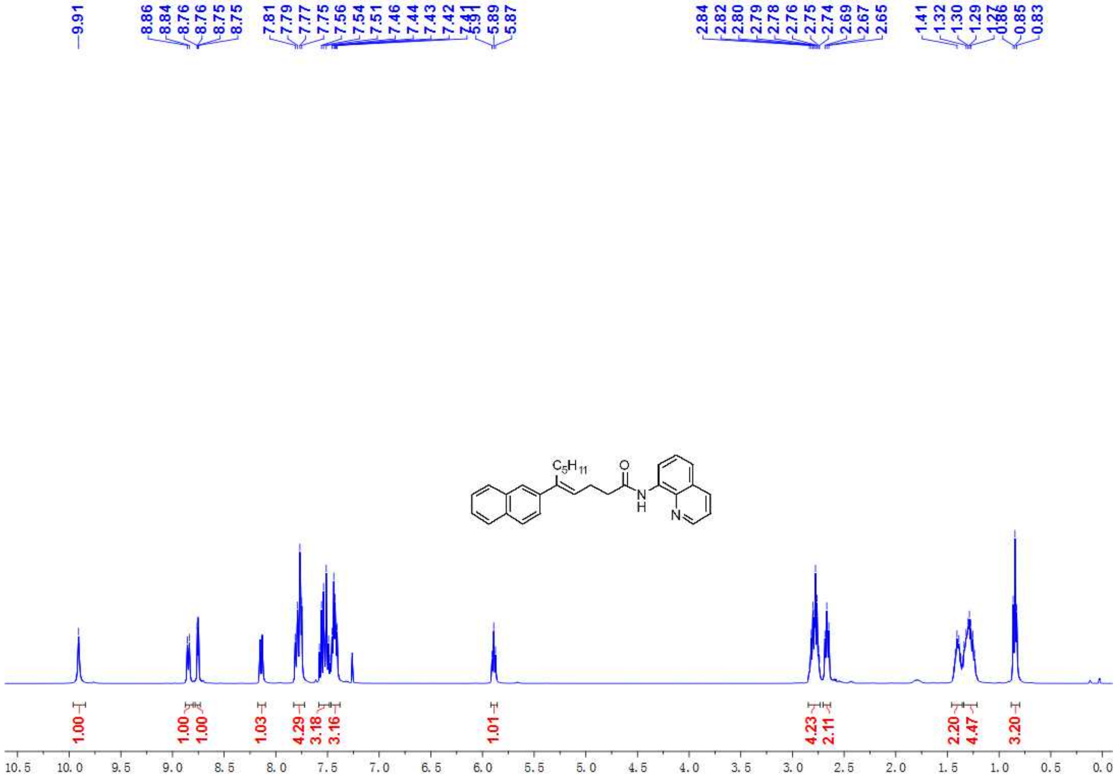

Supplementary Figure 117 ^{13}C NMR (100 MHz, $CDCl_3$) spectrum for 5ka

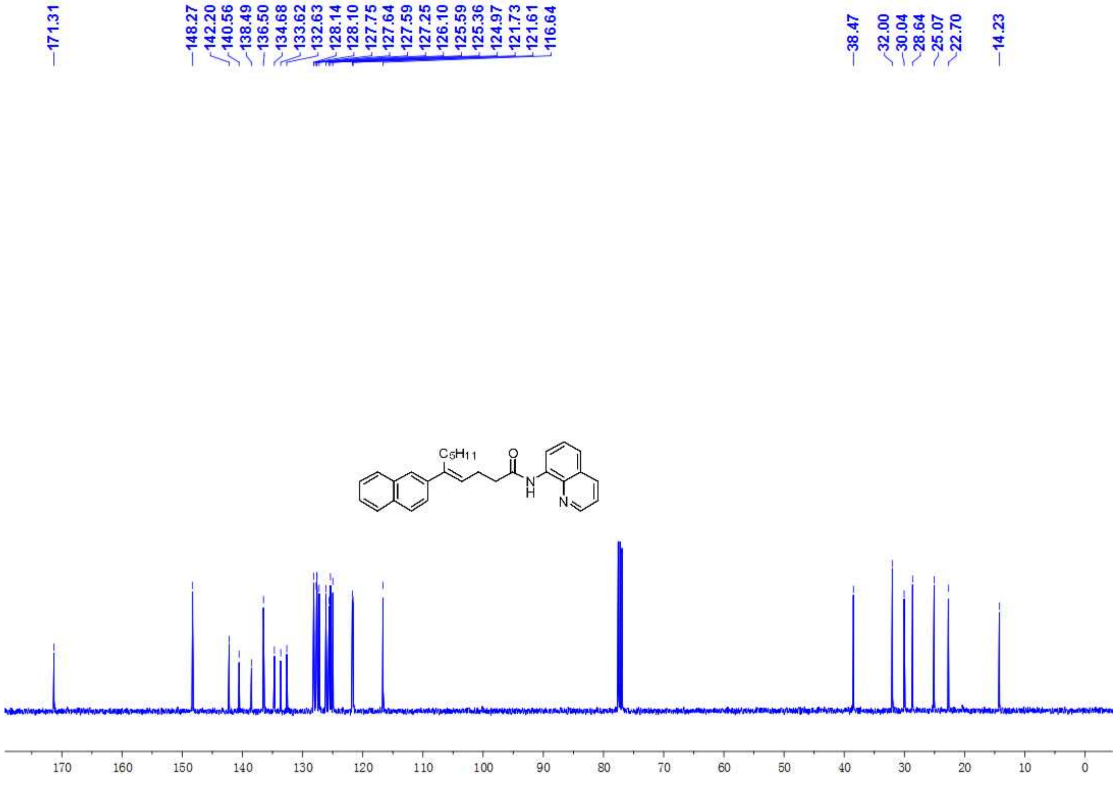

Supplementary Figure 118 ^{1}H NMR (400 MHz, $CDCl_3$) spectrum for 5la

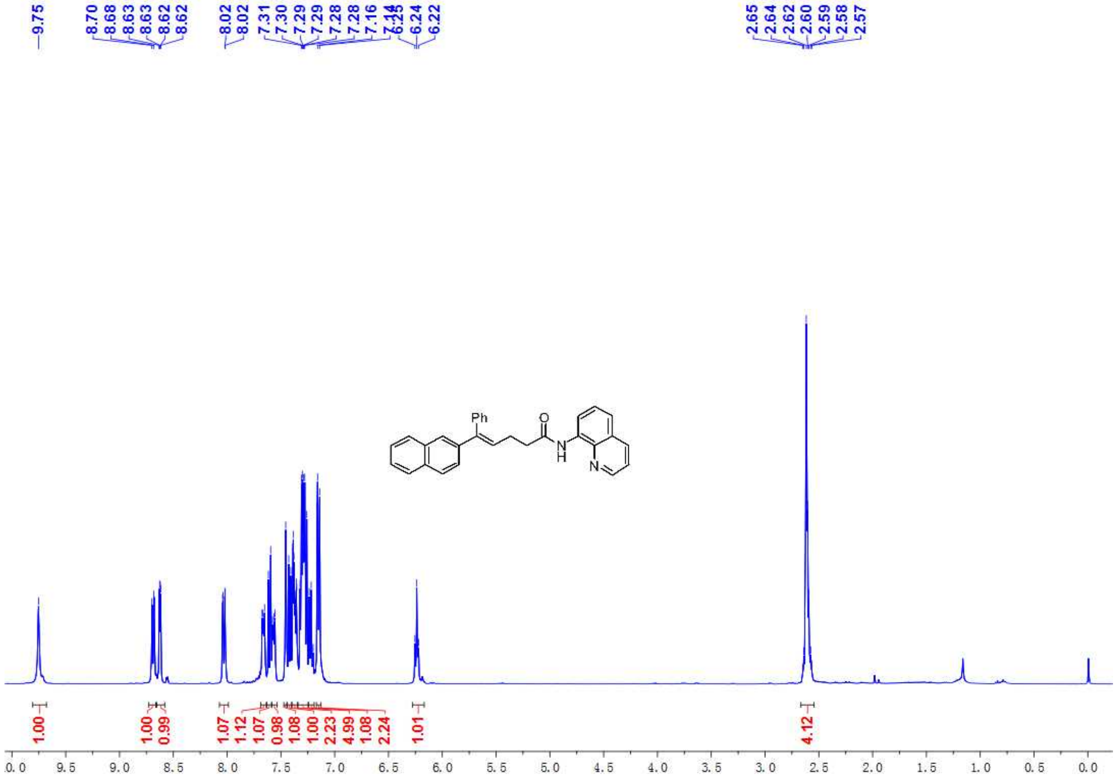

Supplementary Figure 119 ^{13}C NMR (100 MHz, $CDCl_3$) spectrum for 5la

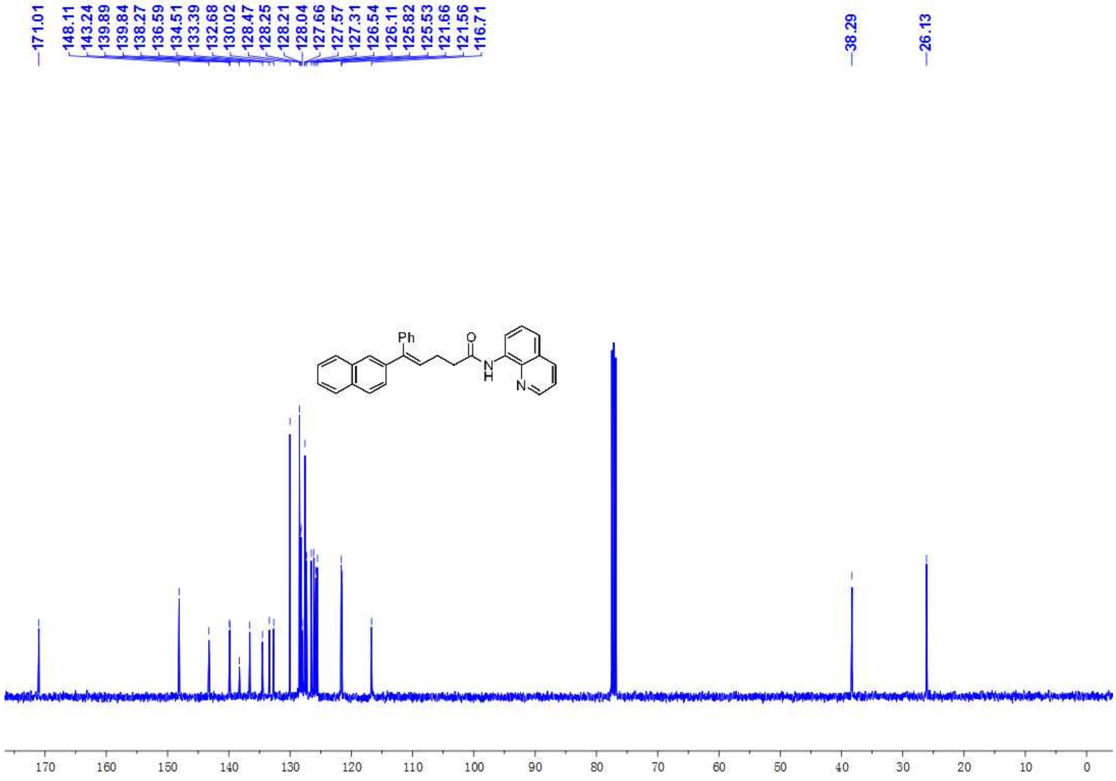

Supplementary Figure 120 ^{1}H NMR (400 MHz, $CDCl_3$) spectrum for 5ma

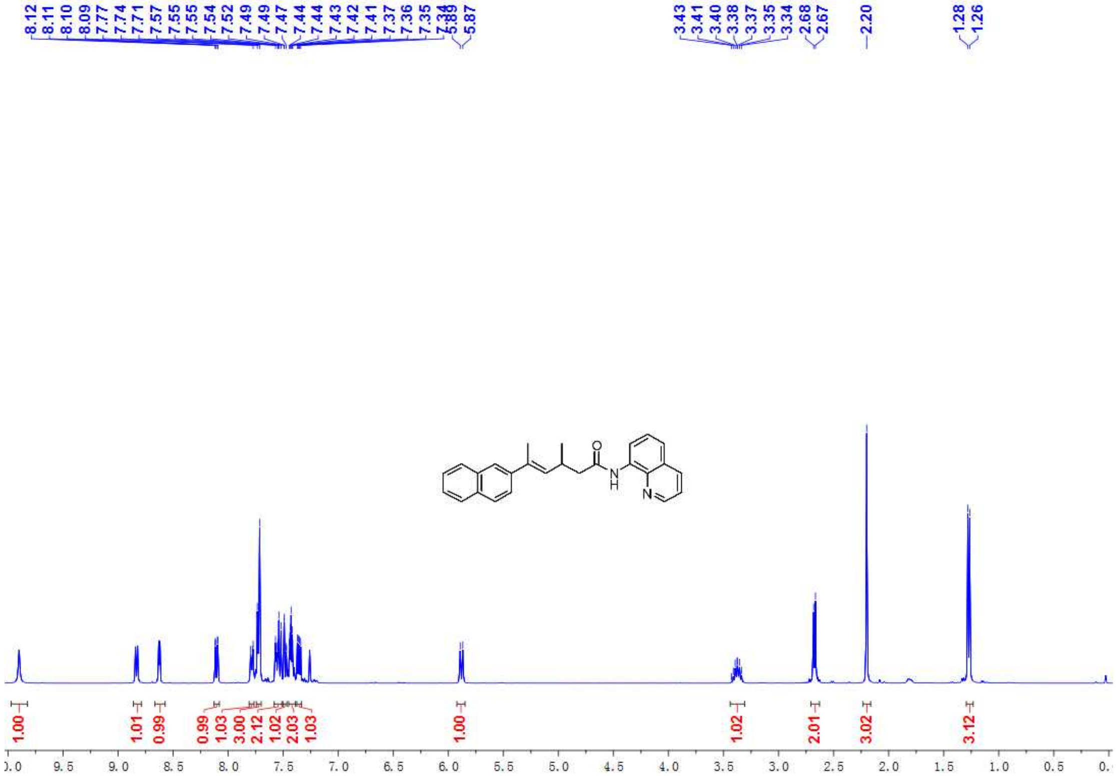

Supplementary Figure 121 ^{13}C NMR (100 MHz, $CDCl_3$) spectrum for 5ma

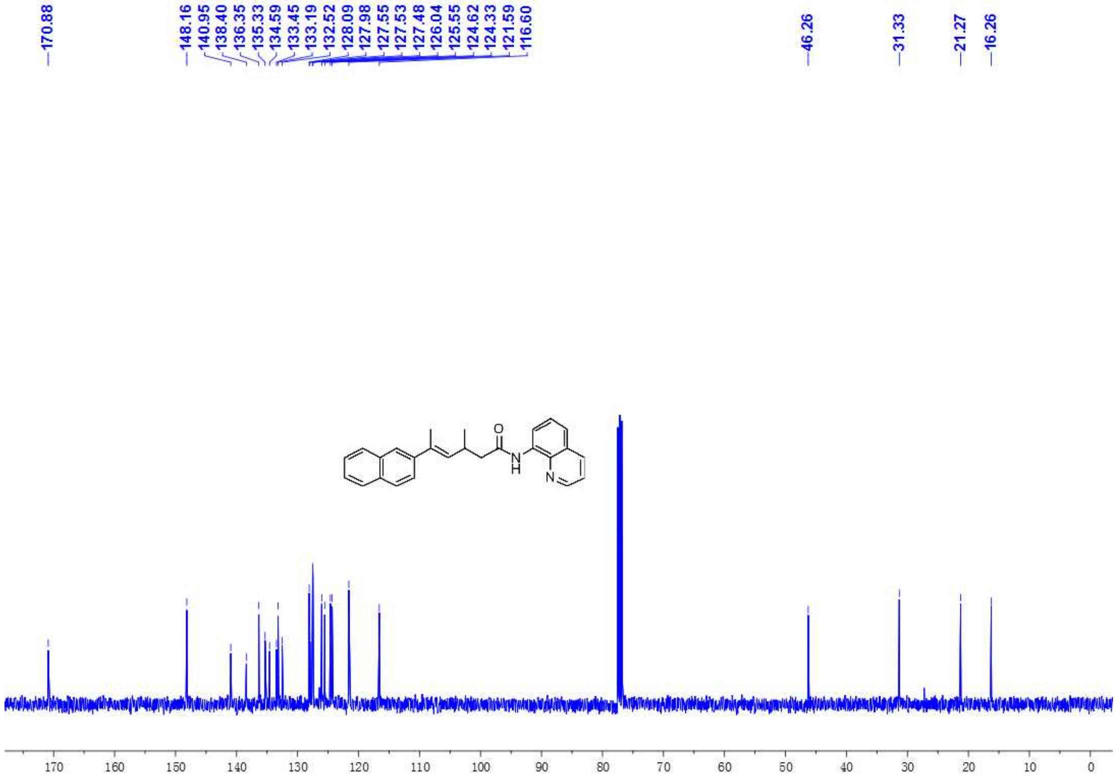

Supplementary Figure 122 ^{1}H NMR (400 MHz, $CDCl_3$) spectrum for 5na

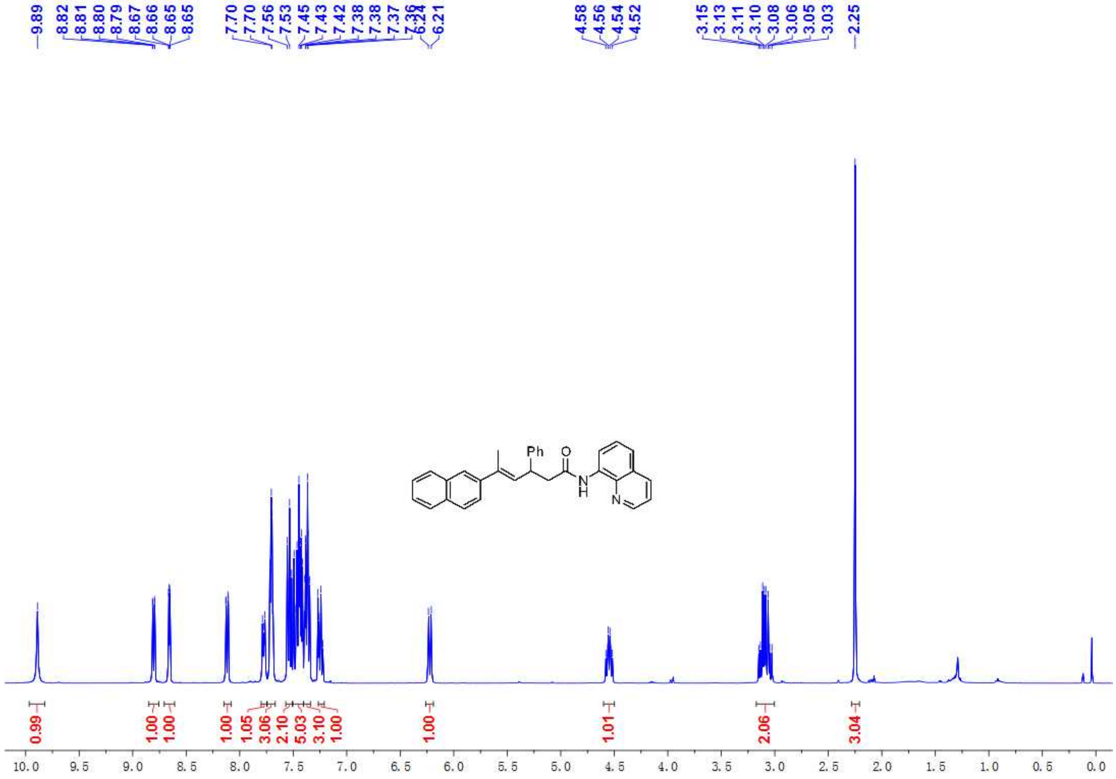

Supplementary Figure 123 ^{13}C NMR (100 MHz, $CDCl_3$) spectrum for 5na

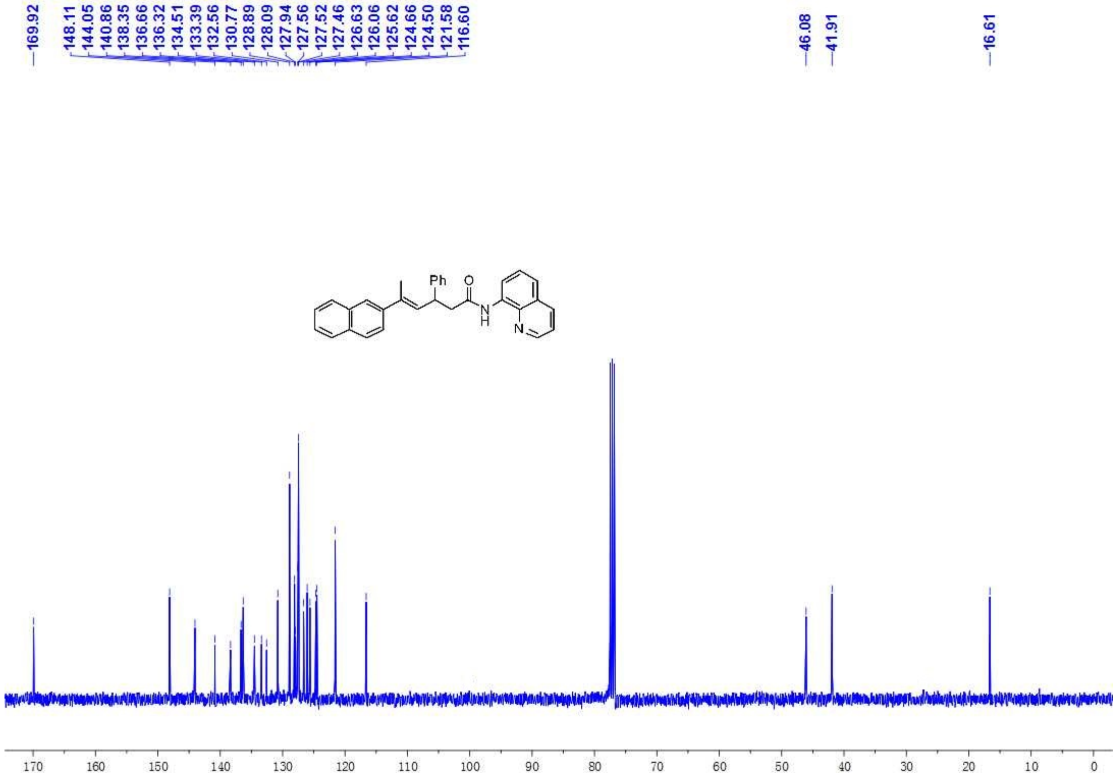

Supplementary Figure 124 ^{1}H **NMR (400 MHz,** $CDCl_3$**) spectrum for 5oa**

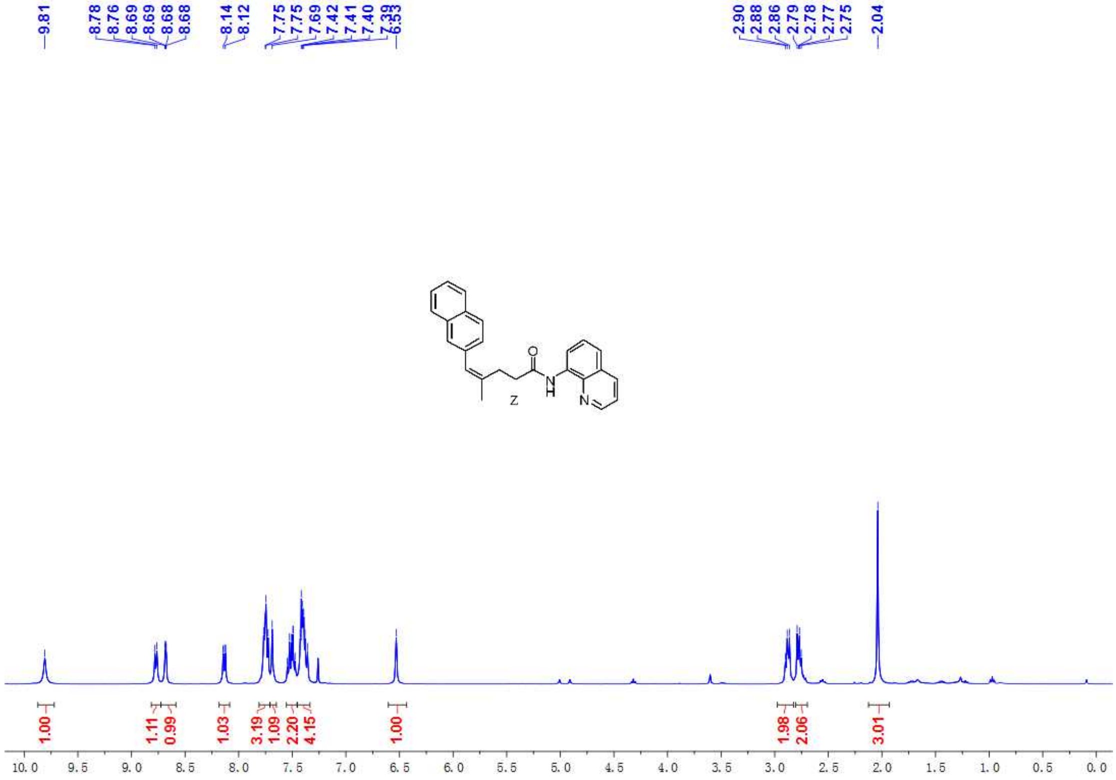

Supplementary Figure 125 ^{13}C **NMR (100 MHz,** $CDCl_3$**) spectrum for 5oa**

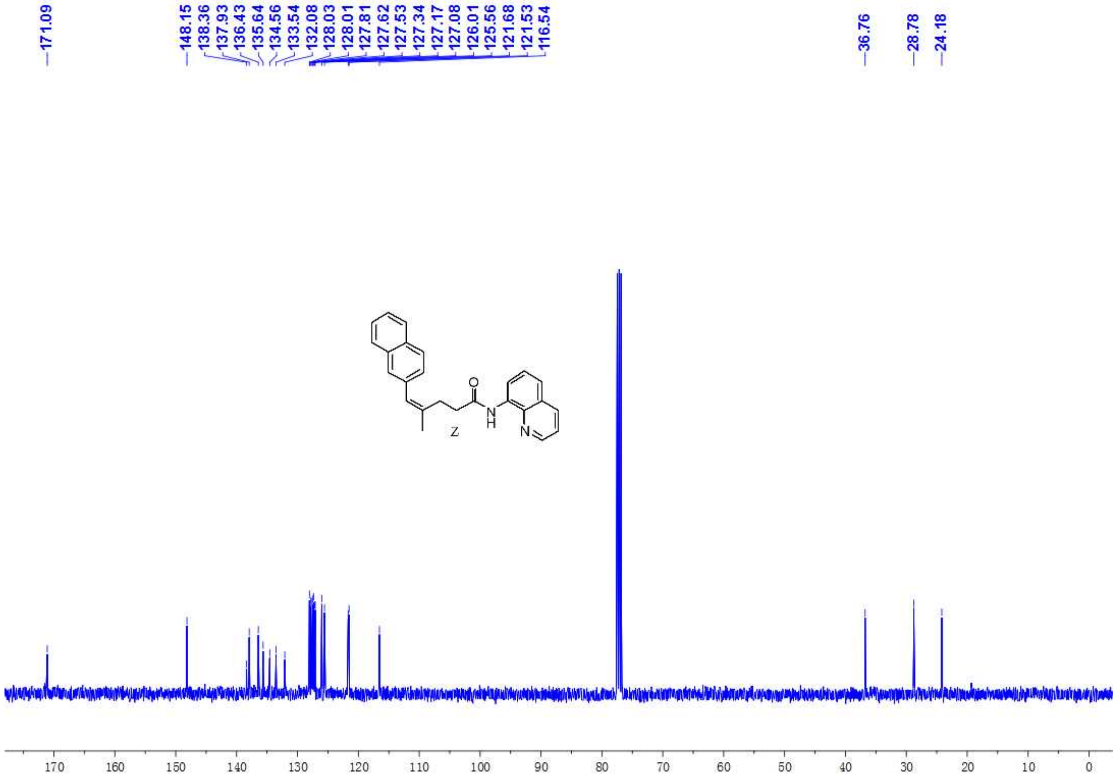

Supplementary Figure 126 ^{1}H NMR (400 MHz, $CDCl_3$) spectrum for 5oa'

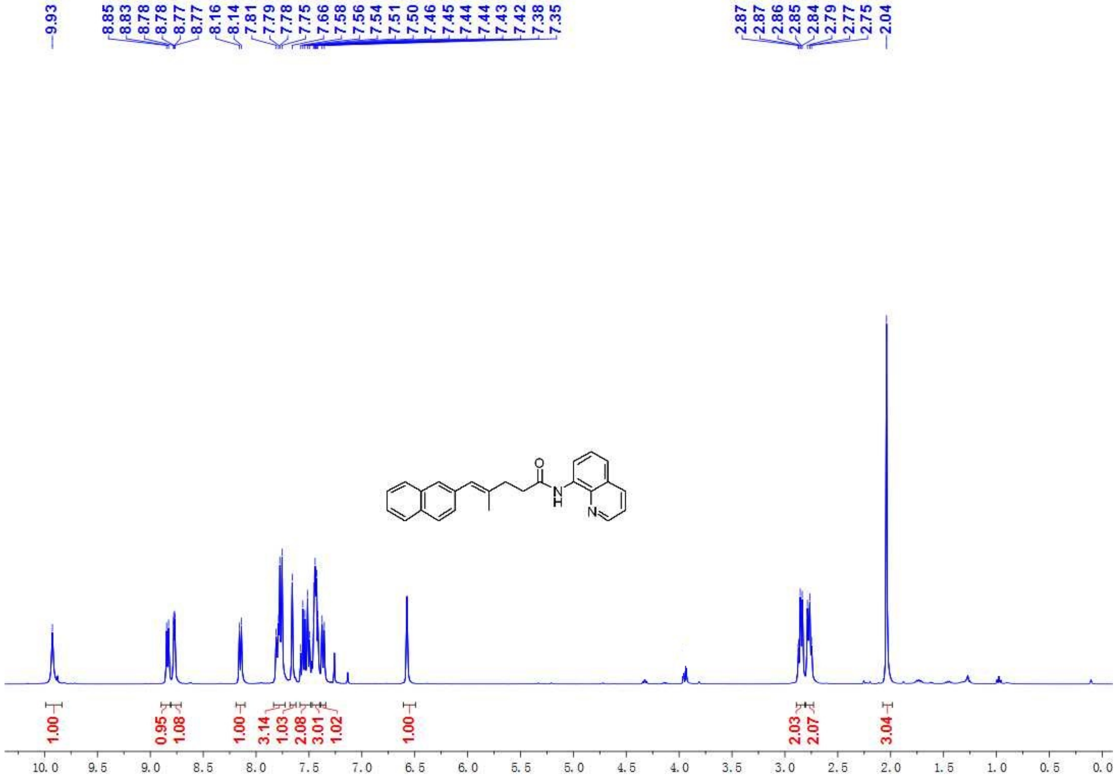

Supplementary Figure 127 ^{13}C NMR (100 MHz, $CDCl_3$) spectrum for 5oa'

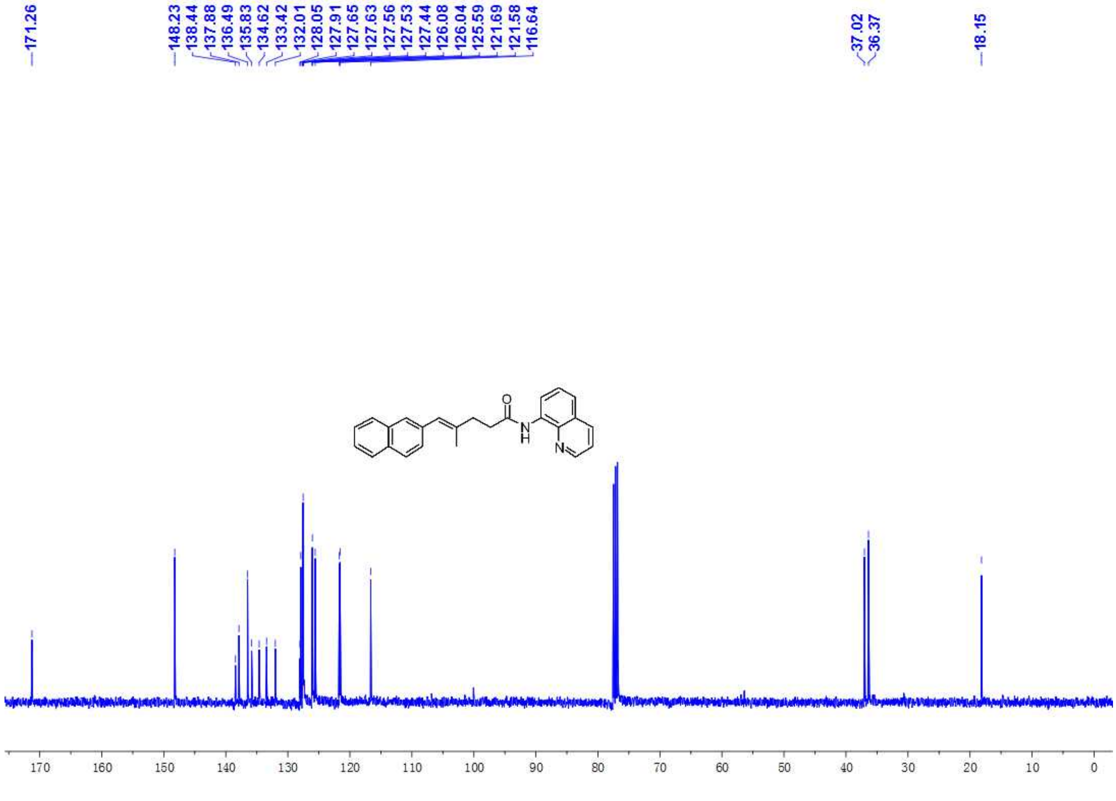

Supplementary Figure 128 ^{1}H NMR (400 MHz, $CDCl_3$) spectrum for 5pa

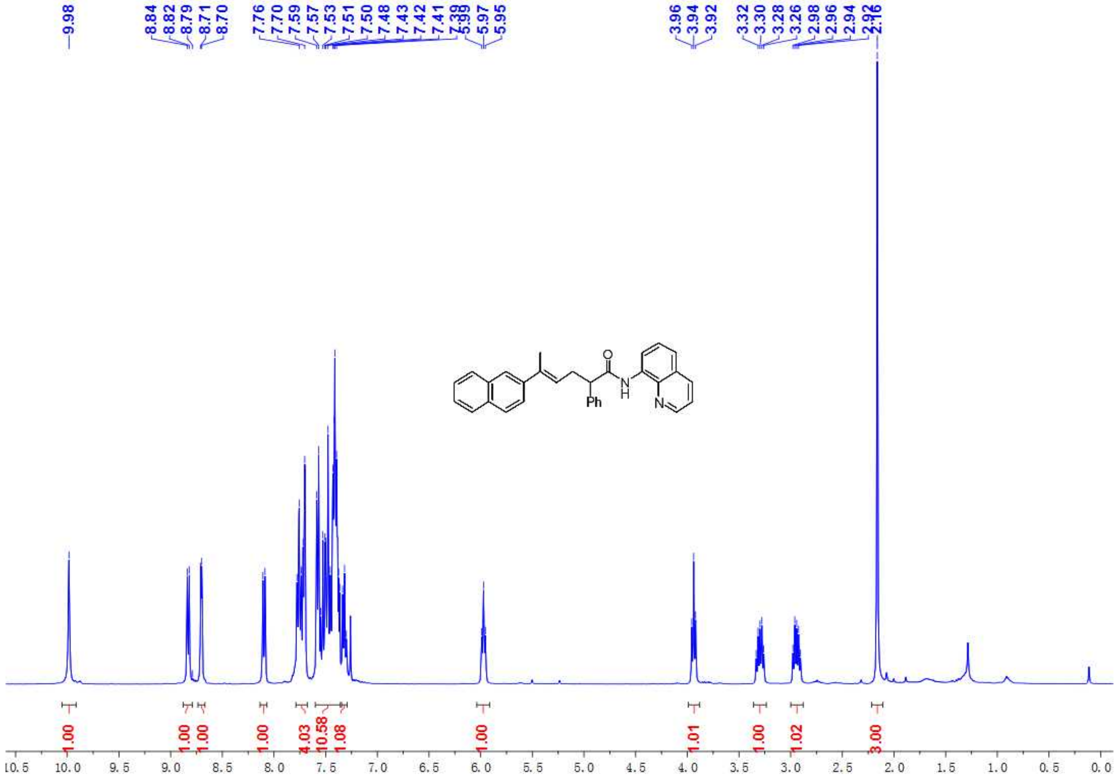

Supplementary Figure 129 ^{13}C NMR (100 MHz, $CDCl_3$) spectrum for 5pa

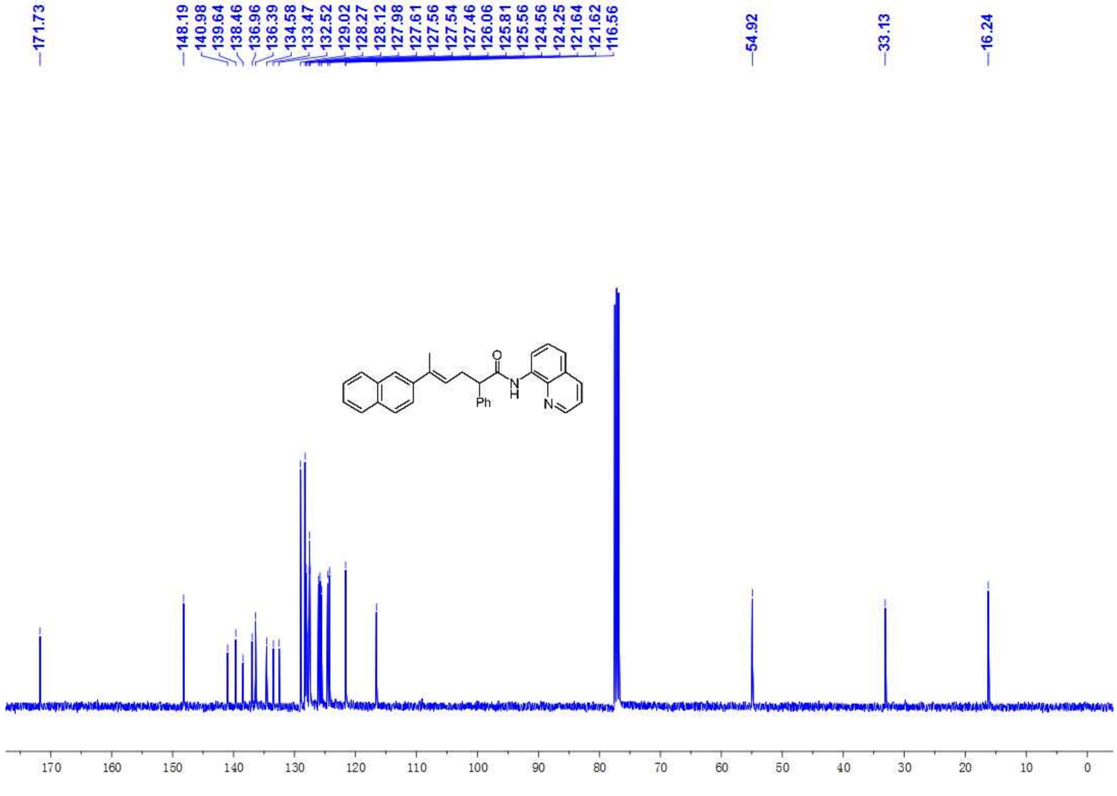

Supplementary Figure 130 ^{1}H NMR (400 MHz, $CDCl_3$) spectrum for 5qa

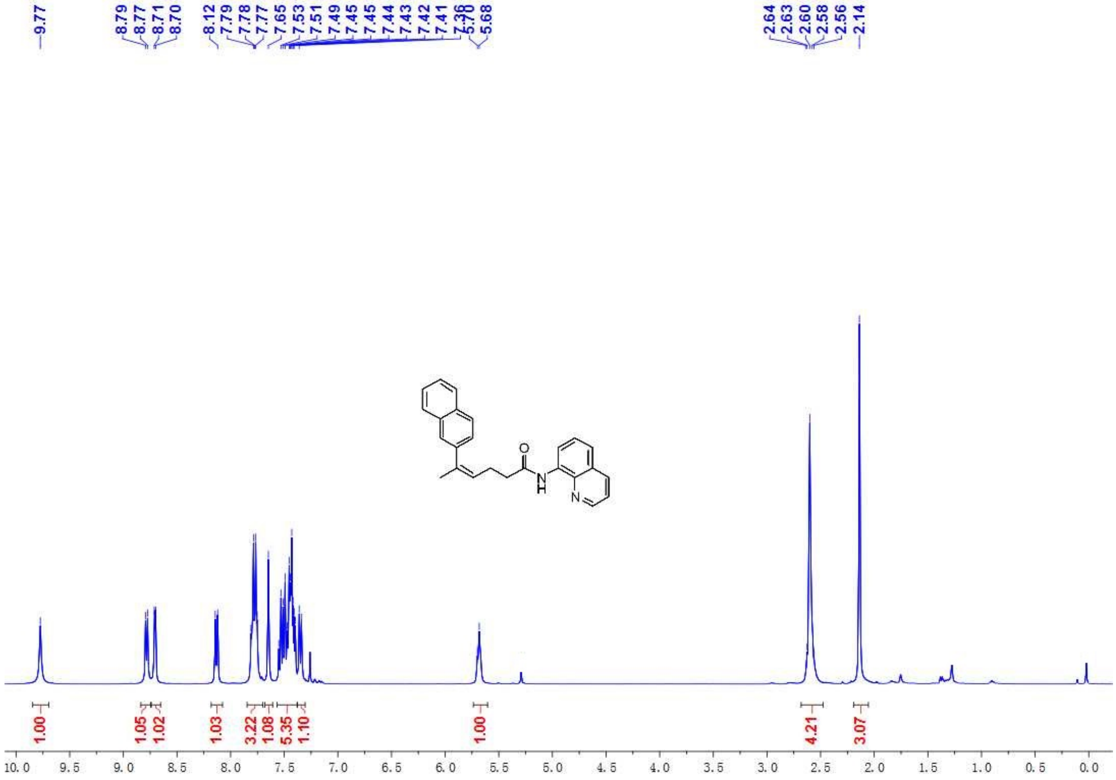

Supplementary Figure 131 ^{13}C NMR (100 MHz, $CDCl_3$) spectrum for 5qa

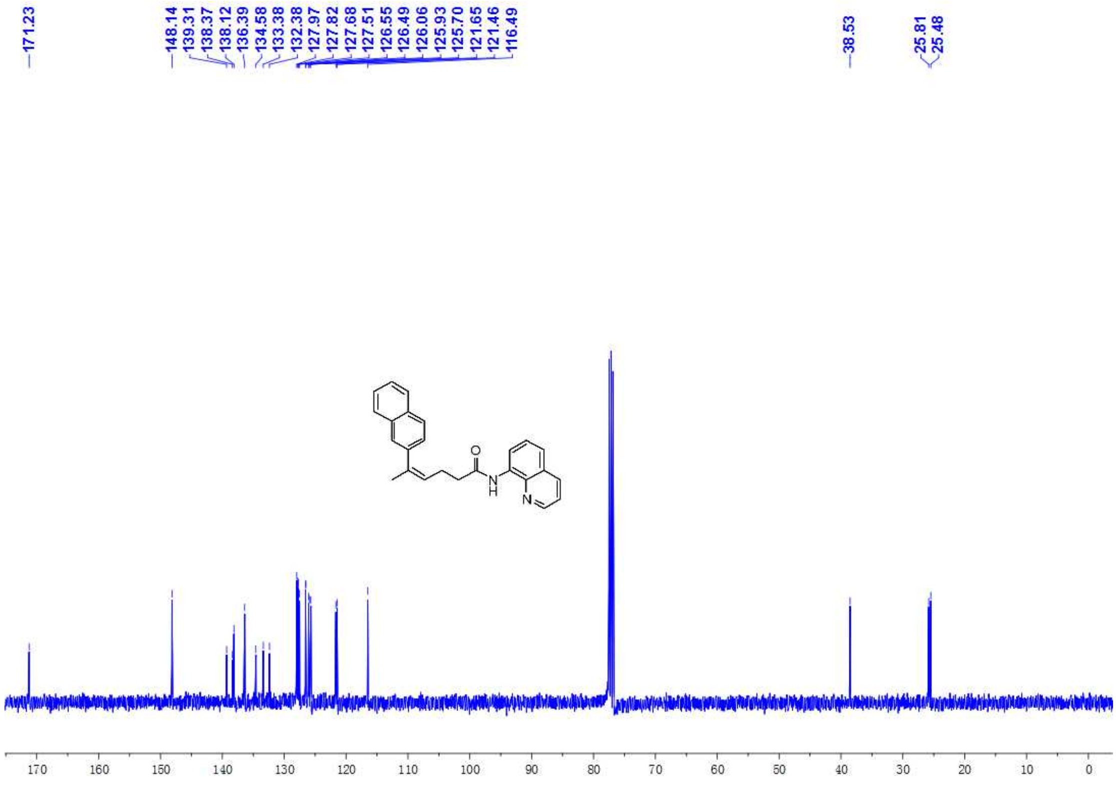

Supplementary Figure 132 ^{1}H NMR (400 MHz, $CDCl_3$) spectrum for 5qb

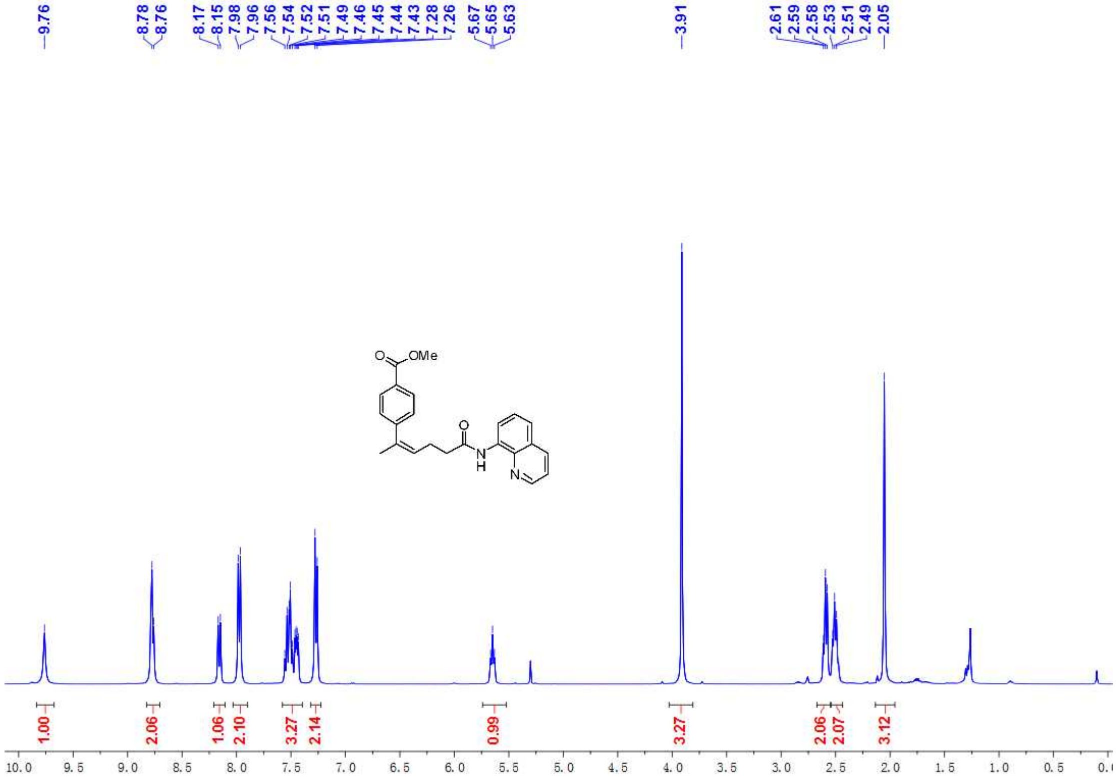

Supplementary Figure 133 ^{13}C NMR (100 MHz, $CDCl_3$) spectrum for 5qb

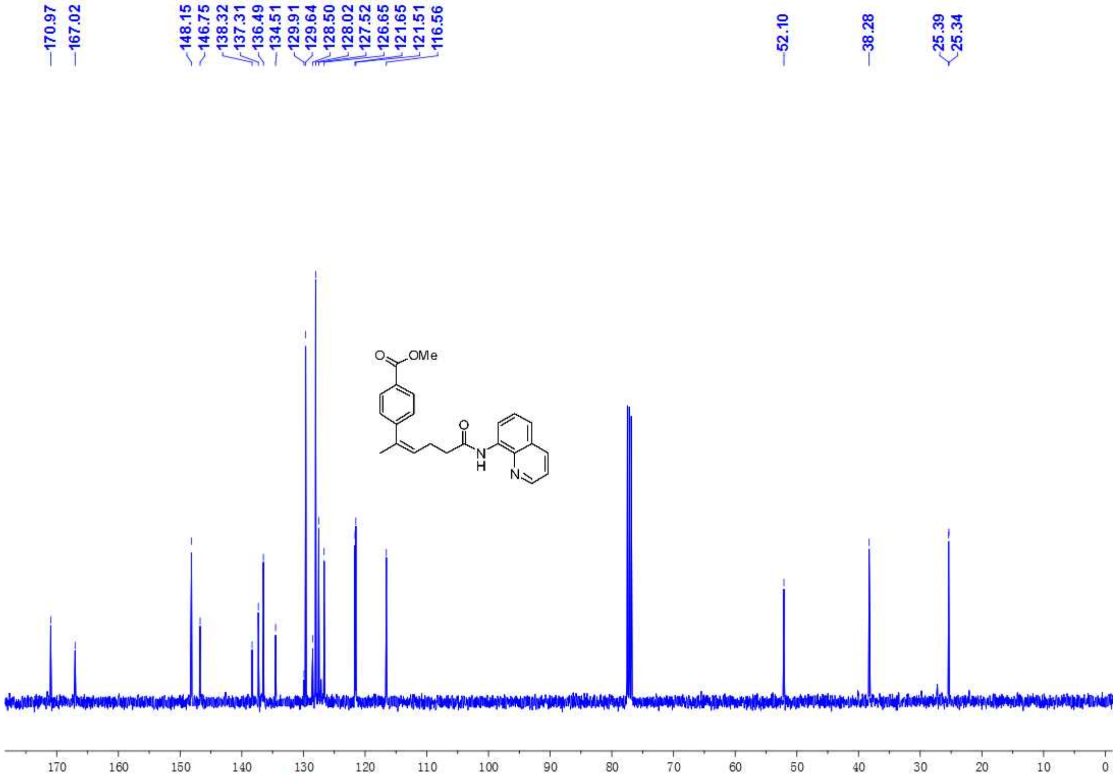

Supplementary Figure 134 ^{1}H NMR (400 MHz, $CDCl_3$) spectrum for 5qc

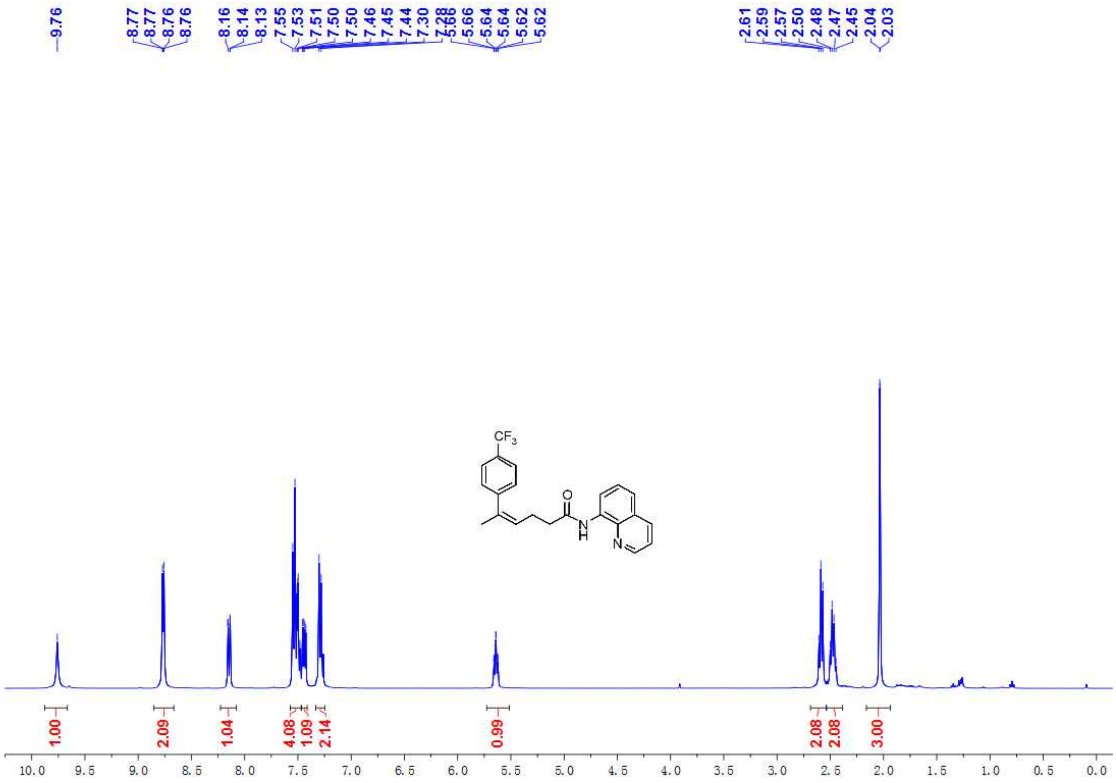

Supplementary Figure 135 ^{13}C NMR (100 MHz, $CDCl_3$) spectrum for 5qc

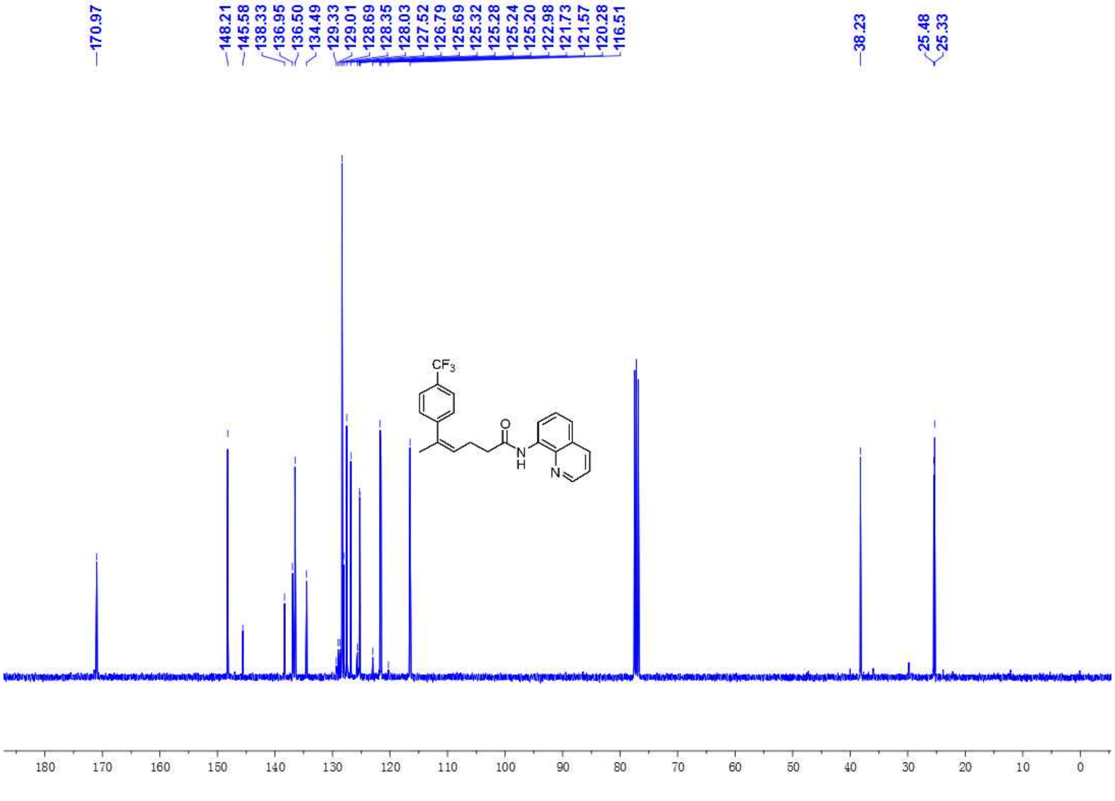

Supplementary Figure 136 ^{19}F NMR (376 MHz, $CDCl_3$) spectrum for 5qc

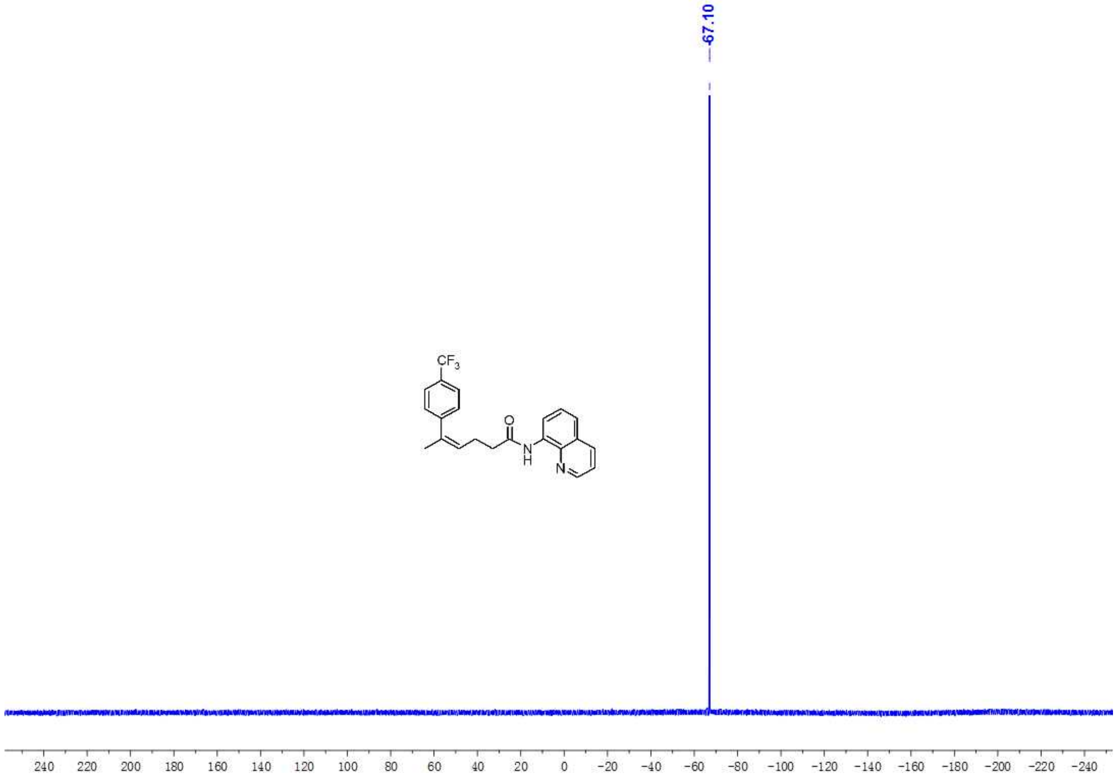

Supplementary Figure 137 ^{1}H NMR (400 MHz, $CDCl_3$) spectrum for 5qd

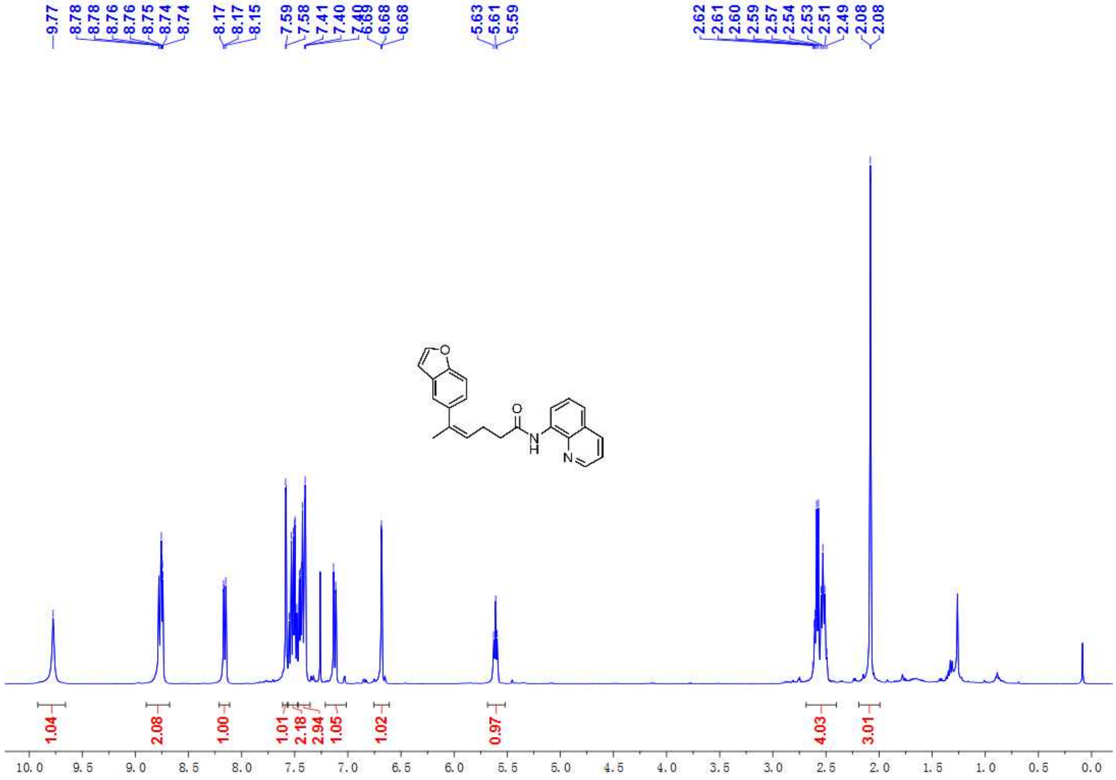

Supplementary Figure 138 ^{13}C NMR (100 MHz, $CDCl_3$) spectrum for 5qd

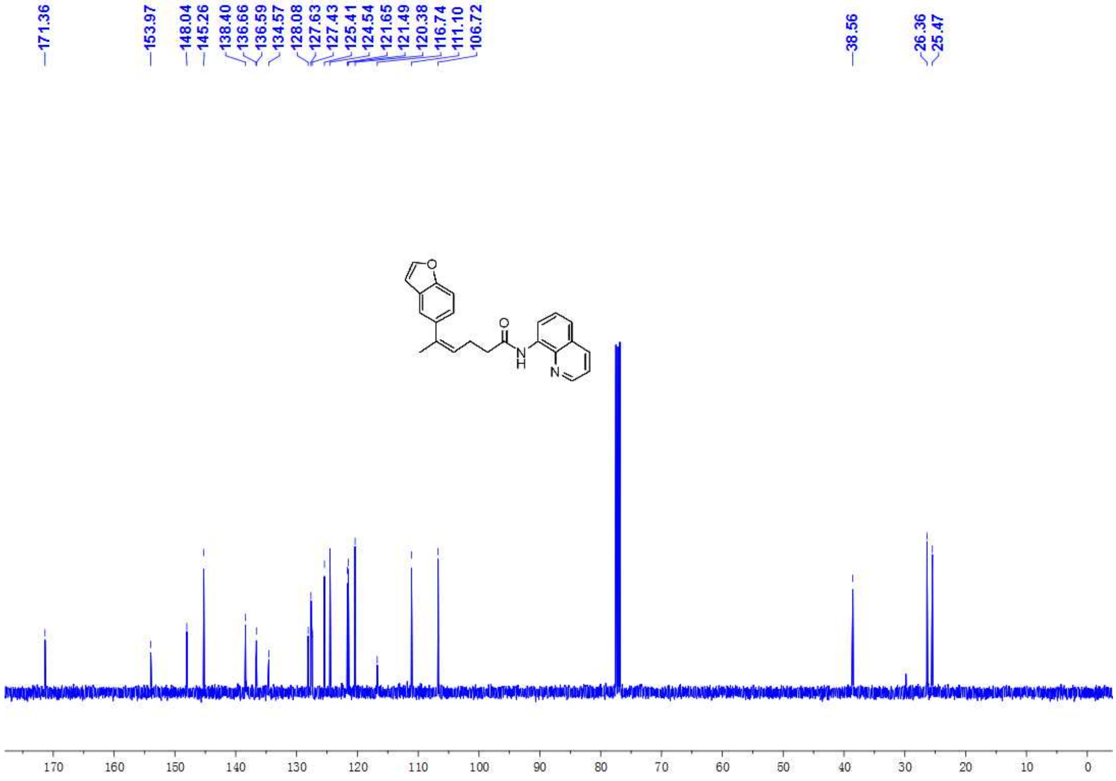

Supplementary Figure 139 ^{1}H NMR (400 MHz, $CDCl_3$) spectrum for 4j

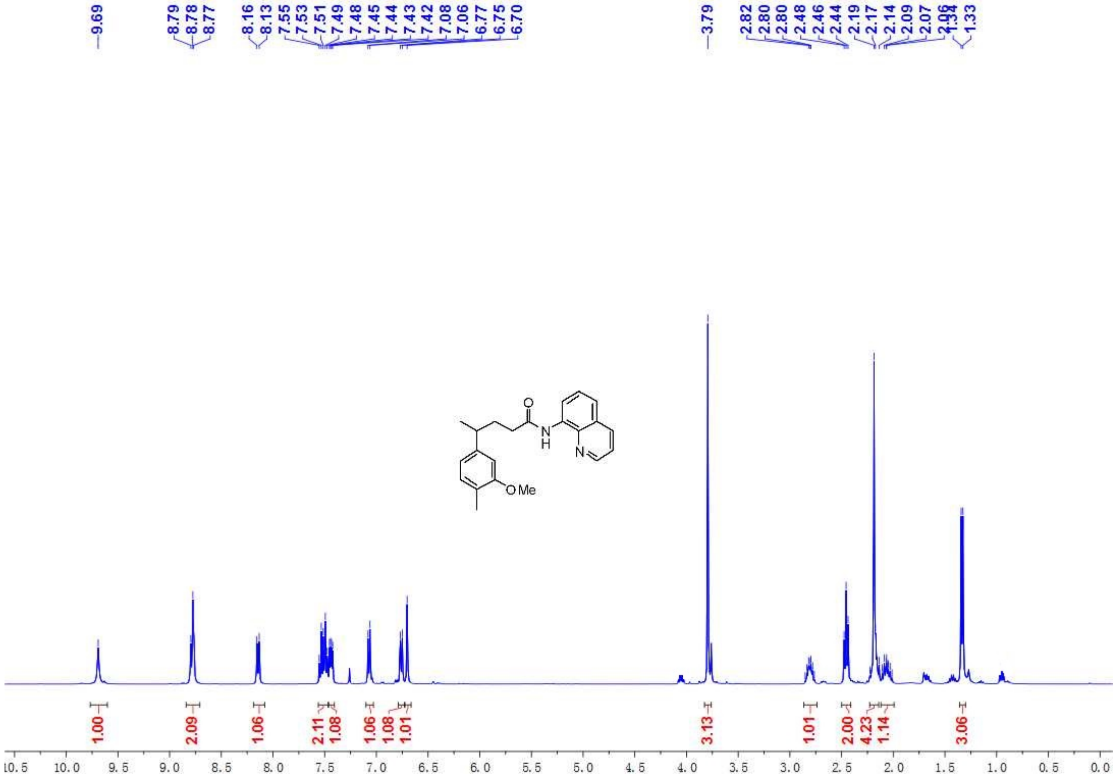

Supplementary Figure 140 ^{13}C NMR (100 MHz, $CDCl_3$) spectrum for 4j

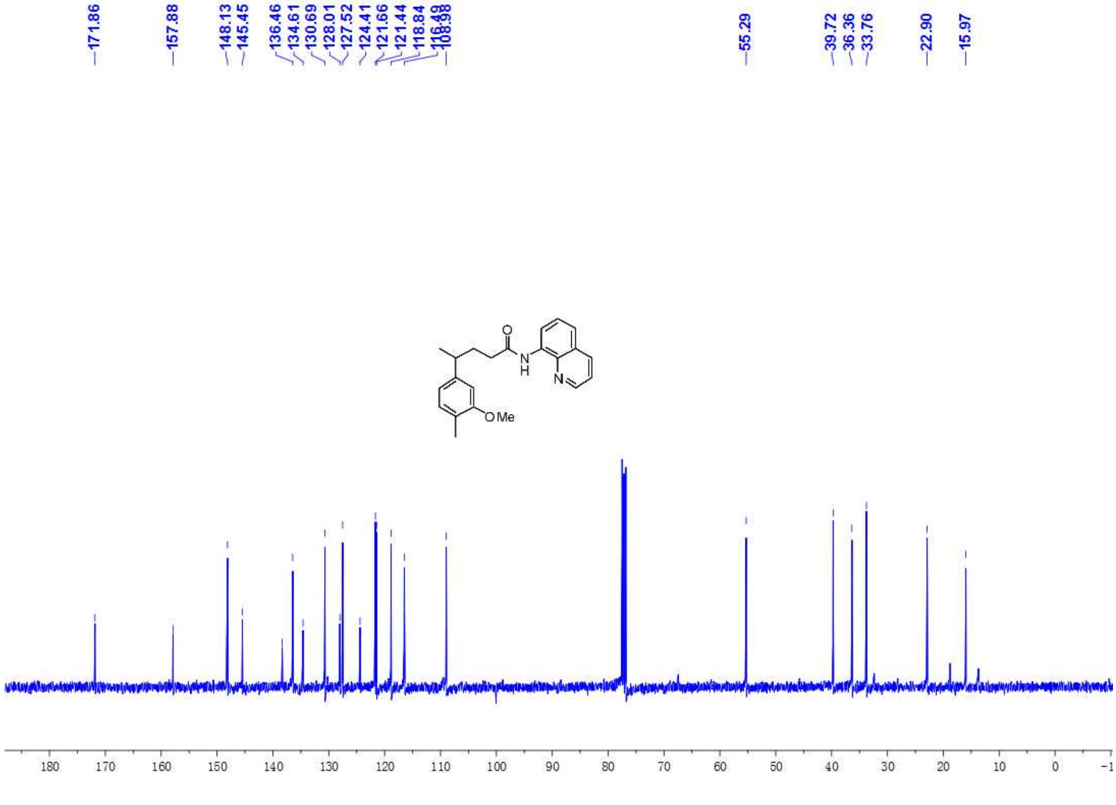

Supplementary Figure 141 ^{1}H NMR (400 MHz, $CDCl_3$) spectrum for 5hz

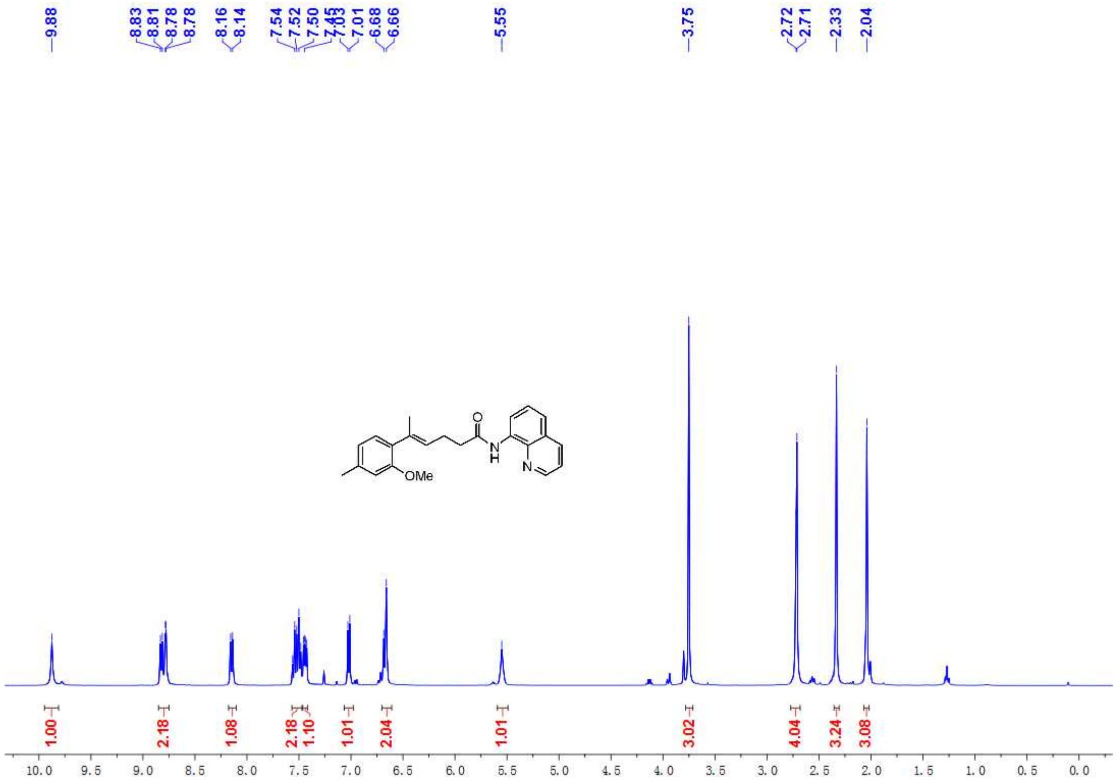

Supplementary Figure 142 ^{13}C NMR (100 MHz, $CDCl_3$) spectrum for 5hz

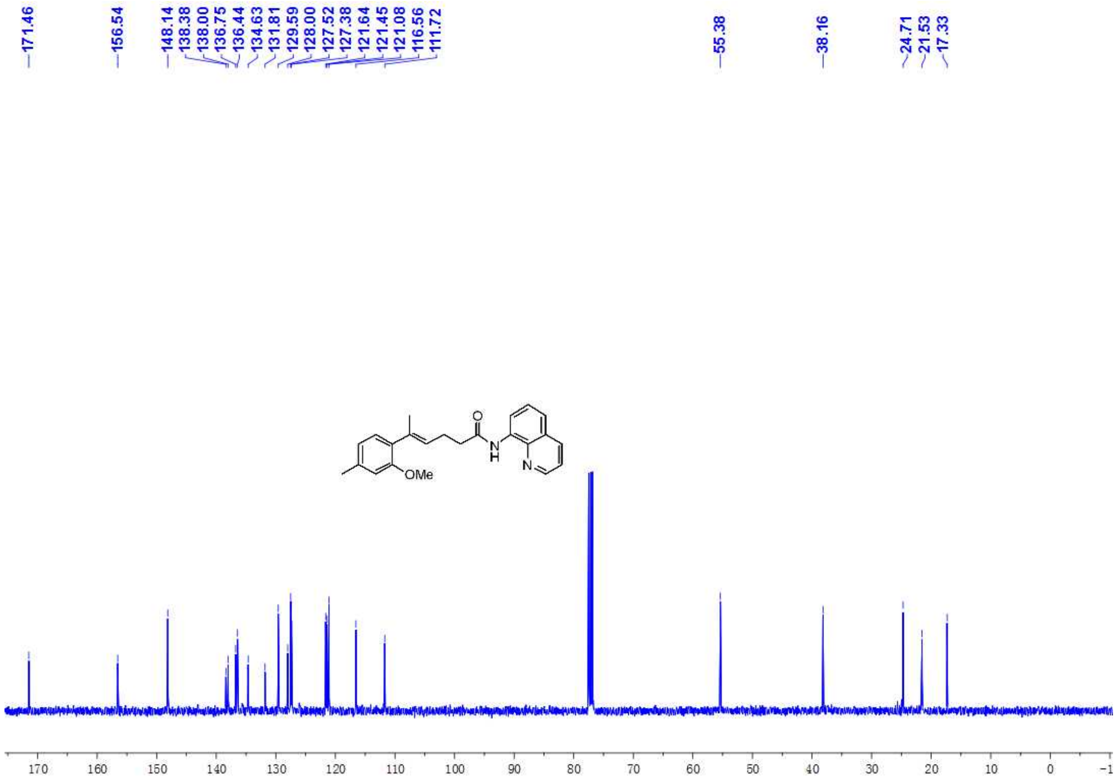

Supplementary Figure 143 ^{1}H NMR (400 MHz, $CDCl_3$) spectrum for 6

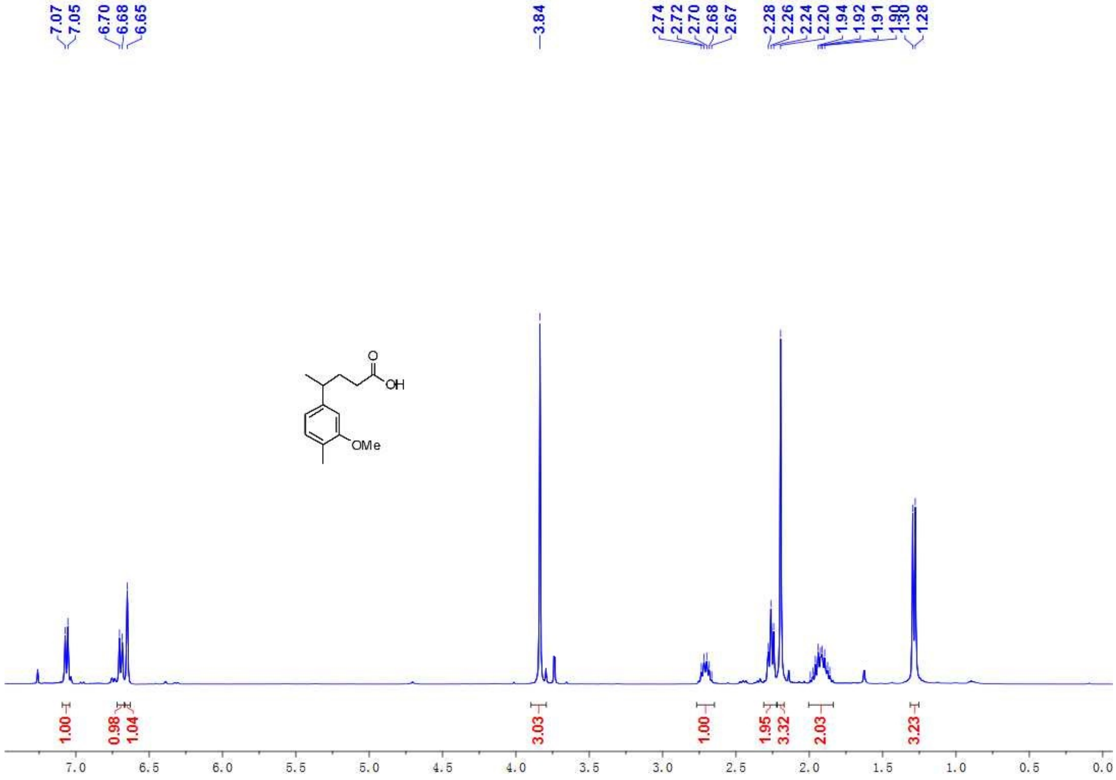

Supplementary Figure 144 ^{13}C NMR (100 MHz, $CDCl_3$) spectrum for 6

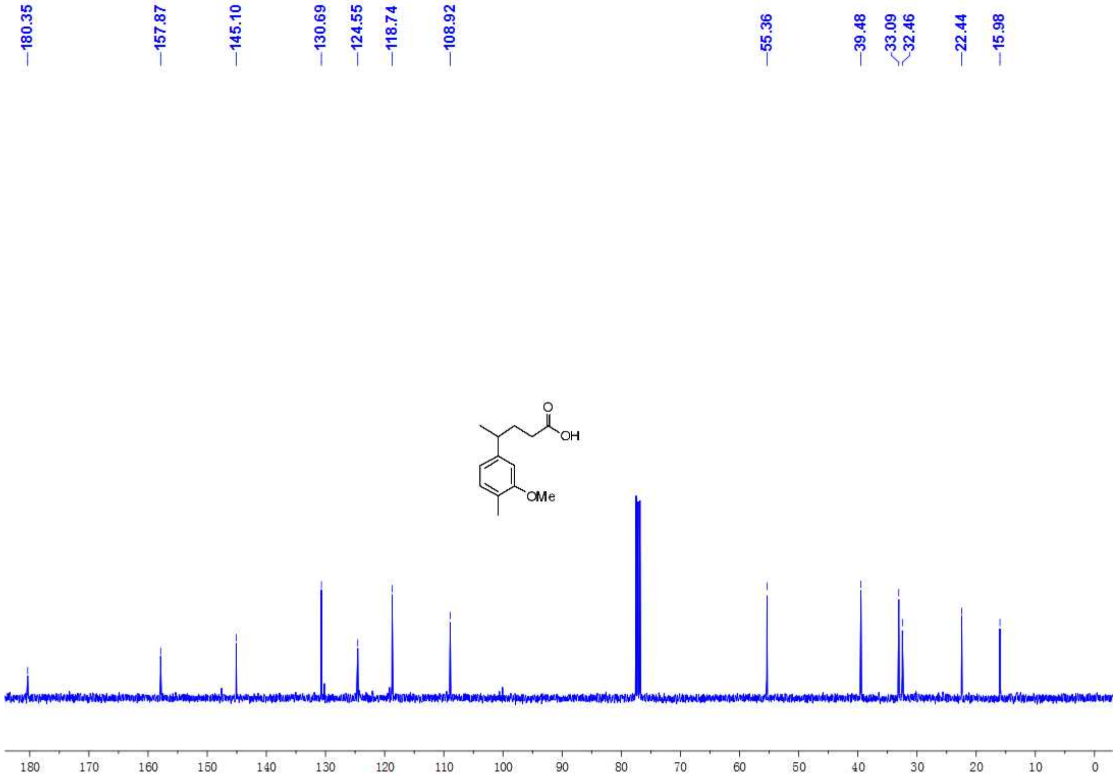

Supplementary **Figure 145** ^{1}**H NMR (400 MHz, $CDCl_3$) spectrum for 7**

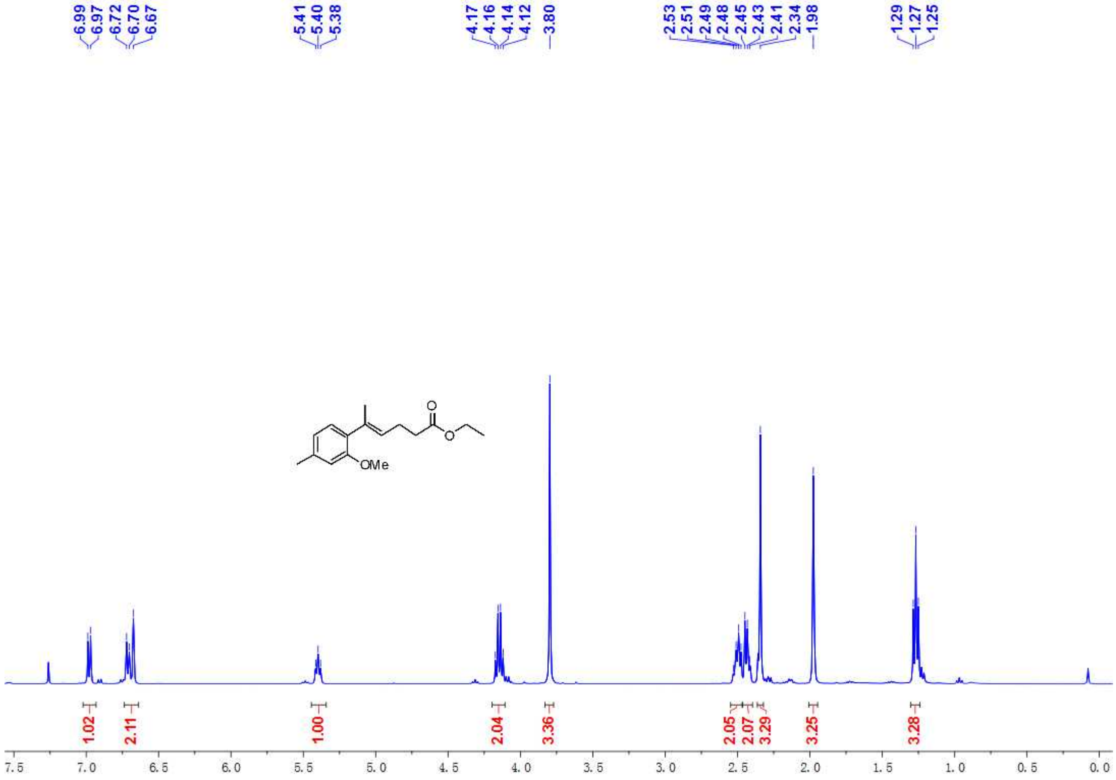

Supplementary **Figure 146** ^{13}**C NMR (100 MHz, $CDCl_3$) spectrum for 7**

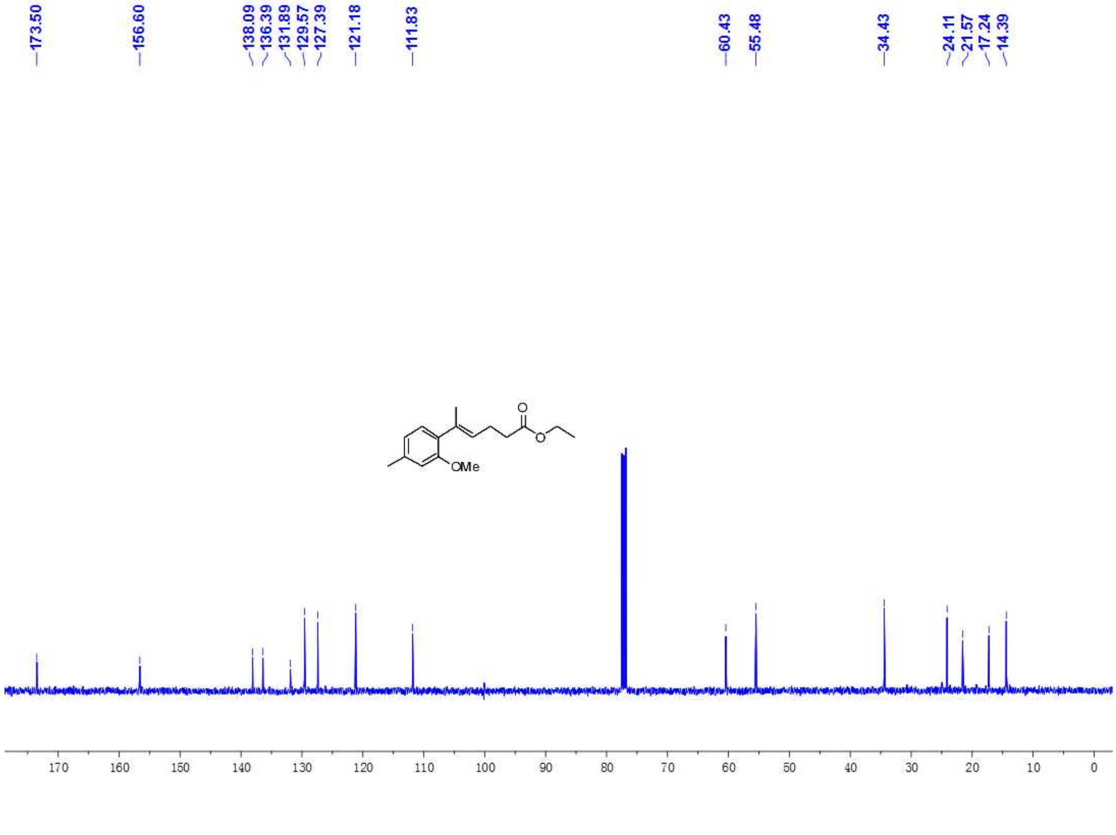

Supplementary References

1. Liu, M., Yang, P., Karunananda, M. K., Wang, Y., Liu, P. & Engle, K. M. C(alkenyl)–H Activation via Six-Membered Palladacycles: Catalytic 1,3-Diene Synthesis. *J. Am. Chem. Soc.* **140**, 5805-5813 (2018).

2. Liu, Z., Ni, H.-Q., Zeng, T. & Engle, K. M. Catalytic Carbo- and Aminoboration of Alkenyl Carbonyl Compounds via Five- and Six-Membered Palladacycles. *J. Am. Chem. Soc.* **140**, 3223-3227 (2018).

3. Derosa, J., Van der Puyl, V. A., Tran, V. T., Liu, M. & Engle, K. M. Directed nickel-catalyzed 1,2-dialkylation of alkenyl carbonyl compounds. *Chem. Sci.* **9**, 5278-5283 (2018).

4. Wang, C., Xiao, G., Guo, T., Ding, Y., Wu, X. & Loh, T.-P. Palladium-Catalyzed Regiocontrollable Reductive Heck Reaction of Unactivated Aliphatic Alkenes. *J. Am. Chem. Soc.* **140**, 9332-9336 (2018).

5. Lv, H., Xiao, L.-J., Zhao, D. & Zhou, Q.-L. Nickel(0)-catalyzed linear-selective hydroarylation of unactivated alkenes and styrenes with aryl boronic acids. *Chem. Sci.* **9**, 6839-6843 (2018).

6. Shan, G., Huang, G. & Rao, Y. Palladium-catalyzed unactivated β-methylene C(sp^3)–H bond alkenylation of aliphatic amides and its application in a sequential C(sp^3)–H/C(sp^2)–H bond alkenylation. *Org. Biomol. Chem.* **13**, 697-701 (2015).

7. Lin, C., Chen, Z., Liu, Z. & Zhang, Y. Nickel-Catalyzed Stereoselective Alkenylation of C(sp^3)–H Bonds with Terminal Alkynes. *Org. Lett.* **19**, 850-853 (2017).

8. Deguchi, T., Xin, H.-L., Morimoto, H. & Ohshima, T. Direct Catalytic Alcoholysis of Unactivated 8-Aminoquinoline Amides. *ACS Catal.* **7**, 3157-3161 (2017).

9. Miiller, U., Keim, W., Kriiger, C. & Betz, P. [{$Ph_2PCH_2C(CF_3)_2O$}$NiH(PCy_3)$]: Support for a Nickel Hydride Mechanism in Ethene Oligomerization. *Angew. Chem. Int. Ed.* **28**, 1011-1013 (1989).

10. Gaussian 16, Revision A.03, Frisch, M. J., Trucks, G. W., Schlegel, H. B., Scuseria, G. E., Robb, M. A., Cheeseman, J. R., Scalmani, G., Barone, V., Petersson,

G. A., Nakatsuji, H., Li, X., Caricato, M., Marenich, A. V., Bloino, J., Janesko, B. G., Gomperts, R., Mennucci, B., Hratchian, H. P., Ortiz, J. V., Izmaylov, A. F., Sonnenberg, J. L., Williams-Young, D., Ding, F., Lipparini, F., Egidi, F., Goings, J., Peng, B., Petrone, A., Henderson, T., Ranasinghe, D., Zakrzewski, V. G., Gao, J., Rega, N., Zheng, G., Liang, W., Hada, M., Ehara, M., Toyota, K., Fukuda, R., Hasegawa, J., Ishida, M., Nakajima, T., Honda, Y., Kitao, O., Nakai, H., Vreven, T., Throssell, K., Montgomery, J. A., Jr., Peralta, J. E., Ogliaro, F., Bearpark, M. J., Heyd, J. J., Brothers, E. N., Kudin, K. N., Staroverov, V. N., Keith, T. A., Kobayashi, R., Normand, J., Raghavachari, K., Rendell, A. P., Burant, J. C., Iyengar, S. S., Tomasi, J., Cossi, M., Millam, J. M., Klene, M., Adamo, C., Cammi, R., Ochterski, J. W., Martin, R. L., Morokuma, K., Farkas, O., Foresman, J. B. & Fox, D. J. Gaussian, Inc., Wallingford CT, 2016.

11. Zhao, Y. & Truhlar, D. G. Density Functionals with Broad Applicability in Chemistry. *Acc. Chem. Res.* **41**, 157-167 (2008).

12. Hay, P. J. & Wadt, W. R. Ab initio effective core potentials for molecular calculations. Potentials for K to Au including the outermost core orbitals. *J. Chem. Phys.* **82**, 299-310 (1985).

13. Marenich, A. V., Cramer, C. J. & Truhlar, D. G. Universal Solvation Model Based on Solute Electron Density and on a Continuum Model of the Solvent Defined by the Bulk Dielectric Constant and Atomic Surface Tensions. *J. Phys. Chem. B.* **113**, 6378 -6396 (2009).

14. Chai, J.-D. & Head-Gordon, M. Long-range corrected hybrid density functionals with damped atom–atom dispersion corrections. *Phy. Chem. Chem. Phy.* **10**, 6615-6620 (2008).

15. (a) Weigend, F. & Ahlrichs, R. Balanced basis sets of split valence, triple zeta valence and quadruple zeta valence quality for H to Rn: Design and assessment of accuracy. *Phy. Chem. Chem. Phy.* **7**, 3297-3305 (2005). (b) Weigend, F. Accurate Coulomb-fitting basis sets for H to Rn. *Phy. Chem. Chem. Phy.* **8**, 1057-1065 (2006).

16. Legault, C. Y. CYLview, 1.0b; Université de Sherbrooke: (Québec) Canada, 2009; http://www.cylview.org.

17. Tong, H.-R., Zheng, S., Li, X., Deng, Z., Wang, H., He, G., Peng, Q. & Chen, G. Pd(0)-Catalyzed Bidentate Auxiliary Directed Enantioselective Benzylic C–H Arylation of 3-Arylpropanamides Using the BINOL Phosphoramidite Ligand. *ACS Catal.* **8**, 11502-11512 (2018).
