# Supplementary material for: Nickel-catalyzed intermolecular oxidative Heck arylation driven by transfer hydrogenation
Source: Nat Commun. 2019 Nov 5;10:5025. doi: 10.1038/s41467-019-12949-1 (PMC6831602; doi:10.1038/s41467-019-12949-1)
Supplement: Supplementary file 2 — Description of Additional Supplementary Files [file 41467_2019_12949_MOESM2_ESM.pdf]

Description of Additional Supplementary Files

File Name: Supplementary Data 1

Description: Calculated Cartesian Coordinates and Single Point Energies.
