# Supplementary material for: Nickel-catalyzed intermolecular oxidative Heck arylation driven by transfer hydrogenation
Source: Nat Commun. 2019 Nov 5;10:5025. doi: 10.1038/s41467-019-12949-1 (PMC6831602; doi:10.1038/s41467-019-12949-1)
Supplement: Supplementary file 3 — Supplementary Data 1 [file 41467_2019_12949_MOESM3_ESM.pdf]

Supplementary Data 1

Calculated Cartesian Coordinates and Single Point Energies

A

Ni	0.32514	0.61159	-0.0517
P	2.48957	0.26058	-0.10064
C	3.37391	0.61541	1.46209
H	4.45139	0.42443	1.35993
H	3.21854	1.6661	1.73824
H	2.97746	-0.01239	2.27063
C	3.34348	1.35777	-1.29287
H	3.14801	2.40588	-1.0337
H	4.42789	1.17949	-1.28682
H	2.95831	1.17743	-2.3045
C	3.14495	-1.38617	-0.56305
H	2.88458	-2.1253	0.20498
H	2.70619	-1.70709	-1.51598
H	4.23892	-1.35259	-0.66301
H	0.83969	1.96659	0.36048
O	-0.27787	-1.0843	-0.65698
C	-0.62009	-2.09428	0.23804
C	-0.05236	-1.845	1.64035
H	-0.33179	-2.64268	2.34707
H	1.04671	-1.79628	1.61478
H	-0.42566	-0.88952	2.0456
C	-0.05658	-3.42444	-0.27836
H	-0.39557	-4.2836	0.32204
H	-0.36087	-3.59668	-1.32007
H	1.04273	-3.40984	-0.25559
C	-2.15514	-2.21337	0.37855
H	-2.39666	-2.96065	1.15419
H	-2.52411	-1.24646	0.76348
C	-2.88265	-2.56687	-0.90725
H	-2.6981	-3.6085	-1.20581
H	-3.9708	-2.4516	-0.80417
H	-2.55299	-1.92302	-1.73462
P	-1.6023	1.66913	0.02405
C	-2.41909	1.70773	1.6635
H	-3.3143	2.34529	1.63891
H	-2.71508	0.69593	1.96825
H	-1.71913	2.10284	2.41115
C	-2.93754	1.10289	-1.09241
H	-3.28968	0.10809	-0.79747

H	-3.78506	1.8021	-1.06449
H	-2.55846	1.0431	-2.12067
C	-1.51254	3.45195	-0.38613
H	-0.88528	3.96756	0.35207
H	-1.05294	3.58286	-1.37427
H	-2.51183	3.90904	-0.39324

Zero-point correction= 0.388676 (Hartree/Particle)
Thermal correction to Energy= 0.420845
Thermal correction to Enthalpy= 0.421932
Thermal correction to Gibbs Free Energy= 0.321829
SCF Done: E(RwB97XD) = -2703.69498585

A-1

C	-1.06953	-2.56592	1.21288
C	-0.88052	-1.05222	1.29776
O	-1.79057	-0.41212	1.87001
N	0.20698	-0.52322	0.70308
C	0.54036	0.81998	0.78872
C	1.80468	1.15142	0.18431
C	-0.16448	1.86353	1.38905
C	2.29082	2.48441	0.17872
C	0.33196	3.18286	1.38308
H	-1.10629	1.64448	1.87939
C	3.68829	0.39226	-0.93921
C	3.53725	2.72761	-0.44481
C	1.52735	3.50685	0.78891
H	-0.25971	3.96101	1.86576
C	4.23304	1.68692	-1.00572
H	3.9285	3.74497	-0.46658
H	1.90313	4.52972	0.78109
H	5.1951	1.83998	-1.48953
N	2.52103	0.12334	-0.37285
C	0.13535	-3.48455	1.00638
H	4.22741	-0.45725	-1.35756
H	0.88144	-3.30992	1.79959
H	-1.78084	-2.73606	0.38203
H	-1.62047	-2.84594	2.12252
H	-0.21927	-4.52455	1.14886
Ni	1.53772	-1.56701	-0.31287
Cs	-3.2291	0.62921	-0.75018
C	0.75767	-3.2729	-0.3499
H	0.03706	-3.40497	-1.17399
C	2.15081	-3.37948	-0.67952
H	2.42911	-3.70519	-1.68922

H	2.85923	-3.73505	0.08207

Zero-point correction= 0.241161 (Hartree/Particle)
Thermal correction to Energy= 0.263280
Thermal correction to Enthalpy= 0.264367
Thermal correction to Gibbs Free Energy= 0.182661
SCF Done: E(RwB97XD) = -2254.74018855

A-2

C	-0.89705	-2.60583	-1.84737
C	-0.00944	-1.41268	-1.51984
O	1.17045	-1.6549	-1.15705
N	-0.59527	-0.21501	-1.60807
C	0.09792	0.94531	-1.36678
C	-0.54952	1.9396	-0.55248
C	1.34817	1.26432	-1.89873
C	0.08873	3.18569	-0.29283
C	1.96421	2.50078	-1.64467
H	1.84391	0.53417	-2.53677
C	-2.36981	2.53392	0.76316
C	-0.58169	4.1112	0.54418
C	1.35972	3.45237	-0.85203
H	2.93922	2.70645	-2.08869
C	-1.80565	3.78461	1.0708
H	-0.11189	5.07208	0.75569
H	1.83852	4.41035	-0.64852
H	-2.35012	4.47346	1.71356
N	-1.77388	1.63194	-0.00845
C	-2.36145	-2.32515	-2.16221
H	-3.34584	2.2552	1.16197
H	-2.40749	-1.5994	-2.98913
H	-0.81704	-3.30383	-0.9969
H	-0.42236	-3.13297	-2.69237
H	-2.80939	-3.2575	-2.55165
Ni	-2.32359	-0.26359	-0.18208
P	-1.33261	-0.98348	1.67533
C	0.01721	0.1157	2.30532
H	0.52148	-0.2998	3.19115
H	0.75809	0.27937	1.51002
H	-0.40043	1.09768	2.57027
C	-0.47082	-2.61411	1.79467
H	0.33491	-2.65073	1.04771
H	-0.04382	-2.78106	2.79561
H	-1.17453	-3.42954	1.57661
C	-2.37249	-1.07603	3.19762

H	-3.16402	-1.82505	3.05615
H	-1.79025	-1.34029	4.09369
H	-2.8604	-0.10512	3.36406
Cs	3.64872	-0.66372	0.34919
C	-3.1965	-1.79959	-1.00868
H	-3.50998	-2.56263	-0.28198
C	-4.00753	-0.65025	-1.18898
H	-4.91269	-0.51116	-0.58851
H	-4.04911	-0.17063	-2.17401

Zero-point correction= 0.354012 (Hartree/Particle)
Thermal correction to Energy= 0.386371
Thermal correction to Enthalpy= 0.387458
Thermal correction to Gibbs Free Energy= 0.285304
SCF Done: E(RwB97XD) = -2715.87480708

A-3

C	-0.67381	-3.0139	1.08232
C	-0.74143	-1.51422	1.34535
O	-1.73153	-1.10899	2.00598
N	0.19783	-0.72623	0.81494
C	0.13929	0.63704	1.12597
C	-0.29233	1.59747	0.1508
C	0.56232	1.10885	2.35966
C	-0.24239	2.98971	0.46715
C	0.5985	2.48206	2.66496
H	0.89034	0.3795	3.10155
C	-1.07729	2.04617	-1.96754
C	-0.63549	3.90489	-0.5372
C	0.20698	3.41842	1.73741
H	0.94472	2.79859	3.64862
C	-1.04831	3.44169	-1.75927
H	-0.59657	4.97344	-0.32164
H	0.23602	4.48565	1.95777
H	-1.35024	4.11901	-2.55543
N	-0.72322	1.15579	-1.06448
C	0.63505	-3.63666	0.61165
H	-1.41424	1.65616	-2.93186
H	1.41774	-3.49263	1.37585
H	-1.45408	-3.22281	0.32609
H	-1.03084	-3.5008	2.00168
H	0.4784	-4.73113	0.54414
Ni	1.716	-1.25915	-0.33935
Cs	-3.59604	-0.45141	-0.41202
C	1.08285	-3.04702	-0.69835

H	0.30348	-3.05316	-1.47567
C	2.43257	-2.90997	-1.11621
H	2.67417	-2.89396	-2.18509
H	3.24289	-3.31471	-0.4949
P	3.27873	0.2587	-0.41757
C	2.97329	2.02605	-0.87066
H	3.91714	2.58277	-0.97021
H	2.42554	2.0894	-1.82107
H	2.36439	2.51023	-0.09515
C	4.67293	-0.10815	-1.56972
H	4.29618	-0.15962	-2.60056
H	5.46661	0.65236	-1.5207
H	5.10446	-1.0878	-1.32295
C	4.22736	0.51229	1.14562
H	4.6448	-0.44512	1.48535
H	5.04956	1.23228	1.01765
H	3.5523	0.88208	1.93005

Zero-point correction= 0.354539 (Hartree/Particle)
Thermal correction to Energy= 0.387141
Thermal correction to Enthalpy= 0.388228
Thermal correction to Gibbs Free Energy= 0.283372
SCF Done: E(RwB97XD) = -2715.88452204

Add

C	2.78769	-0.99664	0.
C	1.42695	-1.27436	-0.00001
C	0.48236	-0.23841	-0.00002
C	0.93832	1.08938	0.
C	2.29664	1.36562	0.00003
C	3.22522	0.32442	0.00004
H	3.50848	-1.81248	-0.00003
H	1.07982	-2.30781	-0.00004
H	0.22209	1.90989	-0.00005
H	2.63781	2.39943	0.00007
H	4.29117	0.54629	0.00009
C	-0.92981	-0.58278	-0.00001
H	-1.14603	-1.65647	0.00013
C	-1.98467	0.25621	-0.00017
H	-1.88637	1.34267	-0.00033
C	-3.32913	-0.28778	0.00002
O	-4.3512	0.38083	0.0001
H	-3.37878	-1.40206	0.00007

Zero-point correction= 0.143327 (Hartree/Particle)
Thermal correction to Energy= 0.154353

Thermal correction to Enthalpy= 0.155440
Thermal correction to Gibbs Free Energy= 0.102389
SCF Done: E(RwB97XD) = -422.974051558

Add-H^{-}

C	-2.74903	-0.65998	-0.12357
C	-1.50909	-1.25361	0.11261
C	-0.35644	-0.48112	0.26018
C	-0.47752	0.9113	0.16404
C	-1.71125	1.50866	-0.05916
C	-2.85581	0.72374	-0.20752
H	-3.635	-1.28415	-0.24111
H	-1.43309	-2.3406	0.18212
H	0.42165	1.5198	0.27732
H	-1.78451	2.59462	-0.11904
H	-3.82329	1.19171	-0.38647
C	1.00083	-1.10058	0.49748
H	1.406	-0.68983	1.43788
C	2.00638	-0.82565	-0.58666
H	1.95033	-1.40133	-1.51381
C	2.91981	0.19485	-0.49001
O	3.10419	1.02602	0.46005
H	3.59403	0.27875	-1.38759
H	0.8631	-2.18277	0.6661

Zero-point correction= 0.152986 (Hartree/Particle)
Thermal correction to Energy= 0.164186
Thermal correction to Enthalpy= 0.165273
Thermal correction to Gibbs Free Energy= 0.111996
SCF Done: E(RwB97XD) = -423.692115676

Add-H2

C	-2.5117	1.20344	0.08462
C	-1.14852	1.20059	-0.19477
C	-0.45003	-0.00025	-0.34033
C	-1.14881	-1.20086	-0.19425
C	-2.51198	-1.20326	0.08512
C	-3.19821	0.00021	0.22503
H	-3.04114	2.14972	0.19015
H	-0.61286	2.14522	-0.30576
H	-0.61335	-2.14565	-0.30483
H	-3.04169	-2.14935	0.19104
H	-4.26571	0.00036	0.44088
C	1.03196	-0.00041	-0.5878
H	1.32124	-0.88225	-1.17735

C	1.81134	0.00056	0.71991
H	1.55067	-0.87133	1.34425
C	3.29382	0.00025	0.55135
O	3.86891	-0.00039	-0.51742
H	3.87231	0.00065	1.50338
H	1.32111	0.88062	-1.17864
H	1.55092	0.87349	1.34288

Zero-point correction= 0.165892 (Hartree/Particle)
Thermal correction to Energy= 0.177655
Thermal correction to Enthalpy= 0.178742
Thermal correction to Gibbs Free Energy= 0.123191
SCF Done: E(RwB97XD) = -424.202260221

B

Ni	0.03214	-0.85542	0.02081
P	-2.13721	-0.97058	0.0439
C	-2.92157	-2.45346	0.77566
H	-4.01521	-2.41831	0.67275
H	-2.53975	-3.35085	0.27317
H	-2.66618	-2.52475	1.84053
C	-2.74832	-1.01103	-1.6808
H	-2.31056	-1.86838	-2.20779
H	-3.84406	-1.09332	-1.70768
H	-2.44724	-0.09349	-2.20269
C	-3.12975	0.38408	0.77454
H	-2.89488	0.49691	1.84068
H	-2.90148	1.33177	0.27039
H	-4.20271	0.17108	0.66871
H	0.08065	-2.40976	0.0523
P	2.20292	-0.84823	-0.04281
C	2.86726	-0.86275	1.66263
H	3.96601	-0.89393	1.6558
H	2.54081	0.03962	2.19565
H	2.48477	-1.74003	2.19952
C	3.10121	0.56027	-0.79254
H	2.82404	1.49467	-0.28817
H	4.1865	0.41204	-0.70407
H	2.84267	0.65441	-1.85505
C	3.04255	-2.28445	-0.80582
H	2.74392	-3.19961	-0.27939
H	2.73811	-2.38156	-1.85561
H	4.13545	-2.17883	-0.75889
C	-0.03354	1.0643	-0.00072
C	0.04232	1.80599	1.18998

C	-0.16742	1.79757	-1.1913
C	-0.01224	3.2004	1.19656
H	0.13623	1.28344	2.14747
C	-0.22962	3.1919	-1.19646
H	-0.22109	1.26957	-2.14888
C	-0.15074	3.90216	0.0002
H	0.05084	3.74366	2.14093
H	-0.33731	3.72876	-2.14046
H	-0.19604	4.99117	0.00039

Zero-point correction= 0.324337 (Hartree/Particle)
Thermal correction to Energy= 0.352650
Thermal correction to Enthalpy= 0.353737
Thermal correction to Gibbs Free Energy= 0.261760
SCF Done: E(RwB97XD) = -2662.91574263

C

C	0	0.44969	-3.2511	0.75718
C	0	-0.52177	-2.0878	0.63187
O	0	-1.74014	-2.34882	0.44282
N	0	0.00241	-0.8753	0.74979
C	0	-0.88447	0.19566	0.83023
C	0	-1.29853	0.93896	-0.32701
C	0	-1.36252	0.61474	2.06487
C	0	-2.09891	2.11278	-0.1662
C	0	-2.18126	1.7493	2.21192
H	0	-1.06477	0.04324	2.94503
C	0	-1.31497	1.16799	-2.61944
C	0	-2.46361	2.82279	-1.33627
C	0	-2.53403	2.50858	1.11971
H	0	-2.52153	2.03365	3.20757
C	0	-2.0708	2.36118	-2.56527
H	0	-3.06132	3.7301	-1.23739
H	0	-3.15004	3.40248	1.22131
H	0	-2.33485	2.883	-3.48285
N	0	-0.94393	0.48369	-1.56039
C	0	1.51167	-3.09156	1.84626
H	0	-1.00785	0.76968	-3.59045
H	0	1.06775	-2.65066	2.75352
H	0	0.94817	-3.41094	-0.21434
H	0	-0.15656	-4.14727	0.94502
H	0	1.86714	-4.09782	2.12969
Ni	0	2.142	-0.28856	1.01105
P	0	2.34664	1.85993	0.68545
C	0	1.30155	3.04608	1.62124

H	0	1.64485	4.07752	1.45541
H	0	0.25452	2.97054	1.29887
H	0	1.34864	2.81698	2.69348
C	0	2.20639	2.57224	-1.00295
H	0	1.20025	2.38287	-1.40398
H	0	2.37974	3.65765	-0.97797
H	0	2.9363	2.10722	-1.67746
C	0	4.02459	2.40703	1.18651
H	0	4.78275	1.88698	0.58696
H	0	4.149	3.49149	1.05299
H	0	4.18464	2.15347	2.24322
Cs	0	-4.27696	-0.90171	-0.5197
C	0	2.70088	-2.29342	1.40982
H	0	3.15528	-2.63346	0.477
C	0	3.43059	-1.44921	2.21832
H	0	4.42995	-1.11996	1.92647
H	0	3.18001	-1.32261	3.27065
H	0	1.7415	0.2137	2.41727
C	0	2.90066	-0.5711	-0.7771
C	0	2.1099	-0.85276	-1.90267
C	0	4.28708	-0.50361	-0.99715
C	0	2.66271	-1.03028	-3.17176
H	0	1.02591	-0.90807	-1.7838
C	0	4.8539	-0.67591	-2.26228
H	0	4.961	-0.30263	-0.15716
C	0	4.04046	-0.93581	-3.36304
H	0	2.01123	-1.24254	-4.02245
H	0	5.93602	-0.60498	-2.38868
H	0	4.4746	-1.06828	-4.35435

Zero-point correction= 0.451889 (Hartree/Particle)
Thermal correction to Energy= 0.492521
Thermal correction to Enthalpy= 0.493607
Thermal correction to Gibbs Free Energy= 0.371425
SCF Done: E(RwB97XD) = -2948.09067862

C1-1

C	0	-0.22797	-2.48925	-0.95056
C	0	-0.84132	-1.48327	-0.00546
O	0	-1.87749	-1.76785	0.631
N	0	-0.10583	-0.36538	0.13152
C	0	-0.58959	0.66318	0.93145
C	0	-1.83351	1.33357	0.64686
C	0	0.18135	1.15787	1.97592
C	0	-2.24917	2.42291	1.4724

C	0	-0.23102	2.2446	2.77106
H	0	1.14291	0.68031	2.17296
C	0	-3.70369	1.55053	-0.67813
C	0	-3.4909	3.03629	1.17677
C	0	-1.43463	2.8658	2.541
H	0	0.41453	2.58344	3.58105
C	0	-4.22978	2.59934	0.10917
H	0	-3.83357	3.86068	1.80375
H	0	-1.77174	3.70179	3.15433
H	0	-5.18524	3.05106	-0.14951
N	0	-2.56541	0.94098	-0.43201
C	0	0.94711	-3.21679	-0.27999
H	0	-4.25722	1.2145	-1.55875
H	0	0.53486	-4.00966	0.37672
H	0	0.14056	-1.96751	-1.84655
H	0	-1.00099	-3.20516	-1.26413
H	0	1.50593	-3.74288	-1.07055
Ni	0	1.86157	-0.4752	-0.2087
P	0	2.03262	1.59522	-1.12287
C	0	0.53912	2.58957	-1.54016
H	0	0.81747	3.49503	-2.09805
H	0	-0.15354	1.99402	-2.14885
H	0	0.01994	2.89094	-0.62066
C	0	2.92332	1.64717	-2.73075
H	0	2.41776	0.99939	-3.45972
H	0	2.96019	2.67091	-3.12914
H	0	3.94799	1.27698	-2.59824
C	0	2.99603	2.83664	-0.16608
H	0	4.01484	2.46485	0.00303
H	0	3.05141	3.79356	-0.70484
H	0	2.52264	3.00621	0.81085
Cs	0	-3.85143	-0.70808	2.71544
C	0	1.89939	-2.31886	0.51732
H	0	2.90609	-2.75757	0.43682
C	0	1.55993	-2.27762	2.00124
H	0	1.5813	-3.28848	2.45511
H	0	0.55878	-1.86546	2.20049
H	0	2.28165	-1.66447	2.56621
C	0	3.72738	-0.65383	-0.42512
C	0	4.30193	-1.28717	-1.54397
C	0	4.62833	-0.16324	0.53792
C	0	5.68191	-1.40451	-1.70438
H	0	3.64868	-1.70056	-2.3203
C	0	6.01227	-0.28098	0.39145

H	0	4.24047	0.33791	1.43232
C	0	6.54973	-0.89984	-0.7353
H	0	6.08567	-1.89748	-2.59085
H	0	6.67549	0.1148	1.16308
H	0	7.62917	-0.99216	-0.85567

Zero-point correction= 0.456386 (Hartree/Particle)
Thermal correction to Energy= 0.496619
Thermal correction to Enthalpy= 0.497705
Thermal correction to Gibbs Free Energy= 0.376672
SCF Done: E(RwB97XD) = -2948.12650765

C'1-1

C	0	-0.39557	-0.8737	3.08063
C	0	-1.40184	-0.04161	3.89903
C	0	-1.64058	1.33979	3.3783
C	0	-2.831	1.84673	3.05793
H	0	-3.74153	1.24564	3.12197
H	0	-2.94632	2.87048	2.6989
Ni	0	-0.91082	1.08574	0.35724
C	0	-0.38372	2.76596	-0.31041
C	0	-0.28346	3.86691	0.54935
C	0	0.10395	5.12247	0.07876
C	0	0.41717	5.30073	-1.26764
C	0	0.33492	4.21447	-2.13592
C	0	-0.06692	2.96473	-1.66013
H	0	-0.14486	2.13316	-2.36723
H	0	0.57874	4.34188	-3.19186
H	0	0.72526	6.27892	-1.63645
H	0	0.16786	5.9655	0.76851
H	0	-0.5083	3.74849	1.61323
N	0	-2.63424	1.15653	-0.47989
C	0	-3.23808	2.20208	-1.02615
C	0	-4.42402	2.09044	-1.77004
C	0	-4.97668	0.84965	-1.96194
C	0	-4.37689	-0.28522	-1.37192
C	0	-4.90876	-1.5899	-1.49226
C	0	-4.28688	-2.62289	-0.83511
C	0	-3.14146	-2.42233	-0.0375
C	0	-2.57578	-1.15539	0.11227
N	0	-1.52948	-0.74388	0.92487
C	0	-1.04255	-1.56012	1.89793
O	0	-1.14207	-2.80194	1.90638
Cs	0	0.52189	-3.10674	-0.88623
C	0	5.06863	-0.3848	-0.35618

C	0	5.20753	0.99701	-0.52206
C	0	6.45548	1.60362	-0.44113
C	0	7.59143	0.8375	-0.17991
C	0	7.46557	-0.53694	-0.00625
C	0	6.21221	-1.14069	-0.09666
H	0	6.11868	-2.22004	0.03815
H	0	8.3459	-1.14468	0.20187
H	0	8.56916	1.31278	-0.11024
H	0	6.5443	2.68097	-0.57893
H	0	4.31703	1.59898	-0.71395
C	0	3.70277	-1.02819	-0.45349
C	0	2.7132	-0.49554	0.54148
C	0	1.67271	0.29601	0.18457
O	0	0.85096	0.90502	1.00318
H	0	1.51696	0.46607	-0.90176
H	0	2.91728	-0.65934	1.60463
H	0	3.83302	-2.12013	-0.35249
H	0	3.30382	-0.859	-1.47007
C	0	-3.20189	-0.08374	-0.60463
H	0	-2.7091	-3.26047	0.49649
H	0	-4.68877	-3.63294	-0.91442
H	0	-5.80381	-1.74733	-2.0928
H	0	-5.88408	0.72188	-2.55222
H	0	-4.87267	2.98654	-2.19146
H	0	-2.76113	3.1698	-0.88638
H	0	-0.74862	1.9698	3.28627
H	0	-2.35929	-0.58083	3.98532
H	0	-1.00029	0.04154	4.92184
H	0	0.02387	-1.66441	3.7153
H	0	0.42042	-0.22545	2.73624

Zero-point correction= 0.487288 (Hartree/Particle)
Thermal correction to Energy= 0.530490
Thermal correction to Enthalpy= 0.531577
Thermal correction to Gibbs Free Energy= 0.399061
SCF Done: E(RwB97XD) = -2909.99375295

C1-2

C	-1.11504	-2.00193	-2.67023
C	0.01056	-1.1291	-2.12928
O	1.15769	-1.63397	-2.10938
N	-0.34286	0.08541	-1.69169
C	0.58263	1.02425	-1.31345
C	0.16602	1.98287	-0.322
C	1.86115	1.1888	-1.85198

C	1.04906	3.01052	0.11401
C	2.71933	2.21646	-1.42492
H	2.19631	0.49472	-2.61829
C	-1.50773	2.74734	1.09961
C	0.57968	3.9111	1.09903
C	2.34326	3.10809	-0.44586
H	3.70532	2.2995	-1.88305
C	-0.69209	3.78146	1.59289
H	1.24135	4.70517	1.44576
H	3.0101	3.90046	-0.1071
H	-1.08574	4.4619	2.34428
N	-1.099	1.88575	0.18647
C	-2.54038	-1.46161	-2.65483
H	-2.52974	2.62982	1.45885
H	-2.61521	-0.52461	-3.22556
H	-1.07485	-2.9511	-2.10877
H	-0.82879	-2.27001	-3.69913
H	-3.18224	-2.19538	-3.17378
Ni	-2.20094	0.40159	-0.52177
Cs	2.91862	-1.20587	0.49362
C	-3.0762	-1.26387	-1.26675
H	-2.85347	-2.08144	-0.57631
C	-4.07106	-0.36528	-0.90522
H	-4.57808	-0.47916	0.05546
H	-4.60185	0.22514	-1.64979
H	-2.68873	1.40551	-1.61063
C	-1.7587	-0.64502	1.07714
C	-0.76004	-1.62598	1.16515
C	-2.46831	-0.37568	2.25715
C	-0.47567	-2.29468	2.35845
H	-0.19569	-1.90171	0.26813
C	-2.19729	-1.03506	3.45821
H	-3.26695	0.37411	2.24937
C	-1.19313	-1.99906	3.51593
H	0.3009	-3.06295	2.38277
H	-2.77602	-0.79872	4.35294
H	-0.97961	-2.52112	4.44869

Zero-point correction= 0.339283 (Hartree/Particle)
Thermal correction to Energy= 0.368966
Thermal correction to Enthalpy= 0.370052
Thermal correction to Gibbs Free Energy= 0.273448
SCF Done: E(RwB97XD) = -2486.94794318

C1-3

C	0.23536	-2.35408	-1.98949
C	-0.687	-1.57342	-1.05983
O	-1.91846	-1.81006	-1.16536
N	-0.09707	-0.78501	-0.17293
C	-0.87561	0.0174	0.6295
C	-1.63723	1.12086	0.09135
C	-0.89097	-0.14831	2.01225
C	-2.38464	1.9628	0.97095
C	-1.62716	0.69174	2.86767
H	-0.31571	-0.97488	2.43274
C	-2.34678	2.30667	-1.75035
C	-3.15321	2.99973	0.38764
C	-2.373	1.73342	2.3667
H	-1.60644	0.50281	3.94115
C	-3.14727	3.17199	-0.97144
H	-3.73944	3.65121	1.03755
H	-2.95277	2.38666	3.01926
H	-3.72648	3.95902	-1.45021
N	-1.62327	1.32714	-1.25474
C	1.6467	-1.87242	-2.19738
H	-2.30627	2.44613	-2.8336
H	2.26427	-2.47459	-0.41775
H	-0.27509	-2.40171	-2.96608
H	0.25114	-3.39206	-1.61929
H	2.32565	-2.6343	-2.593
Ni	2.43139	-0.96637	-0.51587
P	3.10867	-1.24407	1.51234
C	2.40736	-2.66888	2.42753
H	2.72951	-2.6737	3.47833
H	1.31043	-2.63754	2.38709
H	2.73378	-3.59977	1.94604
C	2.83471	0.12761	2.6983
H	3.42066	1.0073	2.4039
H	1.77308	0.41013	2.70445
H	3.13046	-0.17804	3.71194
C	4.90614	-1.54719	1.68041
H	5.46701	-0.67368	1.32502
H	5.17189	-1.73778	2.72991
H	5.19396	-2.41532	1.07361
Cs	-4.57744	-0.81928	0.00762
C	1.98885	-0.54464	-2.46835
H	1.19676	0.20731	-2.40799
C	3.16664	-0.21115	-3.33666
H	3.97406	-0.94892	-3.21501

H	3.58246	0.77956	-3.11286
H	2.88128	-0.21222	-4.40152
C	3.01152	0.88046	-0.41377
C	2.13641	1.94611	-0.14932
C	4.37172	1.21441	-0.54048
C	2.58735	3.25806	0.00248
H	1.06588	1.74776	-0.04672
C	4.83758	2.52173	-0.38859
H	5.09968	0.42758	-0.76563
C	3.94476	3.55455	-0.10924
H	1.8728	4.05677	0.21058
H	5.90347	2.73511	-0.4881
H	4.30108	4.5775	0.01357

Zero-point correction= 0.451472 (Hartree/Particle)
Thermal correction to Energy= 0.492530
Thermal correction to Enthalpy= 0.493616
Thermal correction to Gibbs Free Energy= 0.369603
SCF Done: E(RwB97XD) = -2948.09202383

C'1-2

C	0	0.1116	3.31516	0.02519
C	0	0.79627	2.00177	0.32711
O	0	2.0177	2.01783	0.61279
N	0	0.04243	0.89425	0.29007
C	0	0.75002	-0.28105	0.59437
C	0	1.56687	-0.93448	-0.38824
C	0	0.67582	-0.83656	1.8581
C	0	2.23799	-2.14695	-0.04479
C	0	1.36009	-2.02189	2.19683
H	0	0.06263	-0.3306	2.60545
C	0	2.44567	-0.97414	-2.51415
C	0	3.03419	-2.7614	-1.04131
C	0	2.12669	-2.67771	1.26384
H	0	1.26951	-2.41926	3.2074
C	0	3.14654	-2.17824	-2.27648
H	0	3.55141	-3.69257	-0.80572
H	0	2.6545	-3.60022	1.50763
H	0	3.75184	-2.62145	-3.06452
N	0	1.68496	-0.37462	-1.62483
C	0	-1.33878	3.37201	0.46242
H	0	2.52075	-0.49651	-3.49405
H	0	-1.40808	3.0395	1.51288
H	0	0.17469	3.49754	-1.06217
H	0	0.71265	4.10051	0.50527

H	0	-1.67034	4.43057	0.47032
Ni	0	-1.67635	0.69253	-0.6705
P	0	-1.91771	-1.52707	-1.02283
C	0	-1.4397	-2.8992	0.11608
H	0	-1.85512	-3.85231	-0.2428
H	0	-0.3502	-2.99948	0.18807
H	0	-1.83654	-2.70008	1.1213
C	0	-1.26837	-2.18088	-2.61705
H	0	-0.17156	-2.23581	-2.5816
H	0	-1.66744	-3.18318	-2.83093
H	0	-1.54966	-1.50099	-3.43236
C	0	-3.70448	-1.93715	-1.19092
H	0	-4.14866	-1.35408	-2.0073
H	0	-3.84929	-3.00891	-1.39097
H	0	-4.23098	-1.67538	-0.26172
Cs	0	4.63973	0.49138	0.88671
C	0	-2.25244	2.5565	-0.4374
H	0	-2.23358	3.05072	-1.42527
C	0	-3.70555	2.56786	0.05593
H	0	-4.38199	2.58811	-0.81302
H	0	-3.90846	3.51028	0.60875
C	0	-4.14501	1.41054	0.92521
C	0	-3.31796	0.84422	1.90506
C	0	-5.41996	0.86096	0.76038
C	0	-3.74502	-0.23556	2.67412
H	0	-2.31998	1.24807	2.06919
C	0	-5.8579	-0.21377	1.53227
H	0	-6.08051	1.27949	-0.00165
C	0	-5.01848	-0.7728	2.49184
H	0	-3.07611	-0.65813	3.42466
H	0	-6.8569	-0.62056	1.37513
H	0	-5.35284	-1.6166	3.09447
H	0	-2.84208	0.7188	-1.63146

Zero-point correction= 0.455839 (Hartree/Particle)
Thermal correction to Energy= 0.494999
Thermal correction to Enthalpy= 0.496086
Thermal correction to Gibbs Free Energy= 0.378364
SCF Done: E(RwB97XD) = -2948.12499597

C2

C	0	-3.32946	0.37211	0.24684
C	0	-4.17431	1.24707	0.95464
C	0	-5.4194	1.64073	0.46252
C	0	-5.86838	1.17155	-0.77121

C	0	-3.81636	-0.09095	-0.98909
C	0	-5.05642	0.30097	-1.49644
H	0	-3.84934	1.64403	1.92173
H	0	-6.04417	2.32082	1.04485
H	0	-3.21332	-0.79028	-1.57869
H	0	-5.39373	-0.0781	-2.46318
C	0	2.40537	0.93778	1.6171
C	0	1.10843	0.76188	1.14323
C	0	0.46052	1.90242	0.57663
C	0	1.13956	3.14001	0.42878
C	0	2.46787	3.26067	0.89803
C	0	3.06668	2.17553	1.4954
H	0	2.92282	0.10091	2.07488
C	0	0.4328	4.20129	-0.18272
H	0	2.98527	4.21411	0.79517
H	0	4.0808	2.26376	1.88468
C	0	-0.8579	4.00429	-0.6027
C	0	-1.46	2.74918	-0.39648
H	0	0.92695	5.16491	-0.30876
H	0	-1.42852	4.79788	-1.07942
H	0	-2.49233	2.575	-0.6996
N	0	-0.83554	1.73383	0.17629
N	0	0.34255	-0.39828	1.18742
C	0	0.96174	-1.59915	1.11147
O	0	2.17804	-1.77212	0.89051
C	0	-2.16024	-1.68665	1.6168
C	0	0.10943	-2.8287	1.3409
H	0	0.63224	-3.66374	0.85338
H	0	0.1677	-3.03328	2.42521
C	0	-1.35522	-2.79075	0.93995
H	0	-1.44306	-2.71748	-0.16053
H	0	-1.78237	-3.78248	1.19358
H	0	-1.7681	-1.55795	2.64619
C	0	-3.61813	-2.09742	1.72604
H	0	-4.07301	-2.27051	0.7377
H	0	-3.72349	-3.0422	2.29339
H	0	-4.23583	-1.34233	2.23258
Ni	0	-1.57769	-0.02731	0.799
Cs	0	2.74201	-0.27903	-1.86126
H	0	-6.83899	1.47859	-1.1611

Zero-point correction= 0.342862 (Hartree/Particle)
Thermal correction to Energy= 0.372740
Thermal correction to Enthalpy= 0.373826
Thermal correction to Gibbs Free Energy= 0.274566

SCF Done: E(RwB97XD) = -2486.99722426

C'2

C	-1.38897	-2.83971	-1.58055
C	-2.17347	-2.0276	-0.57824
O	-3.35252	-2.33813	-0.32806
N	-1.51279	-0.97442	-0.03472
C	-2.13644	-0.21003	0.95033
C	-1.85737	1.18648	0.90554
C	-2.97026	-0.66489	1.96299
C	-2.46135	2.09619	1.80823
C	-3.55157	0.23661	2.87886
H	-3.1892	-1.72582	2.04324
C	-0.72539	2.87689	-0.19435
C	-2.14735	3.46623	1.64515
C	-3.32739	1.5931	2.80708
H	-4.19831	-0.16205	3.6601
C	-1.29325	3.85677	0.64357
H	-2.59088	4.19697	2.32182
H	-3.78717	2.28363	3.5134
H	-1.03859	4.9031	0.49252
N	-0.99022	1.59103	-0.0647
C	0.09538	-2.90034	-1.27669
H	-0.02928	3.14694	-0.98798
H	0.23228	-3.19786	-0.22221
H	-1.54754	-2.40304	-2.58209
H	-1.84438	-3.83928	-1.60365
H	0.55177	-3.71142	-1.87631
Ni	-0.08818	-0.01733	-0.8986
Cs	-5.40151	-0.001	-0.73201
C	0.81482	-1.59553	-1.54977
H	0.78381	-1.39501	-2.63801
C	2.28172	-1.68661	-1.13697
H	2.81915	-0.78921	-1.48118
H	2.75167	-2.535	-1.6715
C	2.56582	-1.8719	0.33389
C	1.98863	-1.05652	1.31672
C	3.48111	-2.84271	0.75352
C	2.32389	-1.19804	2.66026
H	1.26185	-0.29508	1.01937
C	3.82191	-2.98974	2.09662
H	3.94242	-3.49157	0.00652
C	3.24702	-2.16311	3.05763
H	1.86202	-0.54701	3.40262

H	4.53899	-3.75578	2.39175
H	3.51046	-2.27334	4.10895
C	6.86848	1.92707	-0.92534
C	5.61262	1.6089	-0.40761
C	5.46259	0.39657	0.27547
C	6.53792	-0.46919	0.4383
C	7.78777	-0.1413	-0.08756
C	7.94928	1.05966	-0.77067
H	7.00057	2.86969	-1.45981
H	4.48325	0.13339	0.68494
C	2.15078	1.58448	-1.06366
O	1.18803	1.06156	-1.78395
H	1.93523	1.76795	0.01026
C	3.38045	1.94047	-1.50147
H	3.65385	1.74571	-2.54373
C	4.42545	2.52544	-0.59791
H	4.79195	3.4973	-0.97082
H	3.96606	2.72749	0.38494
H	8.92116	1.32596	-1.18584
H	8.63033	-0.82074	0.03636
H	6.40068	-1.40573	0.97942 Zero-point correction=

0.490546 (Hartree/Particle)

Thermal correction to Energy= 0.531775

Thermal correction to Enthalpy= 0.532862

Thermal correction to Gibbs Free Energy= 0.406524

SCF Done: E(RwB97XD) = -2910.02999748

C3

C	-2.58843	-0.5182	0.10823
C	-2.36812	-1.90804	0.50875
C	-2.04912	-2.2252	1.86886
C	-1.82085	-1.2452	2.79999
C	-2.34072	0.46127	1.14266
C	-1.95624	0.12102	2.41562
H	-2.82649	-2.70316	-0.0938
H	-1.99864	-3.27657	2.16205
H	-2.5115	1.51724	0.91316
H	-1.79541	0.90629	3.15797
C	2.35794	0.96846	-1.92978
C	1.40934	0.03082	-1.52505
C	1.88124	-1.11926	-0.80633
C	3.2622	-1.30406	-0.54002
C	4.18664	-0.32372	-0.973
C	3.72491	0.7839	-1.65118

H	2.03183	1.85431	-2.47176
C	3.63358	-2.46805	0.1752
H	5.24678	-0.4594	-0.76012
H	4.43278	1.54167	-1.98802
C	2.66642	-3.34974	0.58995
C	1.31672	-3.0716	0.30346
H	4.68773	-2.64658	0.3891
H	2.92171	-4.2542	1.13796
H	0.52896	-3.74924	0.63441
N	0.9264	-1.99736	-0.36546
N	0.06468	0.03924	-1.80808
C	-0.66898	1.17861	-1.87587
O	-0.3569	2.28619	-1.39448
C	-3.58695	-0.16694	-0.98514
C	-1.98462	1.03638	-2.62085
H	-2.01104	1.86837	-3.34213
H	-1.98569	0.09995	-3.19697
C	-3.2483	1.1076	-1.76298
H	-3.17783	1.96955	-1.07802
H	-4.09888	1.32963	-2.42666
H	-3.5867	-1.00141	-1.71054
C	-4.99604	-0.05861	-0.40199
H	-5.06367	0.78551	0.30192
H	-5.75112	0.09923	-1.18625
H	-5.26663	-0.97063	0.14948
Ni	-0.87498	-1.16745	-0.51253
Cs	1.1131	1.98398	1.4047
H	-1.56344	-1.50014	3.82811

Zero-point correction= 0.344288 (Hartree/Particle)
Thermal correction to Energy= 0.373465
Thermal correction to Enthalpy= 0.374552
Thermal correction to Gibbs Free Energy= 0.280055
SCF Done: E(RwB97XD) = -2487.01246340

C'3

C	0	-1.61016	-2.81304	-2.70324
C	0	-2.47988	-2.01593	-1.73685
O	0	-3.4676	-1.43063	-2.23321
N	0	-2.08512	-1.98124	-0.45482
C	0	-2.82706	-1.40118	0.54817
C	0	-2.10576	-1.0557	1.74852
C	0	-4.20239	-1.16165	0.55086
C	0	-2.7762	-0.49294	2.86929
C	0	-4.85461	-0.60582	1.66663

H	0	-4.77422	-1.41072	-0.33767
C	0	-0.06719	-0.996	2.87438
C	0	-2.00295	-0.1816	4.01218
C	0	-4.16955	-0.26533	2.80949
H	0	-5.93113	-0.44347	1.61307
C	0	-0.65484	-0.43092	4.02044
H	0	-2.49949	0.25115	4.88093
H	0	-4.67417	0.17074	3.67105
H	0	-0.03733	-0.20889	4.88727
N	0	-0.75981	-1.28501	1.7875
C	0	-0.38826	-3.56727	-2.18994
H	0	0.99928	-1.22222	2.84827
H	0	-0.67056	-4.31724	-1.43652
H	0	-1.29152	-2.10221	-3.48485
H	0	-2.28947	-3.51038	-3.21644
H	0	0.0347	-4.12783	-3.04229
Ni	0	0.00556	-1.93354	0.09623
Cs	0	-2.33721	1.52065	-1.31817
C	0	0.66427	-2.64685	-1.64579
H	0	0.8226	-1.73673	-2.232
C	0	1.61832	-2.98296	-0.68859
H	0	1.59712	-3.99255	-0.27098
H	0	-0.15741	-3.26052	0.69808
C	0	3.26697	4.88224	-0.32565
C	0	3.08786	3.59456	0.18052
C	0	4.18515	2.72846	0.19969
C	0	5.42836	3.13839	-0.26766
C	0	5.59469	4.42733	-0.77522
C	0	4.50947	5.29726	-0.80406
H	0	2.41976	5.5707	-0.34325
H	0	4.04897	1.71617	0.58796
C	0	0.86867	0.82265	0.10909
O	0	0.28859	-0.09203	-0.61353
H	0	1.2083	0.52883	1.12652
C	0	1.09458	2.11611	-0.2435
H	0	0.83978	2.44856	-1.25667
C	0	1.74328	3.11435	0.67201
H	0	1.09797	3.9963	0.82968
H	0	1.86932	2.64755	1.66384
H	0	4.6289	6.30655	-1.19759
H	0	6.56738	4.75016	-1.14457
H	0	6.27583	2.4529	-0.23578
C	0	2.84699	-2.22833	-0.38857
C	0	3.52407	-2.49958	0.80982

C	0	3.38404	-1.26258	-1.25004
C	0	4.68916	-1.82153	1.1451
H	0	3.11401	-3.25233	1.48647
C	0	4.55383	-0.58859	-0.9179
H	0	2.88966	-1.04625	-2.19655
C	0	5.21153	-0.86143	0.28008
H	0	5.1965	-2.0475	2.08238
H	0	4.95666	0.15656	-1.60385
H	0	6.12997	-0.33398	0.53595

Zero-point correction= 0.486441 (Hartree/Particle)
Thermal correction to Energy= 0.528246
Thermal correction to Enthalpy= 0.529332
Thermal correction to Gibbs Free Energy= 0.403216
SCF Done: E(RwB97XD) = -2909.99301338

C4

C	0.55952	2.58323	-1.25389
C	0.46325	1.10901	-1.65558
O	1.47165	0.60811	-2.21598
N	-0.6238	0.38559	-1.40458
C	-1.81623	0.85637	-0.94087
C	-2.49548	0.10356	0.09459
C	-2.49962	1.95794	-1.46433
C	-3.78322	0.51703	0.55129
C	-3.76883	2.34851	-1.00495
H	-2.03368	2.51725	-2.2756
C	-2.48103	-1.67373	1.56158
C	-4.39176	-0.24751	1.57437
C	-4.41322	1.65243	-0.00933
H	-4.24579	3.21958	-1.45547
C	-3.74699	-1.34295	2.087
H	-5.37515	0.05115	1.94035
H	-5.39726	1.95042	0.35288
H	-4.18992	-1.95034	2.87391
N	-1.87383	-0.99144	0.61259
C	1.91536	3.00891	-0.70381
H	-1.95139	-2.54857	1.95101
H	2.71433	2.50734	-1.27095
H	-0.23523	2.85708	-0.54411
H	0.35641	3.16131	-2.16992
H	2.04598	4.09004	-0.86939
Cs	0.83783	-2.34817	-0.97443
C	2.0972	2.75674	0.79638
H	1.2183	3.18993	1.30512

C	3.3359	3.49013	1.30668
H	3.23375	4.57375	1.15632
H	4.24114	3.16994	0.77075
H	3.50176	3.31106	2.37779
C	2.14726	1.29221	1.17512
C	3.25611	0.49943	0.8517
C	1.11915	0.71153	1.9213
C	3.35459	-0.81335	1.30139
H	4.06594	0.92238	0.25539
C	1.2094	-0.60521	2.37176
H	0.24055	1.30953	2.17206
C	2.33551	-1.36826	2.07704
H	4.23708	-1.40435	1.05588
H	0.40096	-1.02806	2.96806
H	2.42062	-2.39016	2.44699

Zero-point correction= 0.343943 (Hartree/Particle)
Thermal correction to Energy= 0.371635
Thermal correction to Enthalpy= 0.372722
Thermal correction to Gibbs Free Energy= 0.280284
SCF Done: E(RwB97XD) = -978.606910722

C'4

C	1.9515	-1.76896	2.18236
C	1.61394	-2.19463	0.75414
O	1.05051	-3.31551	0.63925
N	1.79226	-1.38311	-0.27909
C	2.4517	-0.18382	-0.23392
C	1.76295	1.01021	-0.66837
C	3.79454	-0.04623	0.1186
C	2.44967	2.26041	-0.69293
C	4.46042	1.19171	0.08209
H	4.33896	-0.94191	0.42124
C	-0.18247	1.99058	-1.41784
C	1.71696	3.39993	-1.10367
C	3.80979	2.33729	-0.31202
H	5.51071	1.23413	0.3717
C	0.40109	3.2746	-1.46561
H	2.21891	4.36819	-1.12701
H	4.31772	3.3014	-0.34065
H	-0.18944	4.13245	-1.78248
N	0.45602	0.90547	-1.03739
C	0.6635	-1.49267	2.96574
H	-1.23232	1.86676	-1.70433
H	0.01867	-2.38386	2.94417

H	2.59263	-0.87824	2.21828
H	2.49676	-2.59163	2.66585
H	0.9255	-1.30845	4.02074
Cs	-1.17143	-1.98038	-1.34185
C	-0.07113	-0.31059	2.42729
H	0.50641	0.61919	2.37724
C	-1.34429	-0.32821	2.01008
H	-1.88773	-1.27957	2.05996
C	-2.11687	0.79993	1.48126
C	-1.65252	2.12445	1.49885
C	-3.37955	0.55408	0.92254
C	-2.42112	3.15593	0.9755
H	-0.674	2.35236	1.92342
C	-4.14695	1.58539	0.38958
H	-3.76526	-0.46737	0.91837
C	-3.67062	2.8935	0.41447
H	-2.03909	4.17619	1.00173
H	-5.12448	1.3665	-0.03893
H	-4.27035	3.70516	0.00511

Zero-point correction= 0.320994 (Hartree/Particle)
Thermal correction to Energy= 0.348022
Thermal correction to Enthalpy= 0.349108
Thermal correction to Gibbs Free Energy= 0.257229
SCF Done: E(RwB97XD) = -977.378286138

cod

C	1.91303	0.03784	-0.02455
C	1.1089	-1.06424	0.6696
H	2.4225	-0.4016	-0.89878
H	1.81726	-1.84958	0.96937
H	0.67929	-0.68819	1.60494
C	-1.10888	1.06422	0.66963
H	-1.81721	1.84955	0.96948
H	-0.67923	0.68811	1.60493
C	-1.91303	-0.03782	-0.02456
H	-2.73228	-0.35894	0.64164
H	-2.42245	0.40166	-0.8988
C	-0.06273	1.68319	-0.21687
H	-0.37722	2.60252	-0.7191
C	1.17118	1.25154	-0.49739
H	1.75501	1.87319	-1.183
C	-1.1712	-1.25153	-0.49738
H	-1.75505	-1.8732	-1.18295
C	0.06271	-1.68319	-0.21688

H	0.37716	-2.60255	-0.71908
H	2.73224	0.35898	0.64169

Zero-point correction= 0.179471 (Hartree/Particle)
Thermal correction to Energy= 0.189312
Thermal correction to Enthalpy= 0.190399
Thermal correction to Gibbs Free Energy= 0.141662
SCF Done: E(RwB97XD) = -312.038921455

$Ni(COD)_2$

C	2.20253	-1.8142	0.63017
C	2.59921	-0.55946	1.41721
H	1.62254	-2.47578	1.29326
H	2.8596	-0.85455	2.44378
H	3.51469	-0.11737	1.00055
C	2.59673	0.56049	-1.41921
H	2.85412	0.85657	-2.44624
H	3.51375	0.11877	-1.00547
C	2.20159	1.81466	-0.6303
H	3.10583	2.38852	-0.35661
H	1.62137	2.47736	-1.29203
C	1.50045	-0.48091	-1.46559
H	0.9605	-0.55956	-2.41185
C	1.35591	-1.53431	-0.58417
H	0.73892	-2.37399	-0.91597
C	1.35611	1.53432	0.58473
H	0.73793	2.37305	0.91659
C	1.50221	0.48094	1.46551
H	0.96196	0.55763	2.41173
H	3.10608	-2.38898	0.35608
C	-2.20264	-1.81409	-0.63007
C	-2.59887	-0.55941	-1.41748
H	-1.62278	-2.47601	-1.29293
H	-2.85878	-0.85468	-2.44411
H	-3.51455	-0.11726	-1.00134
C	-2.59707	0.56043	1.41909
H	-2.85484	0.85642	2.44605
H	-3.5139	0.11872	1.00496
C	-2.20161	1.81464	0.63039
H	-3.10572	2.38864	0.35655
H	-1.62147	2.47719	1.29234
C	-1.50075	-0.48088	1.46572
H	-0.96061	-0.5592	2.41189
C	-1.35604	-1.53422	0.58431
H	-0.73878	-2.37372	0.91599

C	-1.35588	1.53429	-0.58447
H	-0.73772	2.37309	-0.91622
C	-1.50186	0.48103	-1.46549
H	-0.96175	0.55804	-2.41175
H	-3.10641	-2.3885	-0.35595
Ni	0.	-0.00087	-0.00006

Zero-point correction= 0.362137 (Hartree/Particle)
Thermal correction to Energy= 0.383997
Thermal correction to Enthalpy= 0.385084
Thermal correction to Gibbs Free Energy= 0.311190
SCF Done: E(RwB97XD) = -2132.51401948

$PhB(OH)_2$

B	1.73748	0.	-0.00001
O	2.37871	1.20412	0.08691
H	3.34416	1.13081	0.08486
O	2.3787	-1.20412	-0.08689
H	3.34415	-1.1308	-0.08482
C	0.17261	0.00001	-0.00001
C	-0.55083	-1.19918	0.04041
C	-0.55084	1.19918	-0.04043
C	-1.94169	-1.20442	0.04361
H	-0.01199	-2.14656	0.07269
C	-1.94171	1.20441	-0.04361
H	-0.01202	2.14658	-0.07273
C	-2.63969	0.	0.00001
H	-2.48444	-2.14836	0.07959
H	-2.48446	2.14836	-0.07958
H	-3.72924	-0.00002	0.00002

Zero-point correction= 0.124915 (Hartree/Particle)
Thermal correction to Energy= 0.135073
Thermal correction to Enthalpy= 0.136160
Thermal correction to Gibbs Free Energy= 0.085834
SCF Done: E(RwB97XD) = -408.288978202

PMe3

P	0	-1.53834	-0.92251	0.00024
C	0	-0.64817	-0.11165	-1.40554
H	0	-0.93148	-0.58765	-2.35338
H	0	-0.92879	0.948	-1.46575
H	0	0.44362	-0.18022	-1.29007
C	0	-0.64557	-0.11353	1.40546
H	0	-0.92577	0.94611	1.46744
H	0	-0.92733	-0.59065	2.3532

H	0	0.44597	-0.18233	1.28797
C	0	-0.65091	-2.5469	-0.00152
H	0	-0.93312	-3.12937	0.88522
H	0	-0.93455	-3.12822	-0.88855
H	0	0.44109	-2.4144	-0.00236

Zero-point correction= 0.112331 (Hartree/Particle)
Thermal correction to Energy= 0.120752
Thermal correction to Enthalpy= 0.121839
Thermal correction to Gibbs Free Energy= 0.077411
SCF Done: E(RwB97XD) = -461.117889858

S

C	0.0263	2.90329	0.51637
C	0.64135	2.00188	-0.55382
N	-0.05227	0.99163	-1.06031
C	-1.38981	0.77907	-0.84904
C	-1.82298	-0.43196	-0.18918
C	-3.21651	-0.71377	-0.06138
C	-4.17925	0.18501	-0.57711
C	-3.75538	1.3346	-1.20095
C	-2.38618	1.62491	-1.3355
N	-0.87458	-1.27377	0.30997
C	-1.2662	-2.37052	0.9245
C	-2.61587	-2.74041	1.10024
C	-3.58423	-1.90775	0.60293
C	-0.03837	2.18363	1.86862
C	1.29639	1.70096	2.33623
C	1.57388	0.44185	2.67591
O	1.85376	2.19517	-0.83187
Cs	2.39653	-0.99507	-0.59419
H	0.65738	3.79848	0.60391
H	-0.98524	3.23287	0.24426
H	-5.23836	-0.05	-0.47053
H	-4.48595	2.0358	-1.60503
H	-2.07668	2.53128	-1.85785
H	-0.47767	-3.02035	1.31587
H	-2.86709	-3.66371	1.61856
H	-4.64323	-2.14639	0.71104
H	-0.45247	2.88283	2.61356
H	-0.73698	1.33403	1.81179
H	2.0862	2.45775	2.39315
H	2.56034	0.14377	3.03295
H	0.80962	-0.33847	2.62916

Zero-point correction= 0.239017 (Hartree/Particle)

Thermal correction to Energy= 0.260563
Thermal correction to Enthalpy= 0.261649
Thermal correction to Gibbs Free Energy= 0.180534
SCF Done: E(RwB97XD) = -746.317208317

t-AmylO-

O	0.4091	0.00062	1.48519
C	0.43543	-0.00129	0.13963
C	1.65464	-0.78712	-0.40281
H	1.72684	-0.81954	-1.50436
H	2.58339	-0.33831	-0.01532
H	1.61678	-1.82477	-0.03337
C	0.54191	1.43469	-0.43669
H	0.53488	1.48874	-1.53987
H	-0.28384	2.05889	-0.06163
H	1.47618	1.89836	-0.08133
C	-0.82917	-0.6821	-0.47511
H	-0.77607	-0.71819	-1.57948
H	-0.81608	-1.7309	-0.12726
C	-2.1281	-0.03613	-0.03074
H	-2.29877	0.93506	-0.51838
H	-3.00815	-0.66096	-0.23961
H	-2.07625	0.13843	1.05344

Zero-point correction= 0.149356 (Hartree/Particle)
Thermal correction to Energy= 0.159172
Thermal correction to Enthalpy= 0.160258
Thermal correction to Gibbs Free Energy= 0.112164
SCF Done: E(RwB97XD) = -272.488904326

t-AmylOB(OH)$_2$

O	0.32623	-0.51594	-0.2771
C	-0.71339	0.462	-0.10933
C	-0.4644	1.63706	-1.04202
H	-1.2862	2.36274	-0.97122
H	0.46875	2.15622	-0.79379
H	-0.40475	1.29288	-2.08392
C	-0.78616	0.91844	1.34042
H	-1.70381	1.49961	1.50729
H	-0.79851	0.05945	2.02412
H	0.06962	1.55047	1.60366
C	-2.00305	-0.24543	-0.53276
H	-2.81423	0.49918	-0.5241
H	-1.8766	-0.55811	-1.58147
C	-2.39217	-1.44564	0.31165

H	-2.65912	-1.16121	1.33831
H	-3.26223	-1.95789	-0.11888
H	-1.57303	-2.17449	0.3661
B	1.65383	-0.35489	-0.02567
O	2.15873	0.82258	0.46432
H	3.11714	0.80103	0.59022
O	2.42107	-1.45961	-0.30187
H	3.36065	-1.33028	-0.11852

Zero-point correction= 0.189114 (Hartree/Particle)
Thermal correction to Energy= 0.203289
Thermal correction to Enthalpy= 0.204376
Thermal correction to Gibbs Free Energy= 0.145988
SCF Done: E(RwB97XD) = -449.092709338

t-AmylOH

O	0.41361	0.06081	1.44315
C	0.42503	-0.00776	0.01273
C	1.63441	-0.812	-0.44146
H	1.68115	-0.88061	-1.53728
H	2.56552	-0.33922	-0.09568
H	1.59689	-1.83134	-0.03306
C	0.48456	1.39874	-0.56467
H	0.39215	1.38611	-1.65986
H	-0.31542	2.03021	-0.1576
H	1.44526	1.87382	-0.31607
C	-0.85693	-0.73967	-0.37828
H	-0.82304	-0.93274	-1.46195
H	-0.83097	-1.72497	0.1141
C	-2.14693	-0.02292	-0.01795
H	-2.28337	0.89734	-0.60158
H	-3.01709	-0.66223	-0.21492
H	-2.16702	0.24712	1.04578
H	1.20622	0.55178	1.71062

Zero-point correction= 0.163660 (Hartree/Particle)
Thermal correction to Energy= 0.173921
Thermal correction to Enthalpy= 0.175008
Thermal correction to Gibbs Free Energy= 0.126382
SCF Done: E(RwB97XD) = -273.013166708

TS1A

C	0	-0.88386	-3.15659	-2.33887
C	0	0.16563	-2.2275	-1.74479
O	0	1.24885	-2.73286	-1.34801
N	0	-0.1541	-0.93918	-1.73421

C	0	0.72076	-0.02599	-1.17944
C	0	0.90754	0.09395	0.24569
C	0	1.38951	0.88846	-1.98748
C	0	1.72625	1.1428	0.76829
C	0	2.21332	1.90079	-1.46368
H	0	1.2497	0.80701	-3.06649
C	0	0.4649	-0.69978	2.3635
C	0	1.87401	1.21913	2.17491
C	0	2.38053	2.04321	-0.10485
H	0	2.71493	2.58425	-2.14902
C	0	1.24934	0.30165	2.97876
H	0	2.48935	2.0145	2.59806
H	0	3.00753	2.83048	0.31446
H	0	1.34268	0.33454	4.06247
N	0	0.29612	-0.80589	1.06382
C	0	-1.58681	-2.61786	-3.58474
H	0	-0.04959	-1.43808	2.98492
H	0	-0.82763	-2.17086	-4.24708
H	0	-1.63145	-3.39181	-1.56305
H	0	-0.37455	-4.10042	-2.57896
H	0	-2.00314	-3.47656	-4.13847
Ni	0	-2.39355	-0.15796	-1.98498
P	0	-2.57388	1.5903	-0.70802
C	0	-1.35913	2.96173	-0.83848
H	0	-1.69729	3.8356	-0.2631
H	0	-0.3823	2.64479	-0.44955
H	0	-1.23321	3.24867	-1.89053
C	0	-2.68741	1.41906	1.11595
H	0	-1.77171	0.95382	1.50745
H	0	-2.81393	2.40635	1.58275
H	0	-3.53961	0.78225	1.38549
C	0	-4.14099	2.46166	-1.093
H	0	-4.99077	1.78986	-0.91462
H	0	-4.26202	3.36163	-0.47301
H	0	-4.145	2.75046	-2.15268
Cs	0	3.81016	-1.89533	0.11369
C	0	-2.70529	-1.62496	-3.38153
H	0	-3.67531	-2.04694	-3.11355
C	0	-2.68207	-0.38124	-4.02372
H	0	-3.61049	0.15531	-4.23232
H	0	-1.86305	-0.14555	-4.70637
H	0	-1.90874	0.90546	-2.96719
C	0	-3.44527	-1.19377	-0.70809
C	0	-2.94132	-1.88977	0.40508

C	0	-4.84674	-1.17679	-0.83552
C	0	-3.77676	-2.51405	1.3334
H	0	-1.8606	-1.92387	0.56989
C	0	-5.6953	-1.78545	0.09132
H	0	-5.3012	-0.66262	-1.69085
C	0	-5.16202	-2.45985	1.18789
H	0	-3.34196	-3.04241	2.18463
H	0	-6.77741	-1.73519	-0.04473
H	0	-5.81665	-2.93949	1.91595

Zero-point correction= 0.451139 (Hartree/Particle)
Thermal correction to Energy= 0.491260
Thermal correction to Enthalpy= 0.492347
Thermal correction to Gibbs Free Energy= 0.371428
SCF Done: E(RwB97XD) = -2948.08709341

TS1B

C	-1.39923	-1.43765	-2.7495
C	-0.16458	-0.70996	-2.23465
O	0.92253	-1.33876	-2.2741
N	-0.37667	0.50512	-1.7228
C	0.65921	1.23654	-1.19462
C	0.41534	1.92902	0.04387
C	1.91303	1.40798	-1.78204
C	1.43548	2.72261	0.64221
C	2.90739	2.20803	-1.19303
H	2.11724	0.90474	-2.7246
C	-1.04626	2.40922	1.78873
C	1.14338	3.36321	1.86939
C	2.69156	2.84956	0.00579
H	3.86683	2.31435	-1.69991
C	-0.09015	3.2047	2.445
H	1.91061	3.97951	2.33863
H	3.46056	3.46605	0.47073
H	-0.34788	3.6842	3.38642
N	-0.81163	1.80083	0.63939
C	-2.7265	-0.69101	-2.74887
H	-2.0402	2.27563	2.21541
H	-2.60654	0.27029	-3.27199
H	-1.48746	-2.36095	-2.15149
H	-1.1638	-1.77995	-3.76932
H	-3.44469	-1.27832	-3.34816
Ni	-2.16125	0.72528	-0.30043
Cs	2.83881	-1.41392	0.22127
C	-3.34387	-0.43811	-1.39496

H	-3.61687	-1.31958	-0.81183
C	-4.0167	0.77352	-1.15447
H	-4.71535	0.8552	-0.31782
H	-4.2468	1.441	-1.98575
H	-2.68475	2.01645	-0.94081
C	-1.88485	-0.78854	0.90568
C	-0.89601	-1.7811	0.81303
C	-2.70446	-0.84966	2.04733
C	-0.71688	-2.75663	1.79721
H	-0.24791	-1.81465	-0.06749
C	-2.53771	-1.81512	3.04264
H	-3.50933	-0.11601	2.17321
C	-1.53587	-2.77652	2.92428
H	0.06018	-3.51587	1.67791
H	-3.19708	-1.82082	3.91273
H	-1.40213	-3.53584	3.69499

Zero-point correction= 0.337935 (Hartree/Particle)
Thermal correction to Energy= 0.367327
Thermal correction to Enthalpy= 0.368413
Thermal correction to Gibbs Free Energy= 0.272147
SCF Done: E(RwB97XD) = -2486.94301532

TS1C

C	0	-2.8538	-0.4801	0.00801
C	0	-4.0586	0.28402	0.08817
C	0	-4.78161	0.64044	-1.03885
C	0	-4.37124	0.24124	-2.31817
C	0	-2.49439	-0.91201	-1.30014
C	0	-3.22349	-0.54276	-2.42842
H	0	-4.40278	0.62407	1.06819
H	0	-5.67757	1.2539	-0.92547
H	0	-1.60794	-1.53714	-1.43082
H	0	-2.89009	-0.88126	-3.4122
C	0	2.65747	0.97309	1.48409
C	0	1.27855	0.86201	1.36961
C	0	0.57506	1.95672	0.77842
C	0	1.25897	3.12468	0.3479
C	0	2.66202	3.19799	0.50774
C	0	3.33661	2.13124	1.06068
H	0	3.21555	0.14251	1.91657
C	0	0.48952	4.1363	-0.2768
H	0	3.18912	4.09356	0.1792
H	0	4.41935	2.17843	1.17568
C	0	-0.85688	3.94504	-0.46928

C	0	-1.44917	2.74442	-0.0321
H	0	0.98221	5.05029	-0.60946
H	0	-1.47241	4.69935	-0.95477
H	0	-2.51338	2.5528	-0.19302
N	0	-0.77147	1.78822	0.58244
N	0	0.53295	-0.21823	1.81895
C	0	0.65907	-1.40189	1.1779
O	0	1.32463	-1.61865	0.13772
C	0	-2.52887	-1.68456	1.31722
C	0	-0.13947	-2.54427	1.78367
H	0	0.46992	-3.45609	1.69877
H	0	-0.31905	-2.35677	2.85223
C	0	-1.47976	-2.78148	1.08091
H	0	-1.29991	-2.92362	0.0021
H	0	-1.88422	-3.74731	1.43215
H	0	-2.3562	-1.2228	2.32395
C	0	-3.91465	-2.32144	1.39826
H	0	-4.1892	-2.78229	0.43547
H	0	-3.92079	-3.11432	2.16277
H	0	-4.69911	-1.60149	1.65805
Ni	0	-1.42296	0.01259	1.08623
Cs	0	0.9899	0.33698	-2.31422
H	0	-4.94349	0.5237	-3.20092

Zero-point correction= 0.341819 (Hartree/Particle)
Thermal correction to Energy= 0.370854
Thermal correction to Enthalpy= 0.371941
Thermal correction to Gibbs Free Energy= 0.277115
SCF Done: E(RwB97XD) = -2486.95014741

TS1D

C	0.22079	-2.52276	-1.79716
C	-0.69758	-1.68476	-0.91367
O	-1.93333	-1.89393	-1.02977
N	-0.10014	-0.88338	-0.04451
C	-0.86651	-0.04936	0.73817
C	-1.5889	1.07129	0.18258
C	-0.90308	-0.20278	2.1218
C	-2.3117	1.94862	1.04831
C	-1.62023	0.66752	2.96287
H	-0.3579	-1.04262	2.55565
C	-2.23776	2.26819	-1.67483
C	-3.02783	3.01456	0.45115
C	-2.32358	1.73035	2.44569
H	-1.61739	0.48638	4.03785

C	-3.00255	3.17638	-0.90908
H	-3.59011	3.69609	1.09126
H	-2.8856	2.41003	3.08665
H	-3.54143	3.98511	-1.39867
N	-1.5593	1.26359	-1.16547
C	1.62343	-2.03964	-2.05719
H	-2.18636	2.39379	-2.75936
H	2.2826	-2.49184	-0.30449
H	-0.29871	-2.64747	-2.76161
H	0.25444	-3.53009	-1.35091
H	2.30257	-2.81629	-2.42292
Ni	2.42935	-0.9888	-0.47331
P	3.15361	-1.12926	1.55741
C	2.51738	-2.52277	2.56472
H	2.85748	-2.45795	3.60799
H	1.41952	-2.52802	2.54255
H	2.867	-3.46852	2.13054
C	2.83776	0.29913	2.66272
H	3.36796	1.18687	2.29566
H	1.76239	0.52543	2.68062
H	3.17385	0.07457	3.68487
C	4.96365	-1.34934	1.72487
H	5.48628	-0.47796	1.31096
H	5.24469	-1.46227	2.78161
H	5.28325	-2.24163	1.17145
Cs	-4.61219	-0.75252	-0.09153
C	1.93427	-0.72544	-2.42944
H	1.12439	0.01058	-2.41599
C	3.08713	-0.43786	-3.34699
H	3.92029	-1.13494	-3.1708
H	3.47521	0.58112	-3.22149
H	2.78794	-0.54592	-4.40266
C	2.9694	0.87114	-0.51778
C	2.07331	1.93693	-0.33595
C	4.32161	1.22209	-0.68119
C	2.49767	3.26578	-0.29202
H	1.0077	1.72591	-0.21429
C	4.76058	2.54671	-0.63866
H	5.06481	0.43518	-0.84846
C	3.84792	3.58014	-0.43643
H	1.76721	4.06376	-0.14473
H	5.8211	2.77331	-0.76343
H	4.18327	4.61676	-0.39882

Zero-point correction= 0.450997 (Hartree/Particle)

Thermal correction to Energy= 0.491304
Thermal correction to Enthalpy= 0.492391
Thermal correction to Gibbs Free Energy= 0.369948
SCF Done: E(RwB97XD) = -2948.09204337

TS2A

C	0	0.03871	-3.39632	0.18344
C	0	0.88021	-2.15942	-0.11983
O	0	2.08334	-2.19515	0.25374
N	0	0.28564	-1.1975	-0.81061
C	0	0.96744	-0.05321	-1.15034
C	0	1.41788	0.92957	-0.19238
C	0	1.16169	0.24716	-2.4991
C	0	2.0374	2.13464	-0.65429
C	0	1.78254	1.42824	-2.93809
H	0	0.81583	-0.48522	-3.22993
C	0	1.66906	1.5775	2.00338
C	0	2.47411	3.06485	0.32022
C	0	2.2219	2.36929	-2.03585
H	0	1.91281	1.5935	-4.00779
C	0	2.30272	2.78954	1.65083
H	0	2.94608	3.99132	-0.01032
H	0	2.70229	3.293	-2.35933
H	0	2.63234	3.4794	2.42522
N	0	1.23666	0.68792	1.1362
C	0	-1.31392	-3.5236	-0.51136
H	0	1.51293	1.34932	3.0613
H	0	-1.22875	-3.0832	-1.51597
H	0	-0.08562	-3.45839	1.27693
H	0	0.67592	-4.25467	-0.08297
H	0	-1.51712	-4.59895	-0.6649
Ni	0	-2.49227	-1.06704	0.72795
P	0	-2.46616	0.8925	1.74246
C	0	-1.82164	2.31833	0.78102
H	0	-1.66383	3.19269	1.42889
H	0	-0.87708	2.05721	0.28692
H	0	-2.54962	2.58045	0.00144
C	0	-1.5226	0.94993	3.31305
H	0	-0.54108	0.48732	3.16473
H	0	-1.40187	1.97671	3.68745
H	0	-2.06397	0.35659	4.06262
C	0	-4.08364	1.56296	2.30194
H	0	-4.58689	0.83569	2.95236
H	0	-3.93924	2.49919	2.8601

H	0	-4.72973	1.76268	1.43786
Cs	0	4.61635	-0.5334	-0.09936
C	0	-2.52077	-2.92982	0.18135
H	0	-2.89649	-3.5167	1.02488
C	0	-3.52775	-2.26197	-0.61684
H	0	-4.56515	-2.38206	-0.29252
H	0	-3.42276	-2.38859	-1.69743
C	0	-3.52384	-0.37422	-0.84867
C	0	-2.66804	0.12922	-1.84585
C	0	-4.80689	0.18345	-0.74512
C	0	-3.05666	1.19112	-2.65472
H	0	-1.68134	-0.32332	-1.96639
C	0	-5.20019	1.24114	-1.56184
H	0	-5.50441	-0.21119	-0.00196
C	0	-4.32502	1.75816	-2.51646
H	0	-2.36429	1.57535	-3.40549
H	0	-6.19871	1.66567	-1.45127
H	0	-4.63388	2.58328	-3.15727
H	0	-1.69536	-1.62132	1.8857

Zero-point correction= 0.452405 (Hartree/Particle)
Thermal correction to Energy= 0.491863
Thermal correction to Enthalpy= 0.492950
Thermal correction to Gibbs Free Energy= 0.373517
SCF Done: E(RwB97XD) = -2948.06384571

TS2B

C	1.46558	-3.60166	0.01881
C	0.62679	-2.55795	0.73894
O	-0.51416	-2.90068	1.12798
N	1.1809	-1.34327	0.83002
C	0.51814	-0.3146	1.46011
C	0.55495	0.97029	0.81923
C	-0.13924	-0.39341	2.68592
C	-0.13784	2.08158	1.37359
C	-0.78623	0.7216	3.25104
H	-0.16273	-1.34837	3.20726
C	1.23925	2.22801	-1.00741
C	-0.11998	3.29495	0.64542
C	-0.81652	1.93764	2.60595
H	-1.28557	0.60663	4.21355
C	0.5427	3.36462	-0.55319
H	-0.63971	4.16309	1.0526
H	-1.33817	2.7964	3.0285
H	0.56245	4.27813	-1.14258

N	1.25988	1.08897	-0.34474
C	2.9267	-3.26062	-0.23553
H	1.81561	2.26388	-1.93276
H	3.40679	-2.93458	0.70039
H	0.96085	-3.80972	-0.94101
H	1.38481	-4.53306	0.59857
H	3.44778	-4.1859	-0.53999
Ni	2.31509	-0.51063	-0.89961
Cs	-3.12233	-1.18793	1.06388
C	3.14675	-2.22958	-1.30544
H	2.70493	-2.44662	-2.28064
C	4.23178	-1.32685	-1.24783
H	4.6058	-0.89644	-2.17844
H	5.01684	-1.51978	-0.51474
C	3.9047	0.3726	-0.18856
C	4.119	0.32005	1.19592
C	4.42829	1.45739	-0.90341
C	4.78454	1.3526	1.85015
H	3.7428	-0.52865	1.76986
C	5.09549	2.49008	-0.24684
H	4.30232	1.50093	-1.98754
C	5.27357	2.44558	1.13372
H	4.92382	1.30386	2.93069
H	5.48254	3.33258	-0.82098
H	5.79841	3.25003	1.64762
C	-4.82268	0.59739	-1.93818
C	-3.42352	0.65157	-1.96698
C	-2.7864	1.62666	-1.1981
C	-3.52147	2.51191	-0.40946
C	-4.91096	2.43805	-0.37849
C	-5.56133	1.47598	-1.15254
H	-5.33604	-0.1558	-2.54027
H	-1.69608	1.68365	-1.21479
C	-0.24585	-0.86704	-2.26166
O	1.04251	-0.81534	-2.42609
H	-0.62717	-1.5897	-1.49978
C	-1.18637	-0.16504	-2.94376
H	-0.85886	0.58161	-3.67491
C	-2.65323	-0.38899	-2.75942
H	-3.16424	-0.51135	-3.73064
H	-2.7972	-1.36322	-2.25491
H	-6.6494	1.41389	-1.14466
H	-5.48628	3.12913	0.23646
H	-3.00027	3.26467	0.18422

Zero-point correction= 0.486956 (Hartree/Particle)
Thermal correction to Energy= 0.528368
Thermal correction to Enthalpy= 0.529455
Thermal correction to Gibbs Free Energy= 0.406827
SCF Done: E(RwB97XD) = -2909.96845162

TS2C

C	0	-2.16754	-2.03147	-2.83101
C	0	-2.91341	-1.15465	-1.83368
O	0	-3.69719	-0.30418	-2.30999
N	0	-2.61659	-1.34286	-0.53787
C	0	-3.24841	-0.64504	0.46873
C	0	-2.50562	-0.48862	1.69215
C	0	-4.53951	-0.11711	0.44374
C	0	-3.07119	0.17821	2.81332
C	0	-5.09046	0.54079	1.55875
H	0	-5.12586	-0.21898	-0.4642
C	0	-0.52698	-0.88021	2.85691
C	0	-2.28017	0.2916	3.98076
C	0	-4.38161	0.70009	2.72691
H	0	-6.10552	0.93104	1.48494
C	0	-1.01479	-0.23574	4.00794
H	0	-2.69698	0.7981	4.85152
H	0	-4.80701	1.21363	3.58855
H	0	-0.38709	-0.1699	4.89321
N	0	-1.23534	-0.99284	1.74679
C	0	-1.26751	-3.14139	-2.30419
H	0	0.47058	-1.32118	2.84387
H	0	-1.8309	-3.81297	-1.63759
H	0	-1.57056	-1.34597	-3.45775
H	0	-2.92836	-2.4481	-3.50794
H	0	-0.94657	-3.75598	-3.16386
Ni	0	-0.5772	-1.73551	0.06665
Cs	0	-2.03957	2.24776	-1.11904
C	0	-0.0562	-2.61566	-1.59209
H	0	0.50518	-1.8402	-2.11873
C	0	0.57484	-3.32581	-0.54571
H	0	0.26063	-4.36051	-0.36734
H	0	-0.59432	-3.09479	0.66512
C	0	4.5191	3.7818	-0.10075
C	0	3.88445	2.64217	0.39427
C	0	4.62922	1.46407	0.50919
C	0	5.97135	1.42756	0.14955
C	0	6.59411	2.57292	-0.3473

C	0	5.86233	3.74935	-0.47395
H	0	3.95176	4.71011	-0.19253
H	0	4.13812	0.56424	0.88813
C	0	0.92226	0.74144	0.13042
O	0	0.12947	0.04414	-0.6284
H	0	1.10923	0.35881	1.15939
C	0	1.5399	1.91244	-0.18403
H	0	1.44471	2.31261	-1.20027
C	0	2.42382	2.65185	0.77979
H	0	2.10449	3.70222	0.89597
H	0	2.31668	2.18877	1.77566
H	0	6.33911	4.65002	-0.86034
H	0	7.64527	2.5462	-0.63206
H	0	6.53932	0.50295	0.25955
C	0	1.93672	-3.01141	-0.04657
C	0	2.34291	-3.52022	1.19502
C	0	2.84483	-2.2344	-0.77519
C	0	3.60875	-3.25045	1.70028
H	0	1.64229	-4.12857	1.77087
C	0	4.11543	-1.97163	-0.273
H	0	2.56471	-1.83967	-1.75097
C	0	4.50331	-2.47134	0.9677
H	0	3.90081	-3.65404	2.66924
H	0	4.8084	-1.37235	-0.86319
H	0	5.49835	-2.26108	1.35858

Zero-point correction= 0.485149 (Hartree/Particle)
Thermal correction to Energy= 0.526569
Thermal correction to Enthalpy= 0.527656
Thermal correction to Gibbs Free Energy= 0.402712
SCF Done: E(RwB97XD) = -2909.98543471
